# Affinity-Activated Protein Switches for Modular and Signal-ON Detection of Small Molecules

**DOI:** 10.1021/acs.analchem.6c02712

**Published:** 2026-08-24

**Authors:** Chien-Chi Wu, Hsin-Mei Chen, Tzu-Jung Chang, Ling-Ling Weng, Yu-Cheng Wang, Kuo-Chuan Chao, Jing-Cyun Lin, Bo-Lin Jian, Li-An Chu, Kui-Thong Tan

**Affiliations:** † Department of Chemistry, 34881 National Tsing Hua University, Hsinchu 300044, Taiwan; ‡ Department of Biomedical Engineering and Environmental Sciences, 34881 National Tsing Hua University, Hsinchu 300044, Taiwan; § Department of Biomedical Engineering, National Taiwan University, Taipei 10617, Taiwan; ∥ Department of Medicinal and Applied Chemistry, Kaohsiung Medical University, Kaohsiung 807378, Taiwan

## Abstract

Protein switches offer powerful strategies for detecting small molecules, yet current designs often suffer from limited dynamic range, lack of reversible control, and poor adaptability to nonfluorescent platforms. Here, we report a semisynthetic affinity-activated protein switch strategy that enables Signal-ON detection of small molecules across diverse environments, including live cells, *Drosophila* brain, and lateral flow assays. The switch consists of a self-labeling protein fused to a sensing protein and a synthetic probe containing a small-molecule ligand, a sterically shielded N′-3 urea nitrogen biotin derivative (N3B), and a tagging moiety. Ligand binding induces a conformational change that exposes the N3B for streptavidin-based signal output. This design achieves high dynamic range (up to 142-fold), low background, and nanomolar sensitivity for detecting sulfonamide, while demonstrating the transferability of the platform to trimethoprim detection. Reversible switching is enabled by the reduced N3B-streptavidin affinity, allowing signal reset via competitive displacement. In *Drosophila* brain, the protein switch enables spatially resolved *ex vivo* imaging of drug exposure. Furthermore, conjugation to gold nanoparticles allows robust Signal-ON colorimetric detection on lateral flow test strips. This work establishes a generalizable framework for building programmable protein switches with tunable output modes and potential diagnostic applicability.

## Graphical abstract

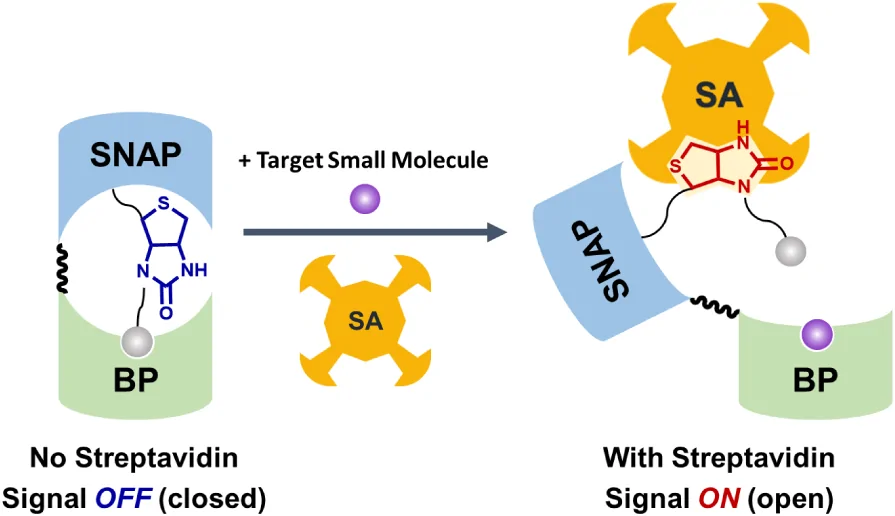

## Introduction

Small molecule-responsive protein switches are valuable tools for the selective and rapid detection of biochemical targets, with applications spanning from cellular activity monitoring to point-of-care diagnostics.^1–5^ Typically, these protein switches entail a sensing protein that undergoes allosteric changes upon binding to a specific small-molecule ligand. This binding event can activate the enzymatic activity or alter the optical properties of attached fluorescent molecules, leading to detectable signal outputs. While numerous such protein switches have been developed for the detection of small molecules such as glutamate,^6^ ATP,^7^ inositol,^8,9^ glucose,^10,11^ lactate,^12,13^ and etc.,^14^ their reliance on allosteric activation constrains the scope of suitable sensing proteins for the detection of diverse small molecules. To address this limitation, semisynthetic strategies have emerged as hybrid systems that integrate natural recognition domains with synthetic ligands. Several notable platforms, such as SNiFiT, LUCID, and CLASH, have demonstrated the feasibility of small-molecule sensing through ligand-displacement-driven switching in semisynthetic protein switches.^15–17^ In these systems, an intramolecular ligand analog is displaced by a target molecule, triggering a conformational change that activates a fluorescent dye or enzymatic activity. This modular strategy enables the engineering of customized sensing capabilities by leveraging the specificity of binding proteins and the tunability of synthetic probes. Many semisynthetic switches have been developed to detect various analytes, including sulfonamides,^15,18^ glutamate,^19,20^ γ-aminobutyric acid (GABA),^21^ and acetylcholine.^22,23^

Despite significant progress in protein switch development, several challenges remain, particularly concerning the mechanisms by which binding events are transduced into measurable signals. Fluorescence-activated protein switches that rely on changes in fluorophore emission often suffer from limited dynamic range and suboptimal photophysical properties such as photobleaching, limited brightness, and interference from cellular autofluorescence.^24–26^ These limitations hinder sensitivity and quantitative interpretation in complex biological environments. Enzyme-activated protein switches, which transduce ligand binding via enzymatic activation, introduce additional issues.^27–32^ Enzyme-generated products may diffuse away from the activation site, compromising spatial resolution and making it difficult to attribute signals to specific cellular compartments or time points. Furthermore, enzymatic activity can be possibly influenced by endogenous cellular components, leading to nonspecific background and reduced reliability. As a result, enzyme-activated protein switches are rarely employed for high-resolution or in vivo imaging applications. In parallel, most existing protein switch designs are reversible, meaning that the signal disappears once the analyte is removed. While this allows real-time monitoring, it also prevents retention of signal, making it impossible to record a “molecular memory” of transient analyte presence or concentration.^33,34^ Together, these limitations underscore the need for new protein switch strategies that offer spatially confined, tunable, and on-demand irreversible or reversible signal outputs compatible with complex biological systems.

In this paper, we present a new class of semisynthetic protein switch strategy that is based on the affinity activation of a biotin derivative under sterically gated conditions to produce Signal-ON responses upon ligand binding (Figure 1a). This design uses a genetically encoded SNAP-tag fused to a sensing domain and a synthetic probe containing a small-molecule ligand, a chemically modified N′-3 urea nitrogen biotin (N3B), and a benzylguanine (BG) moiety. Upon SNAP-tag labeling, the probe forms a closed conformation through intramolecular ligand binding, which sterically shields the N3B group and prevents streptavidin binding. When a target small molecule displaces the tethered ligand, the switch adopts an open conformation, exposing N3B for streptavidin-mediated signal generation. Instead of relying on enzyme activation or fluorophore dequenching as the signal transduction strategy, our protein switch design uses binding to streptavidin as a universal reporter. This mechanism decouples molecular recognition from conventional signal transduction methods and supports modular Signal-ON detection techniques across diverse platforms, including fluorescence imaging, fixed tissues, and instrument-free colorimetric lateral flow assays.

**Figure 1:**
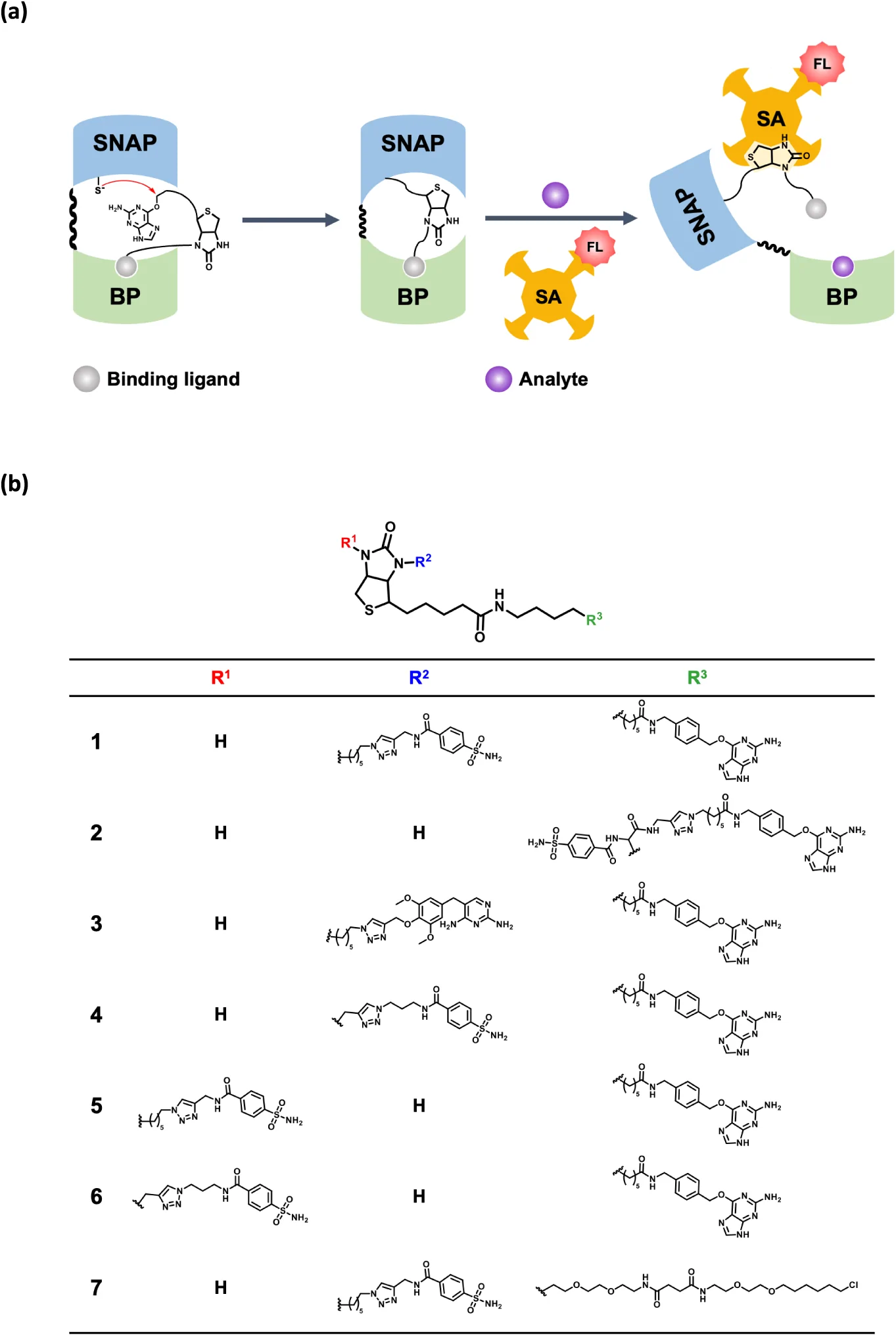
(a) Schematic illustration of the sterically gated affinity-based semisynthetic protein switch. Upon labeling with a benzylguanine (BG)-functionalized probe containing a small-molecule ligand and a biotin derivative (N3B), the protein forms a closed conformation via intramolecular binding. This sterically shields the biotin, preventing interaction with streptavidin. Upon target binding, the intramolecular ligand is displaced, converting the protein switch to an open conformation that exposes N3B for streptavidin binding and fluorescence activation. BP: binding protein. (b) Chemical structures of the N3B- and N1B-based probes (**1**-**7**) used to construct protein switches targeting sulfonamides and trimethoprim. Ligand modifications, linker lengths, and biotin substitution positions were systematically varied to optimize performance.

Relative to our previous affinity-activated protein switches, which relied on native biotin for streptavidin recognition and required reactive electrophiles for probe construction, the N3B-based design offers several distinct advantages and new capabilities.^18,35^ First, the incorporation of a self-labeling protein enables site-specific and covalent protein–probe conjugation, simplifying sensor assembly and improving compatibility with complex biological environments such as live cells and animal tissues.^36–38^ Second, the use of N3B allows on-demand reversible or irreversible imaging of small molecules, giving users precise control over the temporal dynamics and permanence of the detection signal. In the reversible mode, the switch can cycle between closed and open conformations through competitive displacement with native biotin or by the presence of the target and streptavidin. In contrast, irreversible imaging is achieved simply by omitting the biotin-displacement step, a feature that is particularly useful for snapshot readouts or recording analyte exposure history. Third, positioning the ligand on the N′-3 position of biotin increases steric shielding in the closed state, effectively suppressing nonspecific background signals and thereby enhancing detection sensitivity.

To explore the versatility and performance of this affinity-activated protein switch design, we synthesized a series of probes (**1**–**7**) with different linker architectures and ligand positions at N′-1 or N′-3 urea nitrogen of biotin (N1B or N3B) to detect sulfonamides and trimethoprim (Figure 1b). With this design, we demonstrate reversibly control binding of N3B to streptavidin and provide a general strategy to detect small-molecule targets with a very large dynamic range. We show that selective and sensitive detection can be achieved on living cell surfaces, in *Drosophila* brain tissue, and in a point-of-care (POC) diagnostic device using lateral flow assay test strips.

## Experimental Section

### DNA Transfection of MCF7 Cells and Imaging of Sulfonamides and Trimethoprim

MCF7 cells were cultured in MEM medium supplemented with 10% FBS and 1% penicillin–streptomycin. 1 × 10^5^ cells were seeded in 8-well chamber slides (Ibidi μ-slide 8 well) and cultured overnight at 37 °C with 5% CO_2_. For the transfection, 15 μL plasmid (pDisplay- SNAP-hCAII-PDGFR or pDisplay-SNAP-DHFR-PDGFR) and 4 μL Lipofectamine 2000 (Invitrogen) were added into a microcentrifuge tube containing 85 and 96 μL Opti-MEM, respectively. After 5 min, the mediums were mixed without pipetting and stood for 25 min. 25 μL of the plasmid–Lipofectamine mixture was added to a μ-slide well containing 250 μL Opti-MEM. The cells were incubated for 6 h at 37 °C, then washed with MEM and continuously cultured for 24 h at 37 °C with 5% CO_2_. The cells were labeled with 2.5 μM probe in MEM for 30 min at 37 °C. For the detection of sulfonamides, 1 μg/mL SA-Cy5 and 100 μM drugs were mixed, added to the cells, and incubated for 10 min in MEM. For the detection of trimethoprim, 1 μg/mL SA-Cy5 and 500 μM drug were mixed, added to the cells, and incubated for 30 min in MEM. After removing the excess SA-Cy5, the fluorescent images were taken by using a Laser Scanning Confocal Microscope (LSM 700, Zeiss, Germany). The reversibility tests on cell surfaces were conducted by incubating the samples with 50 μM biotin for 10 min to displace the SA-Cy5. Following a wash step to remove the free biotin, the cells were incubated with 100 μM EZA and 1 μg/mL SA-Cy5 for 10 min to trigger the open conformation and label the exposed N3B. The cells were then washed again prior to imaging. For SA-Cy5, images were taken by using a 639 nm excitation laser and an LP640 emission filter. For Hoechst, a 405 nm excitation laser and an SP490 emission filter were used. The fluorescence intensities were quantified by ImageJ software. The titration curves were fitted to a sigmoidal dose–response equation, y = A2 + (A1 – A2)/(1 + 10^(x – log­(x0))), using Origin software.

### Preparation of Fly Stocks and Detection of EZA in *Drosophila* Brain

The following transgenic *Drosophila* lines were used in this study: (1) OK107-Gal4 and (2) pUAST-SNAP-hCAII-PDGFR. Flies were raised and maintained at 25 °C and 50% relative humidity (RH) for 5–7 days prior to experimentation. Fly brains were dissected in phosphate-buffered saline (PBS, pH 7.2) and immediately transferred to a 24-well plate containing probe **1** (2.5 μM) for 30 min. The brains were then transferred to a second well containing 100 μM EZA and 0.5 μg/mL streptavidin-Alexa 633 (SA-Alexa 633) for an additional 40 min. After incubation, samples were fixed in 4% paraformaldehyde in PBS for 30 min. To enhance fixation efficiency, the plate was placed under vacuum for 10 min to facilitate penetration into the brain tissue. After releasing the vacuum, samples were held at atmospheric pressure for 3 min to expel air from the tracheal system. This vacuum–release cycle was repeated four times. Following fixation, brains were permeabilized and blocked overnight at 4 °C in PBS containing 2% Triton X-100 and 10% normal goat serum (NGS). Samples were then cleared and mounted in FocusClear (CelExplorer, Taiwan) and imaged using a Zeiss LSM 780 confocal microscope equipped with a 40× C-Apochromat water-immersion objective. Stacks of optical slices with a thickness of 2 μm were acquired at a resolution of 1024 × 1024 pixels. There was a 50% overlap between any two consecutive slices in order to ensure comprehensive coverage of the sample.

### Preparation of Lateral Flow Assay Test Strips

The control and test lines were coated on the membranes by a rapid test printer (Regabio Inc., Taiwan). 0.5 mg/mL alkaline phosphatase-conjugated streptavidin (SA-ALP) and 0.25 mg/mL goat antimouse IgG were used to coat the test line and control line, respectively. The SA-ALP conjugate was utilized at the test line instead of unmodified streptavidin to enhance protein immobilization on the nitrocellulose membrane. After coating, the membranes were incubated at 37 °C for 30 min to remove extra moisture. The length and width of a test strip were 5.9 and 0.35 cm, respectively.

### Preparation of Citrate-Capped Gold Nanoparticles (**Au@citrate**)

The gold nanoparticles were prepared by using the sodium citrate reduction method (Turkevich method). A stirred solution of chloroauric acid (1 mM) in 50 mL H_2_O was heated until it was boiled vigorously. A trisodium citrate solution (38.8 mM) was added to the reaction mixture and heated for 10 min. The AuNP solution was cooled to room temperature and stored at 4 °C until further use. Based on UV–vis analysis and TEM imaging, the synthesized **Au@citrate** nanoparticles exhibited a uniform spherical morphology with an average diameter of approximately 15 nm.^39^

### Preparation of Mouse IgG-Conjugated Gold Nanoparticles (**Au@control**)

1 mL (Ab*s*_max_ = 520 nm) **Au@citrate** solution was transferred to a microcentrifuge tube and K_2_CO_3_ was added to the microcentrifuge tube to adjust the pH value of the AuNP solution to 9. The AuNPs were functionalized with mouse IgG through physical adsorption at 25 °C for 20 min with 200 rpm stirring. The solution was centrifuged at 12,000 rpm for 15 min. The supernatant was removed. 150 μL of conjugate buffer solution (5 mM sodium borate, 0.1% BSA, 0.15% Tween 20, 5% d-sucrose) was added to the pellet and **Au@control** was stored at 4 °C until further use.

### Preparation of **Au@SNAP-hCAII-1** and **Au@halo-HCAII-7** Gold Nanoparticle Conjugates

2.5 μM protein (**SNAP-hCAII or halo-hCAII**) and 25 μM probe (**1 or 7**) were added to 100 μL of deionized water and incubated at room temperature with shaking at 720 rpm for 2 h to form the **SNAP-hCAII-1 or halo-hCAII-7** complex. 500 μL of **Au@citrate** (Ab*s*_max_= 520 nm) solution was transferred to a microcentrifuge tube. The solution was centrifuged at 5000 × *g* for 30 min. The supernatant was removed, and the pellet was resuspended in 50 μL of deionized water. **SNAP-hCAII-1** or **halo-hCAII-7** was then added to the **Au@citrate** solution and incubated at room temperature with shaking at 720 rpm for 1 h. The reaction mixture was subsequently blocked with 15 μM BSA for 15 min. The reaction mixture was then centrifuged at 10,000 rpm for 15 min, and the supernatant was removed. The pellet was washed with 100 μL of deionized water and centrifuged again at 10,000 rpm for 15 min. The supernatant was removed, and the **Au@SNAP-hCAII-1** and **Au@halo-hCAII-7** pellet was resuspended in Tris buffer (50 mM) to a final volume of 100 μL and stored at 4 °C until further use.

### Detection of Sulfonamides with a Lateral Flow Assay

For the selectivity test, 5 μL **Au@halo-hCAII-7** and 1 μL **Au@control** were spotted onto the conjugate pad of the test strips and dried at 37 °C for 30 min before use. Testing chemicals (10 μM or 100 μM) were added to Tris buffer (50 mM containing 125 μM BSA) to a final volume of 100 μL. The dried test strips were then immersed in the reaction mixture for 15 min. For the sensitivity test, the reaction mixture was prepared by adding 2.5 μL of BSA (5 mM) and different concentrations of EZA were added to the Tris buffer to a final volume of 100 μL. Then, the dried test strips were immersed in the reaction mixture for 15 min. The LFA test strip images were captured using a handphone camera. The test line intensities were recorded using the ChemiDoc Touch Imaging System (Biorad Inc., CA, USA) and analyzed using Image Lab software (Biorad Inc., CA, USA). The limits of detection (LODs) were determined using the formula LOD = 3σ/slope, where σ represents the standard deviation of the blank matrix (N = 10).

## Results and Discussion

### Affinity-Activated Protein Switches Enable Signal-ON Imaging of Small Molecules in Living Cells

The plasma membrane represents a critical microenvironment for small-molecule detection, as dynamic concentration changes of signaling molecules such as hormones and neurotransmitters directly modulate key physiological processes including proliferation, differentiation, stress responses, and apoptosis.^40,41^ To demonstrate the feasibility of our affinity-activated protein switch design in this context, we demonstrated the detection of ethoxzolamide (EZA), a sulfonamide drug, on the surface of MCF7 cells.^42^ The cells were transiently transfected with SNAP-hCAII-PDGFR, a recombinant protein construct in which the SNAP-tagged human carbonic anhydrase II (hCAII) is anchored to the membrane via the platelet-derived growth factor receptor (PDGFR) transmembrane domain. These cells were subsequently labeled with probe **1**, a synthetic molecule featuring a para-substituted sulfonamide ligand at N3B and a benzylguanine (BG) moiety for covalent attachment to the SNAP-tag. This labeling yields the semisynthetic **SNAP-hCAII-1** protein switch. To assess the importance of steric regulation via urea nitrogen modification, we compared **SNAP-hCAII-1** to a control protein switch, **SNAP-hCAII-2**, which was generated using probe **2**, a tridentate sulfonamide, and a BG derivative containing an unmodified biotin moiety. After removal of excess probe, cells were incubated with Cy5-labeled streptavidin (SA-Cy5), either in the presence or in the absence of EZA, and imaged via fluorescence microscopy.

In the absence of EZA, **SNAP-hCAII-1** remained in the closed conformation, exhibiting minimal Cy5 fluorescence due to steric shielding of the N3B biotin moiety (Figure 2a). Upon addition of 100 μM EZA, a robust ≈85-fold increase in fluorescence was observed, indicating a ligand-triggered conformational switch that exposed biotin and enabled streptavidin binding. In contrast, **SNAP-hCAII-2** exhibited high background fluorescence in the absence of EZA and only a modest 2-fold increase upon target addition. Because the unmodified native biotin remains flexible and exposed outward from the protein surface, it is not effectively shielded by the protein matrix. This exposure allows streptavidin to bind even in the absence of the target drug, thereby explaining the high background fluorescence of **SNAP-hCAII-2** without EZA and confirming that the N3B linkage is mandatory for effective steric gating. In addition to live-cell applications, **SNAP-hCAII-1** is also compatible with fixed-cell imaging, allowing postfixation detection of small-molecule exposure (Figure S1). Since the conformational switch irreversibly exposes biotin upon ligand binding, the activated signal can be locked in prior to fixation, enabling robust spatial readout without signal loss during processing. This feature is particularly advantageous for high-resolution microscopy and long-term sample storage where real-time detection is not feasible. We also expressed an “always-open” control construct, SNAP-CFP, which contains a cyan fluorescent protein (CFP) instead of the hCAII sensing domain. When labeled with probe **1** and treated with SA-Cy5, this construct produced strong cell-surface fluorescence even in the absence of the target drug. This demonstrates that the N3B moiety remains completely accessible to streptavidin when a matching intramolecular binding pocket is absent (Figure S2).

**Figure 2:**
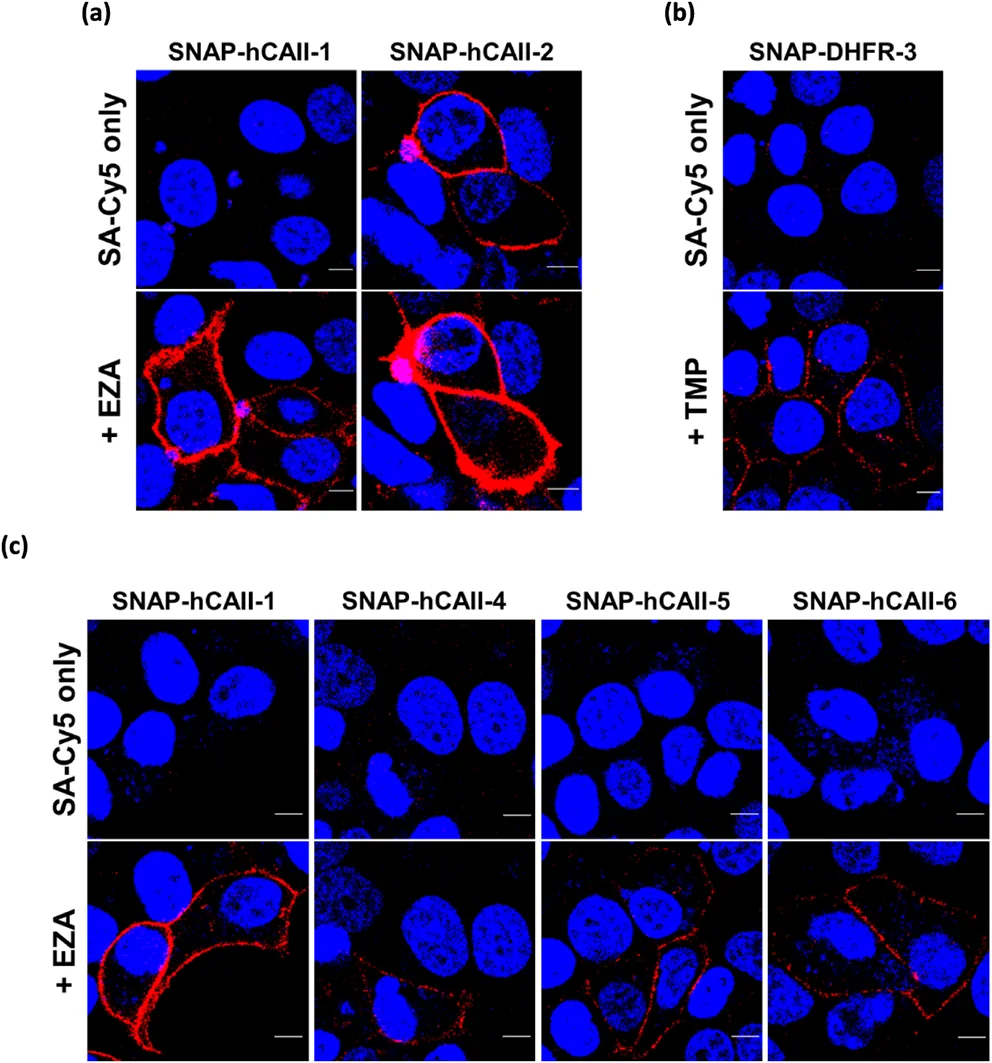
Signal-ON imaging of small molecules in living cells. (a) Fluorescence imaging of MCF7 cells expressing **SNAP-hCAII-1** and **SNAP-hCAII-2** constructs treated with or without 100 μM ethoxzolamide (EZA). Signal is observed only in the presence of EZA due to conformational switching. (b) Detection of 500 μM trimethoprim (TMP) using the **SNAP-DHFR-3** protein switch demonstrates generalizability of the design to other protein–ligand systems. (c) Optimization of steric hindrance using different probe designs (probes **4**–**6**). Fluorescence responses of protein switches with N1B or N3B modifications upon EZA addition show varied dynamic ranges and confirm the importance of linker architecture and substitution site. Scale bar: 20 μm.

To evaluate the generalizability of our affinity-based protein switch design, we further constructed **SNAP-DHFR-3**, a protein switch targeting trimethoprim (TMP) based on the interaction between TMP and dihydrofolate reductase (DHFR).^43^ In this protein switch, only minimal background fluorescence was detected with SA-Cy5. On the other hand, the addition of 500 μM TMP induced a clear fluorescence signal, providing a preliminary proof of concept that demonstrates the structural transferability of our affinity-activated platform to alternative protein–ligand systems (Figure 2b). In general, the protein switch performance is strongly dependent on the properties and binding orientation of the chosen ligand–protein pair, and further probe design and linker optimization will be required to achieve high sensitivity and full quantitative validation for TMP and related targets.

### Steric Tuning and Optimization of Affinity-Activated Protein Switch Performance

To further investigate the structural determinants that govern signal suppression in the closed and the open states upon ligand binding, we synthesized three additional probes (**4**, **5**, and **6**) with variations in the position and linker length of the sulfonamide ligand on the biotin scaffold. These included substitutions at either the N1B or N3B positions and alternative triazole linker orientations. The corresponding protein switches, **SNAP-hCAII-4**, **-5**, and **-6**, were generated and evaluated alongside **SNAP-hCAII-1** in MCF7 cells. All variants formed closed conformations that effectively suppressed streptavidin binding, exhibiting low background fluorescence in the absence of EZA (Figure 2c). Upon addition of 100 μM EZA, each protein switch showed pronounced activation and significant fluorescence on the cell surface, with dynamic ranges of approximately 85-fold (**SNAP-hCAII-1**), 39-fold (**SNAP-hCAII-4**), 142-fold (**SNAP-hCAII-5**), and 53-fold (**SNAP-hCAII-6**) (Figure S3). While blocking either the N3B or N1B urea nitrogen provides a viable structural basis for suppressing background signals, the specific attachment site and linker geometry significantly dictate overall switch dynamics. Specifically, probe **5** utilizes an N1B modification combined with a proximal triazole linker. In the closed state, this configuration drives a highly compact, shielded conformation that yields an exceptionally low background, allowing it to achieve the highest dynamic range of 142-fold. However, this restricted N1B geometry ultimately compromises its spatial accessibility to streptavidin in the activated open state, resulting in a lower absolute signal intensity. Conversely, probe **1**, which features an N3B substitution site and a distal triazole orientation from the biotin core, effectively balances background-suppressing steric hindrance in the closed state with optimal unmasking upon target binding. Although its background is marginally higher than probe **5**, it yields the strongest absolute signal intensity in the open state. Consequently, **1** was selected as our lead candidate for subsequent experiments due to its optimal combination of low background and strong activation.

### Selectivity, Sensitivity, and Reversible Imaging of EZA in Live Cells

To validate the specificity of the **SNAP-hCAII-1** protein switch, we examined its ability to distinguish the target compound ethoxzolamide (EZA) from structurally related and unrelated small molecules. Three sulfonamide ligands with known hCAII binding affinities, EZA (K_d_ ≈ 0.5 nM), acetazolamide (AZA, K_d_ ≈ 7 nM), and methazolamide (MZA, K_d_ ≈ 7 nM), were tested alongside four nontarget molecules: indomethacin, folic acid, adenosine, and γ-aminobutyric acid (GABA). Fluorescence microscopy revealed strong signal activation for the sulfonamides, with signal intensity correlating with their binding affinity to hCAII (Figure 3a and Figure S4). No detectable activation was observed for the nontarget compounds, confirming the high target selectivity of the protein switch. We next evaluated the sensitivity of **SNAP-hCAII-1** by titrating EZA and AZA from nanomolar to micromolar concentrations. The protein switch responded robustly to EZA at concentrations as low as 0.1 μM, with a calculated limit of detection (LOD) of approximately 113 nM (Figure 3b and Figure S5). For AZA, signal onset occurred at about 5 μM, yielding an LOD of approximately 8.04 μM. The binding isotherms (K_d_) of EZA and AZA with **SNAP-hCAII-1** were estimated to be 6.01 × 10^–6^ M and 5.31 × 10^–5^ M, respectively. The very low background obtained using this protein switch design should help to deliver higher accuracy and precision to analyze target molecules at low concentrations. Another key advantage of this sensing platform is its compatibility with a wide range of fluorescent dyes. In addition to SA-Cy5, streptavidin conjugated to Cy3 and Alexa Fluor 488 dyes also produced clear fluorescence upon EZA activation, enhancing the versatility of the system for different imaging and detection modalities (Figure S6).

**Figure 3:**
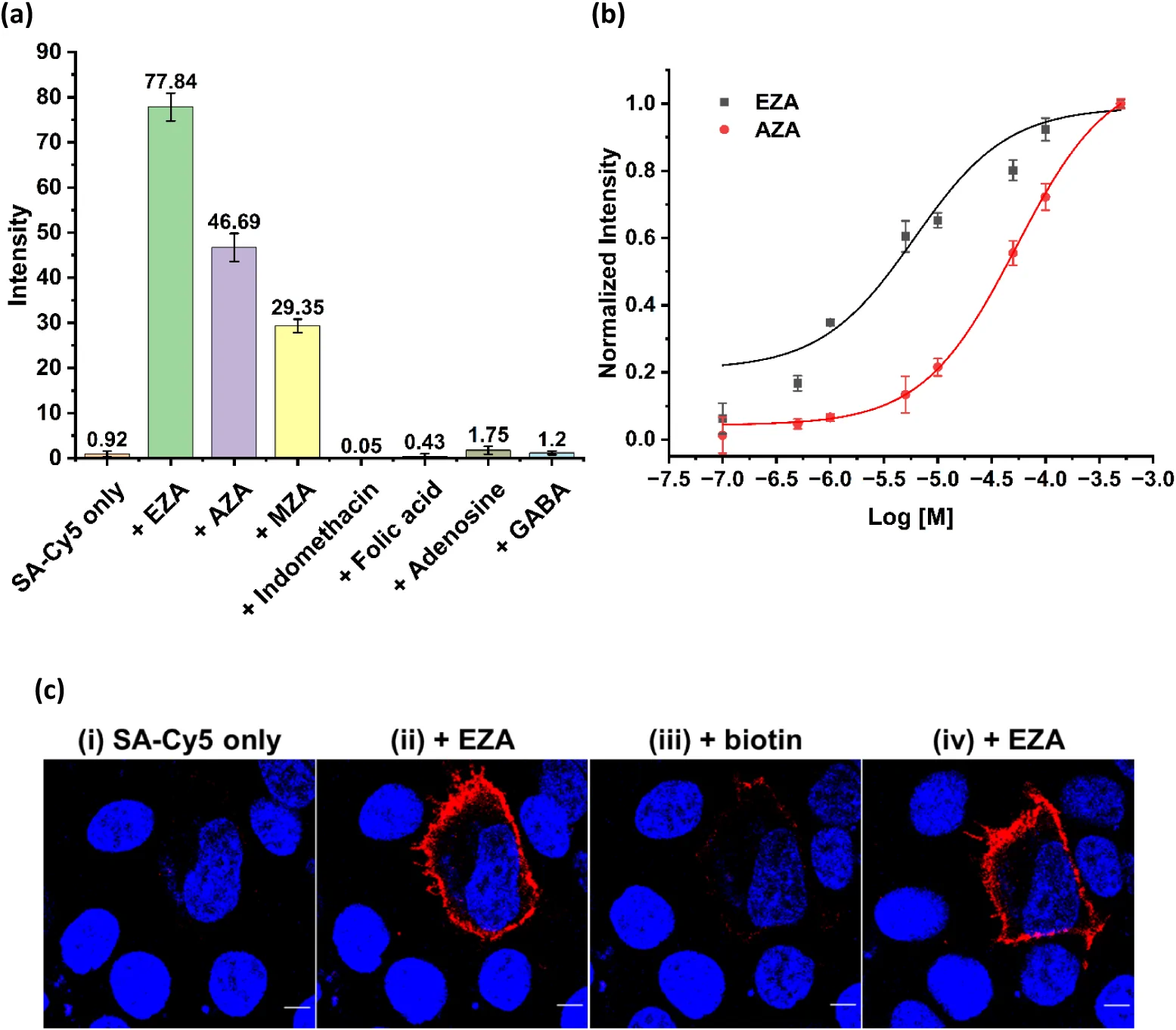
Selectivity, sensitivity, and reversible imaging of EZA in live cells. (a) Selectivity analysis of **SNAP-hCAII-1** with structurally related sulfonamides (EZA, AZA, MZA, 100 μM each) and unrelated small molecules (indomethacin, folic acid, adenosine, GABA, 100 μM each). (b) Dose-dependent fluorescence response of **SNAP-hCAII-1** to EZA and AZA. (c) Reversible imaging demonstrated by sequential activation and deactivation of **SNAP-hCAII-1** using EZA and native biotin, respectively: (i) SA-Cy5 only, (ii) addition of 100 μM EZA and SA-Cy5, (iii) incubation with 50 μM biotin, (iv) washing followed by the reintroduction of 100 μM EZA and SA-Cy5. Fluorescence is restored upon reintroduction of EZA, confirming on-demand imaging and switching behavior. Scale bar: 20 μm.

The concentrations of many small molecules in living organisms change rapidly as a result of cellular metabolism and neurotransmission.^44,45^ Therefore, it is very important that a protein switch possesses the ability to reversibly sense the small-molecule target. Previous affinity-based detection methods using native biotin for streptavidin binding restrict applications to a single end-point measurement due to the very strong streptavidin and native biotin interaction (K_d_ = 10^–14^ M).^18,35^ To assess protein switch reversibility which is a critical feature for dynamic sensing, the binding affinity between the modified biotin and streptavidin was measured using isothermal titration calorimetry (ITC), yielding a dissociation constant of ≈5.63 × 10^–7^ M (Figure S7). This weaker affinity compared to native biotin enables reversible displacement. Indeed, **SNAP-hCAII-1** treated with EZA and SA-Cy5 exhibited strong membrane fluorescence, which could be erased upon treatment with 50 μM native biotin. Reapplication of EZA and SA-Cy5 regenerated the fluorescence signal, demonstrating reversible and reproducible switching behavior (Figure 3c and Figure S8). In contrast, the **SNAP-hCAII-2** construct fails to undergo signal reversal, indicating that the weaker biotin derivative in N3B is required to enable reversible streptavidin engagement (Figure S9). While this “snapshot” reversibility allows for repeated sensing cycles on the same sample, we recognize that fully exploiting this feature for continuous, time-resolved monitoring of fluctuating analyte concentrations would require further optimization of reporter exchange and temporal control. In summary, these results collectively highlight the high specificity, tunable sensitivity, and reversibility of the affinity-based switch system for live-cell applications.

### Affinity-Activated Protein Switch Enables *Ex Vivo* Small-Molecule Detection in *Drosophila* Brain Tissue

The ability to visualize small-molecule activity within intact brain tissue is critical for studying neuromodulation and disease pathophysiology.^46–48^ However, traditional genetically encoded sensors often suffer from a limited signal-to-noise ratio and restricted tissue penetration.^49,50^ To evaluate the applicability of our protein switch in a neural context, we generated a transgenic *Drosophila* line in which the SNAP-hCAII-PDGFR construct was inserted downstream of a UAS promoter via CRISPR-mediated genomic integration. This transgene was selectively expressed in the mushroom body, a key center for olfactory learning and memory, by crossing it to the OK107-Gal4 driver line. The mushroom body’s well-characterized connectivity, functional relevance, and genetic accessibility make it an ideal target for probing neuromodulatory signaling in behaving animals.^51^

To activate the protein switch in fly brain tissue, a small window was cut at the top of the fly brains and a drop of PBS-based solution containing 2.5 μM of probe **1** was added and incubated for 30 min. This was followed by incubation with 0.5 μg SA-Alexa633, with or without 100 μM EZA, for an additional 40 min. The tissues were then fixed and imaged via confocal microscopy. In the EZA-treated samples, we observed strong and spatially resolved fluorescence signals in the mushroom body region, confirming successful activation of the protein switch and streptavidin binding (Figure 4). Three-dimensional image stacks further corroborated selective signal generation in the presence of EZA (Video S1). By contrast, brains not treated with EZA displayed negligible background fluorescence, supporting the steric suppression mechanism of the open and closed switch conformations. These results demonstrate that our affinity-based switch can function in a native tissue environment and can be genetically targeted to defined neuronal populations. It is important to clarify that the imaging of sulfonamides in cellular and brain tissues is intended as a technological validation of the new switch architecture, rather than as a claim of direct physiological relevance regarding specific neurochemical targets.

**Figure 4:**
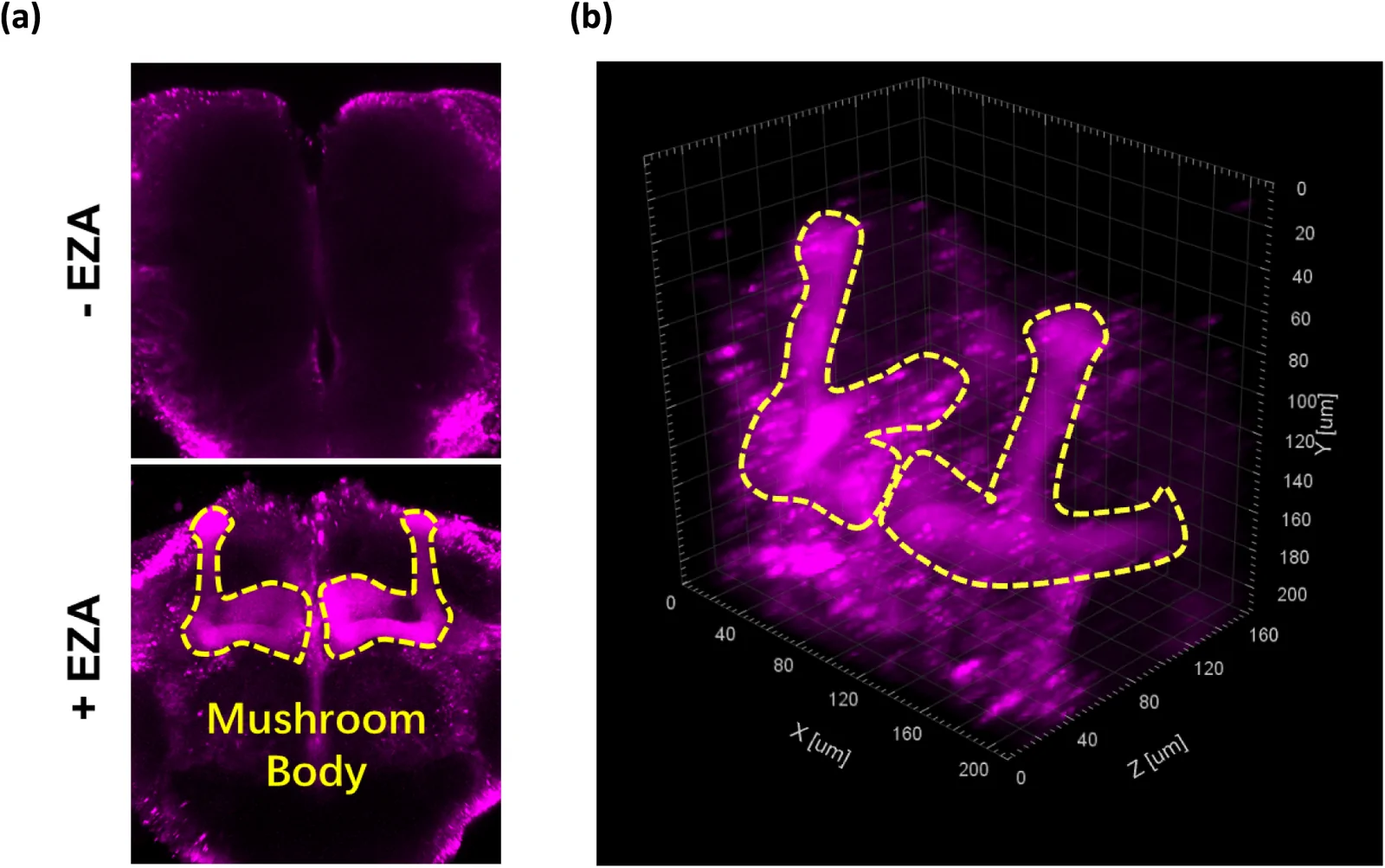
Affinity-based protein switch enables small-molecule detection in *Drosophila* brain tissue. (a) Confocal imaging of *Drosophila* brain tissue expressing **SNAP-hCAII-PDGFR** labeled with probe **1** and treated with or without 100 μM EZA. The brain tissues were coincubated with 0.5 μg/mL SA-Alexa 633. (b) 3D reconstruction illustrates three-dimensional localization of protein switch activation in the brain, highlighting spatial precision and imaging capability in complex tissue environments.

### Signal-ON and Rapid Colorimetric Sulfonamide Sensing Using a Protein Switch Lateral Flow Assay

Building on the successful application of our sterically gated protein switch for fluorescence imaging of sulfonamides in live cells and *Drosophila* brain tissue, we next adapted the protein switch design for point-of-care (POC) diagnostics using lateral flow assay (LFA) technology. LFAs are widely used for their speed, simplicity, and instrument-free operation, with applications ranging from pregnancy tests (hCG) and COVID-19 antigen detection to small-molecule analysis in food safety and drug testing.^52–54^ However, small-molecule detection on LFAs poses a persistent challenge due to the monovalent nature of these targets, which precludes the use of classical signal-on sandwich formats commonly employed for macromolecules like proteins and oligonucleotides. As a result, most small-molecule LFAs rely on competitive binding formats that produce signal-off outputs, which are often less intuitive to interpret and typically suffer from reduced sensitivity.^55–57^ The mismatch in signal behavior between macromolecule and small-molecule assays can potentially lead to misinterpretation by untrained users, especially in the absence of clear reference guidance. In general, fluorescence- or enzyme-activated protein switches are not readily compatible with LFA platforms, as they lack a universal binding element to couple molecular recognition to a signal output in a surface-immobilized format.^58^ However, notable exceptions exist, such as CRISPR-based RNA and DNA diagnostics, which harness enzyme-mediated collateral cleavage to enable lateral flow readouts.^59,60^

To overcome the fundamental Signal-OFF problem in rapid small-molecule sensing, we developed a sterically gated protein-switch LFA that enables direct Signal-ON colorimetric detection of small molecules. In this design, the protein switch is conjugated to gold nanoparticles (AuNPs), and upon ligand binding, undergoes a conformational change that exposes the N3B moiety. The exposed N3B is then captured by streptavidin immobilized on the test line, producing a visible red signal from the accumulated AuNPs (Figure 5a). We tested this approach using two protein switch constructs: **SNAP-hCAII-1**, previously validated in live-cell studies, and **HaloTag-hCAII-7**, designed to demonstrate compatibility with alternative self-labeling protein tags. Both constructs were successfully conjugated to AuNPs via passive adsorption, forming **Au@SNAP-hCAII-1** and **Au@Halo-hCAII-7**. UV–vis spectroscopy confirmed a gradual bathochromic shift in the absorption maximum (λ_max_) with increasing functionalization, indicating successful surface coating (Figure S10).^61^

**Figure 5:**
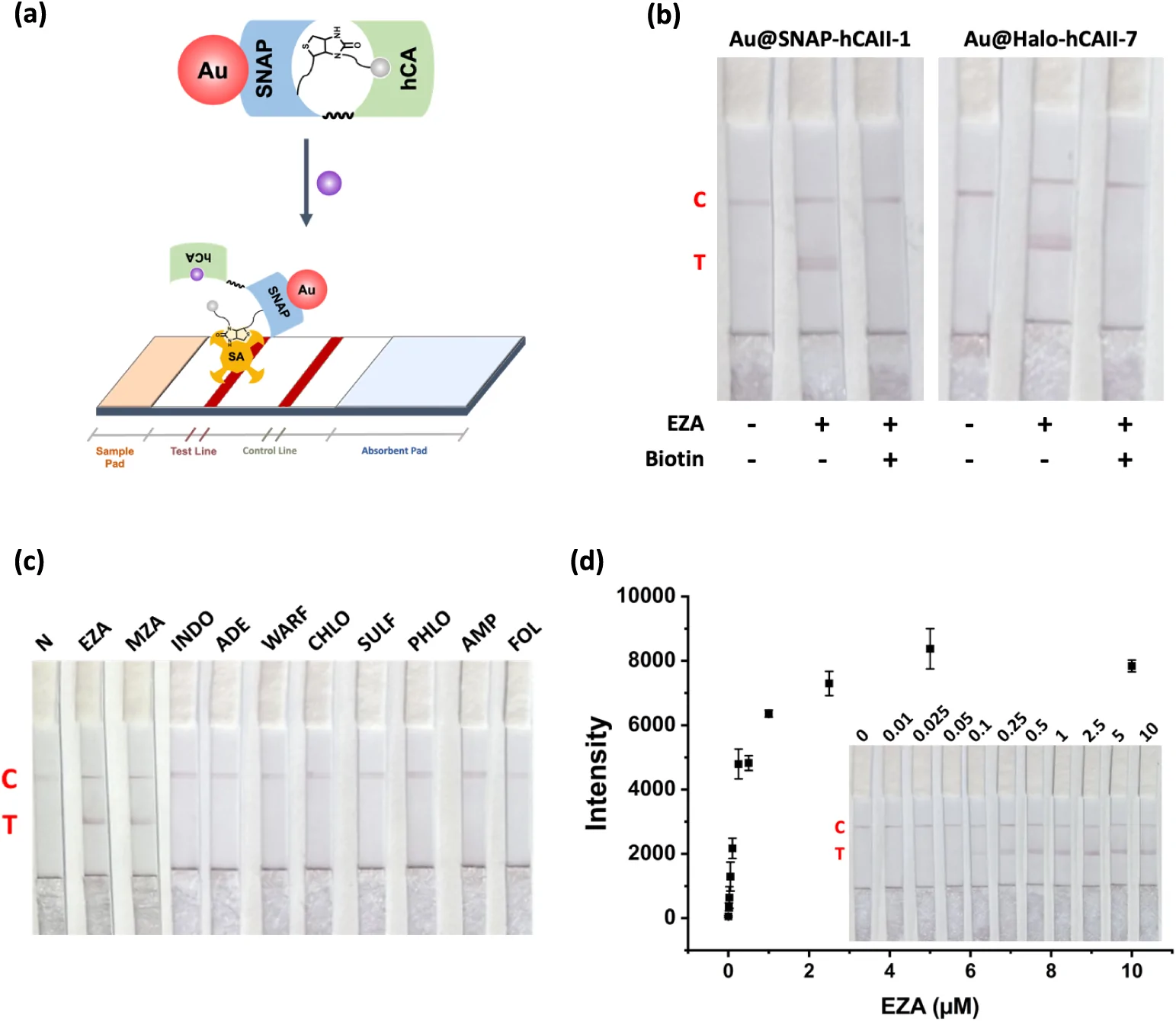
Signal-ON testing of sulfonamides using protein switch-conjugated lateral flow assays. (a) Schematic illustration of the protein switch LFA. **Au@SNAP-hCAII-1** or **Au@Halo-hCAII-7** undergoes a ligand-induced conformational change upon sulfonamide binding, exposing the biotin derivative (N3B) for streptavidin capture at the test line (T). (b) Visual results of LFA strips incubated with 10 μM EZA, 100 μM biotin, or no ligand. (c) Selective testing of sulfonamide drugs (10 μM) and other nontarget analytes (100 μM each) using **Au@Halo-hCAII-7**. (d) Quantitative analysis and images of test strips (inset) after incubating **Au@Halo-hCAII-7** with various concentrations of EZA. EZA, ethoxzolamide; AZA, acetazolamide; MZA, methazolamide; INDO, indomethacin; ADE, adenosine; WAR, warfarin; CHLO, chloramphenicol; SULF, sulfamethazine; PHLO, phloroglucinol; AMP, ampicillin; FOL, folic acid.

Upon addition of 10 μM EZA, the LFA test strips produced a clear test line signal (T), indicative of successful activation of the protein switch (Figure 5b). In contrast, samples lacking EZA or supplemented with 100 μM biotin displayed only a control line signal (C), confirming that activation is specific to EZA and dependent on the sterically gated exposure of the N3B-modified biotin. Further validation revealed that the protein switch LFA selectively responds to sulfonamide drugs, with strong signals observed for 10 μM of EZA and MZA but not for unrelated small molecules at 100 μM (Figure 5c and Figure S11). With regard to the precision of sulfonamide detection, the relative standard deviation (RSD) was determined to be approximately 1.47% (negative) and 1.77% (positive) (Figure S12). These results demonstrate the feasibility of deploying the affinity-based protein switch as a universal Signal-ON approach for rapid small-molecule sensing in POC platforms with good precision and high specificity. Furthermore, we performed a preliminary stress test to evaluate the thermal stability of the unsealed test strips under accelerated aging conditions at 55 °C. The assay maintained its performance for 3 days before showing signs of gradual degradation (Figure S13).

To assess detection sensitivity, decreasing concentrations of EZA were applied to the LFA test strips. The titration experiments yielded a visual limit of detection (vLOD) of approximately 25 nM (6.5 ppb) and a theoretical LOD of 12 nM (3.1 ppb), with a linear range from 0 to 250 nM (Figure 5d and Figure S14). This integrated protein switch design not only improves detection sensitivity but also reduces sensing protein consumption to 2.5 μM, compared to the previous strategy which required 60 μM of sensing protein to block the biotin interaction. Reducing protein consumption is critical for future practical applications, as most current lateral flow assays (LFAs) rely on antibodies which are expensive to produce. Additionally, this approach offers a simplified testing procedure by eliminating the need for external protein reagents.^35^ Importantly, this LFA strategy maintained strong colorimetric performance in spiked complex matrices like milk and blood serum, underscoring its general robustness (Figure S15). These results highlight the modularity and practical potential of the protein switch LFA for rapid, specific, and intuitive detection of small molecules. However, it should be noted that while the platform performs well in these complex environments, the current setup serves as a fundamental technological validation of the Signal-ON mechanism rather than an immediate field-ready tool for broad regulatory diagnostics. Nevertheless, this work addresses a long-standing limitation of small-molecule rapid assays by introducing a highly adaptable platform that successfully harnesses ligand-induced biotin exposure as a general Signal-ON transduction mechanism.

## Conclusions

We have developed a semisynthetic affinity-based protein switch platform that enables reversible and irreversible Signal-ON detection of small molecules with high specificity, sensitivity, and modularity. While conceptually inspired by ligand-displacement-driven switching, our design introduces a fundamentally distinct signal transduction mechanism based on affinity activation. In this strategy, target binding induces steric unmasking of a chemically modified biotin (N3B), triggering a detectable signal through streptavidin recognition. The system overcomes long-standing limitations of fluorescence- and enzyme-based sensing strategies, particularly in small-molecule detection. Our design is broadly adaptable to various protein–ligand pairs and supports diverse detection modalities, including live-cell and tissue imaging, as well as colorimetric readouts on lateral-flow assay devices. Compared to our previous native biotin-based protein switch, the N3B design offers key improvements, including reversible sensing, enhanced detection sensitivity, and reduced protein consumption.^18,35^ While the requirement for specific ligand-binding domains currently represents a practical bottleneck, the emergence of machine-learning-based protein design is expected to significantly expand the library of accessible targets. Furthermore, we acknowledge that the two-component nature of this system, requiring both the protein switch and a streptavidin-based reporter, introduces stoichiometric complexities that can influence signal output. While we have utilized genetically encoded tags to localize the protein switch, precise control over component ratios may remain application-dependent and could limit certain in vivo implementations. In general, this work not only advances the design principles of protein-based sensors but also introduces a versatile and practical strategy for integrating molecular recognition with robust signal generation across research and diagnostic platforms.

## Supplementary Material





## Notes

Probe synthesis, fluorescent imaging, gold nanoparticle preparation, LFA test strip images, quantitative analysis, and NMR and HRMS spectra (PDF) (PDF)
Video S1: Orthogonal slicing of a 3D image reconstruction showing spatial localization of protein switch activation in the mushroom body region of the *Drosophila* brain (MP4)
