# Supplementary material for: Affinity-Activated Protein Switches for Modular and Signal-ON Detection of Small Molecules
Source: Anal Chem. 2026 Aug 24;98(35):25741–51. doi: 10.1021/acs.analchem.6c02712 (PMC13564102; doi:10.1021/acs.analchem.6c02712)
Supplement: Supplementary file 1 [file ac6c02712_si_001.pdf]

Supporting Information

Affinity-Activated Protein Switches for Modular and Signal-ON Detection of Small Molecules

Chien-Chi Wu,[†,§] Hsin-Mei Chen,[†,§] Tzu-Jung Chang,[†] Ling-Ling Weng,[†] Yu-Cheng Wang,[†] Kuo-Chuan Chao,[‡] Jing-Cyun Lin,[†] Bo-Lin Jian,[†] Li-An Chu,[‡,∥,*] and Kui-Thong Tan[†,#,*]

† Department of Chemistry, National Tsing Hua University, Hsinchu 300044, Taiwan

‡ Department of Biomedical Engineering and Environmental Sciences, National Tsing Hua University, Hsinchu 300044, Taiwan

∥ Department of Biomedical Engineering, National Taiwan University, Taipei 10617, Taiwan

Department of Medicinal and Applied Chemistry, Kaohsiung Medical University, Kaohsiung 807378, Taiwan

§ C.-C. W. and H.-M. C. contributed equally to this work.

Corresponding author: kttan@mx.nthu.edu.tw and lachu@ntu.edu.tw

Table of Content

Materials and instruments....7
Figure S1....8
Figure S2....9
Figure S3....10
Figure S4....11
Figure S5....12
Figure S6....13
Figure S7....14
Figure S8....15
Figure S9....16
Supplementary Video S1....17
Figure S10....18
Figure S11....19
Figure S12....20
Figure S13....21
Figure S14....22
Figure S15....23
Scheme S1....24
Scheme S2....25
Scheme S3....26
Scheme S4....27
Scheme S5....28
Scheme S6....29
Scheme S7....30
Synthesis of Compound 10....31
Synthesis of Compound 11 and 12....31
Synthesis of Compound 13 and 14....31
Synthesis of Compound 15 and 16....32
Synthesis of Compound 17....32

Synthesis of Compound 18 33
Synthesis of Compound 19 33
Synthesis of Compound 20 33
Synthesis of Compound 21 34
Synthesis of Compound 22 34
Synthesis of Compound 23 35
Synthesis of Compound 24 35
Synthesis of Compound 25 35
Synthesis of Probe 1 36
Synthesis of Compound 32 36
Synthesis of Probe 3 37
Synthesis of Compound 33 and 34 37
Synthesis of Compound 35 and 36 37
Synthesis of Compound 37 38
Synthesis of Compound 38 38
Synthesis of Probe 4 39
Synthesis of Compound 39 39
Synthesis of Probe 5 40
Synthesis of Compound 40 41
Synthesis of Probe 6 41
Synthesis of Compound 41 42
Synthesis of Compound 42 42
Synthesis of Compound 43 42
Synthesis of Compound 44 43
Synthesis of Compound 45 43
Synthesis of Compound 46 43
Synthesis of Compound 47 44
Synthesis of Compound 48 44
Synthesis of Probe 7 44
Figure S16 46

Figure S17..47
Figure S18..48
Figure S19..49
Figure S20..50
Figure S21..51
Figure S22..52
Figure S23..53
Figure S24..54
Figure S25..55
Figure S26..56
Figure S27..57
Figure S28..58
Figure S29..59
Figure S30..60
Figure S31..61
Figure S32..62
Figure S33..63
Figure S34..64
Figure S35..65
Figure S36..66
Figure S37..67
Figure S38..68
Figure S39..69
Figure S40..70
Figure S41..71
Figure S42..72
Figure S43..73
Figure S44..74
Figure S45..75
Figure S46..76

Figure S47.....77
Figure S48.....78
Figure S49.....79
Figure S50.....80
Figure S51.....81
Figure S52.....82
Figure S53.....83
Figure S54.....84
Figure S55.....85
Figure S56.....86
Figure S57.....87
Figure S58.....88
Figure S59.....89
Figure S60.....90
Figure S61.....91
Figure S62.....92
Figure S63.....93
Figure S64.....94
Figure S65.....95
Figure S67.....97
Figure S68.....98
Figure S69.....99
Figure S70.....100
Figure S71.....101
Figure S72.....102
Figure S73.....103
Figure S74.....104
Figure S75.....105
Figure S76.....106
Figure S77.....107

Figure S78......108
Figure S79......109
Figure S80......110
Figure S81......111
Figure S82......112
Figure S83......113
Figure S84......114
Figure S85......115
Figure S86......116
Figure S87......117
Figure S88......118
Figure S89......119
Figure S90......120
Figure S91......121
Figure S92......122
Figure S93......123
Figure S94......124
Figure S95......125
Figure S96......126
Figure S97......127
Figure S98......128
Figure S99......129
Figure S100......130
Figure S101......131
Figure S102......132

Materials and instruments

Chemicals and reagents were purchased from Sigma-Aldrich, Acros and TCI and used without further purification. All solvents (DMSO, DMF, acetonitrile, dichloromethane, hexane, ethyl acetate, and methanol) were purchased from Sigma-Aldrich and TCI and used without further treatment or distillation. Cell culture media (DMEM, MEM, folate-free RPMI), PBS buffer, FBS and penicillin-streptomycin were supplied by Corning. Thin layer chromatography (TLC) was performed on TLC-aluminum sheets (Silica gel 60 F_{254}, Merck). Flash column chromatography was performed with silica gel (230-400 mesh, Merck). Reverse-phase column chromatography was performed with Lichroprep RP-18 (40-63µm). HPLC analysis was performed with analytical column (XBridge BEH C18 Column, 130Å, 5 mm, 4.6 mm x 250 mm). Products were purified by semi-preparative column (COMOSIL 5C18-AR-300, 4.6mmI.D. x 150mm). ^{1}H, and ^{13}C nuclear magnetic resonance (NMR) spectra were recorded either on VARIAN 700 NMR, Bruker AVANCE 500 NMR or Bruker 400 NMR with ^{1}H chemical shifts (δ) reported in ppm relative to the solvent residual signals of d-chloroform (7.24 ppm), d-methanol (3.30 ppm), d-DMSO (2.49 ppm). ^{13}C chemical shifts (δ) were reported in ppm relative to the solvent residual signals of d-chloroform (77.0 ppm), d-methanol (49.0 ppm) and d-DMSO (39.5 ppm). Coupling constants were reported in Hz. High resolution mass spectra (HRMS) were recorded on Varian 901-MS TQ-FTMS. Streptavidin-Cy5, -Cy3 and -Alexa488 were from Jackson ImmunoResearch Inc. The lateral flow assay membrane (Whatman FF120HP membranes) was purchased from Cytiva. The backing card was purchased from Prisma Biotech. The absorbent pad was purchased from Merck. The sample pad was purchased from Advanced Microdevices. All the LFA images presented in the manuscript were captured using a handphone camera. The test line intensities were recorded using ChemiDoc Touch Imaging system (Bio-rad Inc, CA, USA) and analyzed using Image lab software (Bio-rad Inc, CA, USA).

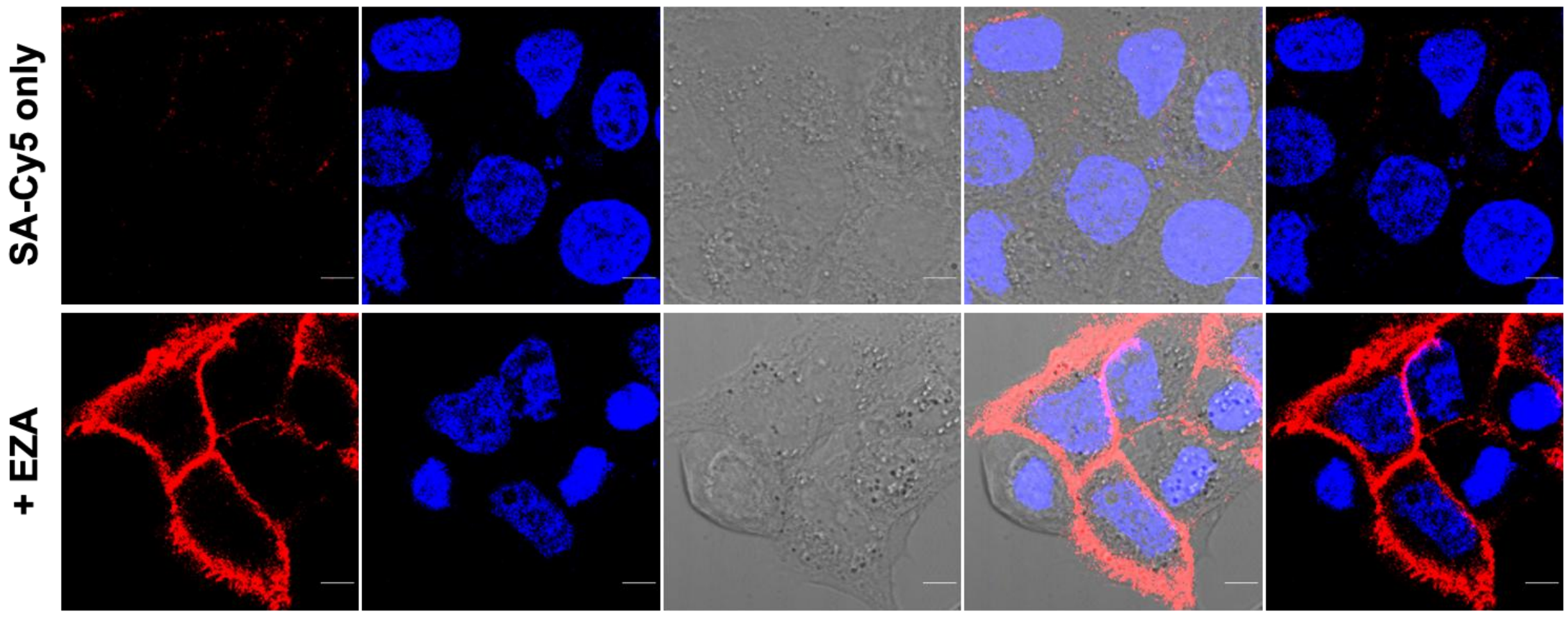

Figure S1. Fluorescent images of SNAP-hCAII-1 after post-fixation. Scale bar: 20 μm.

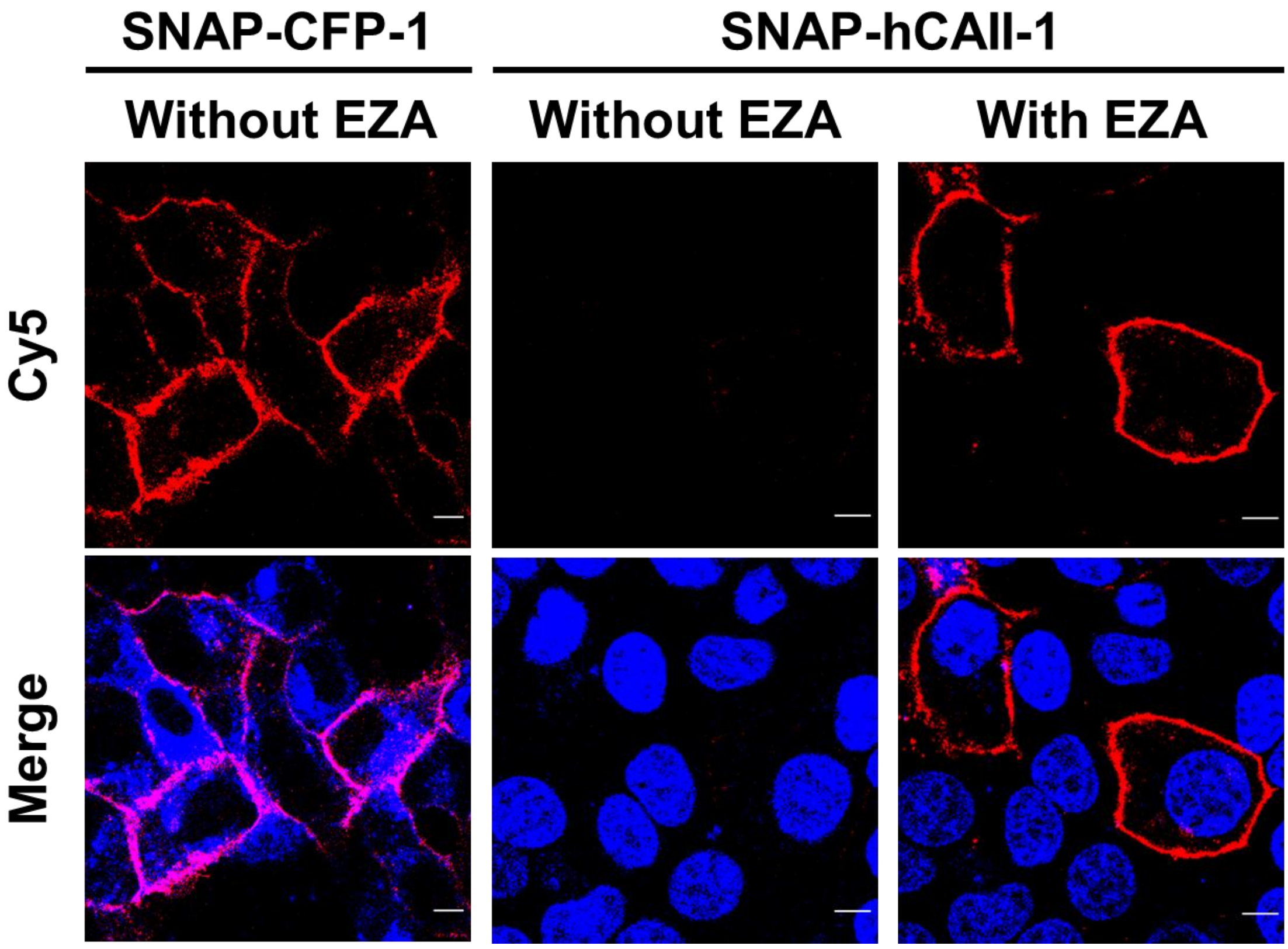

Figure S2. Fluorescence imaging of the always-open SNAP-CFP control construct in MCF7 cells labeled with probe **1**. Strong cell-surface signal is observed in the absence of the target drug, confirming that the N3B moiety remains constitutively accessible to streptavidin when a matching intramolecular binding pocket is absent. Scale bar: 20 μm.

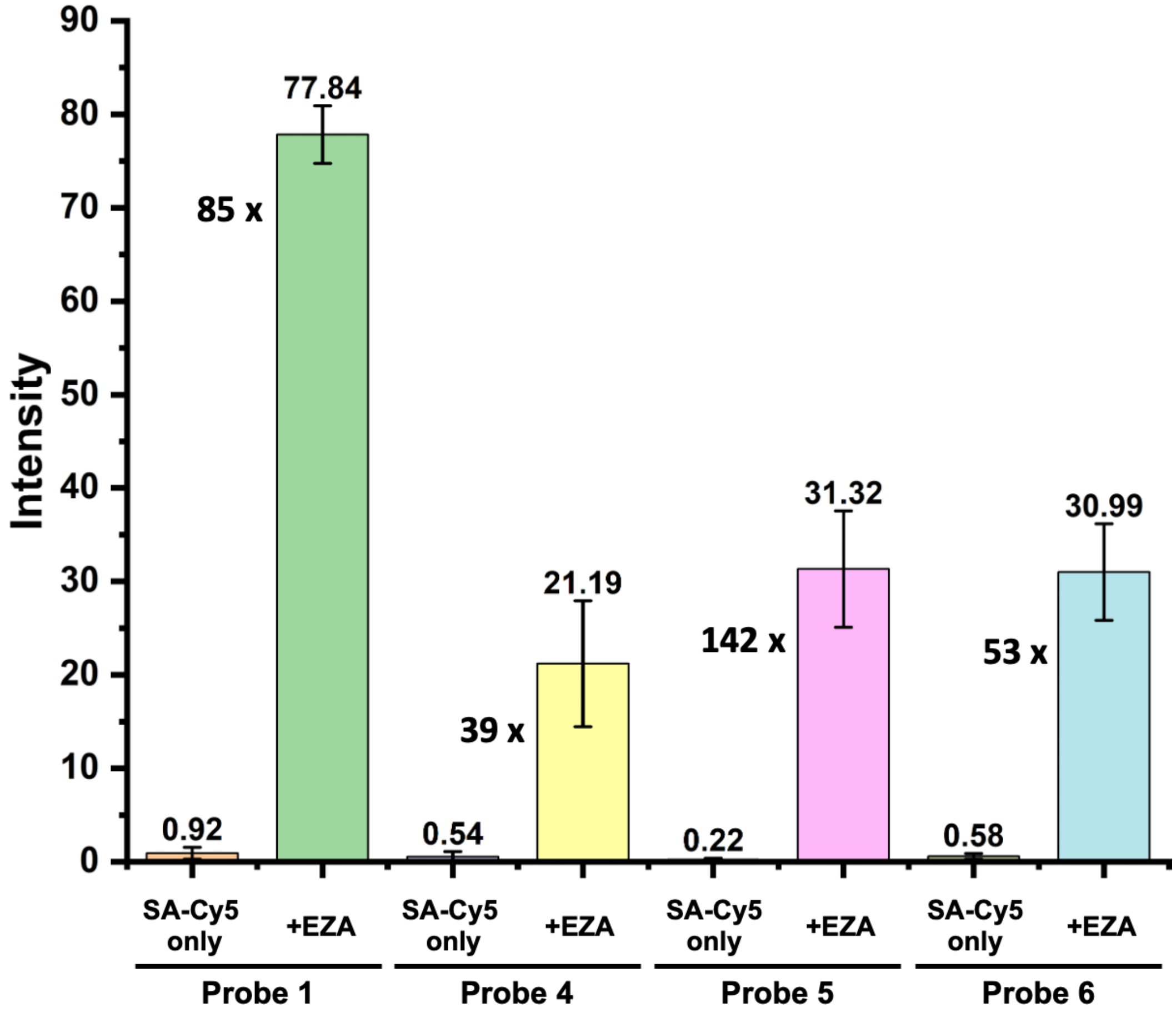

Figure S3. The relative fluorescent intensities of **SNAP-hCAII-1**, **-4**, **-5**, and **-6** on plasma membrane of MCF7 cells in the absence or presence of 100 μM EZA. The fluorescent enhancement ratios were shown beside the bars. The average fluorescent intensities and the error bars were calculated from 30 cells and quantified by using Image J software.

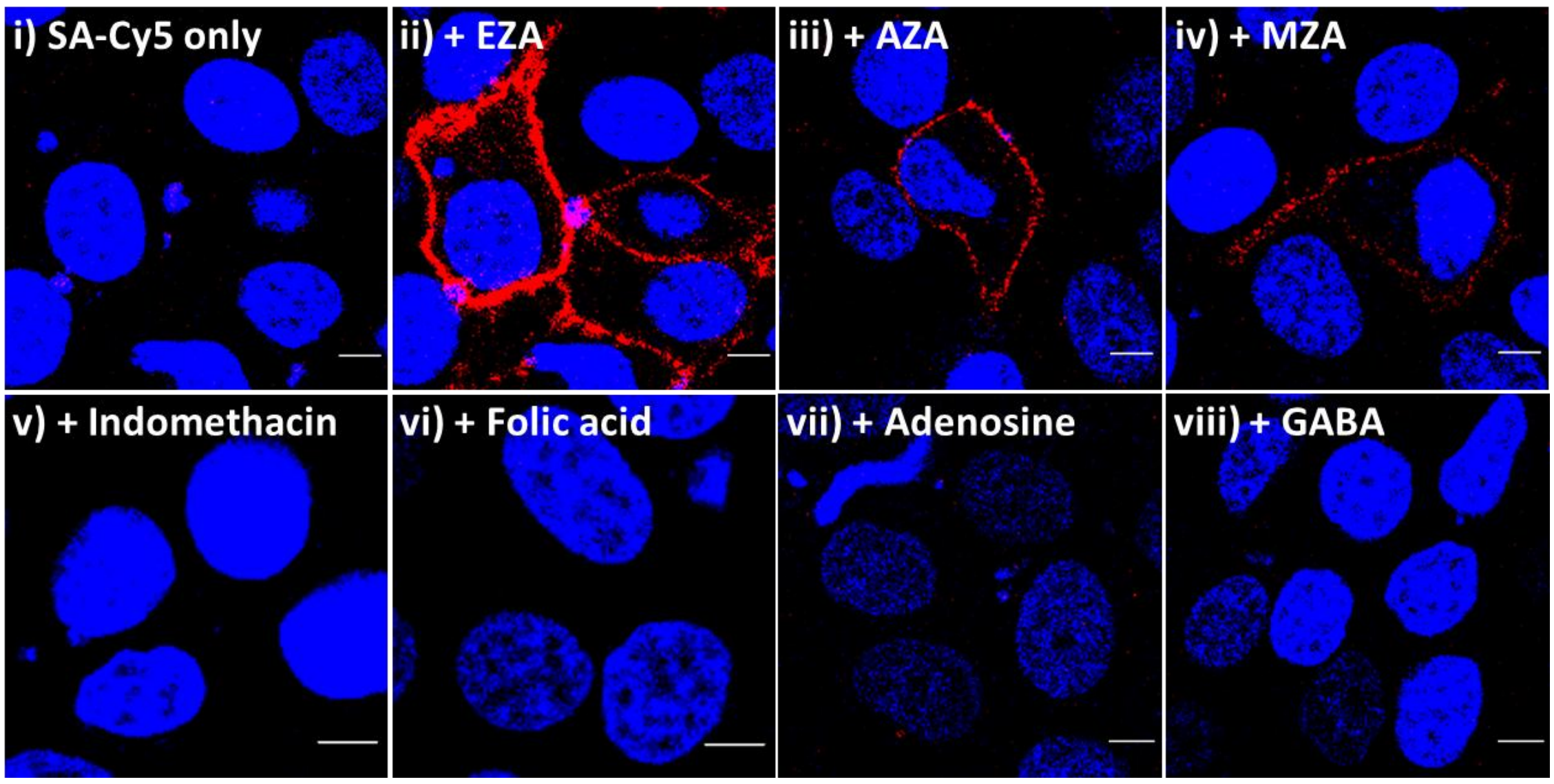

Figure S4. Fluorescent images of **SNAP-hCAII-1** co-incubated with SA-Cy5 (1 μg/mL) and various small molecules. Scale bar: 20 μm.

(a)

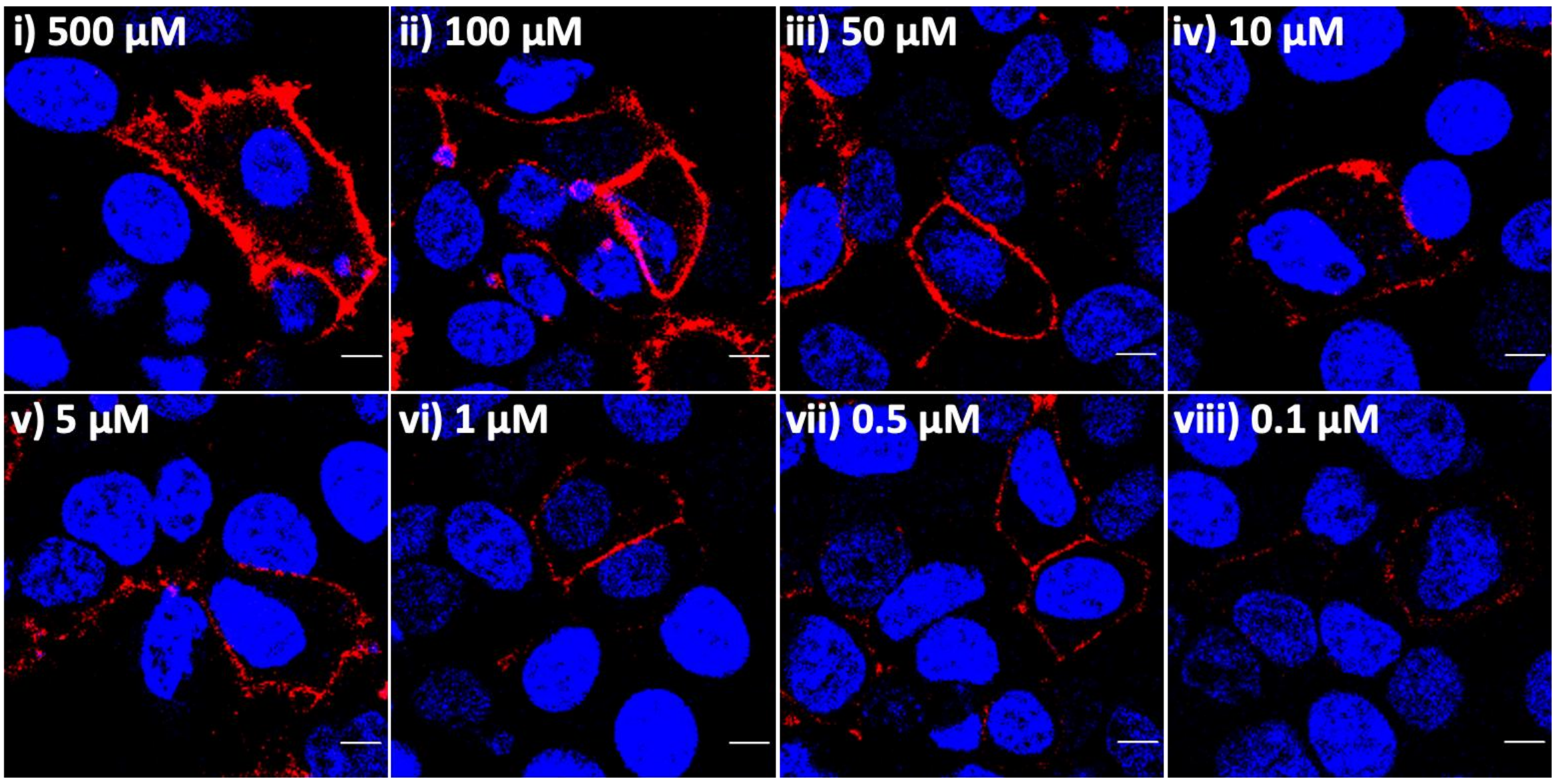

(b)

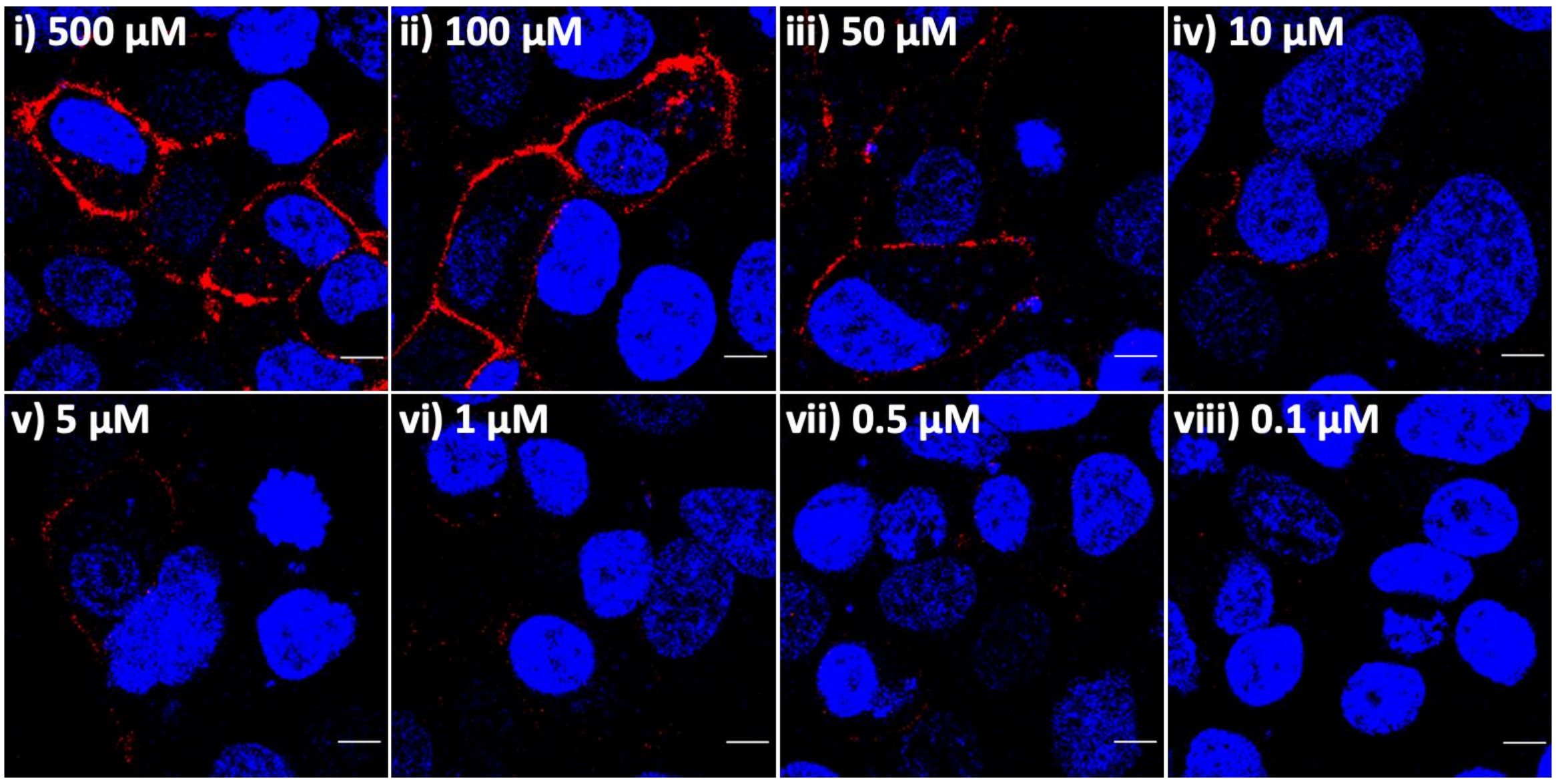

Figure S5. Fluorescent images of **SNAP-hCAII-1** co-incubated with SA-Cy5 (1 µg/mL) and different concentrations of (a) EZA or (b) AZA. Scale bar: 20 µm.

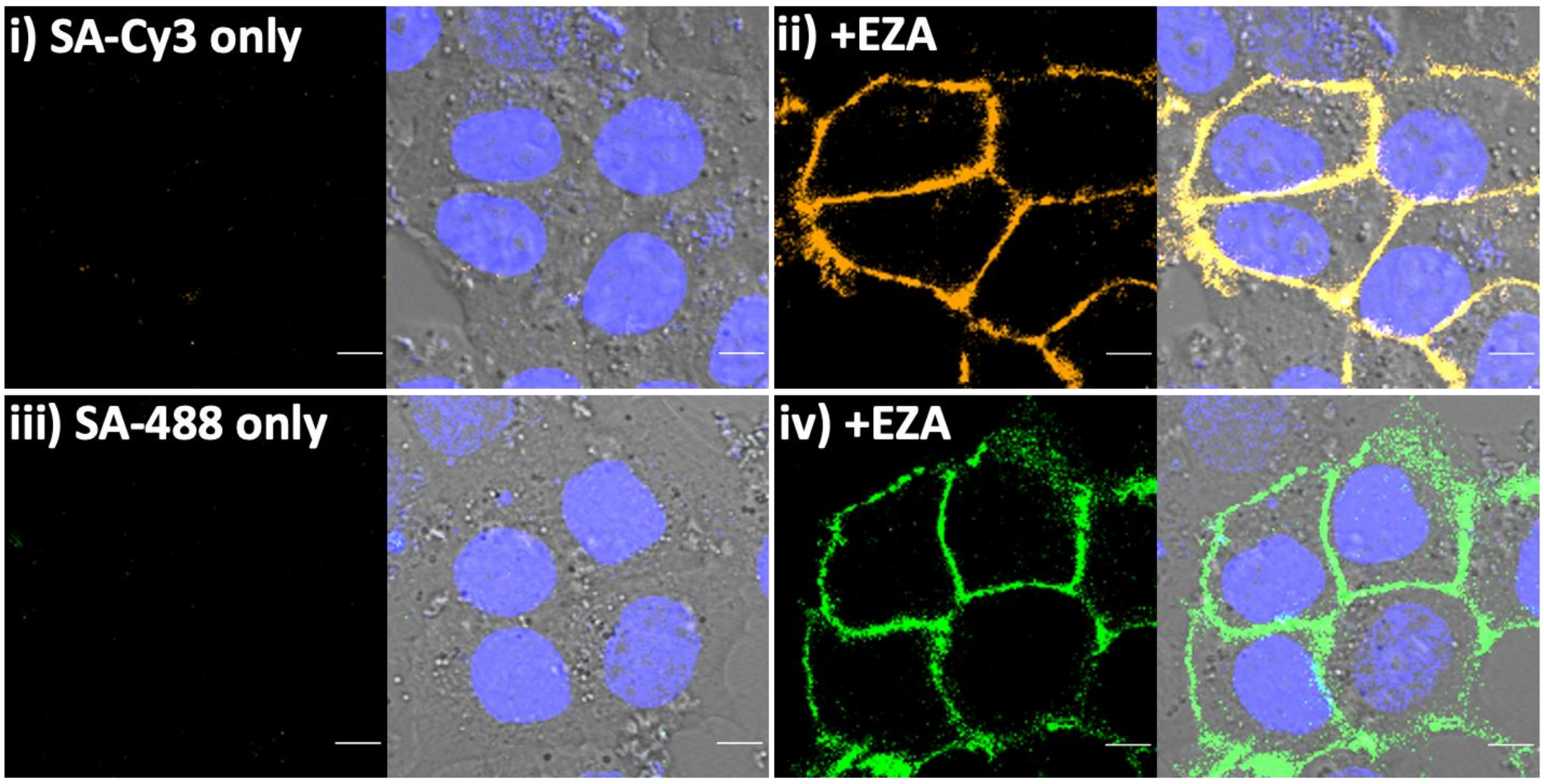

Figure S6. EZA detection with Cy3 or Alexa488 dyes. **SNAP-hCAII-1** treated with 1 µg/mL (i) SA-Cy3 and (iii) SA-488 only or co-incubation with (ii) and (iv) 100 µM EZA for 10 minutes in MEM medium. Scale bar: 20 µm.

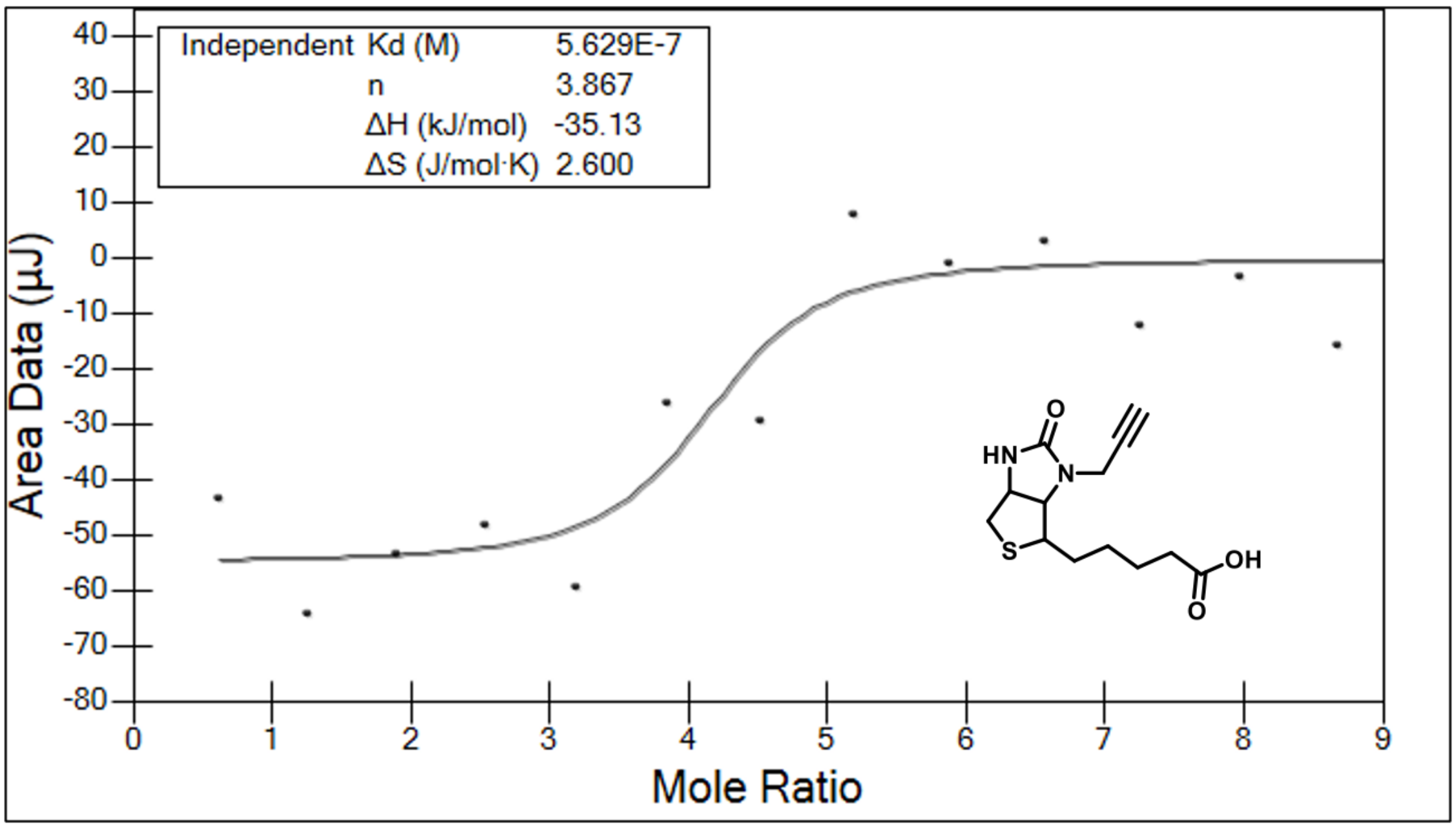

Figure S7. Isothermal titration calorimetry (ITC) result of the binding affinity between the N'-3 urea nitrogen biotin derivative (N3B) and streptavidin.

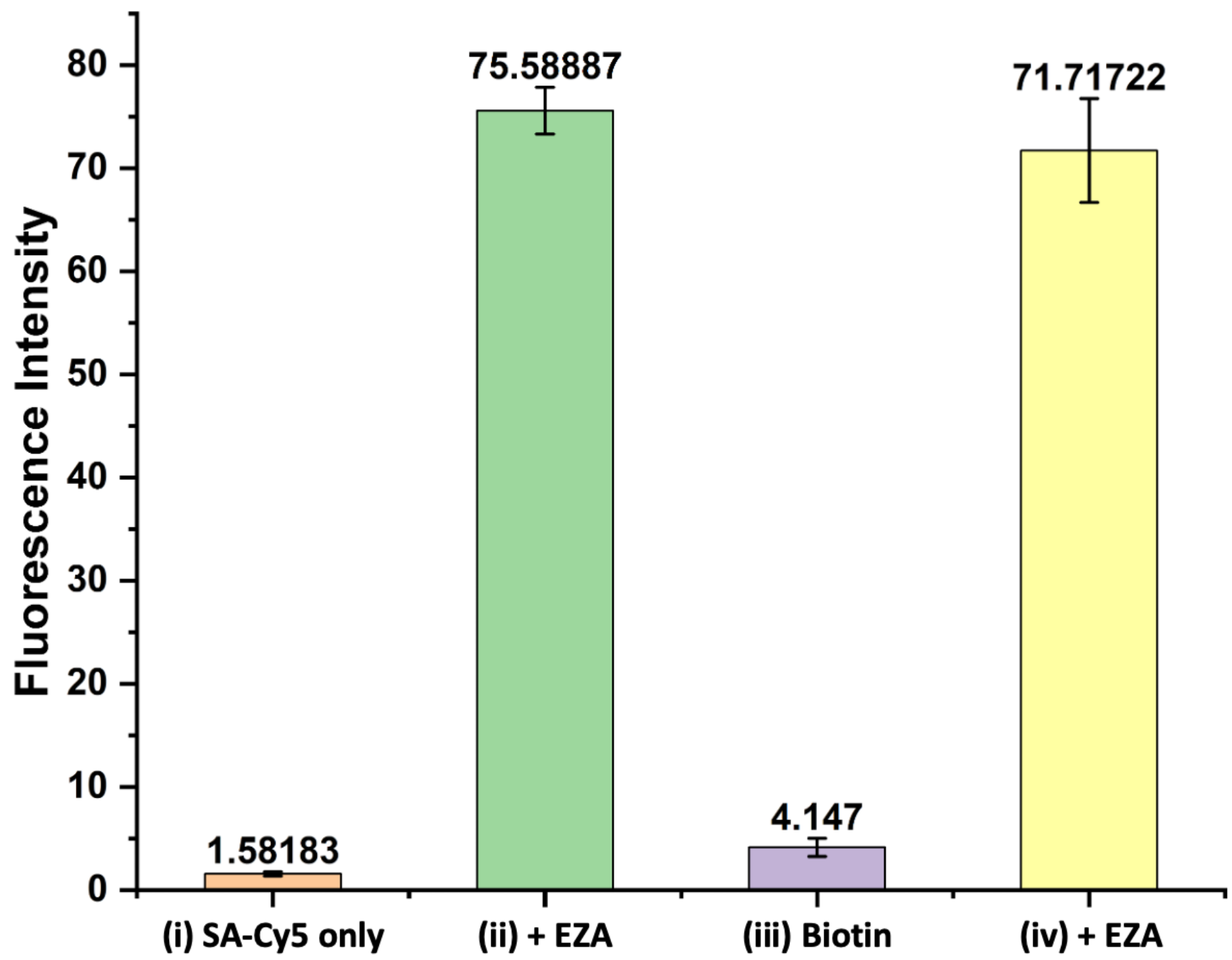

Figure S8. The relative fluorescent intensity of **SNAP-hCAII-1** (i) in the absence of EZA, (ii) in the presence of 100 μM EZA, (iii) recover the protein switch by using 50 μM biotin, (iv) incubate with 100 μM EZA again. The average fluorescent intensities and the error bars were calculated from 30 cells and quantified by using Image J software.

(a)

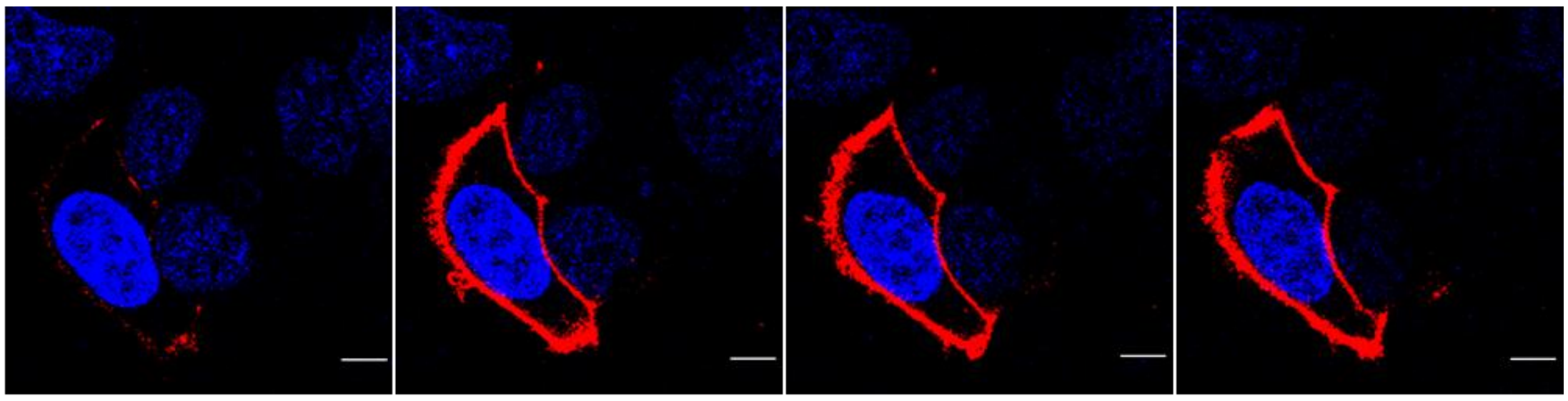

(b)

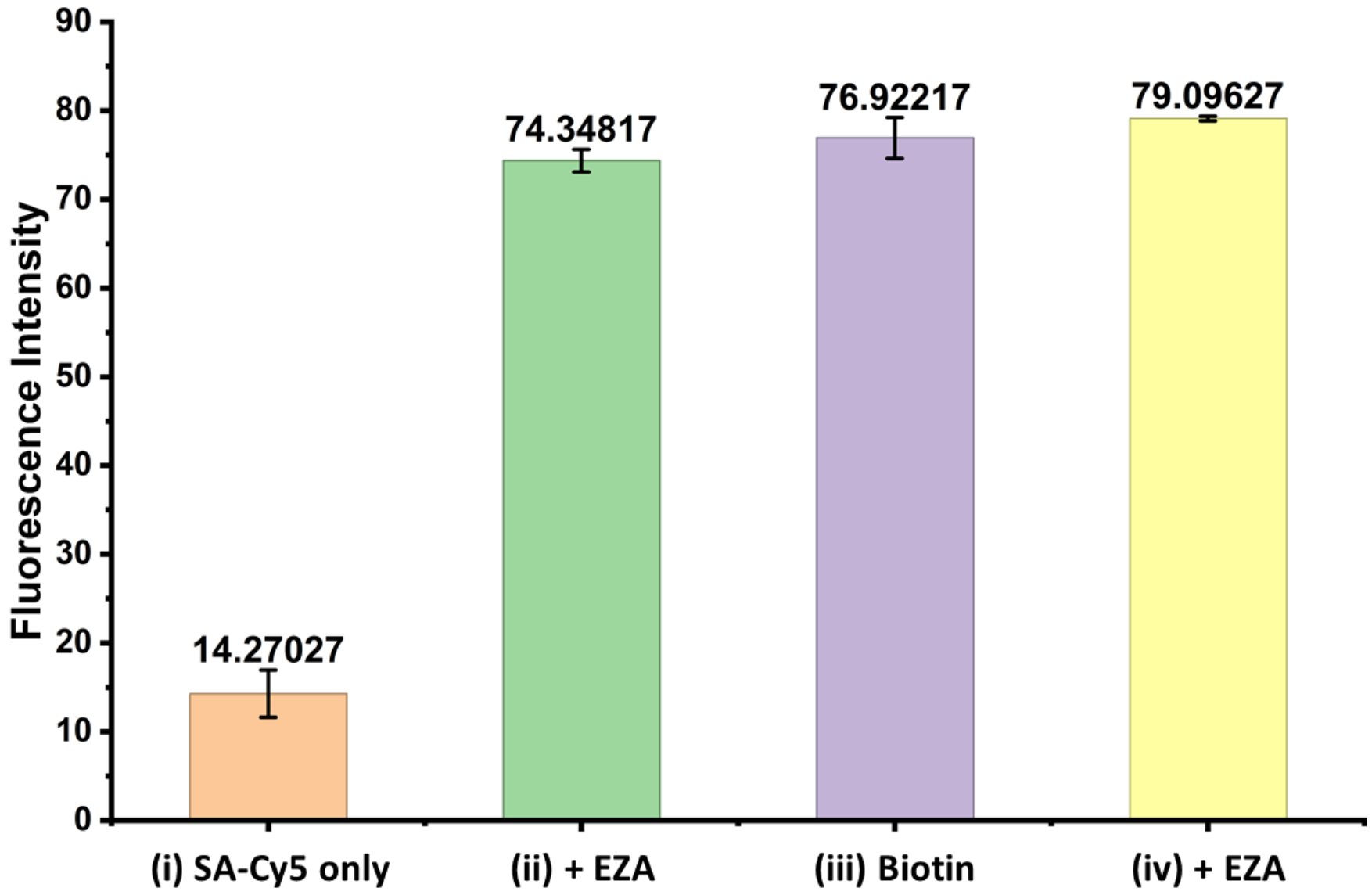

Figure S9. (a) Fluorescence imaging of MCF7 cells expressing **SNAP-hCAII-2** treated sequentially with 100 μM EZA and 50 μM biotin. Due to the nearly irreversible binding between the native biotin of probe **2** and SA-Cy5, the fluorescence cannot be switched OFF upon the addition of biotin. (i) SA-Cy5 only, (ii) addition of 100 μM EZA and SA-Cy5, (iii) incubation with 50 μM biotin, (iv) washing followed by the re-introduction of 100 μM EZA and SA-Cy5. (b) The relative fluorescent intensities of MCF7 cells expressing **SNAP-hCAII-2** upon sequential addition of EZA and biotin. Scale bar: 20 μm.

Supplementary Video

Supplementary Video S1. This video presents orthogonal slicing of a 3D image, displaying the fly brain from the surface down to the base. The centrally labeled region with SA-Alexa 633 corresponds to the mushroom body area in Drosophila.

(a)

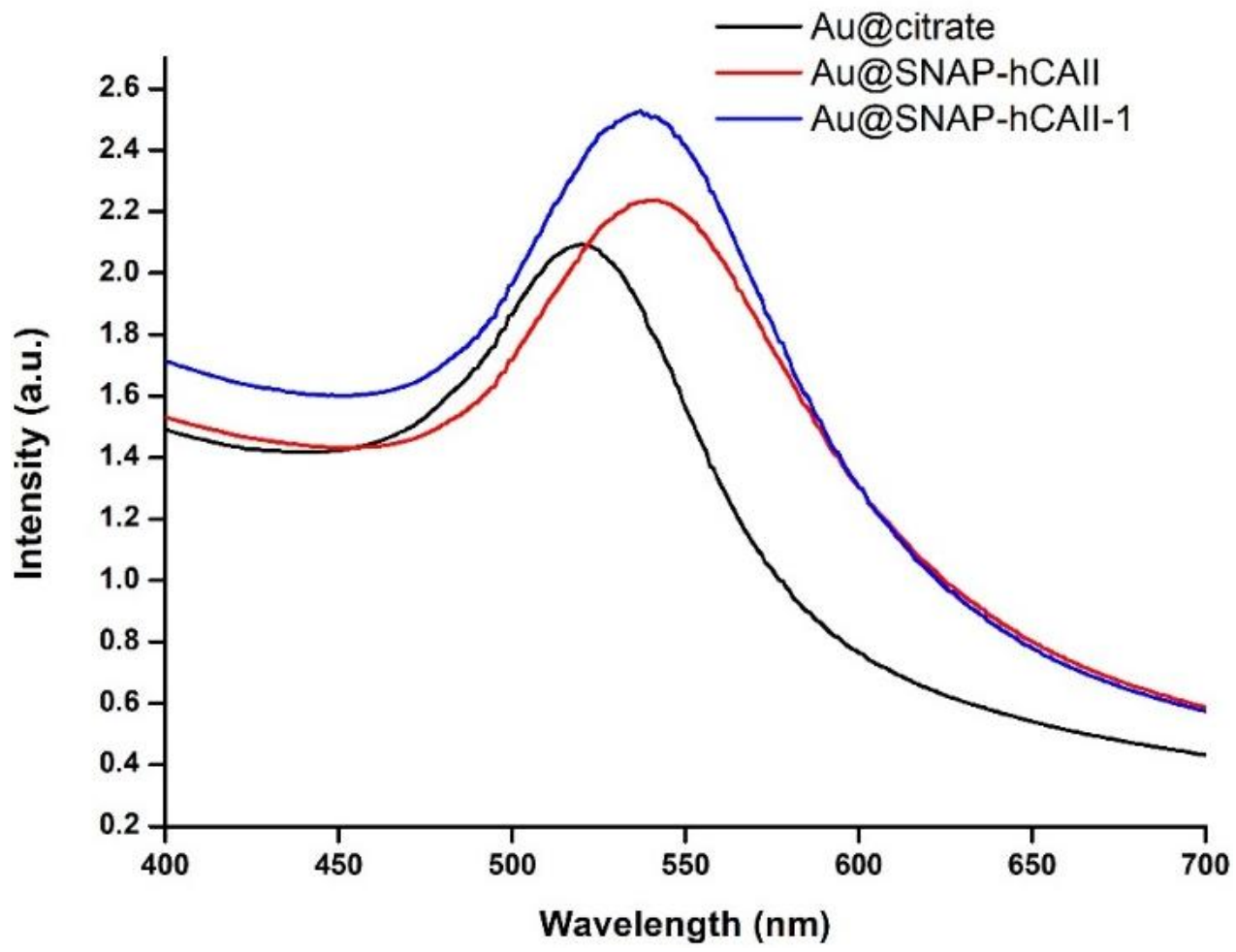

(b)

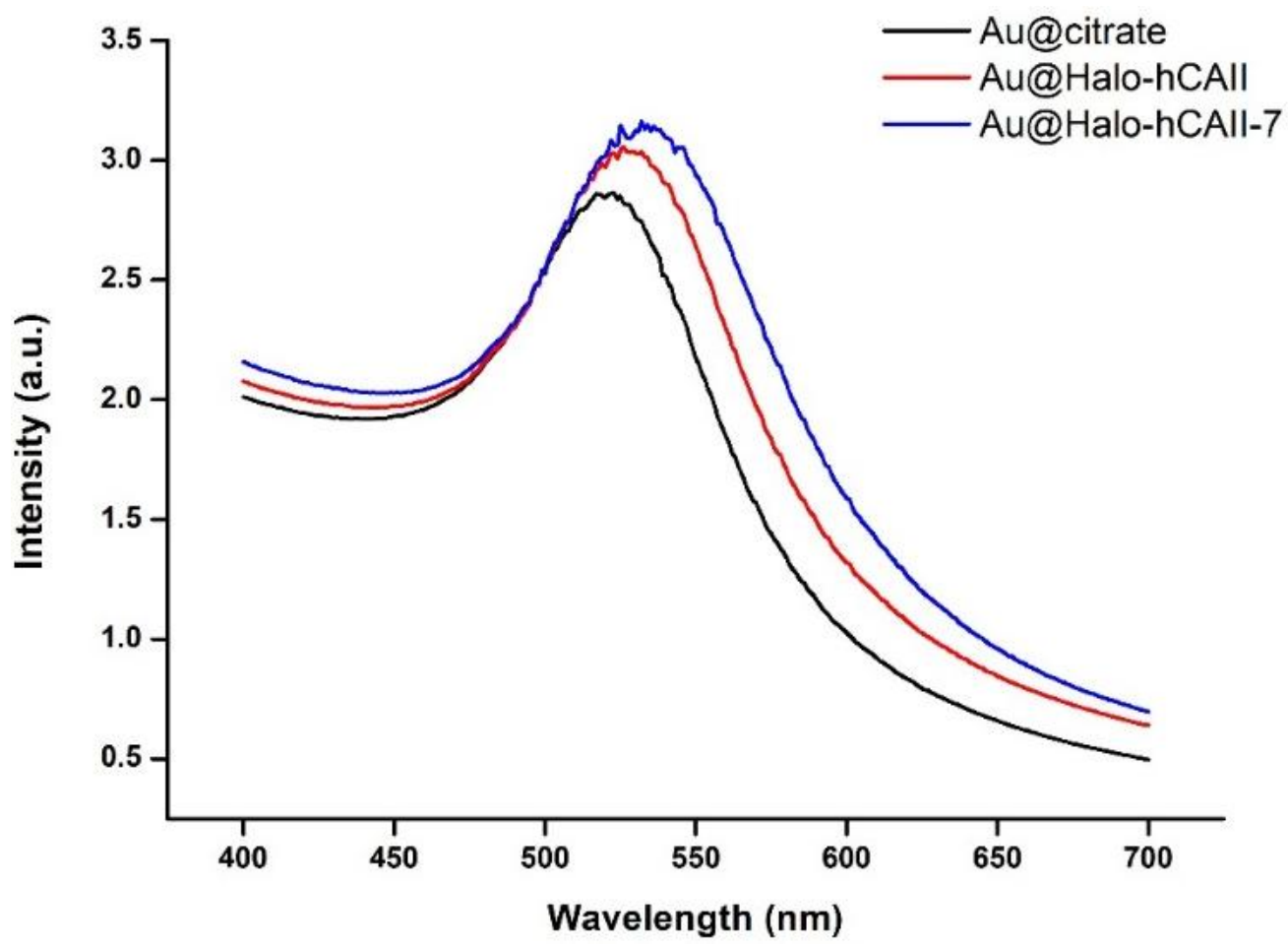

Figure S10. Absorption spectra of (a) **Au@SNAP-hCAII-1** and (b) **Au@Halo-hCAII-7**.

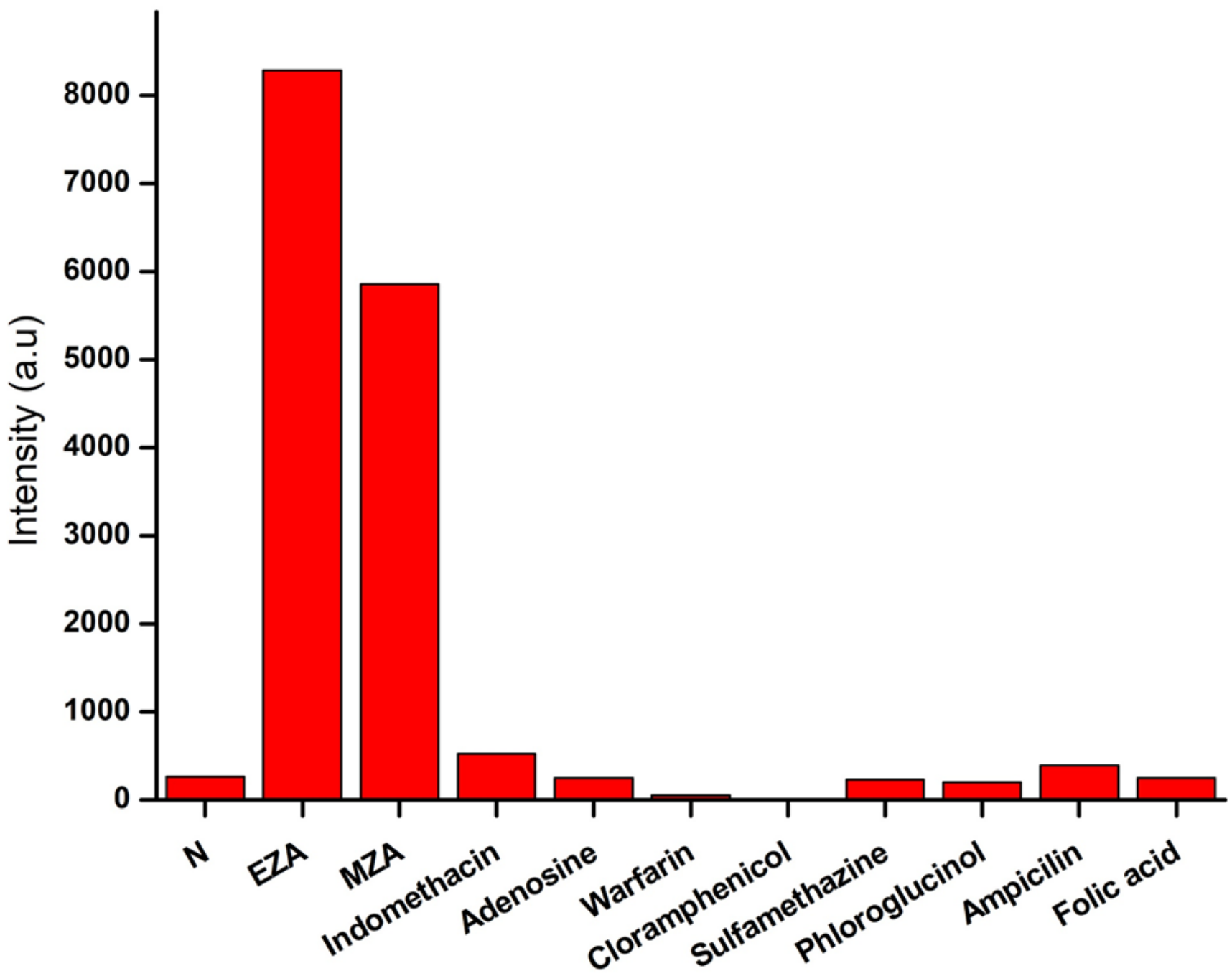

Figure S11. Selective testing of sulfonamide drugs (10 μM) and other non-target analytes (100 μM each) using **Au@Halo-hCAII-7**. Quantitative analyses of the test line intensities. The signals were quantified using Image Lab software (Biorad, Inc).

(a)

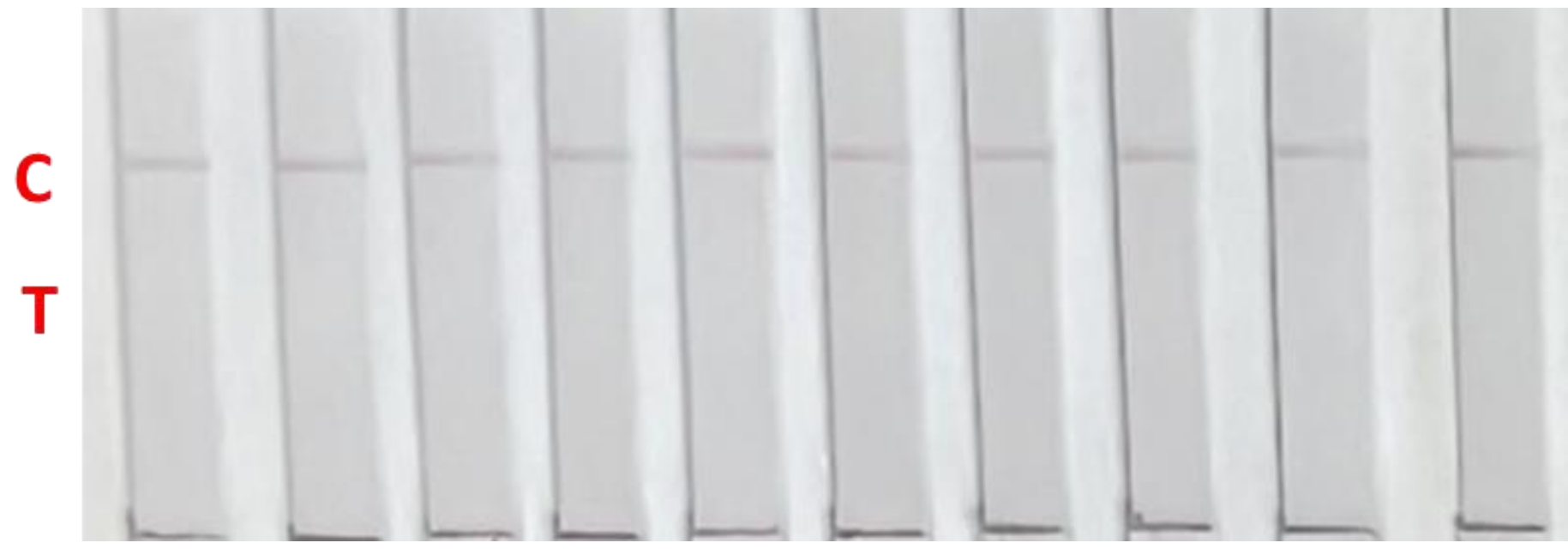

(b)

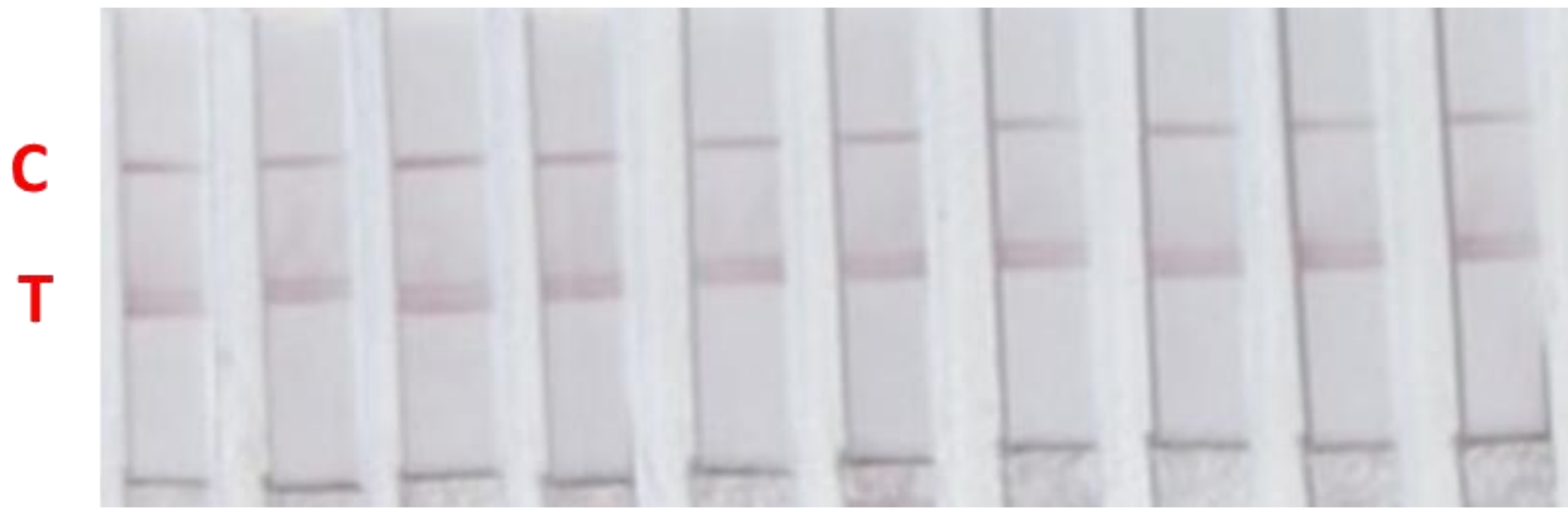

(c)

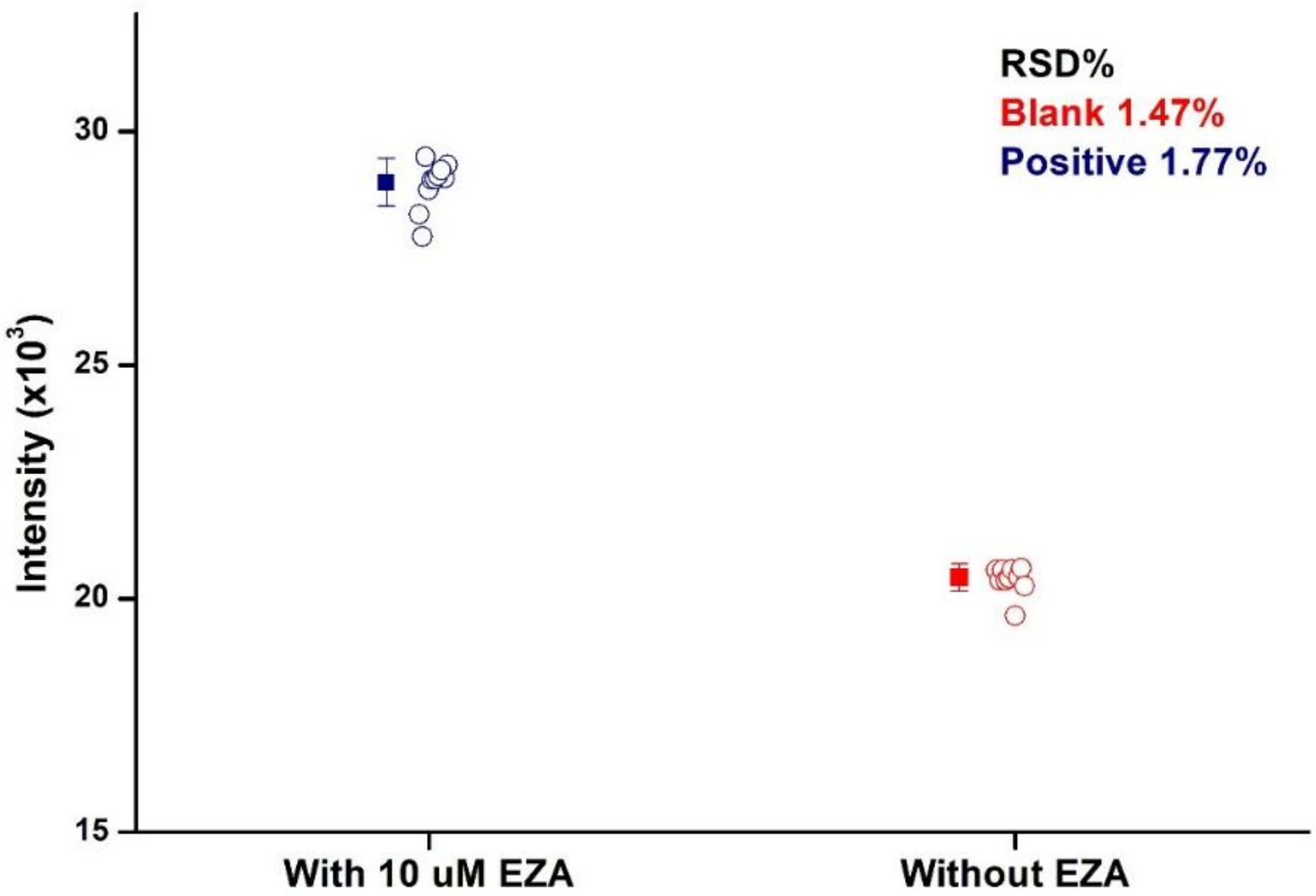

Figure S12. Precision study of EZA detection with **Au@Halo-hCAII-7**. Images of the LFA test strips after testing (a) without EZA or (b) with 10 μM EZA. (c) Quantitative analysis of test line intensity. The signals were quantified using Image Lab software (Biorad, Inc).

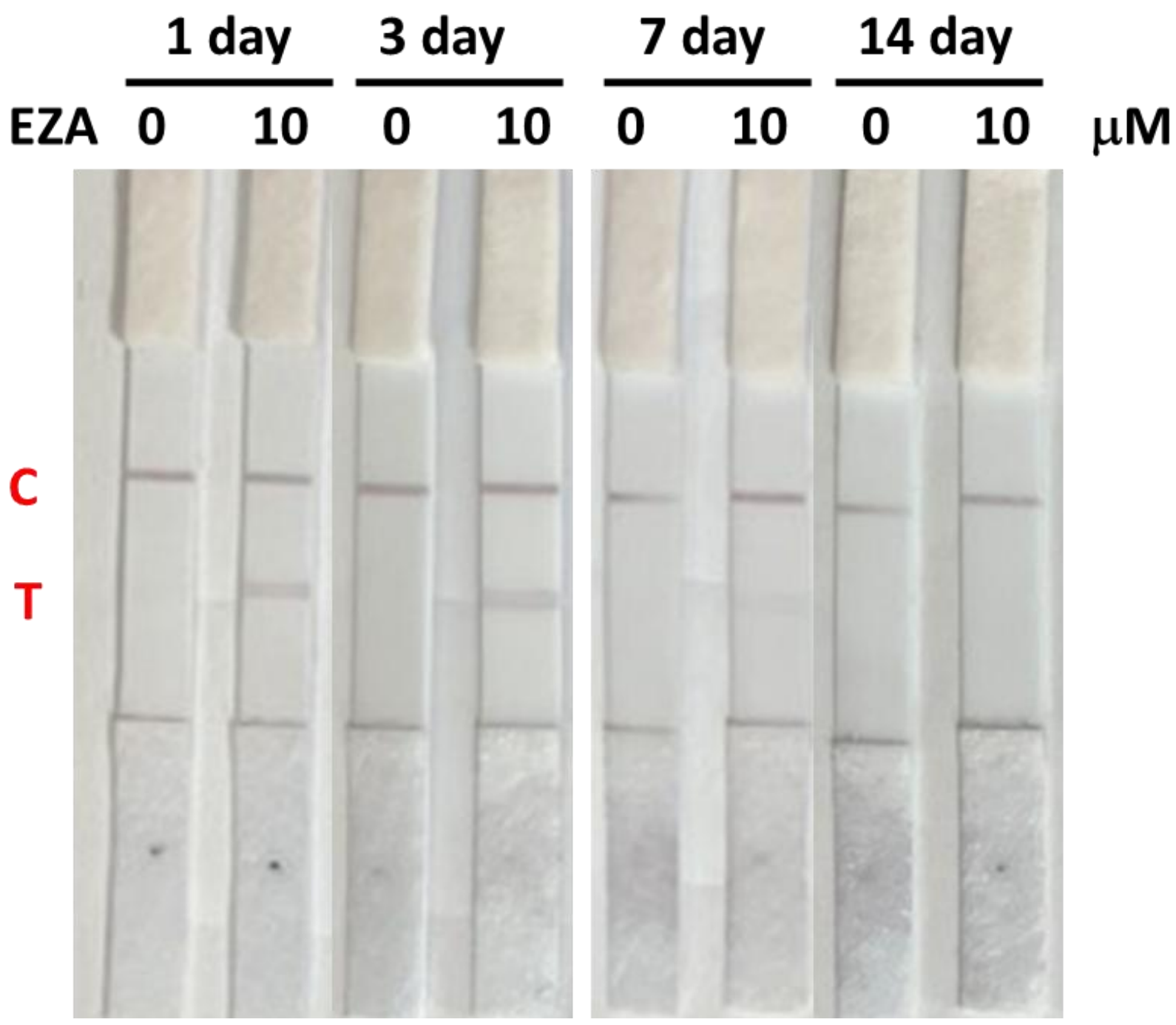

Figure S13. Thermal stability evaluation of unsealed lateral flow assay (LFA) test strips under accelerated aging conditions at 55 °C. The colorimetric sensing performance is maintained for 3 days before displaying signs of gradual degradation.

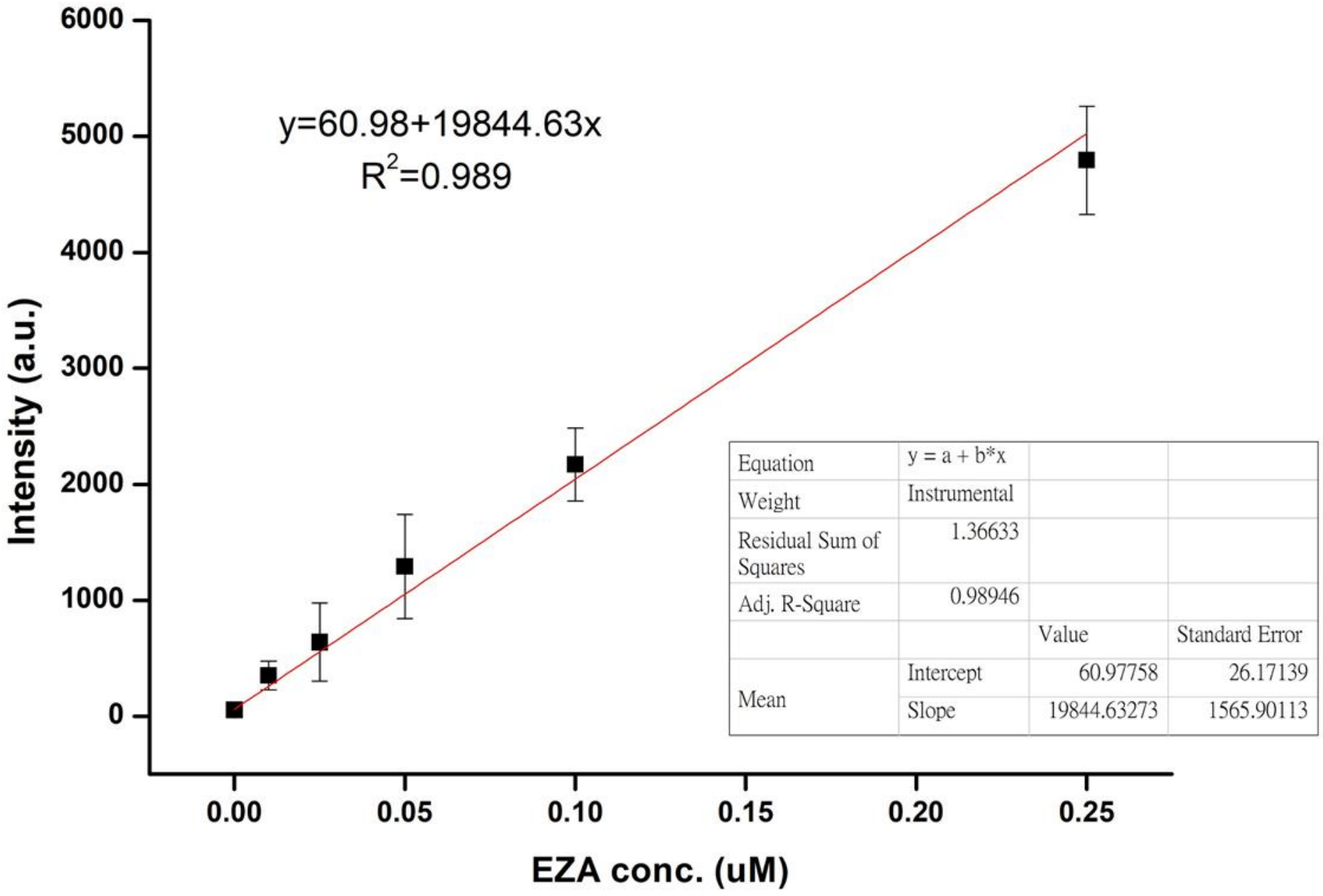

Figure S14. Quantitative analysis of the test strips after testing various concentrations of EZA with **Au@Halo-hCAII-7.**

(a)

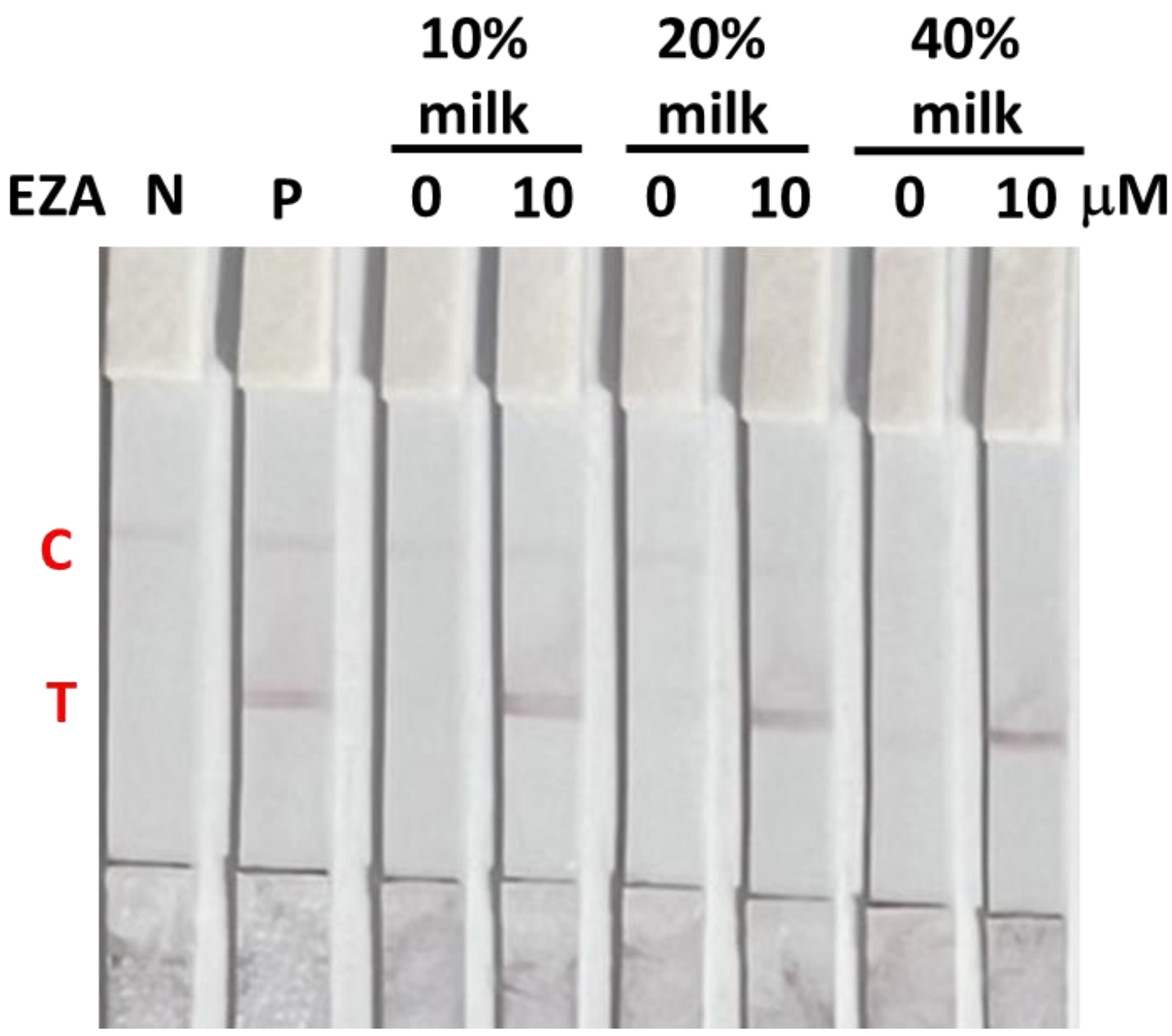

(b)

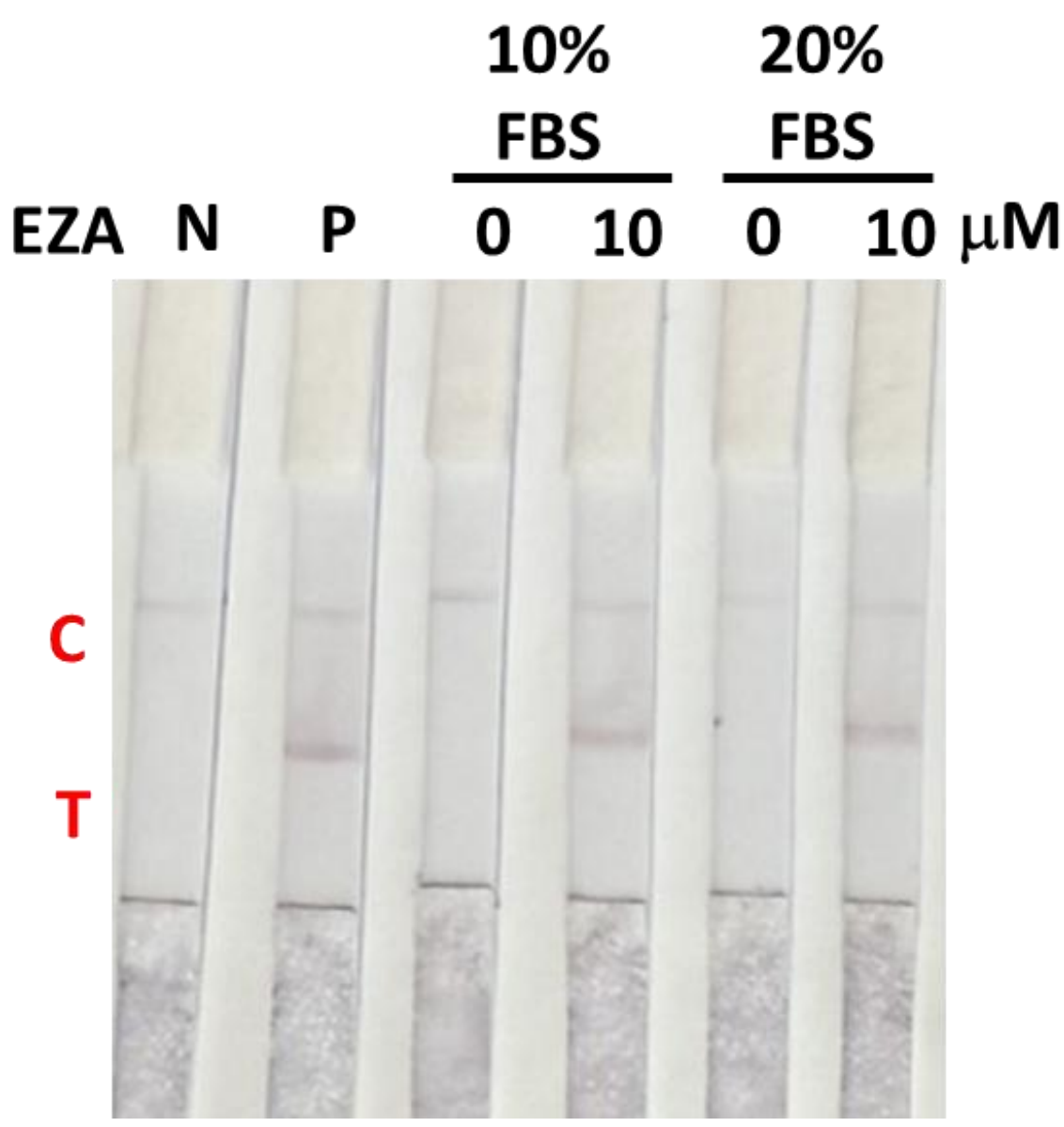

Figure S15. Testing of 10 μM EZA in (a) milk and (b) FBS solutions with **Au@Halo-hCAII-7**.

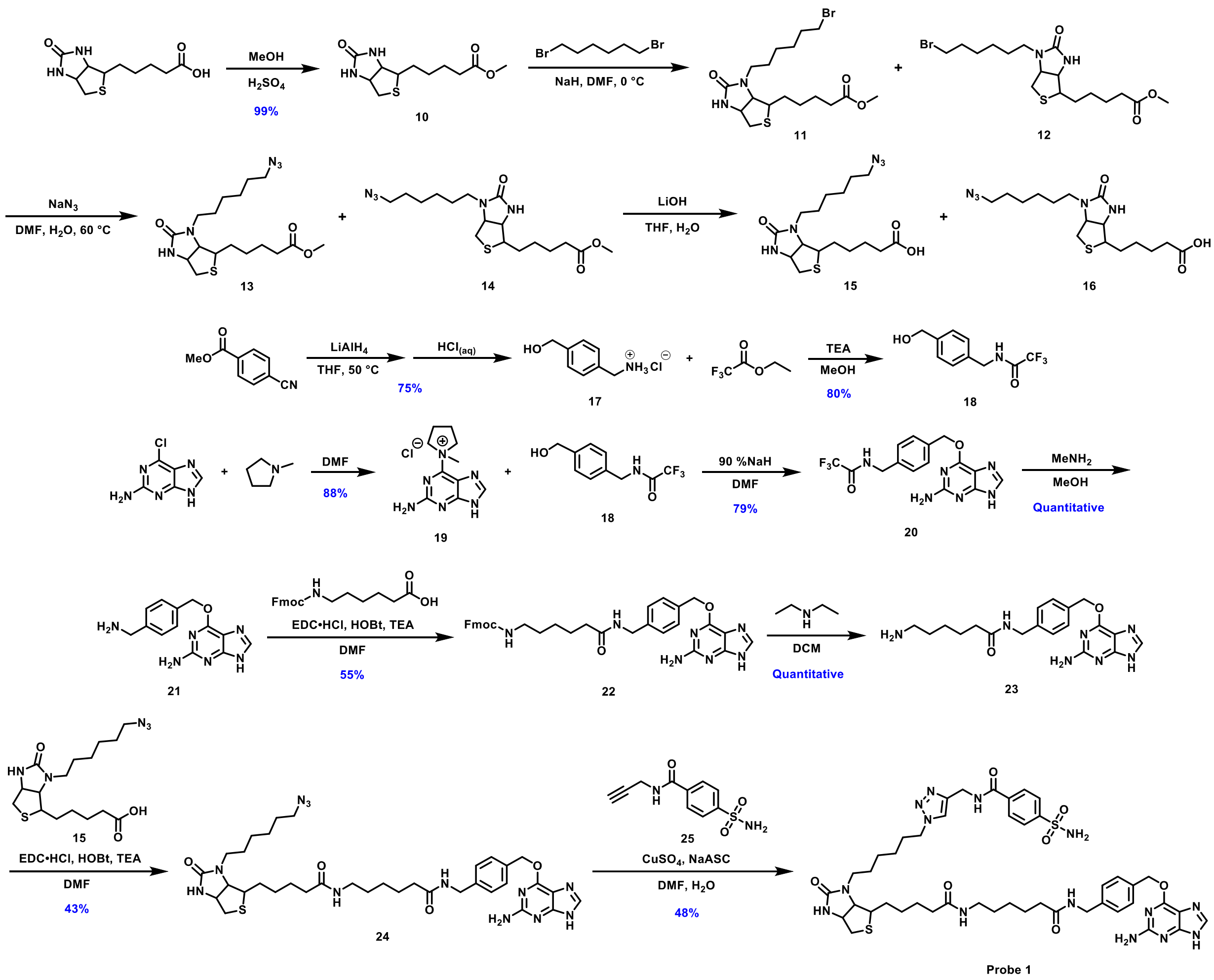

Scheme S1. Synthesis of probe **1**.

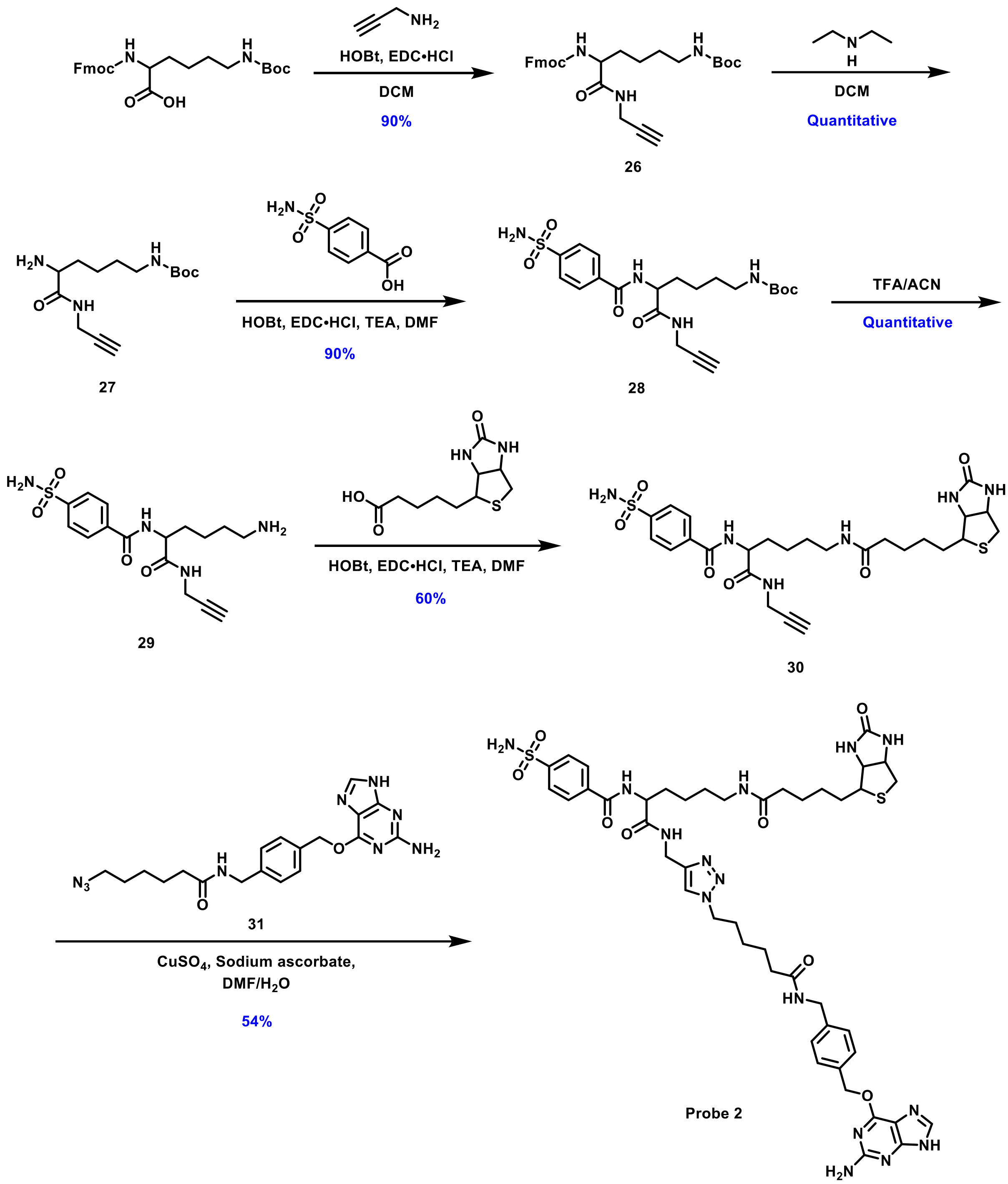

Scheme S2. Synthesis of probe **2**.

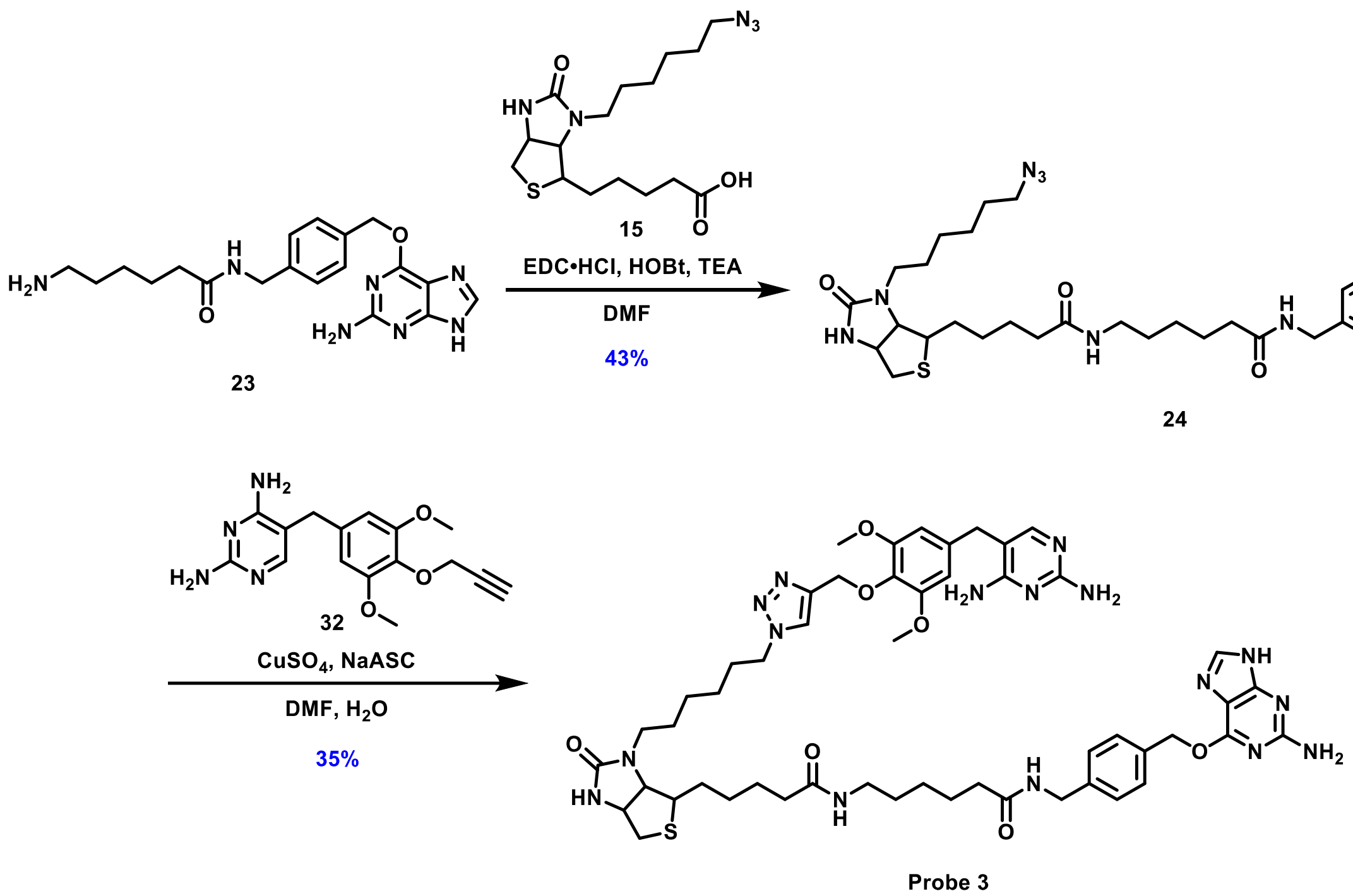

Scheme S3. Synthesis of probe **3**.

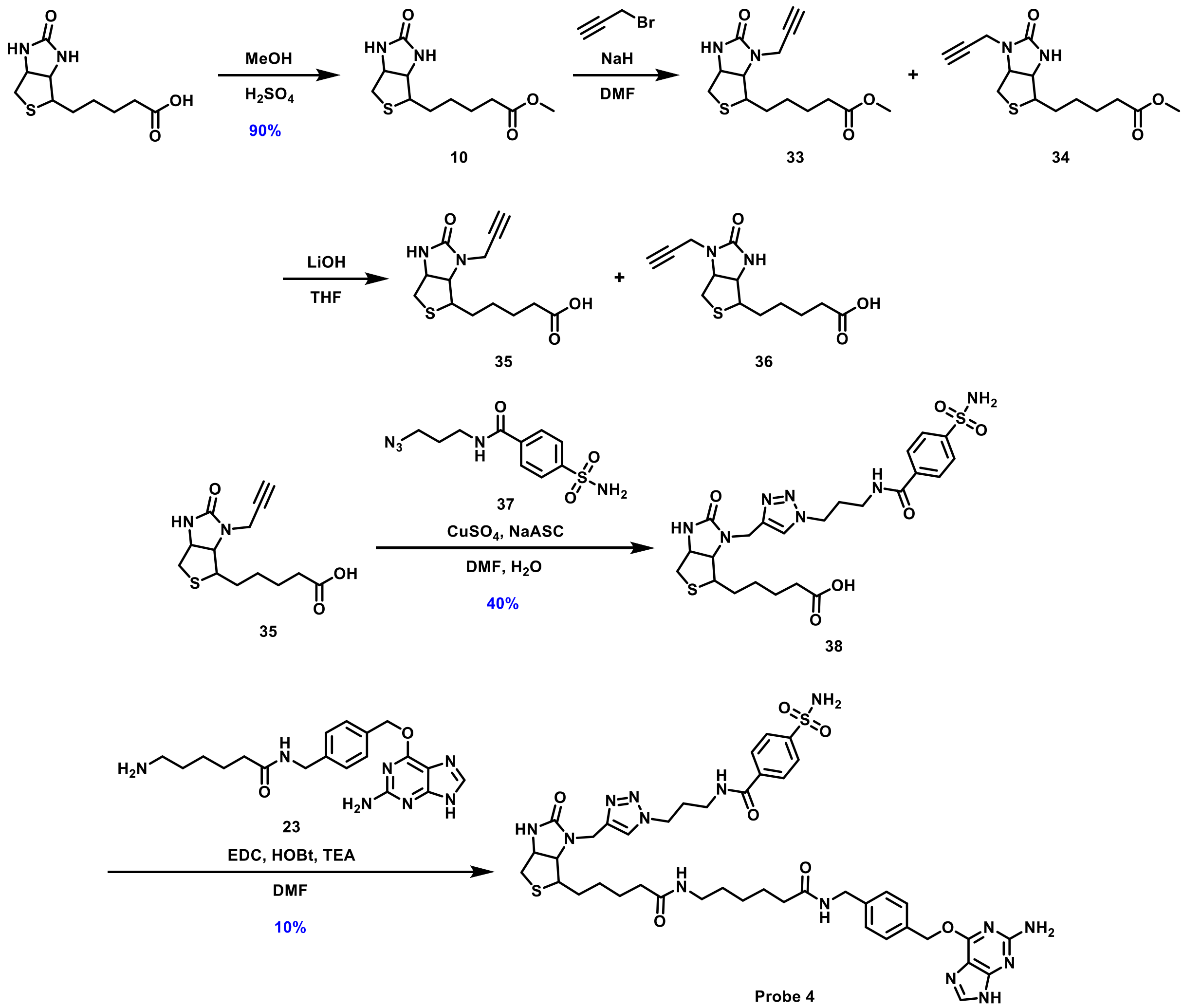

Scheme S4. Synthesis of probe 4.

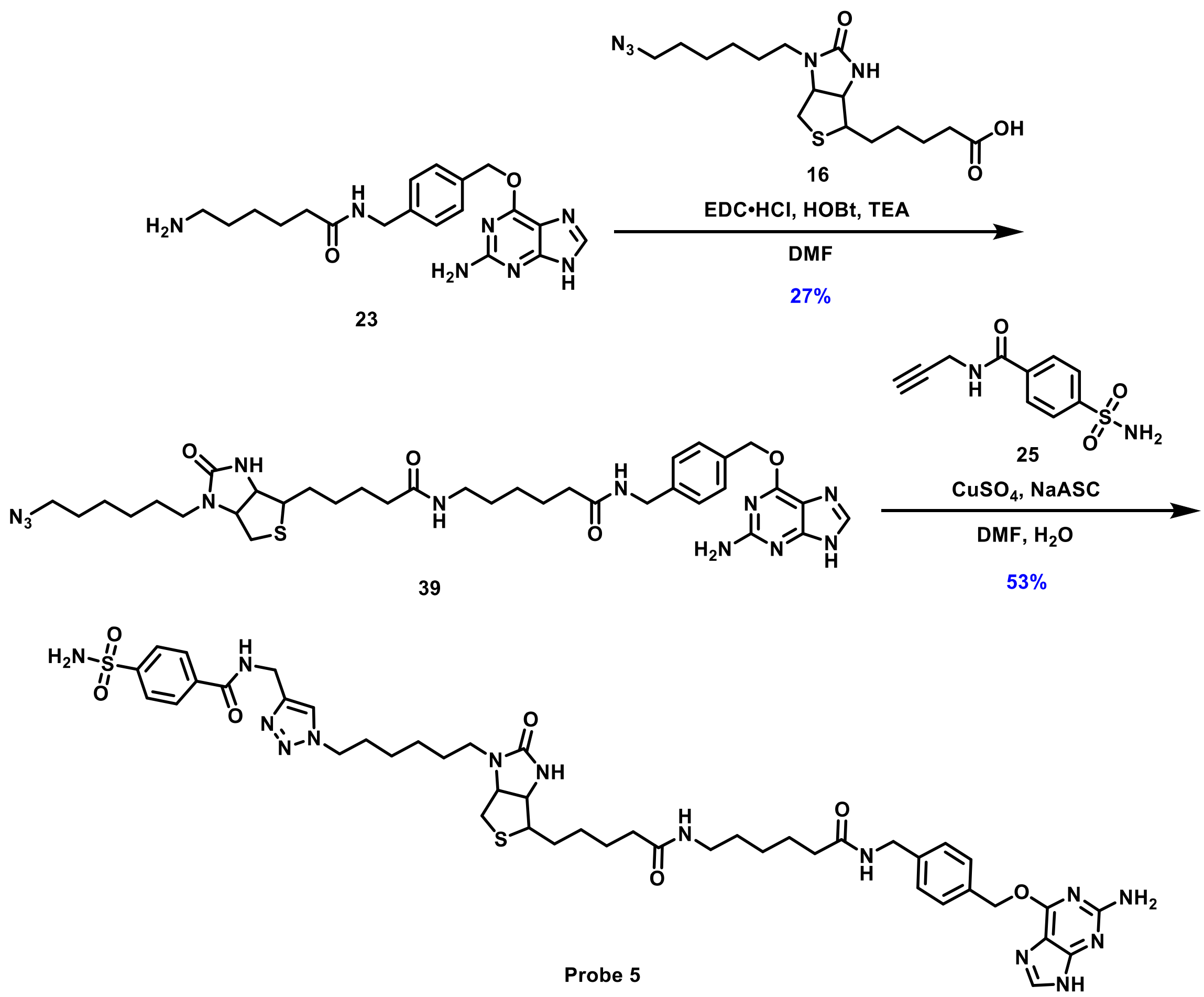

Scheme S5. Synthesis of probe **5**.

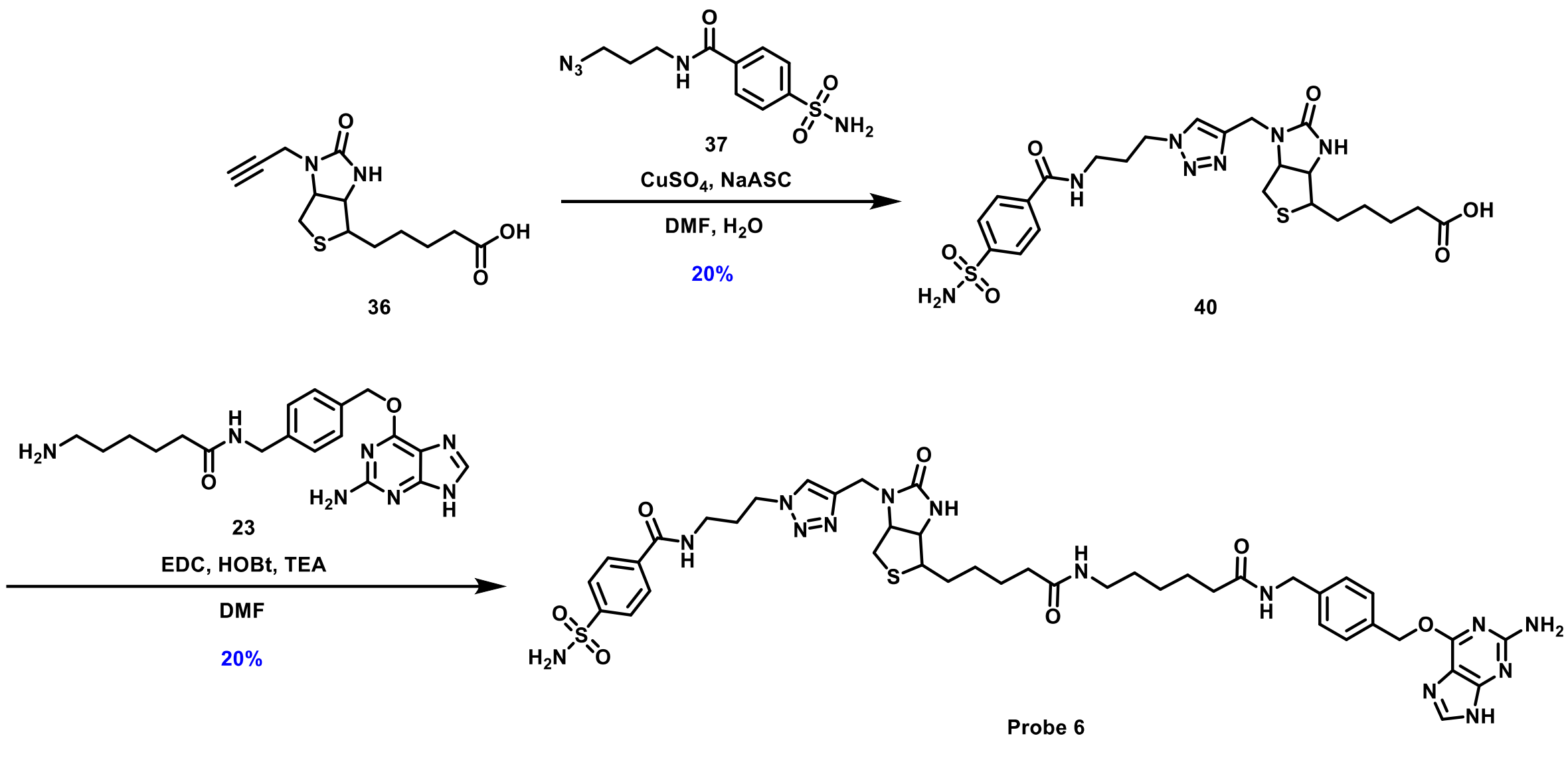

Scheme S6. Synthesis of probe **6**.

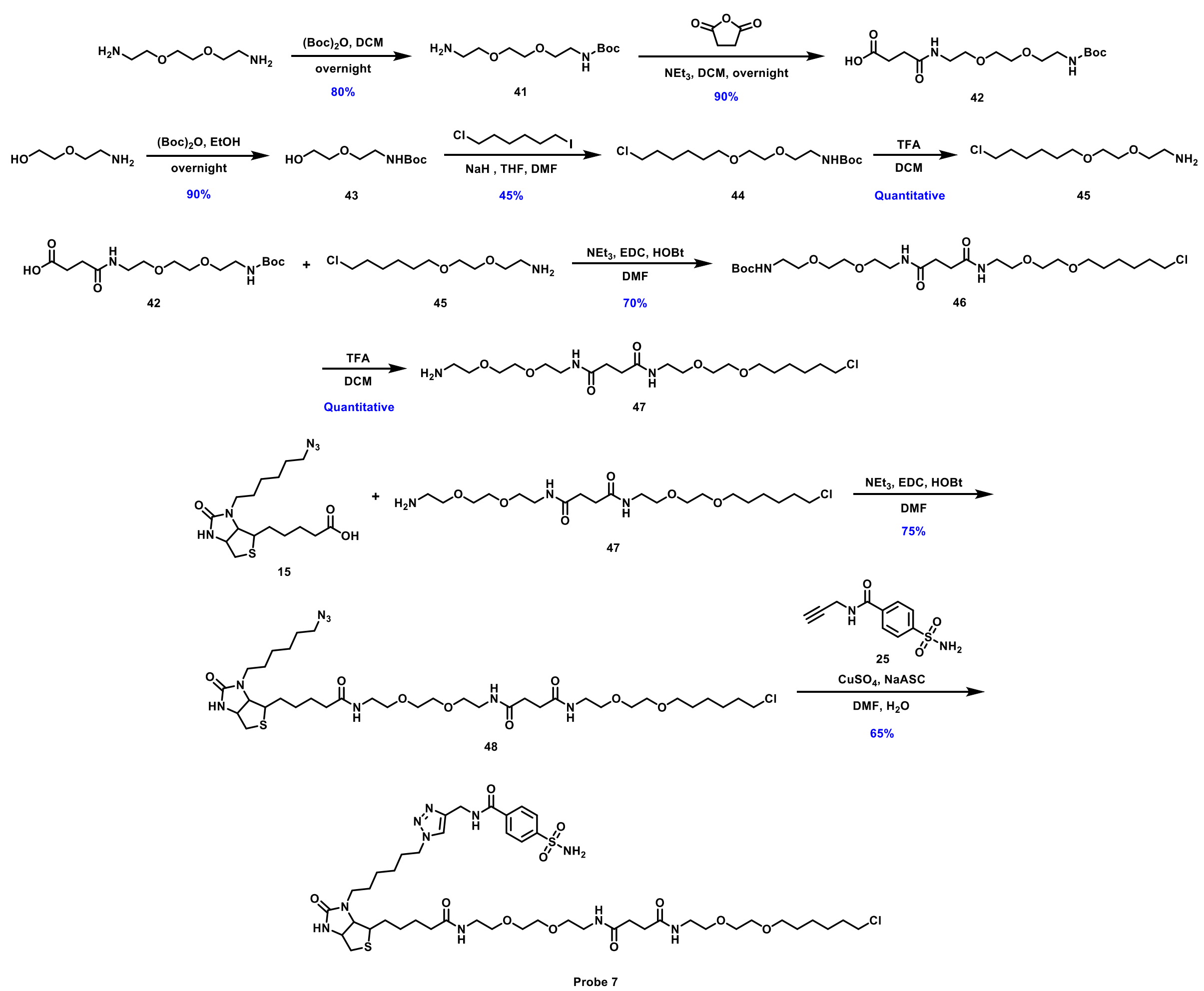

Scheme S7. Synthesis of probe **7**.

Synthesis of Compound 10

To a solution of biotin (10 g, 41 mmol) in MeOH (250 mL) was added conc. H_2SO_4 (14 mL), with ice-bath cooling. The reaction mixture was stirred at room temperature for 18 hours. The reaction mixture was concentrated under reduced pressure then extracted with 5% MeOH/DCM and water. The combined organic layers were washed with brine, dried over Na_2SO_4, filtered and concentrated to obtain the desired product as a white solid in 99 % yield. **^{1}H NMR** (500 MHz, CD_3OD) δ 4.51 – 4.48 (m, 1H), 4.31 (dd, *J* = 5, 10 Hz, 1H), 3.66 (s, 3H), 3.21 (quint, *J* = 3.8 Hz, 1H), 2.93 (dd, *J* = 5, 15 Hz, 1H), 2.71 (d, *J* = 5 Hz, 1H), 2.35 (t, *J* = 7.5 Hz, 2H), 1.78 – 1.70 (m, 1H), 1.69 – 1.63 (m, 2H), 1.62 – 1.55 (m, 1H), 1.45 (quint, *J* = 7.5 Hz, 2H) ppm. **^{13}C NMR** (126 MHz, CD_3OD) δ 175.86, 166.14, 63.39, 61.63, 56.95, 52.01, 41.02, 34.55, 29.69, 29.46, 25.91 ppm. **HRMS** (ESI)：m/z calc.for $[C_{11}H_{18}N_2Na_1O_3S_1]^+$ $[M+Na]^+$ 281.09358 found 281.09331.

Synthesis of Compound 11 and 12

To a stirred solution of compound **10** (200 mg, 0.77 mmol) in DMF (10 mL) was added NaH (22 mg, 0.92 mmol) at 0 °C under N_2 atmosphere. 1,6-Dibromohexane (0.11 mL, 0.77 mmol) was added and the mixture was stirred at 0 °C for 3 hours. The reaction was quenched with 1 M hydrochloric acid at 0°C. The reaction mixture was concentrated under reduced pressure to afford compound **11** and **12** which was used in the next step without further purification.

Synthesis of Compound 13 and 14

To a stirred solution of compound **11** and **12** (318 mg) in DMF (5 mL) was added NaN_3 (245 mg, 3.77 mmol) in H_2O (5 mL) and the mixture was stirred at 60 °C for 18 hours. The reaction mixture was concentrated under reduced pressure, then extracted with DCM (3x) and water (1x). The combined organic layers were washed with brine, dried over Na_2SO_4, filtered and concentrated to afford compound **13** and **14** which was used in the next step without further purification.

Synthesis of Compound 15 and 16

To a solution of compound **13** and **14** (317 mg) in THF (5 mL) was added 1 M lithium hydroxide (4.13 mL, 4.13 mmol) and the mixture was stirred at room temperature for 4 hours. The reaction mixture was concentrated under reduced pressure, then purified by reverse-phase column chromatography (ACN/H_2O) to afford compound **15** and **16** as transparent liquid.

Compound **15**: **^{1}H NMR** (700 MHz, Chloroform-*d*) δ 5.71 (s, 1H), 4.41 – 4.36 (m, 1H), 4.21 (dd, J = 9.4, 5.7 Hz, 1H), 3.62 (ddd, J = 14.0, 8.9, 7.1 Hz, 1H), 3.25 (t, J = 6.9 Hz, 2H), 3.18 (ddd, J = 11.5, 5.7, 3.5 Hz, 1H), 2.98 (dd, J = 12.4, 6.7 Hz, 1H), 2.78 (dt, J = 14.0, 4.8 Hz, 2H), 2.32 (t, J = 7.0 Hz, 2H), 1.69 – 1.50 (m, 8H), 1.46 (qd, J = 13.5, 11.8, 3.7 Hz, 1H), 1.38 (dt, J = 15.6, 7.7 Hz, 3H), 1.33 – 1.27 (m, 2H) ppm. **^{13}C NMR** (176 MHz, Chloroform-*d*) δ 177.41, 162.38, 65.62, 57.60, 53.72, 51.23, 42.78, 37.31, 33.81, 28.65, 28.35, 28.15, 26.33, 26.24, 24.43 ppm. **HRMS** (ESI): m/z calc. for $[C_{16}H_{27}N_5Na_1O_3S_1]^+$ $[M+Na]^+$ 392.17323 found 392.17362.

Compound **16**: **^{1}H NMR** (700 MHz, Chloroform-*d*) δ 7.12 (s, 1H), 4.39 (dd, *J* = 8.0, 4.7 Hz, 1H), 4.23 (dd, *J* = 8.0, 4.9 Hz, 1H), 3.43 (ddd, *J* = 14.3, 8.6, 7.2 Hz, 1H), 3.24 (t, *J* = 6.9 Hz, 2H), 3.18 (td, *J* = 7.5, 4.8 Hz, 1H), 2.91 (td, *J* = 8.7, 4.2 Hz, 1H), 2.87 (d, *J* = 13.4 Hz, 1H), 2.80 (dd, *J* = 13.1, 4.8 Hz, 1H), 2.35 (ddd, *J* = 15.4, 9.1, 5.9 Hz, 1H), 2.26 (ddd, *J* = 15.8, 9.1, 6.5 Hz, 1H), 1.76 (dtd, *J* = 16.2, 9.2, 8.2, 3.5 Hz, 1H), 1.68 – 1.63 (m, 2H), 1.61 – 1.53 (m, 4H), 1.50 – 1.42 (m, 2H), 1.37 (tq, *J* = 8.4, 5.5, 4.0 Hz, 3H), 1.30 (tt, *J* = 9.6, 3.7 Hz, 2H) ppm. **^{13}C NMR** (176 MHz, Chloroform-*d*) δ 177.62, 162.84, 63.75, 59.17, 55.27, 51.31, 40.72, 36.44, 33.85, 28.73, 28.47, 28.25, 27.41, 26.38, 26.27, 25.25 ppm. **HRMS** (ESI): m/z calc. for $[C_{16}H_{27}N_5Na_1O_3S_1]^+$ $[M+Na]^+$ 392.17323 found 392.17209.

Synthesis of Compound 17

To a solution of methyl 4-cyanobenzoate (5 g, 31 mmol) in anhydrous THF (50 mL) was added 0.6 M lithium hydroxide solution (200 mL, 120.66 mmol) dropwise and the mixture was stirred at room temperature for 1 hour. It was then heated to 50 °C and stirred for an additional 15 hours. The reaction was quenched with water (5.9 mL) followed by 15% aqueous sodium hydroxide solution (5.9 mL). The mixture was filtered through Celite and rinsed with THF. The filtrate was concentrated under reduced pressure, then extracted with 1 M hydrochloric acid (50 mL) and ethyl acetate (EA, 50 mL). The aqueous layer was collected and concentrated again under reduced pressure. The residue was treated with acetone (50 mL), followed by

vacuum filtration to obtain the desired product as a white solid in 75% yield. **^{1}H NMR** (400 MHz, DMSO-d_6) δ 8.54 (s, 2H), 7.44 (d, *J* = 8.3 Hz, 2H), 7.33 (d, *J* = 8.5 Hz, 2H), 4.49 (s, 2H), 3.96 (q, J = 5.9 Hz, 2H) ppm. **^{13}C NMR** (101 MHz, DMSO-d_6) δ 142.95, 132.37, 128.84, 126.57, 62.52, 42.00 ppm. **HRMS** (ESI): m/z calc. for $[C_8H_{12}N_1O_1]^+$ $[M+H]^+$ 138.09189 found 138.09262.

Synthesis of Compound 18

To a solution of compound **17** and triethylamine (2.02 mL, 23 mmol) in MeOH (23 mL) was added ethyl trifluoroacetate (3.6 mL, 30 mmol) and the mixture was stirred at room temperature for 17 hours. The reaction mixture was extracted with EA and the combined organic layers were washed with brine, dried over Na_2SO_4, filtered and concentrated. The residue was purified by column chromatography (DCM/MeOH) to afford compound **18** as a white solid in 80% yield. **^{1}H NMR** (500 MHz, MeOD) δ 7.33 (d, *J* = 7.9 Hz, 2H), 7.27 (d, *J* = 8.1 Hz, 2H), 4.58 (s, 2H), 4.43 (s, 2H) ppm. **^{13}C NMR** (126 MHz, MeOD) δ 158.96 (q, J = 37.0 Hz), 142.08, 137.36, 128.71, 128.29, 117.54 (q, J = 286.7 Hz), 64.81, 43.98 ppm. **^{19}F NMR** (471 MHz, MeOD) δ -77.24 ppm. **HRMS** (ESI): m/z calc. for $[C_{10}H_9F_3N_1O_2]^-$ $[M-H]^-$ 232.05854 found 232.05908.

Synthesis of Compound 19

To a solution of 6-chloroguanine (4 g, 23.70 mmol) in DMF (23 mL) was added 1-methylpyrrolidine (4.26 g, 49.80 mmol) and the mixture was stirred at room temperature for 24 hours. Acetone (12 mL) was added to the reaction mixture, forming a precipitate, followed by vacuum filtration to obtain the desired product as a white solid in 88% yield. **^{1}H NMR** (400 MHz, DMSO-d_6) δ 8.34 (s, 1H), 7.11 (s, 2H), 4.63 – 4.55 (m, 2H), 4.01 – 3.93 (m, 2H), 3.65 (s, 3H), 2.30 – 2.18 (m, 2H), 2.08 – 2.02 (m, 2H) ppm. **^{13}C NMR** (101 MHz, DMSO-d_6) δ 159.03, 158.68, 151.61, 143.11, 116.01, 64.02, 51.55, 40.15, 39.94, 39.73, 39.52, 39.31, 39.10, 38.90, 21.44 ppm. **HRMS** (ESI): m/z calc. for $[C_{10}H_{14}Cl_1N_6]^-$ $[M-H]^-$ 253.09685 found 253.09692.

Synthesis of Compound 20

To a solution of compound **18** (300 mg, 1.28 mmol) in anhydrous DMF (12.8 mL) was added 90% NaH under N_2 atmosphere. Compound **19** (327 mg, 1.28 mmol) was added after 30 minutes and the mixture was stirred at room temperature for 19 hours. The reaction mixture

was concentrated under reduced pressure, then purified by column chromatography (DCM/MeOH) to afford compound **20** as a white solid in 79% yield. **^{1}H NMR** (500 MHz, DMSO-*d*$_6$) δ 12.43 (s, 1H), 10.02 (s, 1H), 7.81 (s, 1H), 7.49 (d, *J* = 7.8 Hz, 2H), 7.30 (d, *J* = 7.9 Hz, 2H), 6.30 (s, 2H), 5.46 (s, 2H), 4.40 (d, *J* = 5.9 Hz, 2H) ppm. **^{13}C NMR** (126 MHz, DMSO-*d*$_6$) δ 159.77 (d, *J* = 24.2 Hz), 156.42 (q, *J* = 36.2 Hz), 155.23, 137.86, 137.27, 135.98, 129.51, 128.70, 127.49, 116.04 (q, *J* = 288.3 Hz), 113.53, 66.43, 42.41 ppm. **^{19}F NMR** (471 MHz, DMSO-*d*$_6$) δ -74.31 ppm. **HRMS** (ESI): m/z calc. for $[C_{15}H_{13}F_3N_6Na_1O_6]^+$ $[M+Na]^+$ 389.09498 found 389.09558.

Synthesis of Compound 21

To a stirred solution of compound **20** (370 mg, 1.01 mmol) in MeOH (10 mL) was added methylamine solution (33 wt.% in EtOH, 10 mL) and the mixture was stirred at room temperature for 18 hours. The reaction mixture was concentrated under reduced pressure to afford compound **21** which was used in the next step without further purification.

Synthesis of Compound 22

Mixture of compound **21** (176.5 mg, 0.5 mmol), EDC·HCl (286 mg, 1.5 mmol) and HOBt (202 mg, 1.5 mmol) in DMF (5 mL) was stirred for 10 minutes in a reaction flask. Triethylamine (TEA, 0.21 mL) and 6-((((9*H*-fluoren-9-yl)methoxy)carbonyl)amino)hexanoic acid (135 mg, 0.5 mmol) were added and the mixture was stirred at room temperature for 4 hours. The reaction mixture was concentrated using a lyophilizer, then purified by reverse-phase column chromatography (ACN/H_2O) to obtain the desired product as a white solid in 55% yield. **^{1}H NMR** (700 MHz, DMSO-*d*$_6$) δ 8.41 (s, 1H), 8.33 (s, 1H), 7.87 (d, *J* = 7.5 Hz, 2H), 7.67 (d, *J* = 7.5 Hz, 2H), 7.50 – 7.47 (m, 2H), 7.40 (t, *J* = 7.4 Hz, 2H), 7.32 (td, *J* = 7.4, 1.1 Hz, 2H), 7.27 (d, *J* = 7.4 Hz, 2H), 5.51 (d, *J* = 2.0 Hz, 2H), 4.29 (d, *J* = 7.1 Hz, 2H), 4.27 – 4.25 (m, 2H), 4.20 (t, *J* = 7.0 Hz, 1H), 2.96 (d, *J* = 6.7 Hz, 2H), 2.13 (td, *J* = 7.5, 2.5 Hz, 2H), 1.51 (t, *J* = 7.7 Hz, 2H), 1.42 – 1.37 (m, 2H), 1.24 (t, *J* = 7.9 Hz, 2H) ppm. **^{13}C NMR** (176 MHz, DMSO-*d*$_6$) δ 172.19, 158.92, 158.07, 156.13, 153.60, 143.96, 140.86, 140.76, 140.20, 134.03, 128.89, 127.62, 127.28, 127.06, 125.16, 124.81, 120.13, 68.09, 65.19, 46.81, 41.81, 40.16, 35.33, 29.19, 25.98, 25.06 ppm. **HRMS** (ESI): m/z calc. for $[C_{34}H_{35}F_3N_7Na_1O_4]^+$ $[M+Na]^+$ 389.09498 found 389.09558.

Synthesis of Compound 23

To a stirred solution of compound **22** (166 mg, 0.27 mmol) in DCM (2.7 mL) was added diethylamine (1.35 mL) and the mixture was stirred at room temperature for 4 hours. The reaction mixture was concentrated under reduced pressure to afford compound **23** which was used in the next step without further purification.

Synthesis of Compound 24

To a stirred solution of compound **23** (74 mg, 0.2 mmol), EDC·HCl (115 mg, 0.6 mmol) and HOBt (92 mg, 0.6 mmol) in DMF (2 mL) was added compound **15** (77 mg, 0.2 mmol) and TEA (84 µL). The solution was stirred at room temperature for 4 hours. The reaction mixture was concentrated using a lyophilizer, then purified by reverse-phase column chromatography (ACN/H_2O) to obtain the desired product as a white solid in 43% yield. **^{1}H NMR** (700 MHz, DMSO-d_6) δ 8.32 (t, *J* = 5.9 Hz, 1H), 7.74 (d, *J* = 5.2 Hz, 2H), 7.49 (d, *J* = 7.8 Hz, 2H), 7.27 (d, *J* = 7.6 Hz, 2H), 6.57 (s, 1H), 5.52 (s, 2H), 4.26 (d, *J* = 6.2 Hz, 2H), 4.14 (d, *J* = 5.2 Hz, 2H), 3.29 (s, 1H), 3.21 (d, *J* = 5.7 Hz, 1H), 3.00 (d, *J* = 6.6 Hz, 2H), 2.89 – 2.85 (m, 2H), 2.77 – 2.73 (m, 2H), 2.60 (d, *J* = 12.0 Hz, 1H), 2.12 (t, *J* = 7.4 Hz, 2H), 2.03 (t, *J* = 3.3 Hz, 2H), 1.51 (q, *J* = 6.8 Hz, 8H), 1.39 – 1.36 (m, 5H), 1.31 (t, *J* = 8.4 Hz, 3H), 1.22 (d, *J* = 12.2 Hz, 4H) ppm. **^{13}C NMR** (176 MHz, DMSO-d_6) δ 172.21, 171.87, 161.79, 158.98, 158.09, 157.90, 140.17, 134.12, 128.89, 127.28, 126.98, 126.46, 64.13, 57.08, 55.16, 50.61, 42.75, 41.81, 38.36, 37.94, 35.43, 35.35, 29.00, 28.78, 28.22, 26.43, 26.19, 25.93, 25.78, 25.23, 25.09 ppm. **HRMS** (ESI): m/z calc. for $[C_{35}H_{50}N_{12}Na_1O_4S_1]^+$ $[M+Na]^+$ 757.36964 found 757.36936.

Synthesis of Compound 25

Mixture of 4-sulfamoylbenzoic acid (200 mg, 1 mmol), EDC·HCl (210 mg, 1.4 mmol), HOBt (167 mg, 1.4 mmol) and TEA (0.21 mL, 1.4 mmol) in DMF (10 mL) was stirred for 5 minutes in a reaction flask. Propargyl amine (60 mg, 1.4 mmol) was added and the mixture was reacted at room temperature for 3 hours. The reaction mixture was concentrated using a lyophilizer, then purified by reverse-phase column chromatography (ACN/H_2O) to obtain the desired product as a white solid in 70 % yield. **^{1}H NMR** (500 MHz, CD_3OD) δ 7.98 (d, *J* = 5 Hz, 2H), 4.17 (d, *J* = 0 Hz, 2H), 2.64 (t, *J* = 5 Hz, 1H) ppm. **^{13}C NMR** (126 MHz, DMSO-d6) δ 164.97, 146.47, 136.68,

127.99, 125.71, 81.01, 73.12, 28.65 ppm. **HRMS** (ESI)：m/z calc. for $[C_{10}H_9N_2O_3S_1]^-$ $[M-H]^-$ 237.03339 found 237.03315.

Synthesis of Probe 1

To a stirred solution of compound **24** (18 mg, 2.5 µmol) and compound **25** (6.3 mg, 2.7 µmol) in DMF (0.2 mL) was added a solution of sodium ascorbate (5.3 mg, 2.7 µmol) and $CuSO_4$ (4.3 mg, 2.7 µmol) in H_2O (0.05 mL). The solution was stirred at room temperature for 1 hour. The reaction mixture was concentrated using a lyophilizer, then purified by reverse-phase column chromatography (ACN/H_2O) to obtain **probe 1** as a white solid in 48% yield. **^{1}H NMR** (700 MHz, DMSO-d_6) δ 9.19 (q, *J* = 6.2, 5.7 Hz, 1H), 8.32 (q, *J* = 5.8 Hz, 1H), 8.02 (d, *J* = 8.2 Hz, 2H), 7.98 (s, 1H), 7.89 (d, *J* = 8.1 Hz, 2H), 7.74 (q, *J* = 5.6 Hz, 1H), 7.50 (d, *J* = 7.8 Hz, 1H), 7.48 (s, 2H), 7.27 (d, *J* = 7.9 Hz, 1H), 6.57 (s, 1H), 5.54 (s, 2H), 4.52 (d, *J* = 5.6 Hz, 2H), 4.29 (d, *J* = 7.3 Hz, 2H), 4.26 (s, 1H), 4.13 – 4.11 (m, 2H), 3.51 (s, 2H), 3.39 (dd, *J* = 14.5, 7.5 Hz, 1H), 3.20 (dt, *J* = 9.4, 4.5 Hz, 1H), 3.00 (q, *J* = 6.6 Hz, 2H), 2.89 – 2.85 (m, 1H), 2.72 (td, *J* = 9.3, 4.4 Hz, 1H), 2.60 (dd, *J* = 12.3, 2.7 Hz, 1H), 2.11 (q, *J* = 7.3 Hz, 2H), 2.03 (td, *J* = 7.3, 2.5 Hz, 2H), 1.78 (h, *J* = 7.8 Hz, 2H), 1.60 (m, 1H), 1.50 (m, 5H), 1.37 (dq, *J* = 15.8, 8.0, 6.6 Hz, 5H), 1.23 (dt, *J* = 15.6, 8.0 Hz, 7H) ppm. **^{13}C NMR** (176 MHz, DMSO-d_6) δ 172.17, 171.82, 165.15, 161.72, 158.39, 158.19, 154.82, 153.64, 153.01, 146.34, 144.63, 140.32, 137.06, 133.75, 128.95, 127.98, 127.26, 126.95, 126.43, 125.63, 122.88, 69.80, 64.10, 57.03, 55.10, 49.22, 42.68, 41.78, 38.34, 37.89, 35.33, 35.00, 29.69, 28.98, 28.75, 28.20, 26.35, 26.17, 25.63, 25.60, 25.20, 25.06 ppm. **HRMS** (ESI): m/z calc. for $[C_{45}H_{60}N_{14}Na_1O_7S_2]^+$ $[M+Na]^+$ 995.41085 found 995.41101.

Synthesis of Compound 32

Mixture of trimethoprim (50 mg, 0.18 mmol), Cs_2CO_3 (118 mg, 0.36 mmol) and propargyl bromide (81 µL, 0.90 mmol) in DMF (50 mL) was stirred at 70° C under N_2 atmosphere for 3 hours. The reaction mixture was concentrated under reduced pressure, then purified by reverse-phase column chromatography (ACN/H_2O) to obtain the desired product as a white solid in 80 % yield. **^{1}H NMR** (500 MHz, CD_3OD) δ 7.23 (s, 1H), 6.58 (s, 2H), 4.63 (s, 2H), 3.82 (s, 6H), 3.67 (s, 2H), 2.82 (s, 1H) ppm. **^{13}C NMR** (126 MHz, CD_3OD) δ 166.30, 156.17, 155.21, 140.42, 134.47, 110.96, 107.27, 80.24, 76.21, 60.68, 56.72, 33.91 ppm. **HRMS** (ESI)：m/z calc. for $[C_{16}H_{19}N_4O_3]^+$ $[M+H]^+$ 315.14571 found 315.14571.

Synthesis of Probe 3

To a stirred solution of compound **24** (10 mg, 14 µmol) and compound **32** (3 mg, 10 µmol) in DMF (1.3 mL) was added a solution of sodium ascorbate (1.8 mg, 10 µmol) and $CuSO_4$ (1.4 mg, 10 µmol) in H_2O (0.4 mL). The solution was stirred at room temperature for 3 hours. The reaction mixture was purified by reverse-phase column chromatography (ACN/H_2O) to afford **probe 3** as a colorless viscous oil in 35 % yield. **^{1}H NMR** (700 MHz, CD_3OD) δ 7.99 (s, 1H), 7.52 – 7.51 (m, 3H), 7.34 – 7.32 (m, 3H), 7.22 (s, 2H), 6.55 (s, 2H), 5.65 (d, *J* = 7 Hz, 2H), 5.07 (s, 2H), 4.40 – 4.39 (m, 1H), 4.37 (s, 2H), 4.26 (dd, *J* = 10.5 Hz, 1H), 3.78 (s, 6H), 3.66 (s, 2H), 3.26 (t, *J* = 7 Hz, 2H), 3.15 (t, *J* = 7 Hz, 2H), 2.94 – 2.93 (m, 2H), 2.87 – 2.86 (m, 2H), 2.25 (td, *J* = 7, 10.5 Hz, 2H), 2.17 (t, *J* = 7 Hz, 2H), 1.68 – 1.63 (m, 4H), 1.61 – 1.57 (m, 4H), 1.53 – 1.49 (m, 4H), 1.38 – 1.31 (m, 8H) ppm. **^{13}C NMR** (176 MHz, CD_3OD) δ 176.00, 175.94, 166.28, 164.77, 161.14, 156.15, 155.17, 141.06, 140.39, 136.11, 135.36, 134.30, 130.21, 128.79, 125.66, 111.06, 107.23, 70.83, 66.58, 66.32, 59.30, 56.65, 56.53, 51.18, 44.39, 43.72, 43.29, 40.55, 40.20, 38.90, 36.93, 36.82, 33.95, 31.18, 30.10, 29.78, 29.54, 27.85, 27.54, 27.33, 27.14, 27.06, 26.83, 26.65, 26.52 ppm. **HRMS** (ESI)：m/z calc.for $[C_{51}H_{67}Cl_1N_{16}O_7S_1]^-$ $[M\text{-}H]^-$ 1047.50993 found 1047.50978.

Synthesis of Compound 33 and 34

To a stirred solution of compound **10** (1.0 g, 3.85 mmol) in DMF (10 mL) was added 60% NaH (185 mg, 4.6 mmol) at 0 °C under N_2 atmosphere for 15 minutes. Propargyl bromide (458 mg, 3.85 mmol) was added and the mixture was stirred at 0 °C for 3 hours. The reaction was quenched with 3 M hydrochloric acid at 0°C. The reaction mixture was concentrated using a lyophilizer to afford compound **33** and **34** which was used in the next step without further purification.

Synthesis of Compound 35 and 36

To a solution of compound **33** and **34** (114 mg) in THF (5 mL) was added 2.5 M lithium hydroxide (1 mL, 2.5 mmol) and the mixture was stirred at room temperature for 4 hours. The reaction mixture was acidified to pH 2 with 3 M hydrochloric acid and concentrated using a lyophilizer. It was then purified by reverse-phase column chromatography (ACN/H_2O) to afford compound **35** and **36** as a white solid.

Compound **35**: **^{1}H NMR** (700 MHz, DMSO-d_6) δ 4.41 – 4.39 (m, 1H), 4.14 – 4.12 (m, 1H), 4.09 (dd, *J* = 17.9, 2.5 Hz, 1H), 3.75 (dd, *J* = 17.9, 2.4 Hz, 1H), 3.15 (dt, *J* = 8.8, 5.4 Hz, 1H), 2.98 (d, *J* = 12.8 Hz, 1H), 2.78 (dd, *J* = 12.9, 4.9 Hz, 1H), 2.20 (t, *J* = 7.4 Hz, 2H), 1.66 – 1.60 (m, 1H), 1.56 – 1.49 (m, 2H), 1.45 (m, 1H), 1.38 – 1.29 (m, 2H) ppm. **^{13}C NMR** (176 MHz, DMSO-d_6) δ 174.41, 160.36, 79.69, 74.22, 62.77, 58.69, 54.96, 35.91, 33.46, 30.53, 28.09, 27.91, 24.50 ppm. **HRMS** (ESI): m/z calc. for [$C_{13}H_{18}N_2O_3S_1$] [M+] 281.09599 found 281.09592

Compound **36**: **^{1}H NMR** (400 MHz, DMSO-d_6) δ 4.35 – 4.29 (m, 1H), 4.28 – 4.20 (m, 2H), 3.60 (dd, *J* = 17.9, 2.4 Hz, 1H), 3.29 – 3.25 (m, 1H), 2.86 (dd, *J* = 12.3, 5.3 Hz, 1H), 2.62 – 2.59 (d, *J* = 12 Hz, 1H), 2.21 (t, *J* = 7.3 Hz, 2H), 1.80 – 1.69 (m, 1H), 1.60 – 1.33 (m, 5H) ppm. **^{13}C NMR** (101 MHz, DMSO-d_6) δ 174.41, 161.83, 79.34, 75.13, 64.08, 57.32, 55.84, 34.01, 33.50, 28.83, 28.18, 24.39. **HRMS** (ESI): m/z calc. for [$C_{13}H_{18}N_2O_3S_1Na_1$] [M+Na]$^+$ 305.09358 found 305.09409

Synthesis of Compound 37

Mixture of 4-sulfamoylbenzoic acid (500 mg, 2.5 mmol), EDC·HCl (921 mg, 4.8 mmol), HOBt (734 mg, 4.8 mmol) and TEA (808 mg, 8 mmol) in DMF (10 mL) was stirred for 10 minutes in a reaction flask. 3-Azido-1-propanamine (160 mg, 1.6 mmol) was added and the mixture was reacted at room temperature for 4 hours. The reaction mixture was concentrated using a lyophilizer, then extracted with EA and water. The combined organic layers were washed with brine, dried over Na_2SO_4, filtered and concentrated. The residue was purified by reverse-phase column chromatography (ACN/H_2O) to afford compound **37** as a light yellow solid in 60% yield. **^{1}H NMR** (400 MHz, DMSO-d_6) δ 7.99 (d, *J* = 8.6 Hz, 2H), 7.89 (d, *J* = 8.6 Hz, 2H), 3.42 (t, *J* = 6.7 Hz, 2H), 3.37 – 3.31 (m, 2H), 1.79 (p, *J* = 6.8 Hz, 2H) ppm. **^{13}C NMR** (101 MHz, DMSO-d_6) δ 164.79, 145.68, 136.87, 127.34, 125.09, 47.99, 36.26, 27.77. **HRMS** (ESI): m/z calc. for [$C_{10}H_{13}N_5O_3S_1$] [M+]$^+$ 282.06608 found 282.06595.

Synthesis of Compound 38

To a stirred solution of compound **35** (30 mg, 0.11 mmol) and compound **37** (104 mg, 0.21 mmol) in DMF (2 mL) was added a solution of sodium ascorbate (21 mg, 0.11 mmol) and $CuSO_4$ (17 mg, 0.11 mmol) in H_2O (0.5 mL). The solution was stirred at room temperature for 3 hours. The reaction mixture was concentrated using a lyophilizer, then purified by reverse-phase column chromatography (ACN/H_2O) to obtain the desired product as a white solid in 40% yield.

^{1}H NMR (700 MHz, DMSO-d_6) δ 8.01 (s, 1H), 7.99 (d, J = 8.4 Hz, 2H), 7.90 (d, J = 8.4 Hz, 2H), 4.68 (d, J = 15.7 Hz, 1H), 4.42 (t, J = 7.0 Hz, 2H), 4.21 –4.18 (m, 1H), 4.06 – 3.99 (m, 2H), 3.30 –3.27 (m, 2H), 3.21 –3.18 (m, 1H), 2.84 (dd, J = 12.4, 5.5 Hz, 1H), 2.60 (dd, J = 12.4, 2.1 Hz, 1H), 2.21 (t, J = 7.4 Hz, 2H), 2.09 (p, J = 7.0 Hz, 2H), 1.80 – 1.75 (m, 1H), 1.58 – 1.33 (m, 5H) ppm. **^{13}C NMR** (176 MHz, DMSO-d_6) δ 173.90, 164.88, 161.42, 145.70, 142.51, 136.82, 127.35, 127.32, 125.08, 122.96, 63.59, 56.68, 55.07, 46.77, 38.36, 37.75, 36.15, 33.02, 29.21, 28.28, 27.85, 23.90. **HRMS** (ESI): m/z calc. for [$C_{23}H_{31}N_7O_6S_2$] $[M+]^+$ 588.16749 found 588.16738.

Synthesis of Probe 4

Mixture of compound **38** (20 mg, 0.04 mmol), EDC·HCl (20 mg, 0.11 mmol), HOBt (14 mg, 0.11 mmol) and TEA (11 mg, 0.11 mmol) in DMF (1 mL) was stirred for 10 minutes in a reaction flask. Compound **23** (27 mg, 0.07 mmol) was added and the mixture was reacted at room temperature for 4 hours. The reaction mixture was concentrated using a lyophilizer, then purified by reverse-phase column chromatography (ACN/H_2O) to obtain **probe 4** as a white solid in 10% yield. **^{1}H NMR** (700 MHz, DMSO-d_6) δ 8.01 (s, 1H), 7.98 (s, 2H), 7.89 (s, 2H), 7.50 (d, J = 8.0 Hz, 2H), 7.27 (d, J = 7.9 Hz, 2H), 5.54 (s, 1H), 4.69 (s, 2H), 4.42 (s, 2H), 4.26 (d, J = 6.0 Hz, 2H), 4.20 (dd, J = 5.6, 2.9 Hz, 2H), 4.03 – 4.02 (m, 2H), 3.29 – 3.28 (m, 2H), 3.20 – 3.19 (m, 1H), 3.00 (q, J = 6.6 Hz, 2H), 2.85 – 2.81 (m, 2H), 2.23 – 2.20 (m, 2H), 2.12 (t, J = 7.5 Hz, 2H), 2.09 (s, 2H), 1.57 – 1.41 (m, 10H) ppm. **^{13}C NMR** (176 MHz, DMSO-d_6) δ 173.88, 171.61, 171.28, 164.87, 161.39, 161.38, 157.80, 157.60, 145.71, 142.49, 136.81, 128.42, 127.34, 126.71, 125.07, 122.94, 116.07, 114.41, 63.58, 56.67, 55.09, 55.06, 46.76, 37.73, 36.15, 34.77, 33.02, 29.20, 28.49, 28.44, 28.40, 28.30, 28.27, 27.88, 27.85, 25.64, 24.73, 24.52, 23.89. **HRMS** (ESI): m/z calc. for [$C_{42}H_{54}N_{14}O_7S_2$] $[M+]^+$ 929.36630 found 929.36678.

Synthesis of Compound 39

To a stirred solution of compound **23** (20 mg, 0.06 mmol), EDC·HCl (10 mg, 0.06 mmol) and HOBt (8 mg, 0.06 mmol) in DMF (0.5 mL) was added compound **16** (19 mg, 0.05 mmol) and TEA (21 μL). The solution was stirred at room temperature for 4 hours. The reaction mixture was concentrated using a lyophilizer, then purified by reverse-phase column chromatography (ACN/H_2O) to obtain the desired product as a white solid in 27% yield. **^{1}H NMR** (700 MHz, DMSO-d_6) δ 8.43 (s, 1H), 8.32 (t, J = 6.0 Hz, 1H), 7.74 (q, J = 6.9, 6.2 Hz, 2H), 7.49 (d, J = 7.9 Hz,

2H), 7.27 (d, *J* = 7.9 Hz, 2H), 5.52 (s, 2H), 4.33 (d, *J* = 6.6 Hz, 1H), 4.26 (d, *J* = 6.0 Hz, 2H), 4.08 – 4.06 (m, 1H), 3.30 (s, 1H), 3.23 (s, 1H), 3.14 – 3.11 (m, 2H), 2.99 (s, 2H), 2.83 (d, *J* = 5.0 Hz, 2H), 2.75 (d, *J* = 5.1 Hz, 1H), 2.12 (s, 2H), 2.03 (s, 2H), 1.51 (t, *J* = 7.4 Hz, 6H), 1.47 – 1.43 (m, 4H), 1.38 – 1.35 (m, 4H), 1.31 (d, *J* = 7.3 Hz, 2H), 1.23 (s, 4H) ppm. **^{13}C NMR** (176 MHz, DMSO-d_6) δ 172.25, 171.98, 161.00, 158.91, 158.31, 158.12, 140.21, 134.07, 128.92, 127.29, 126.99, 126.47, 62.69, 58.64, 55.36, 50.63, 35.44, 29.00, 28.23, 27.97, 26.92, 26.19, 25.90, 25.82, 25.38, 25.09 ppm. **HRMS** (ESI): m/z calc. for $[C_{35}H_{50}N_{12}Na_1O_4S_1]^+$ $[M+Na]^+$ 757.36964 found 757.36826.

Synthesis of Probe 5

To a stirred solution of compound **39** (7.7 mg, 9.5 µmol) and compound **25** (2.7 mg, 11 µmol) in DMF (0.1 mL) was added a solution of sodium ascorbate (2.2 mg, 6.5 µmol) and $CuSO_4$ (2 mg, 6.5 µmol) in H_2O (25 µL). The solution was stirred at room temperature for 1 hour. The reaction mixture was concentrated using a lyophilizer, then purified by reverse-phase column chromatography (ACN/H_2O) to obtain **probe 5** as a white solid in 53% yield. **^{1}H NMR** (700 MHz, DMSO-d_6) δ 9.19 (t, *J* = 5.7 Hz, 1H), 8.32 (t, *J* = 6.1 Hz, 1H), 8.02 (d, *J* = 8.5 Hz, 2H), 7.98 (s, 1H), 7.89 (d, *J* = 8.4 Hz, 2H), 7.74 (s, 1H), 7.50 (d, *J* = 8.0 Hz, 1H), 7.48 (s, 2H), 7.27 (d, *J* = 8.1 Hz, 1H), 6.51 (s, 1H), 5.54 (s, 2H), 4.52 (d, *J* = 5.7 Hz, 2H), 4.30 (s, 2H), 4.25 (s, 1H), 4.22 (s, 1H), 4.07 – 4.06 (m, 2H), 3.21 (dt, *J* = 13.6, 7.9 Hz, 1H), 3.14 – 3.10 (m, 1H), 3.00 (q, *J* = 6.6 Hz, 2H), 2.81 (d, *J* = 12.8 Hz, 2H), 2.74 – 2.71 (m, 1H), 2.12 (t, *J* = 7.4 Hz, 2H), 2.03 (t, *J* = 7.5 Hz, 2H), 1.77 (q, *J* = 6.9 Hz, 2H), 1.60 (m, 1H), 1.54 – 1.47 (m, 4H), 1.43 (td, *J* = 10.6, 9.0, 4.8 Hz, 2H), 1.37 (p, *J* = 7.3 Hz, 4H), 1.29 (dtd, *J* = 9.5, 6.4, 2.8 Hz, 2H), 1.22 (d, *J* = 9.0 Hz, 5H) ppm. **^{13}C NMR** (176 MHz, DMSO-d_6) δ 172.19, 171.90, 165.16, 160.94, 158.33, 158.12, 154.86, 153.62, 146.34, 144.65, 140.35, 137.07, 133.71, 128.97, 127.99, 127.46, 127.26, 126.95, 125.63, 122.90, 62.64, 58.60, 55.33, 49.23, 41.78, 40.43, 38.33, 35.98, 35.32, 35.24, 35.01, 29.69, 28.98, 28.21, 27.95, 26.82, 26.17, 25.63, 25.57, 25.35, 25.06 ppm. **HRMS** (ESI): m/z calc. for $[C_{45}H_{60}N_{14}Na_1O_7S_2]^+$ $[M+Na]^+$ 971.41325 found 971.41456.

Synthesis of Compound 40

To a stirred solution of compound **36** (60 mg, 0.21 mmol) and compound **37** (104 mg, 0.21 mmol) in DMF (4 mL) was added a solution of sodium ascorbate (42 mg, 0.21 mmol) and $CuSO_4$ (34 mg, 0.21 mmol) in H_2O (1 mL). The solution was stirred at room temperature for 3 hours. The reaction mixture was concentrated using a lyophilizer, then purified by reverse-phase column chromatography (ACN/H_2O) to obtain the desired product as a white solid in 20% yield. **^{1}H NMR** (700 MHz, DMSO-d_6) δ 8.01 (s, 1H), 7.98 (d, *J* = 8.4 Hz, 2H), 7.90 (d, *J* = 8.4 Hz, 2H), 4.52 (d, *J* = 15.7 Hz, 1H), 4.41 (t, *J* = 7.0 Hz, 2H), 4.27 –4.26 (m, 1H), 4.13 (d, *J* = 15.7 Hz, 1H), 4.06 (m, 1H), 3.28 (q, *J* = 6.6 Hz, 2H), 3.13 (dt, *J* = 8.9, 5.4 Hz, 1H), 2.92 (d, *J* = 12.8 Hz, 1H), 2.72 (dd, *J* = 12.8, 4.9 Hz, 1H), 2.20 (t, *J* = 7.4 Hz, 2H), 2.08 (p, *J* = 6.9 Hz, 2H), 1.65 – 1.60 (m, 1H), 1.55 – 1.43 (m, 3H), 1.37 – 1.28 (m, 2H) ppm. **^{13}C NMR** (176 MHz, DMSO-d_6) δ 174.89, 165.86, 161.10, 146.70, 143.82, 137.81, 128.34, 126.07, 123.78, 63.35, 59.10, 55.66, 47.77, 37.16, 36.47, 36.42, 33.95, 30.19, 28.58, 28.40, 25.00. **HRMS** (ESI): m/z calc. for [$C_{23}H_{31}N_7O_6S_2$] [M+]$^+$ 588.16749 found 588.16827.

Synthesis of Probe 6

Mixture of compound **40** (20 mg, 0.04 mmol), EDC·HCl (20 mg, 0.11 mmol), HOBt (14 mg, 0.11 mmol) and TEA (11 mg, 0.11 mmol) in DMF (1 mL) was stirred for 10 minutes in a reaction flask. Compound **23** (27 mg, 0.07 mmol) was added and the mixture was reacted at room temperature for 4 hours. The reaction mixture was concentrated using a lyophilizer, then purified by reverse-phase column chromatography (ACN/H_2O) to obtain **probe 6** as a white solid in 20% yield. **^{1}H NMR** (500 MHz, DMSO-d_6) δ 8.00 (s, 1H), 7.97 (d, *J* = 8.5 Hz, 2H), 7.88 (d, *J* = 8.5 Hz, 2H), 7.49 (d, *J* = 8.0 Hz, 2H), 7.46 (s, 1H), 7.26 (d, *J* = 7.9 Hz, 2H), 4.51 (d, *J* = 15.6 Hz, 1H), 4.40 (t, *J* = 7.0 Hz, 2H), 4.25 (d, *J* = 6.2 Hz, 2H), 4.11 (d, *J* = 15.6 Hz, 1H), 4.04 (m, 1H), 3.27 (q, *J* = 6.5 Hz, 2H), 3.14 – 3.08 (m, 1H), 2.99 (q, *J* = 6.6 Hz, 2H), 2.70 (dd, *J* = 12.9, 4.9 Hz, 1H), 2.09 (m, 4H), 2.02 (t, *J* = 7.5 Hz, 2H), 1.61 (m, 1H), 1.54 – 1.41 (m, 5H), 1.35 (q, *J* = 7.2 Hz, 2H), 1.31 – 1.18 (m, 4H) ppm. **^{13}C NMR** (126 MHz, DMSO-d_6) δ 171.60, 171.30, 164.87, 160.12, 158.18, 157.77, 157.50, 145.70, 142.80, 139.79, 136.81, 133.21, 128.41, 127.33, 125.07, 67.88, 62.36, 58.09, 54.70, 46.77, 41.24, 36.16, 35.39, 34.77, 34.69, 29.19, 28.44, 27.69, 27.40, 25.63, 24.81, 24.51. **HRMS** (ESI): m/z calc. for [$C_{42}H_{54}N_{14}O_7S_2$] [M+]$^+$ 929.36630 found 929.36624.

Synthesis of Compound 41

To a stirred solution of 1,8-diamino-3,6-dioxaoctane (4.0 g, 27 mmol) in DCM (25 mL) was added dropwise di-tert-butyl decarbonate (1.96 g, 9 mmol) in DCM (25 mL) at 0 °C. The resulting mixture was stirred at room temperature for 12 hours. The mixture was extracted with DCM (3x) and water (1x). The combined organic layers were washed with brine, dried over Na_2SO4, filtered and concentrated to obtain the desired product as a colorless oil in 80 % yield. **^{1}H NMR** (500 MHz, CD_3OD) δ：3.61 (s, 4H), 3.52 (dd, *J* = 5, 10 Hz, 4H), 3.22 (t, *J* = 5 Hz, 2H), 2.78 (t, *J* = 5 Hz, 2H), 1.44 (s, 9H) ppm. **^{13}C NMR** (126 MHz, CD_3OD) δ：158.41, 80.04, 73.57, 71.30, 71.24, 71.03, 54.84, 42.09, 41.22, 28.76 ppm. **HRMS** (ESI)：m/z calc.for $[C_{11}H_{25}N_2O_4Na]^+$ $[M+Na]^+$ 249.18143 found 249.18131.

Synthesis of Compound 42

To a stirred solution of compound **41** (500 mg, 2 mmol) and TEA (0.56 mL, 4 mmol) in DCM (5 mL) was added succinic anhydride (201 mg, 2 mmol) and the mixture was reacted for 12 hours. The reaction mixture was acidified to pH 2 with 1 M hydrochloric acid, then extracted with DCM (3x) and water (1x). The combined organic layers were washed with brine, dried over Na_2SO_4, filtered and concentrated to obtain the desired product as a colorless oil in 90 % yield. **^{1}H NMR** (500 MHz, CD_3OD) δ：3.61 (s, 4H), 3.55 – 3.50 (m, 4H), 3.36 (t, *J* = 5 Hz, 2H), 3.23 (t, *J* = 7.5 Hz, 2H), 2.59 (t, *J* = 5 Hz, 2H), 2.48 (t, *J* = 7.5 Hz, 2H), 1.44 (s, 9H) ppm. **^{13}C NMR** (126 MHz, CD_3OD) δ：176.16, 174.58, 158.40, 80.05, 71.26, 71.04, 70.56, 54.83, 41.18, 40.35, 31.48, 30.27, 28.75 ppm. **HRMS** (ESI): m/z calc. for $[C_{15}H_{27}N_2O_7]^-$ $[M-H]^-$ 347.18183 found 347.18133.

Synthesis of Compound 43

To a stirred solution of 2-(2-aminoethoxy)ethanol (4.0 g, 38.04 mmol) in EtOH (80 mL) was added dropwise di-tert-butyl decarbonate (9.13 g, 41.9 mmol) in DCM (10 mL). The resulting mixture was stirred at room temperature for 12 hours. The mixture was extracted with DCM (3x) and water (1x). The combined organic layers were washed with brine, dried over Na_2SO4, filtered and concentrated to obtain the desired product as a yellow viscous oil in 90 % yield. **^{1}H NMR** (400 MHz, $CDCl_3$) δ：5.31 (br, 1H), 3.62 – 3.59 (m, 2H), 3.47 – 3.41 (m, 4H), 3.22 –

3.18 (m, 2H), 1.32 (s, 9H) ppm. **^{13}C NMR** (101 MHz, $CDCl_3$) δ：156.07, 79.00, 72.10, 70.05, 61.24, 40.12, 28.18 ppm.

Synthesis of Compound 44

To a stirred solution of compound **43** (5.0 g, 24.36 mmol) in THF (15 mL) and DMF (15 mL) was added NaH (975 mg, 36.54 mmol) at 0 °C under N_2 atmosphere. 1-Chloro-6-iodohexane (8.4 g, 34.1 mmol) was added and the mixture was stirred at room temperature for 12 hours. The reaction was quenched with saturated ammonium chloride solution (20 mL) at 0°C and extracted with EA. The combined organic layers were dried over Na_2SO_4, filtered and evaporated to dryness. The crude mixture was separated by column chromatography (EA/HEX) to obtain the desired product as yellow viscous oil in 45 % yield. **^{1}H NMR** (400 MHz, $CDCl_3$) δ 4.99 (br, 1H), 3.59 – 3.48 (m, 8H), 3.43 (t, *J* = 6.6 Hz, 2H), 3.28 (q, *J* = 5.3 Hz, 2H), 1.75 (quint, *J* = 7 Hz, 2H), 1.58 (quint, *J* = 7 Hz, 2H), 1.47 – 1.41 (m, 11H), 1.38 – 1.32 (m, 2H) ppm. **^{13}C NMR** (101 MHz, $CDCl_3$) δ 155.93, 79.08, 71.20, 70.19, 70.14, 69.96, 44.97, 40.27, 32.46, 29.36, 28.35, 26.62, 25.35 ppm. **HRMS** (ESI): m/z calc. for $[C_{15}H_{30}Cl_1N_1Na_1O_4]^+$ $[M+Na]^+$ 346.17610 found 346.17689.

Synthesis of Compound 45

To a solution of compound **44** (100 mg, 0.31 mmol) in DCM (1 ml) was added trifluoroacetic acid (1 mL), with ice-bath cooling. The reaction mixture was stirred at room temperature for 2 hours, then concentrated under reduced pressure to afford compound **45** which was used in the next step without further purification.

Synthesis of Compound 46

Mixture of compound **42** (50 mg, 0.014 mmol), HOBt (39 mg, 0.028 mmol), EDC·HCl (55 mg, 0.028 mmol) and TEA (60 µL, 0.042 mmol) in DMF (5 mL) was stirred for 5 minutes in a reaction flask. Compound **45** (39 mg, 0.017 mmol) was added and the mixture was reacted for 3 hours. The reaction mixture was concentrated under reduced pressure, then purified by reverse-phase column chromatography (ACN/H_2O) to obtain the desired product as a colorless viscous oil in 70 % yield. **^{1}H NMR** (500 MHz, CD_3OD) δ 3.61 (s, 4H), 3.60 – 3.56 (m, 6H), 3.55 – 3.48 (m, 10H), 3.34 (td, *J* = 7.5, 5 Hz, 4H), 3.23 (t, *J* = 7.5 Hz, 2H), 2.49 (s, 4H), 1.77 (quint, *J* = 7.5 Hz,

2H), 1.60 (quint, J = 7.5 Hz, 2H), 1.50 – 1.36 (m, 13H) ppm. **^{13}C NMR** (126 MHz, CD_3OD) δ 174.68, 174.64, 158.39, 80.06, 72.20, 71.28, 71.27, 71.25, 71.16, 71.07, 70.58, 70.54, 45.69, 41.22, 40.41, 40.37, 33.72, 32.21, 30.51, 28.77, 27.71, 26.46 ppm. **HRMS** (ESI): m/z calc. for $[C_{25}H_{48}Cl_1N_3Na_1O_8]^+$ $[M+Na]^+$ 576.30276 found 576.30279.

Synthesis of Compound 47

To a solution of compound **46** (100 mg, 0.18 mmol) in DCM (1 mL) was added trifluoroacetic acid (1 mL), with ice-bath cooling. The reaction mixture was stirred at room temperature for 2 hours, then concentrated under reduced pressure to afford compound **47** which was used in the next step without further purification.

Synthesis of Compound 48

Mixture of compound **15** (20 mg, 0.05 mmol), EDC·HCl (19 mg, 0.1 mmol), HOBt (14 mg, 0.1 mmol) and TEA (21 µL, 0.15mmol) in DMF (3 mL) was stirred for 5 minutes in a reaction flask. Compound **47** (27 mg, 0.06 mmol) was added and the mixture was reacted for 4 hours. The reaction mixture was concentrated under reduced pressure, then purified by reverse-phase column chromatography (ACN/H_2O) to obtain the desired product as a colorless oil in 75 % yield. **^{1}H NMR** (400 MHz, CD_3OD) δ 4.43 – 4.39 (m, 1H), 4.30 (dd, J = 5, 10 Hz, 1H), 3.62 (s, 4H), 3.61 – 3.58 (m, 6H) , 3.56 – 3.52 (m, 8H), 3.49 (t, J = 10 Hz, 2H), 3.38 – 3.35 (m, 6H), 3.29 (t, J = 5 Hz, 2H), 2.98 (dd, J = 5, 12.5 Hz, 1H), 2.94 – 2.88 (m, 1H), 2.75 (dd, J = 5, 12.5 Hz, 1H), 2.49 (s, 4H), 2.22 (t, J = 7.5 Hz, 2H), 1.77 (quint, J = 7.5 Hz, 2H), 1.73 – 1.65 (m, 2H), 1.65 – 1.52 (m, 8H), 1.50 – 1.38 (m, 8H), 1.36 – 1.29 (m, 2H) ppm. **^{13}C NMR** (101 MHz, CD_3OD) δ 176.09, 174.72, 174.66, 164.74, 72.23, 71.33, 71.32, 71.27, 71.18, 70.64, 70.59, 70.55, 66.57, 59.30, 56.50, 52.37, 45.71, 44.38, 40.44, 40.39, 40.32, 38.89, 36.84, 33.74, 32.23, 32.21, 30.52, 30.06, 29.83, 29.79, 27.96, 27.73, 27.52, 27.36, 26.73, 26.48 ppm. **HRMS** (ESI) ： m/z calc.for $[C_{36}H_{65}Cl_1N_8Na_1O_8S_1]^+$ $[M+Na]^+$ 827.42323 found 827.42302.

Synthesis of Probe 7

To a stirred solution of compound **48** (15 mg, 19 µmol) and compound **25** (4.7 mg, 20 µmol) in DMF (1.3 mL) was added a solution of sodium ascorbate (3.7 mg, 19 µmol) and $CuSO_4$ (2.9 mg, 19 µmol) in H_2O (0.4 mL). The reaction mixture was stirred at room temperature for 3

hours. The crude mixture was purified by reverse-phase column chromatography (ACN/H_2O) to afford **probe 7** as a colorless viscous oil in 65 % yield. **^{1}H NMR** (700 MHz, CD_3OD) δ 7.99 (s, 4H), 4.66 (s, 1H), 4.41 – 4.38 (m, 2H), 4.26 (dd, *J* = 7, 7 Hz, 1H), 3.61 (s, 4H), 3.59 – 3.56 (m, 6H) , 3.55 – 3.51 (m, 8H), 3.48 (t, *J* = 5 Hz, 2H), 3.37 – 3.34 (m, 8H), 3.28 – 3.26 (m, 1H), 2.98 (dd, *J* = 7, 14 Hz, 1H), 2.89 – 2.85 (m, 1H), 2.75 (dd, *J* = 10.5 Hz, 1H), 2.48 (s, 4H), 2.21 (t, *J* = 7 Hz, 2H), 1.93 – 1.89 (m, 2H), 1.77 (quint, *J* = 7 Hz, 2H), 1.71 – 1.65 (m, 2H), 1.62 – 1.45 (m, 8H), 1.42 – 1.29 (m, 8H) ppm. **^{13}C NMR** (176 MHz, CD_3OD) δ 176.11, 174.72, 174.66, 164.76, 147.85, 129.17, 127.36, 72.23, 71.31, 71.27, 71.17, 70.64, 70.59, 70.56, 66.52, 59.28, 56.48, 51.35, 49.85, 49.53, 45.72, 44.24, 40.43, 40.38, 40.32, 38.89, 36.85, 36.48, 33.74, 32.25, 32.23, 31.05, 30.52, 30.05, 29.77, 27.80, 27.73, 27.06, 26.72, 26.47 ppm. **HRMS** (ESI) ： m/z calc.for $[C_{46}H_{76}Cl_1N_{10}O_{11}S_2]^+$ $[M+H]^+$1043.4819 found 1043.4829.

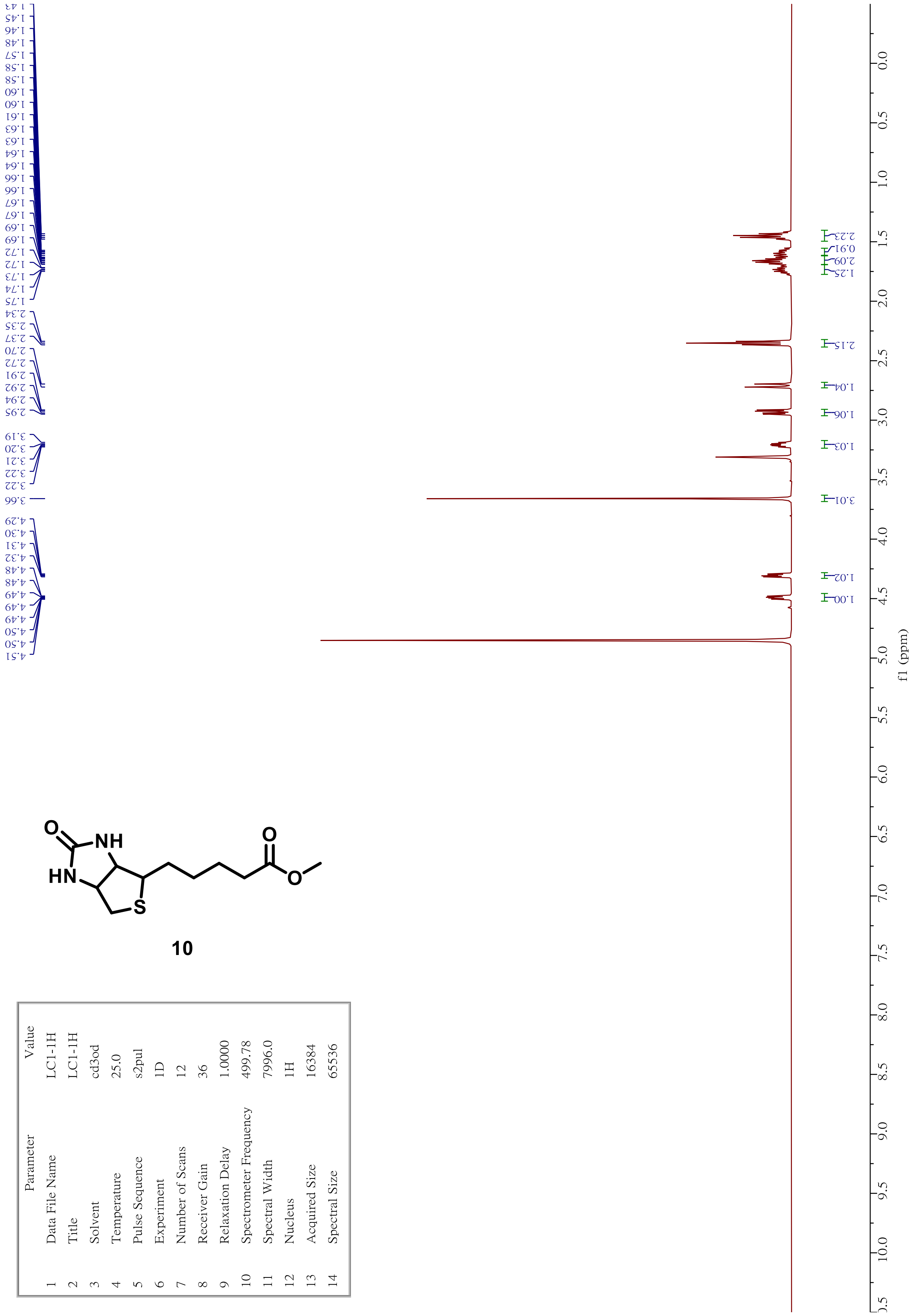

Figure S16. ^{1}H NMR spectra of compound **10**.

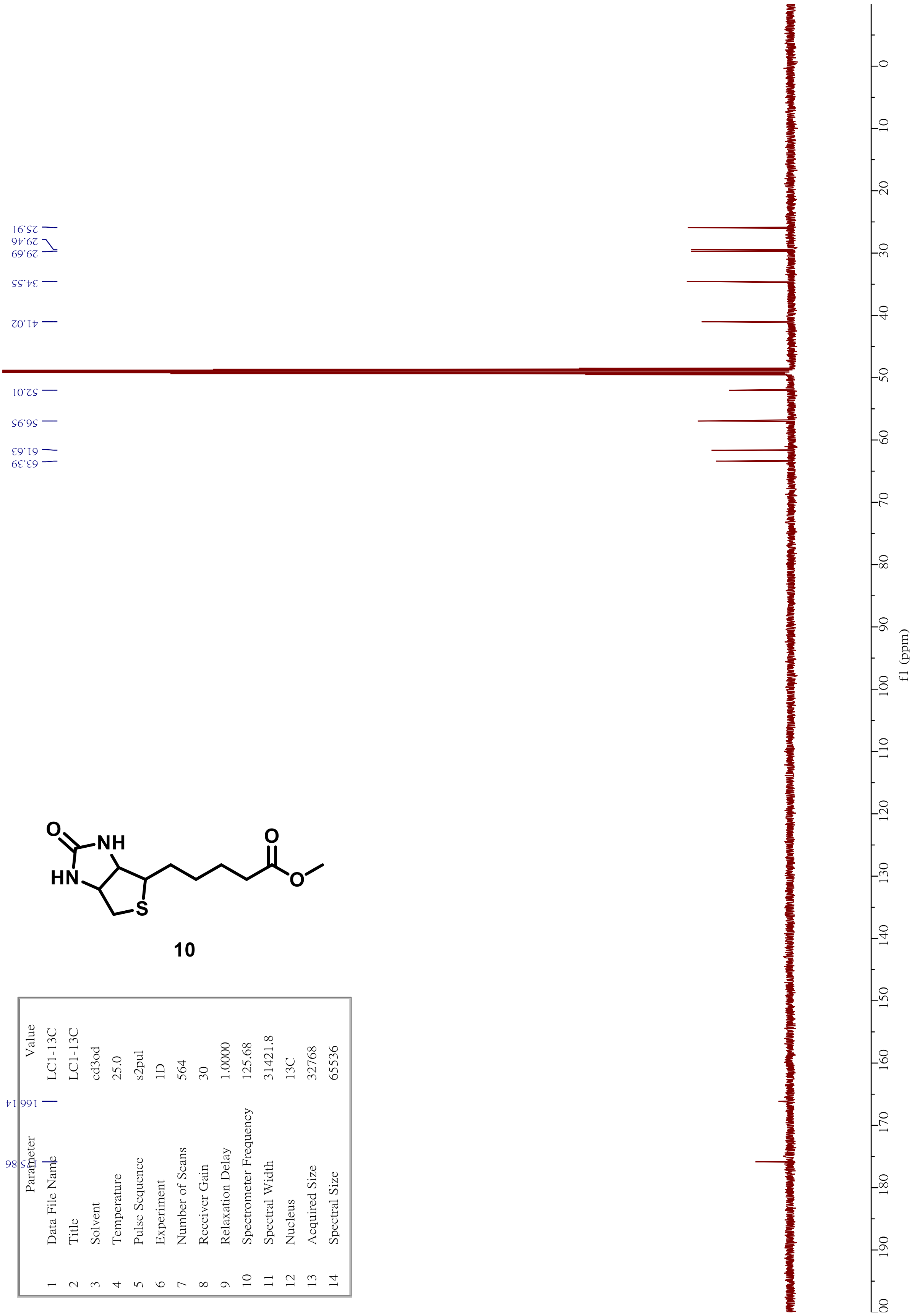

Figure S17. ^{13}C NMR spectra of compound **10**.

Data:XY53
Comment:
Description:
Ionization Mode:ESI+
History:Average(MS[1] 0.56..0.59)

Acquired:3/17/2021 2:48:03 PM
Operator:AccuTOF
m/z Calibration File:20210225-TFANa_...
Created:3/17/2021 5:01:09 PM
Created by:AccuTOF

Charge number:1
Tolerance:200.00[ppm], 200.00 .. 200....
Unsaturation Number:-100.5 .. 200.0 (...
Element:^{12}C:11 .. 11, ^{1}H:0 .. 19, ^{14}N:2 .. 2, ^{23}Na:0 .. 1, ^{16}O:3 .. 3, ^{32}S:1 .. 1

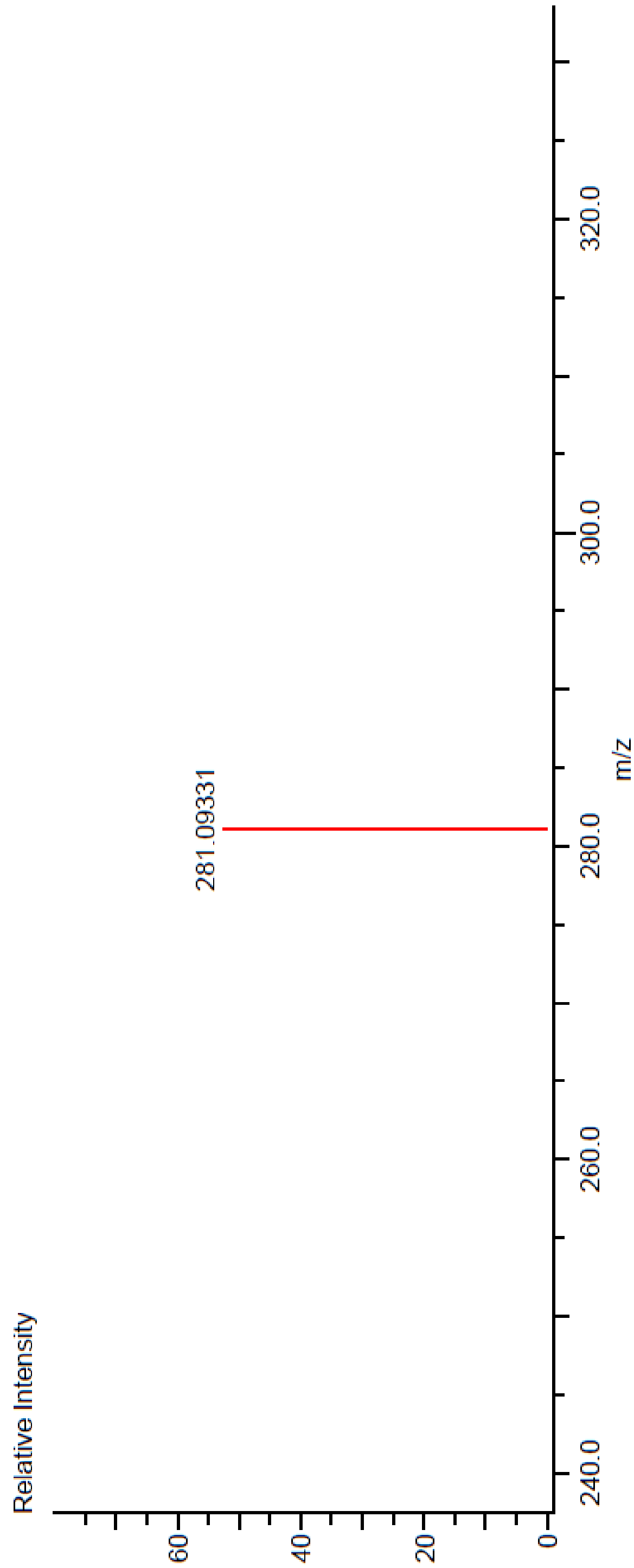

Mass	Intensity	Calc. Mass	Mass Difference [mDa]	Mass Difference [ppm]	Possible Formula
281.09331	952.63	281.09358	-0.27	-0.96	$^{12}C_{11}{}^{1}H_{18}{}^{14}N_{2}{}^{23}Na_{1}{}^{16}O_{3}{}^{32}S_{1}$

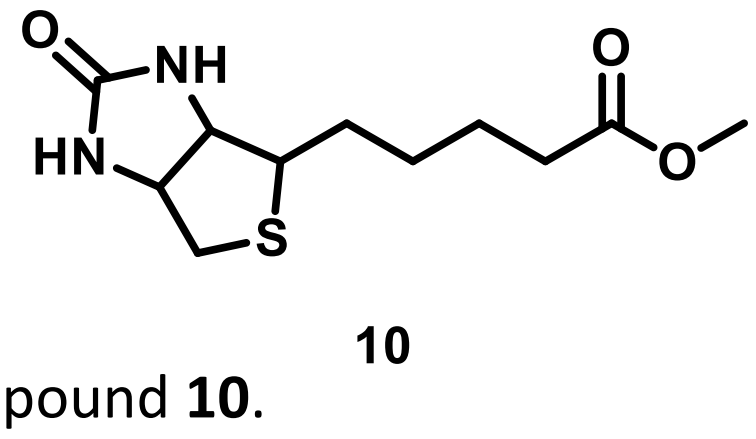

10

Figure S18. HRMS-ESI spectra of compound **10**.

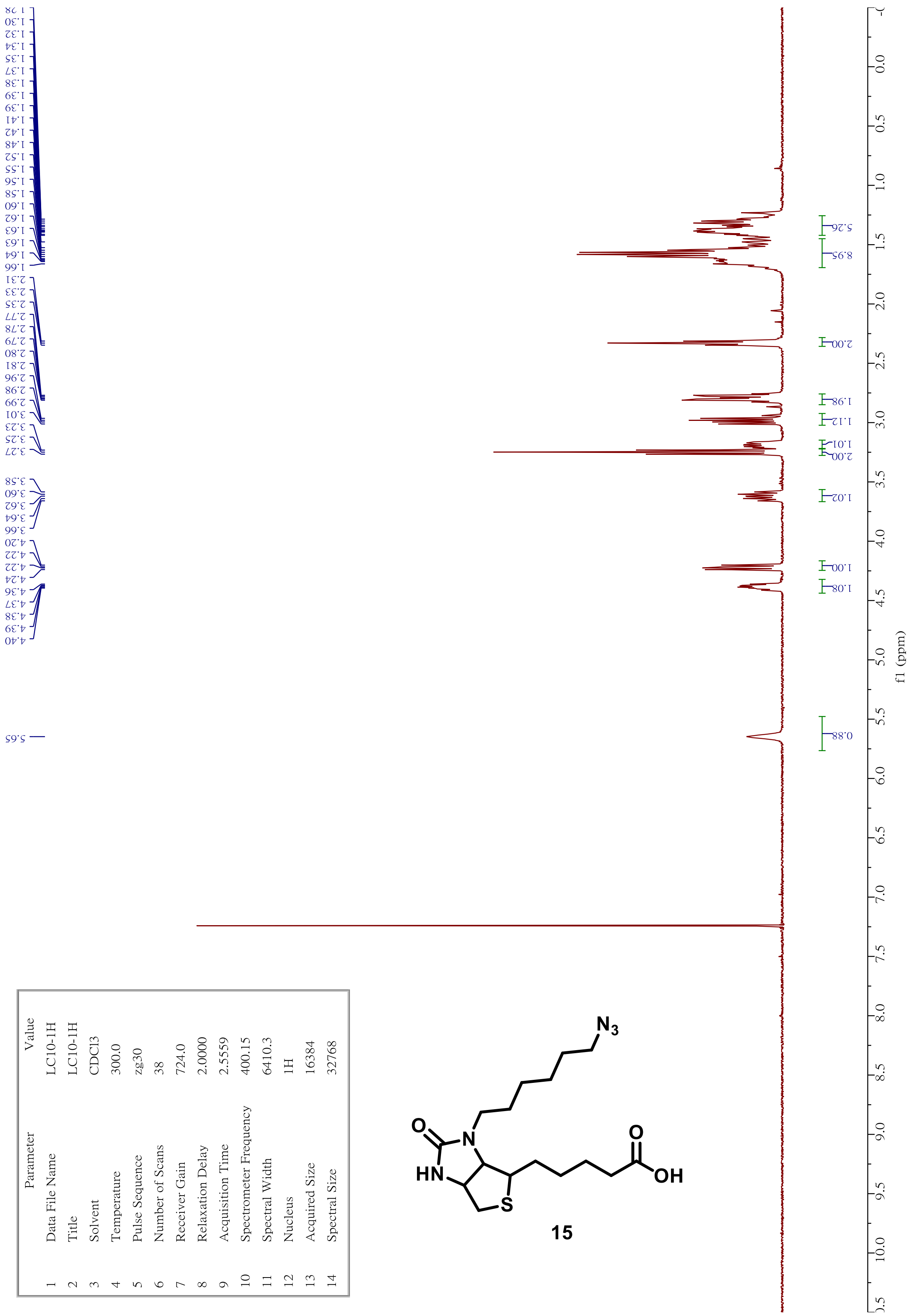

	Parameter	Value
1	Data File Name	LC10-1H
2	Title	LC10-1H
3	Solvent	CDCl3
4	Temperature	300.0
5	Pulse Sequence	zg30
6	Number of Scans	38
7	Receiver Gain	724.0
8	Relaxation Delay	2.0000
9	Acquisition Time	2.5559
10	Spectrometer Frequency	400.15
11	Spectral Width	6410.3
12	Nucleus	1H
13	Acquired Size	16384
14	Spectral Size	32768

Figure S19. ^{1}H NMR spectra of compound **15**.

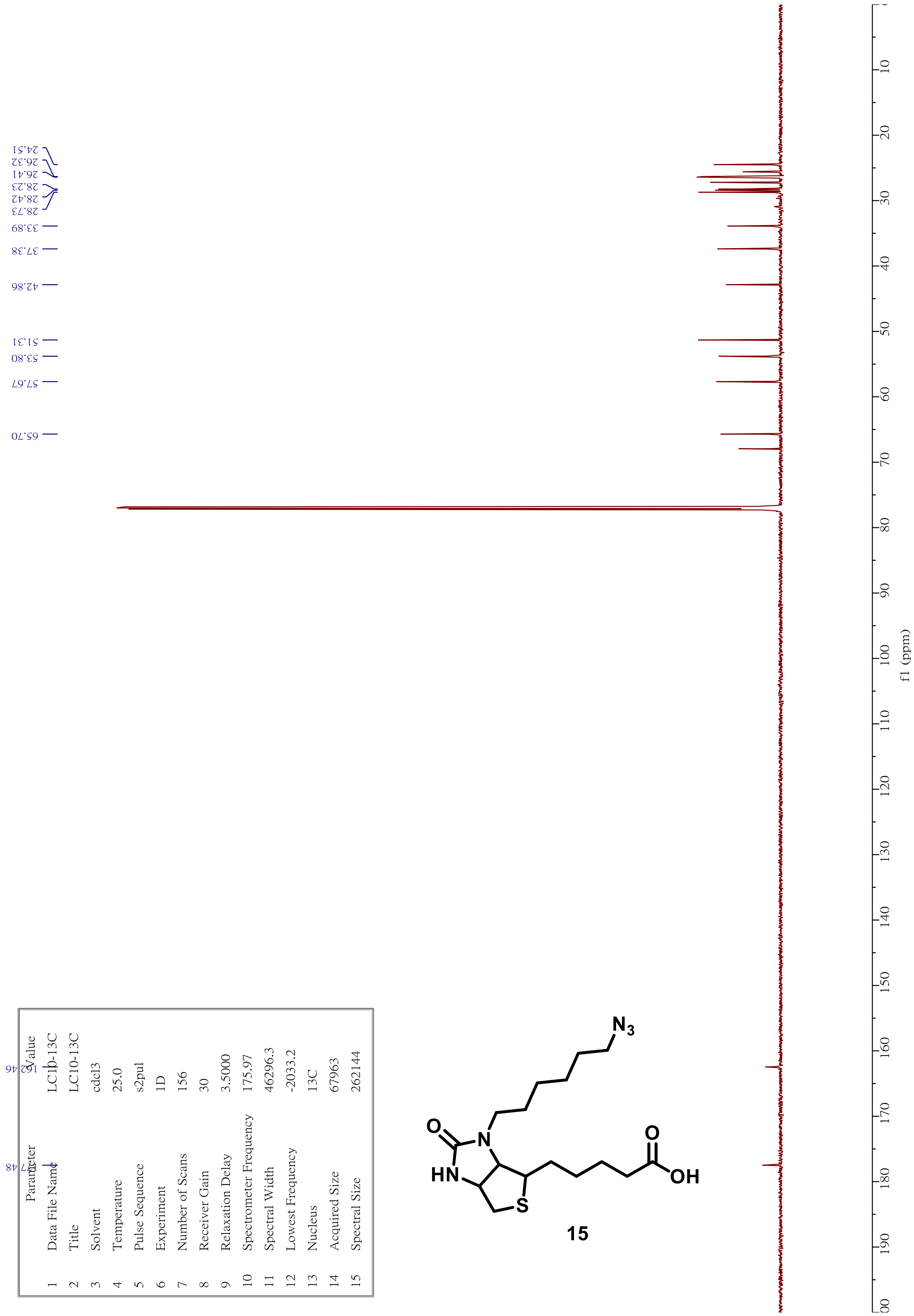

Figure S20. ^{13}C NMR spectra of compound **10**.

Data:LC13
Comment:
Description:
Ionization Mode:ESI+
History:Average(MS[1] 0.45..0.47)

Acquired:10/25/2021 11:23:21 PM
Operator:AccuTOF
m/z Calibration File:20210902-TFANa_...
Created:10/26/2021 2:26:25 PM
Created by:AccuTOF

Charge number:1
Tolerance:300.00[ppm], 250.00 .. 250....
Unsaturation Number:-100.0 .. 200.0 (...
Element:^{12}C:16 .. 16, ^{1}H:27 .. 28, ^{14}N:5 .. 5, ^{23}Na:0 .. 2, ^{16}O:3 .. 3, ^{32}S:1 .. 1

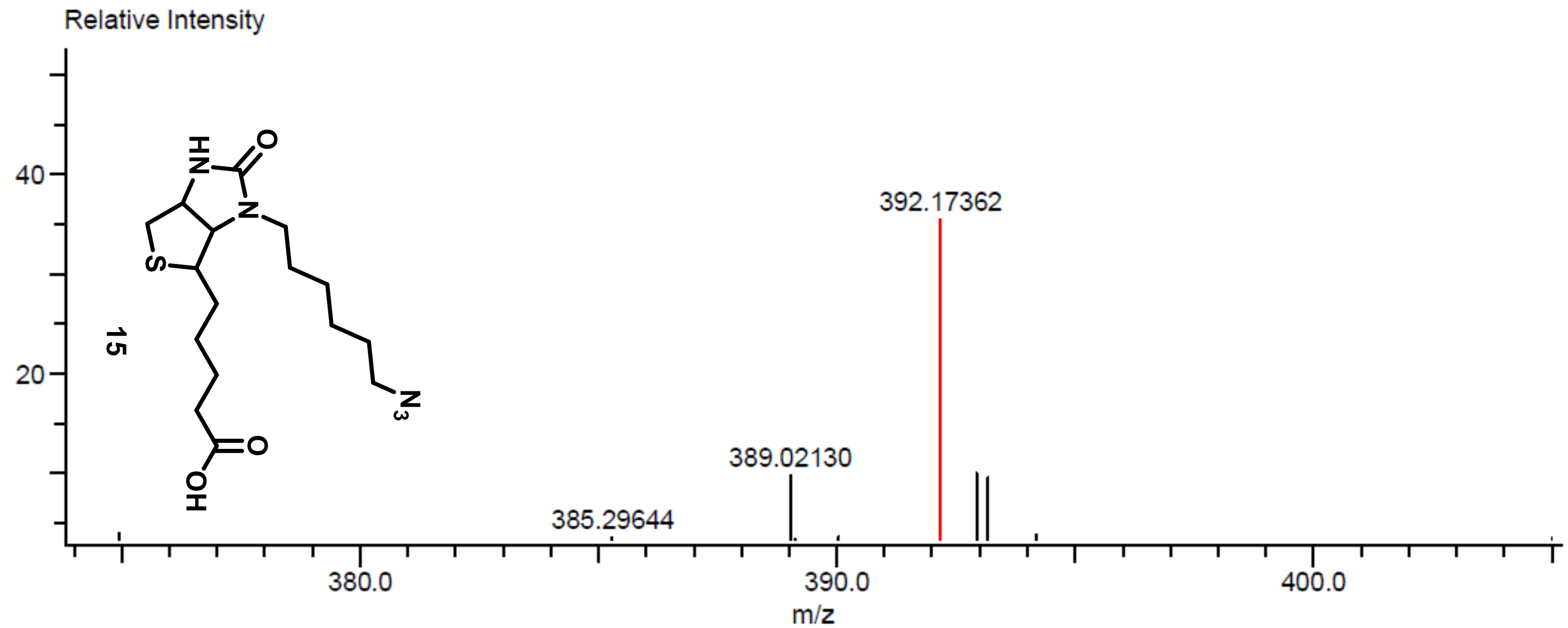

Mass	Intensity	Calc. Mass	Mass Difference [mDa]	Mass Difference [ppm]	Possible Formula
392.17362	11725.50	392.17323	0.39	0.99	$^{12}C_{16}{}^{1}H_{27}{}^{14}N_{5}{}^{23}Na_{1}{}^{16}O_{3}{}^{32}S_{1}$

Figure S21. HRMS-ESI spectra of compound **15**.

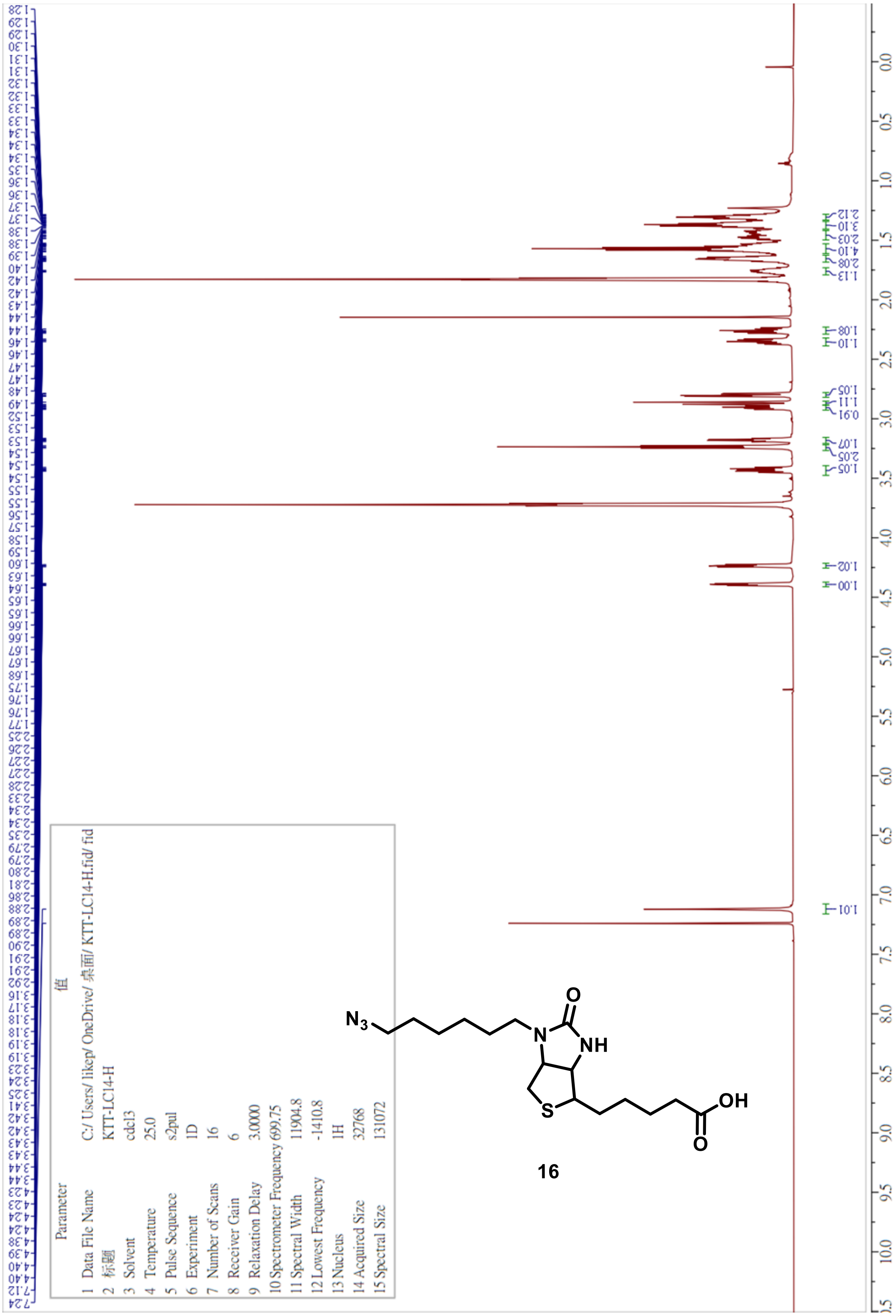

Figure S22. ^{1}H NMR spectra of compound **16**.

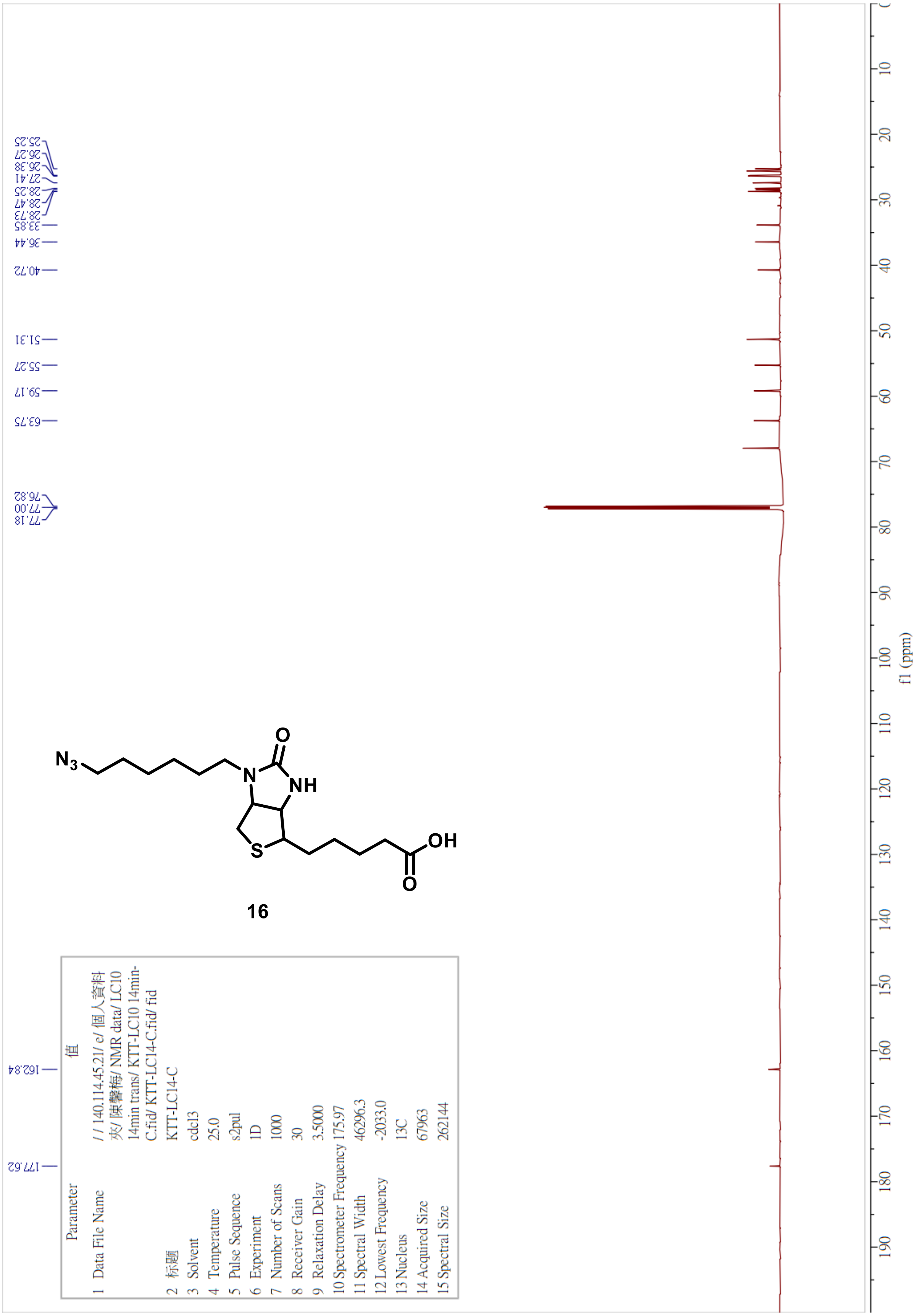

Figure S23. ^{13}C NMR spectra of compound **16.**

Data:LC14
Comment:
Description:
Ionization Mode:ESI+
History:Average(MS[1] 0.45..0.49)

Acquired:10/25/2021 11:27:19 PM
Operator:AccuTOF
m/z Calibration File:20210902-TFANa_...
Created:10/26/2021 2:27:22 PM
Created by:AccuTOF

Charge number:1
Tolerance:300.00[ppm], 250.00 .. 250....
Unsaturation Number:-100.0 .. 200.0 (...
Element:^{12}C:16 .. 16, ^{1}H:27 .. 28, ^{14}N:5 .. 5, ^{23}Na:0 .. 2, ^{16}O:3 .. 3, ^{32}S:1 .. 1

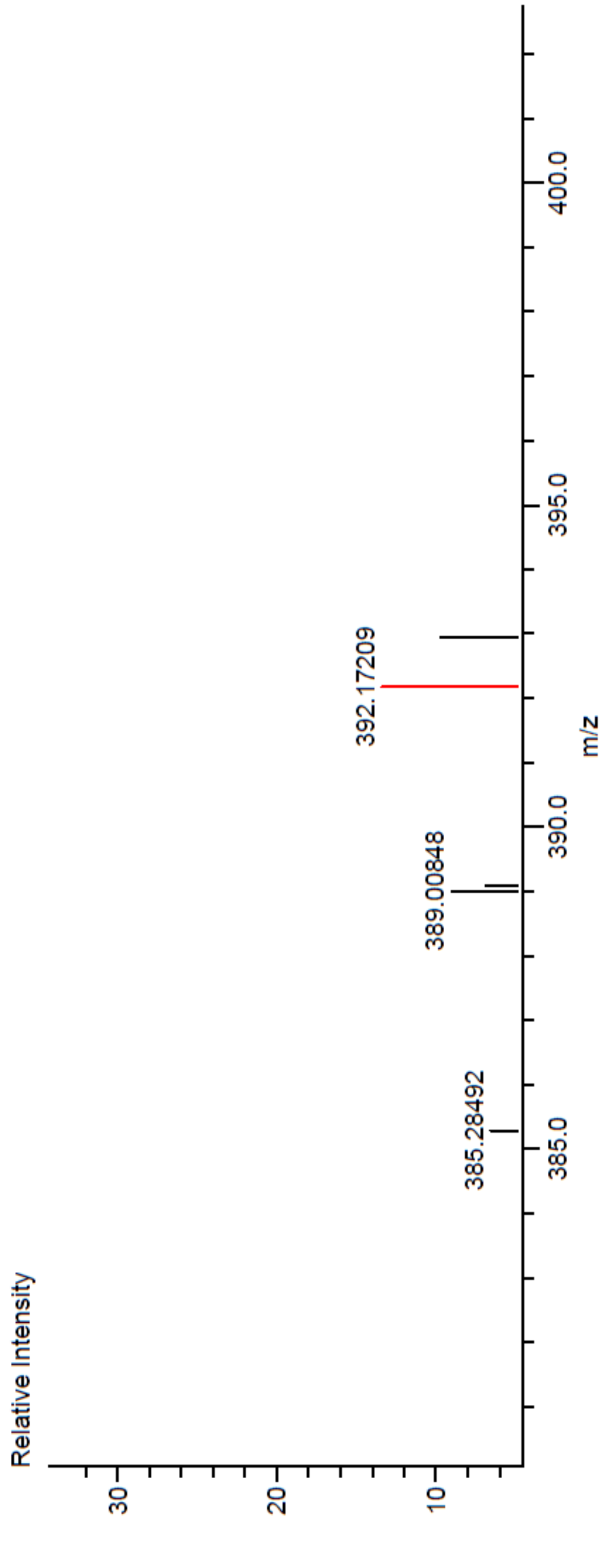

Mass	Intensity	Calc. Mass	Mass Difference [mDa]	Mass Difference [ppm]	Possible Formula
392.17209	1339.25	392.17323	-1.14	-2.92	$^{12}C_{16}{}^{1}H_{27}{}^{14}N_{5}{}^{23}Na_{1}{}^{16}O_{3}{}^{32}S_{1}$

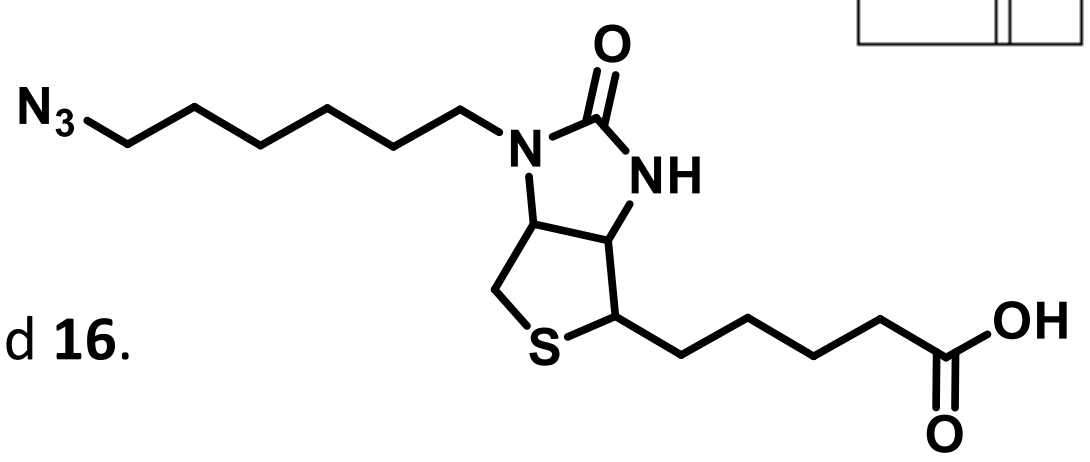

16

Figure S24. HRMS-ESI spectra of compound **16**.

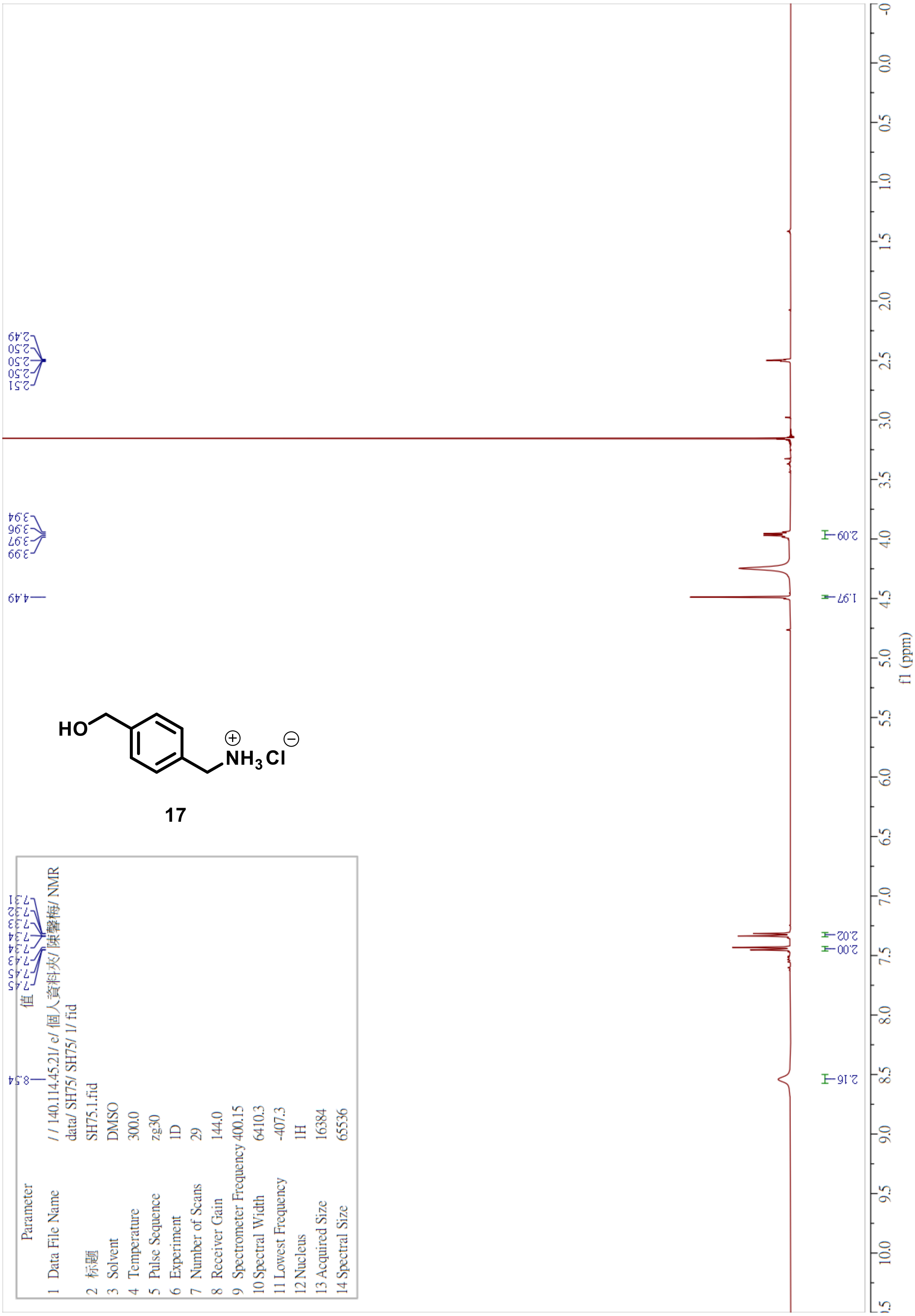

Figure S25. ^{1}H NMR spectra of compound **17**.

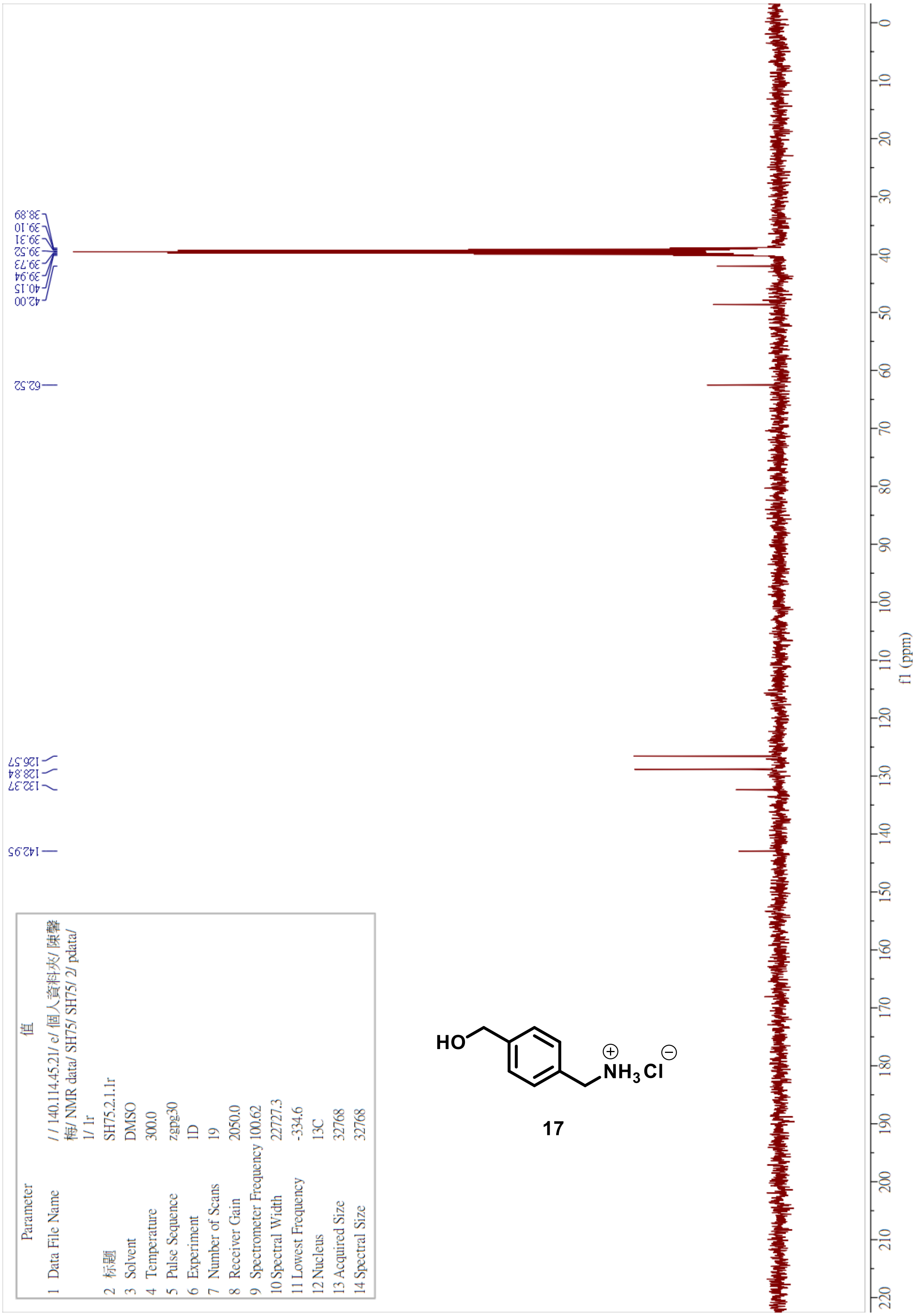

Figure S26. ^{13}C NMR spectra of compound **17**.

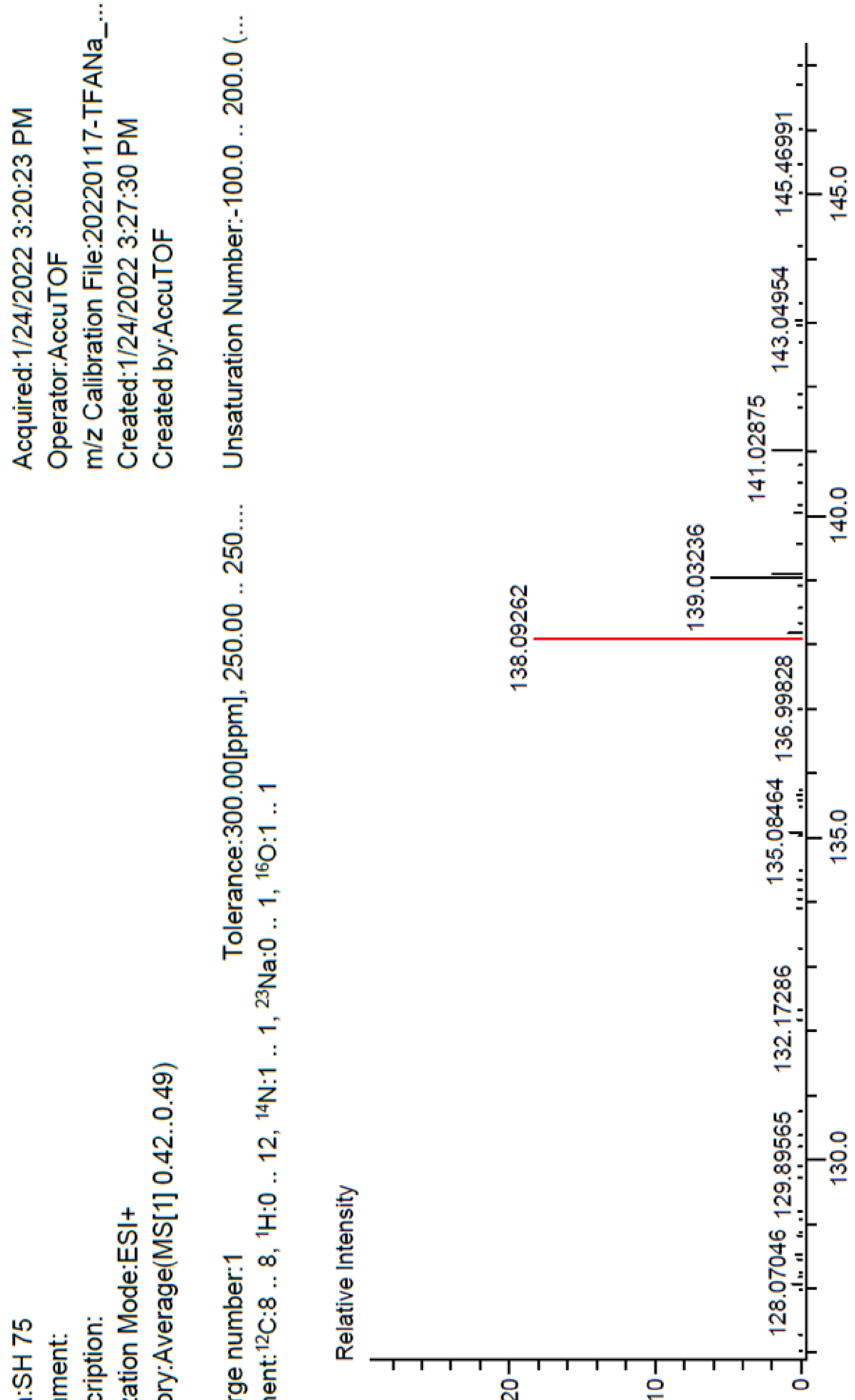

Mass	Intensity	Calc. Mass	Mass Difference [mDa]	Mass Difference [ppm]	Possible Formula
138.09262	8991.12	138.09189	0.73	5.27	$^{12}C_8{}^{1}H_{12}{}^{14}N_1{}^{16}O_1$

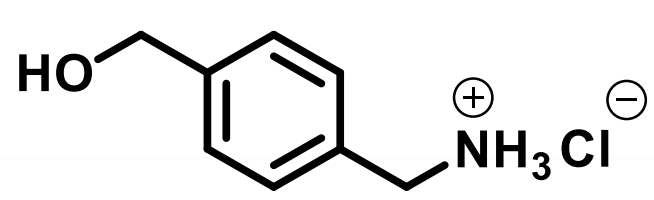

17

Figure S27. HRMS-ESI spectra of compound **17**.

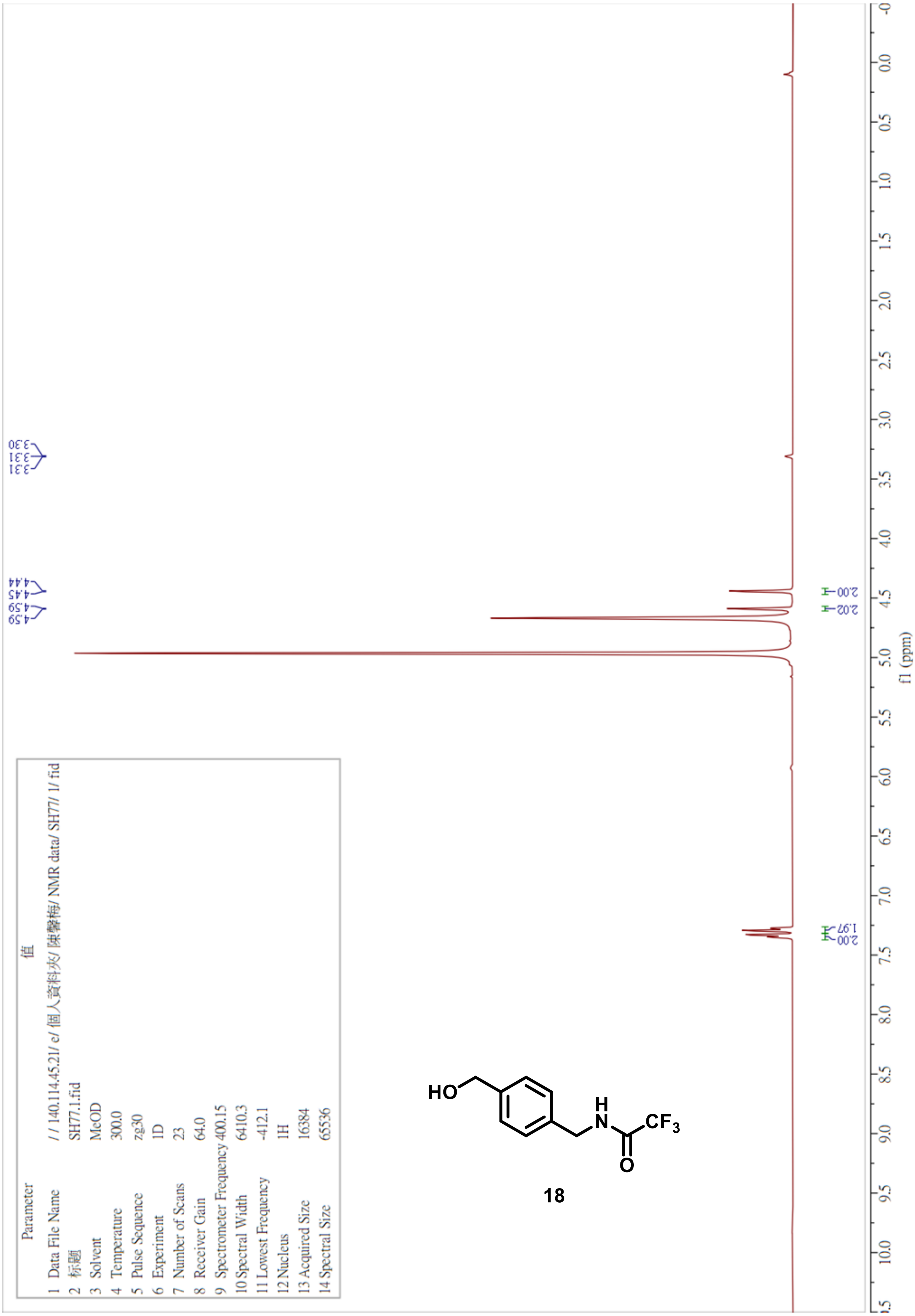

Figure S28. ^{1}H NMR spectra of compound **18**.

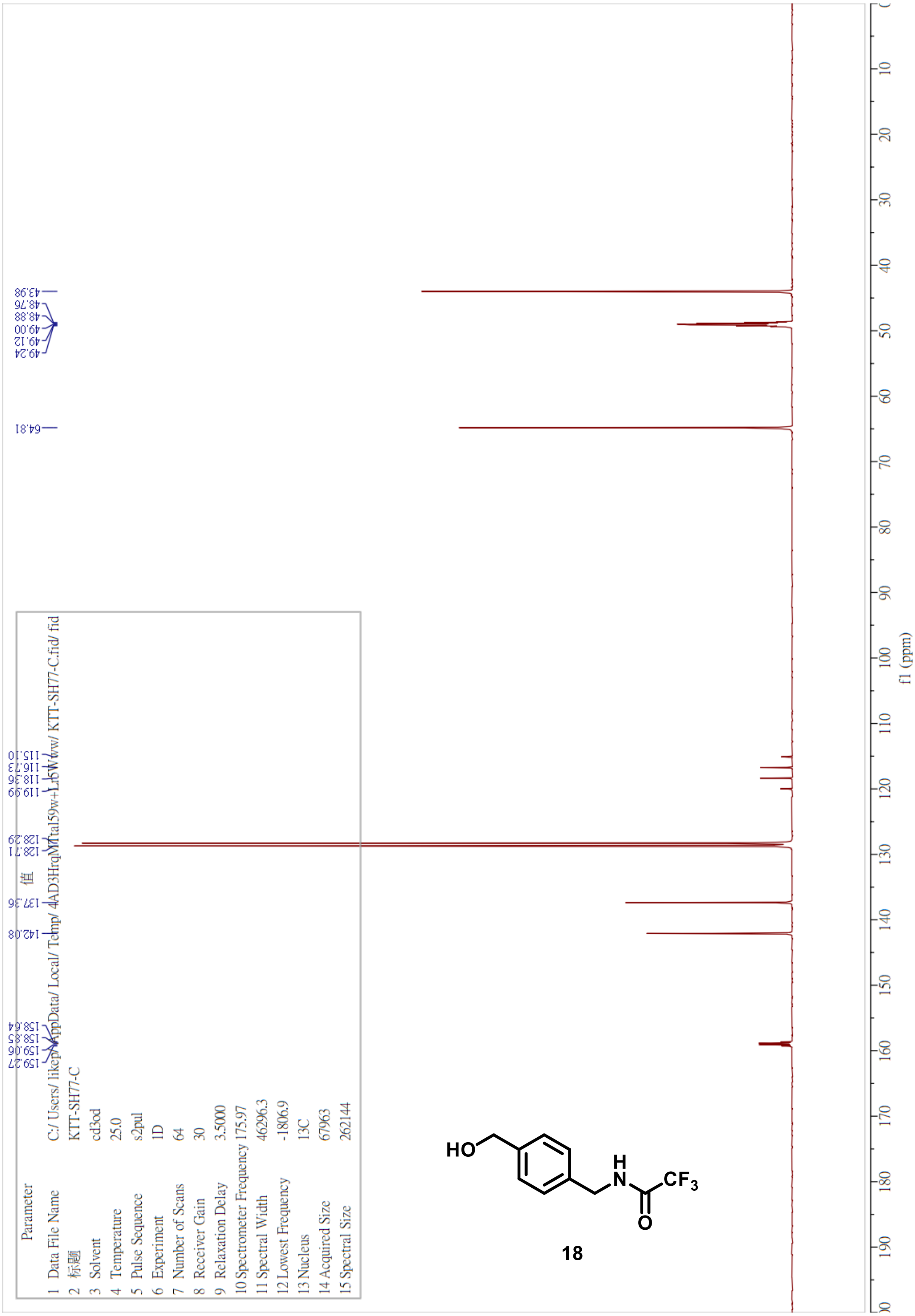

Figure S29. ^{13}C NMR spectra of compound **18**.

Data:SH77
Comment:
Description:
Ionization Mode:ESI-
History:Average(MS[1] 0.21..0.28)

Acquired:1/24/2022 10:35:16 AM
Operator:AccuTOF
m/z Calibration File:2021201-TFANa_...
Created:1/24/2022 3:17:49 PM
Created by:AccuTOF

Charge number:1
Tolerance:300.00[ppm], 250.00 .. 250....
Unsaturation Number:-100.0 .. 200.0 (...
Element:^{12}C:10 .. 10, ^{1}H:0 .. 10, ^{19}F:3 .. 3, ^{14}N:1 .. 1, ^{23}Na:0 .. 1, ^{16}O:2 .. 2

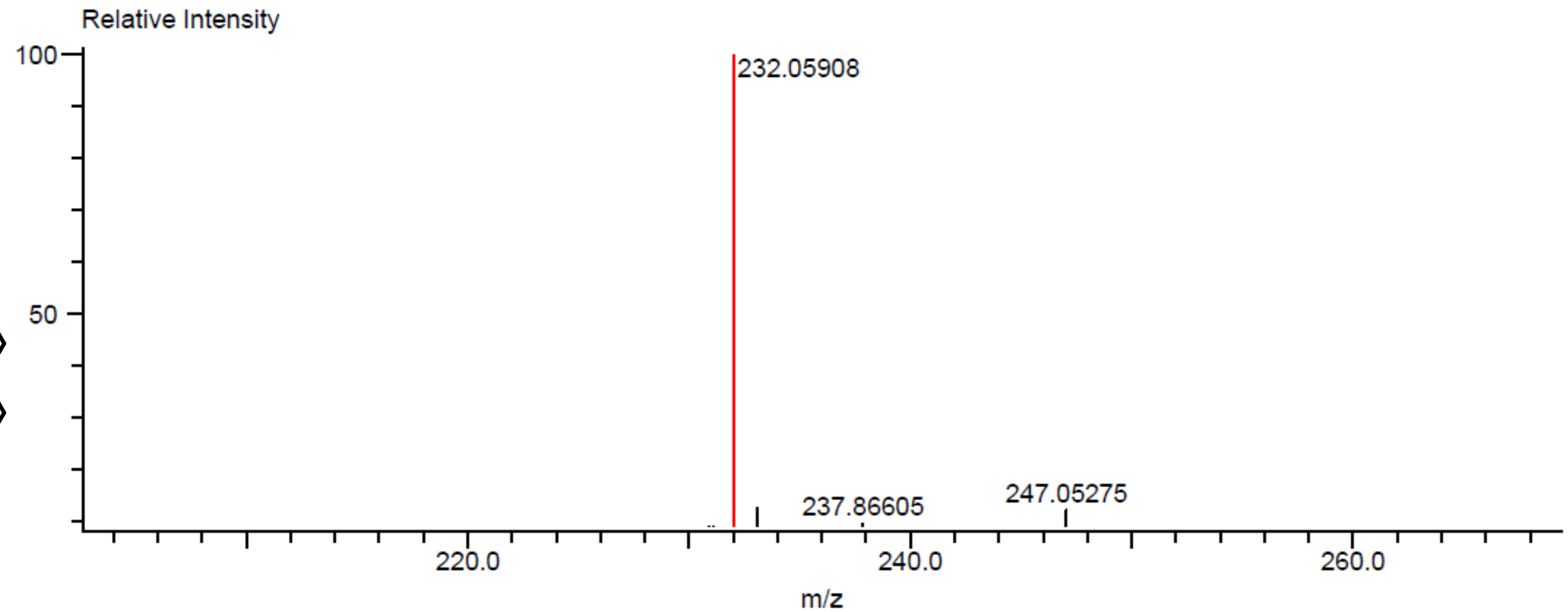

Mass	Intensity	Calc. Mass	Mass Difference [mDa]	Mass Difference [ppm]	Possible Formula
232.05908	4336.04	232.05854	0.54	2.32	$^{12}C_{10}{}^{1}H_{9}{}^{19}F_{3}{}^{14}N_{1}{}^{16}O_{2}$

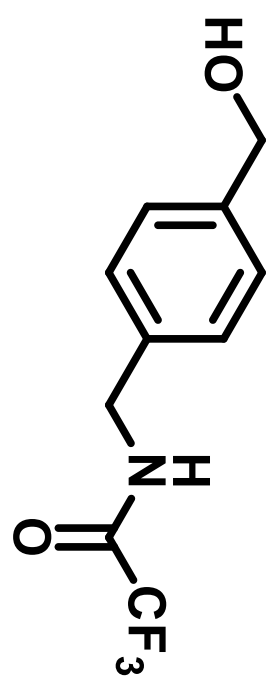

18

Figure S30. HRMS-ESI spectra of compound **18**.

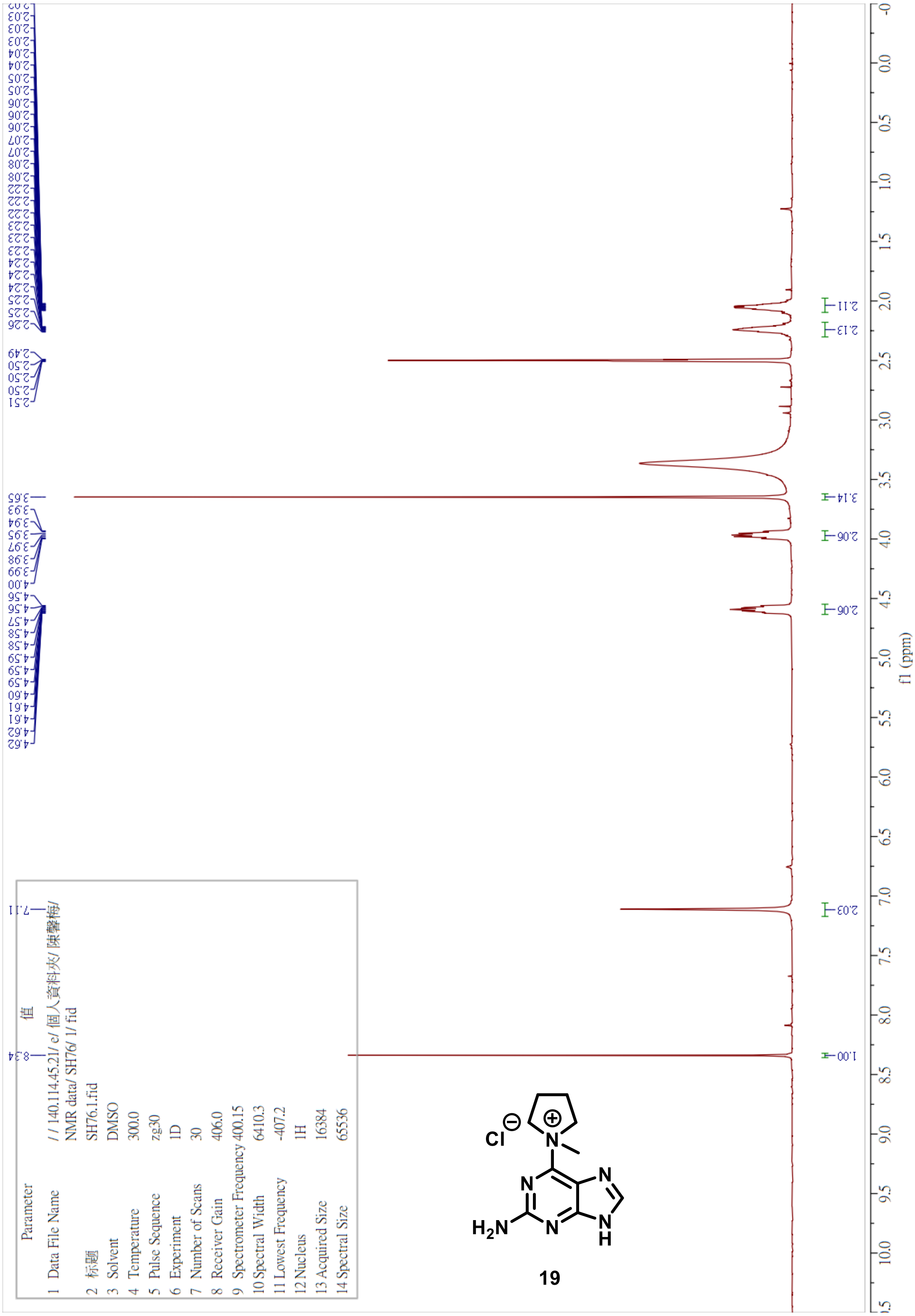

Figure S31. ^{1}H NMR spectra of compound **19**.

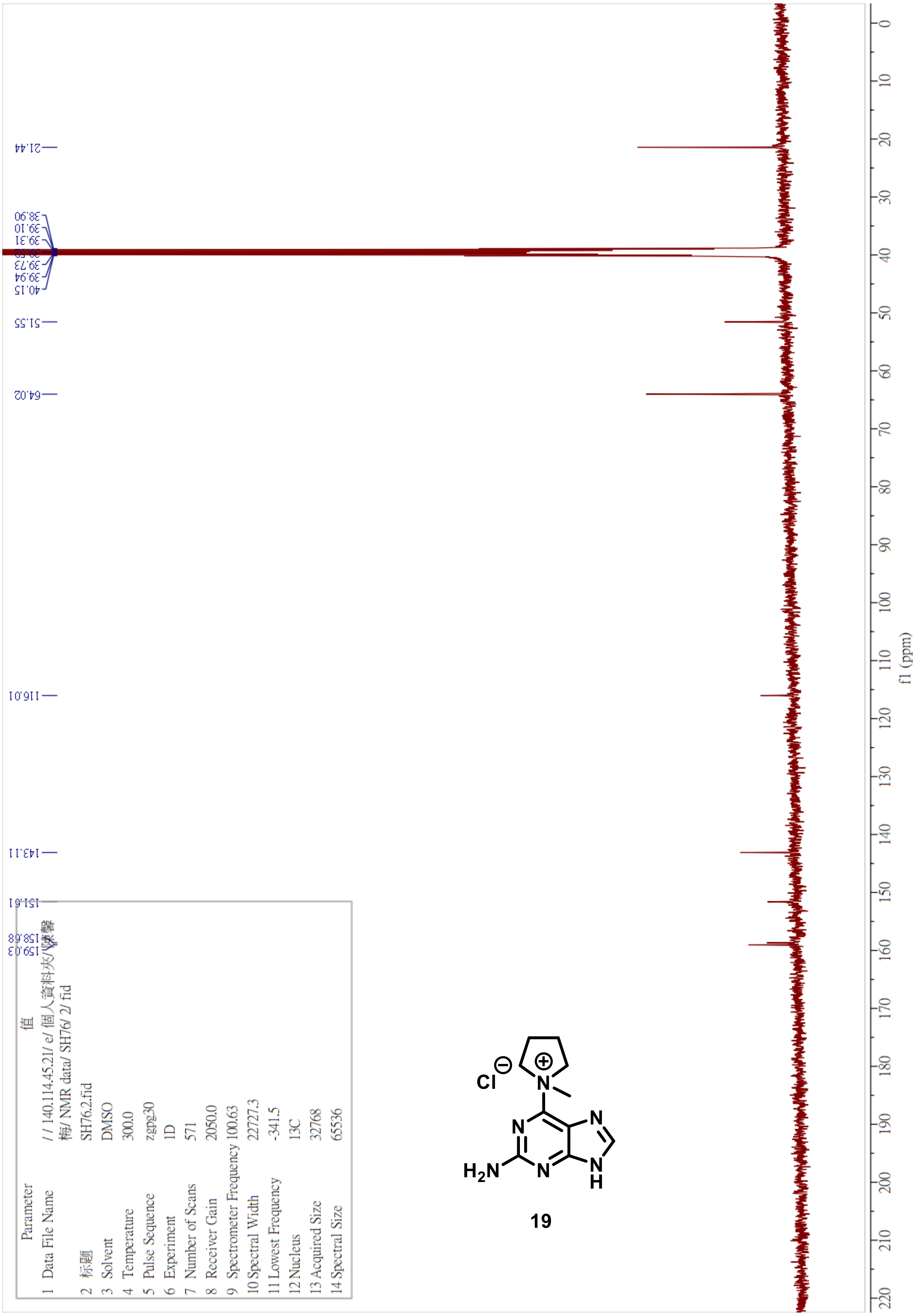

Figure S32. ^{13}C NMR spectra of compound **19**.

Data:SH76
Comment:
Description:
Ionization Mode:ESI-
History:Average(MS[1] 0.34..0.36)

Acquired:1/24/2022 10:31:39 AM
Operator:AccuTOF
m/z Calibration File:2021201-TFANa_...
Created:1/24/2022 3:16:20 PM
Created by:AccuTOF

Charge number:1
Tolerance:300.00[ppm], 250.00 .. 250....
Unsaturation Number:-100.0 .. 200.0 (...
Element:^{12}C:10 .. 10, ^{1}H:0 .. 15, ^{35}Cl:1 .. 1, ^{14}N:6 .. 6, ^{23}Na:0 .. 1

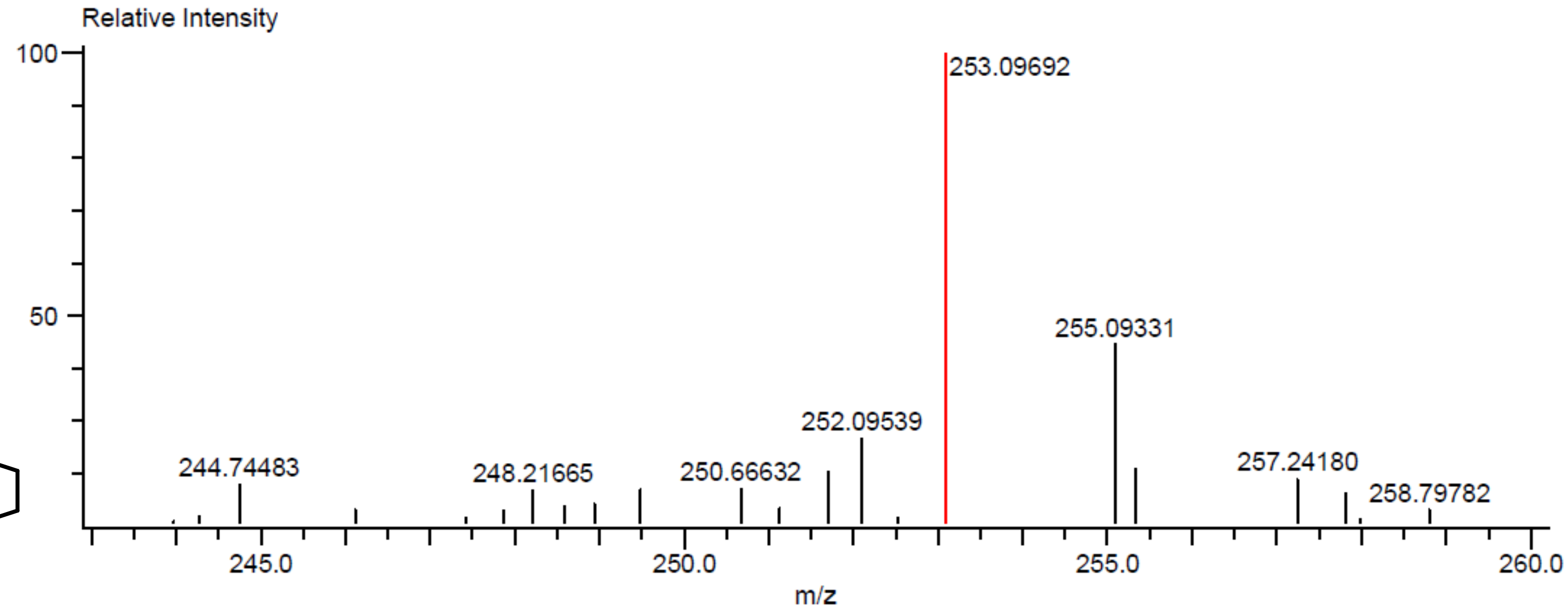

Mass	Intensity	Calc. Mass	Mass Difference [mDa]	Mass Difference [ppm]	Possible Formula
253.09692	2671.75	253.09685	0.07	0.28	$^{12}C_{10}{}^{1}H_{14}{}^{35}Cl_{1}{}^{14}N_{6}$

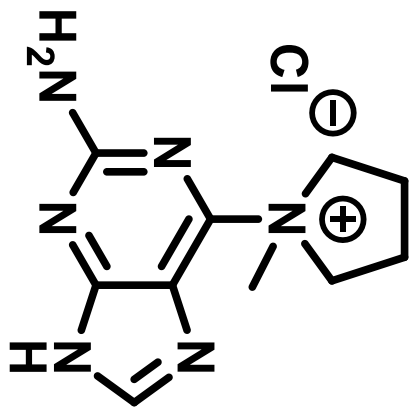

19

Figure S33. HRMS-ESI spectra of compound **19**.

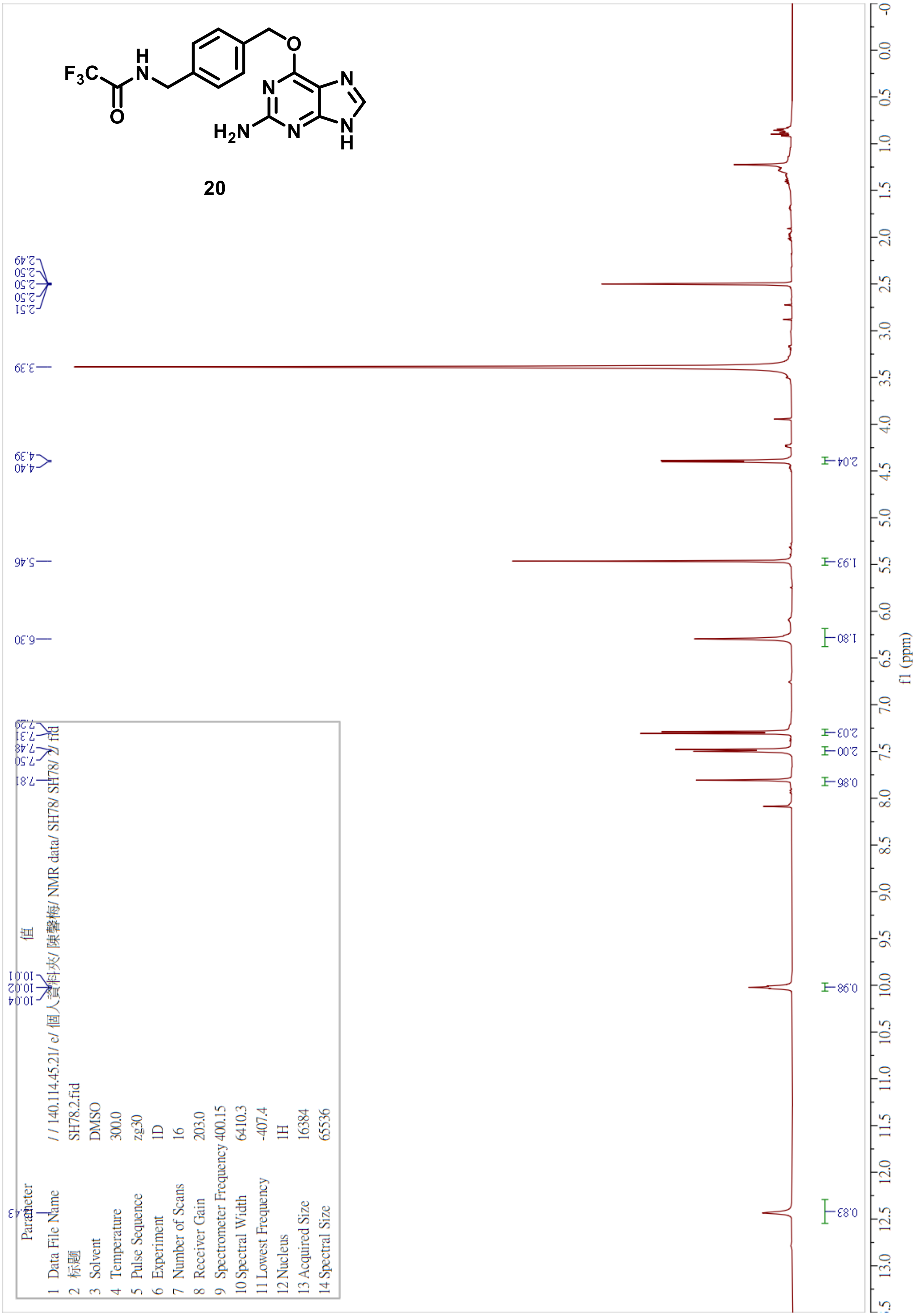

Figure S34. ^{1}H NMR spectra of compound **20**.

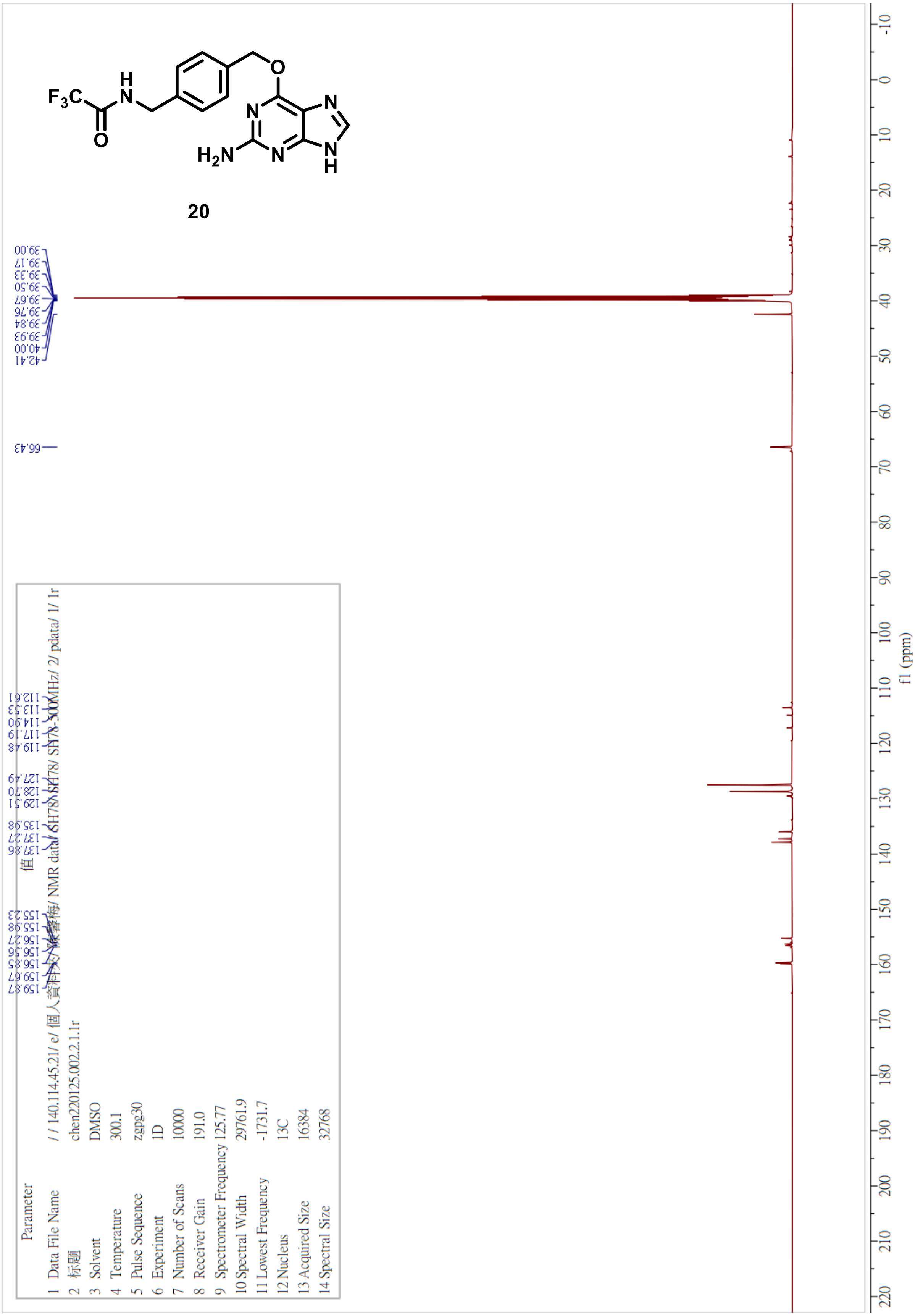

Figure S35. ^{13}C NMR spectra of compound **20**.

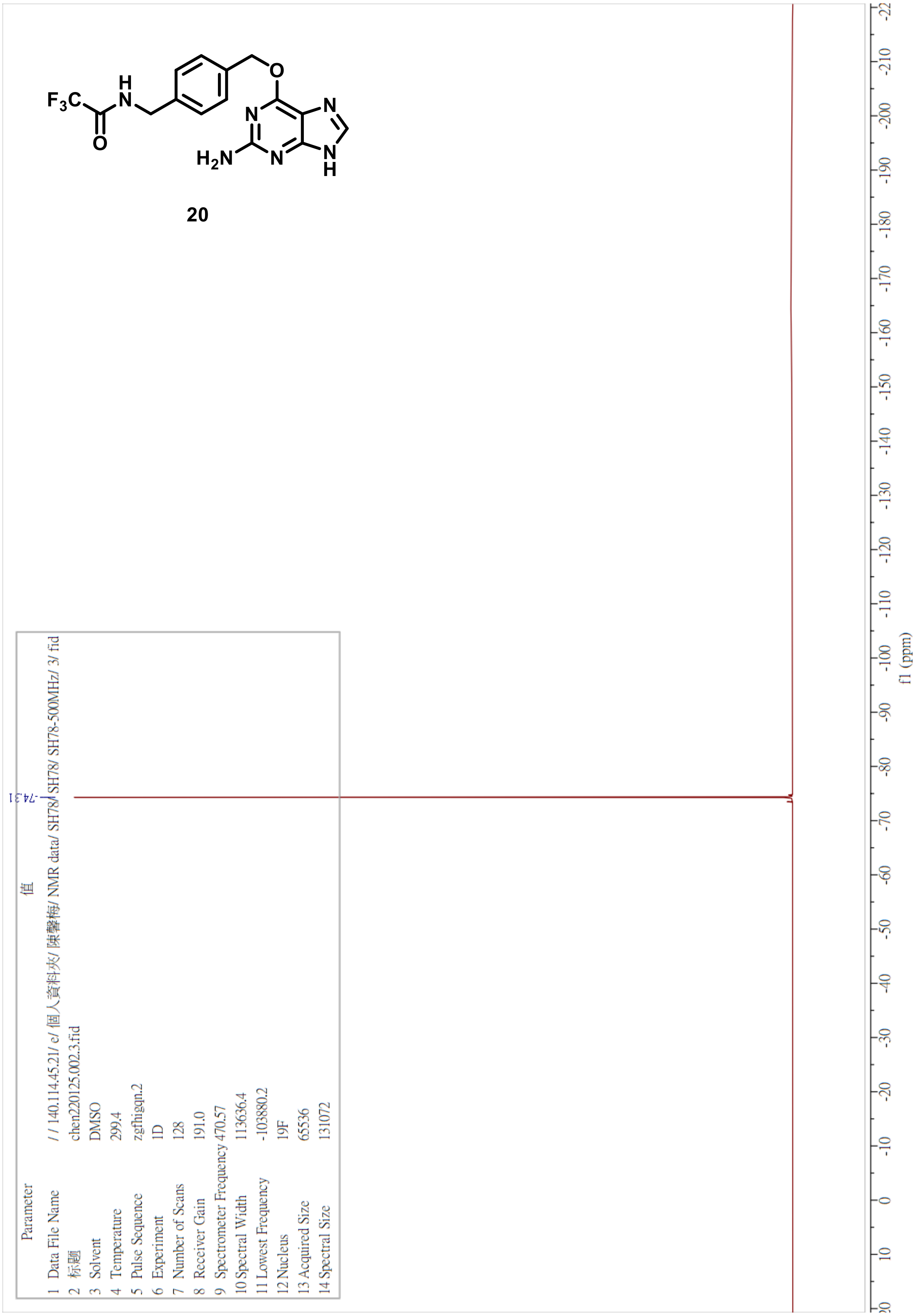

Figure S36. ^{19}F NMR spectra of compound **20**.

Data:SH78
Comment:
Description:
Ionization Mode:ESI+
History:Average(MS[1] 0.45..0.48)

Acquired:2/8/2022 10:34:53 AM
Operator:AccuTOF
m/z Calibration File:202200208-TFANa...
Created:2/8/2022 10:58:54 AM
Created by:AccuTOF

Charge number:1
Tolerance:300.00[ppm], 250.00 .. 250....
Unsaturation Number:-100.0 .. 200.0 (...
Element:^{12}C:15 .. 15, ^{1}H:0 .. 14, ^{19}F:3 .. 3, ^{14}N:6 .. 6, ^{23}Na:0 .. 1, ^{16}O:2 .. 2

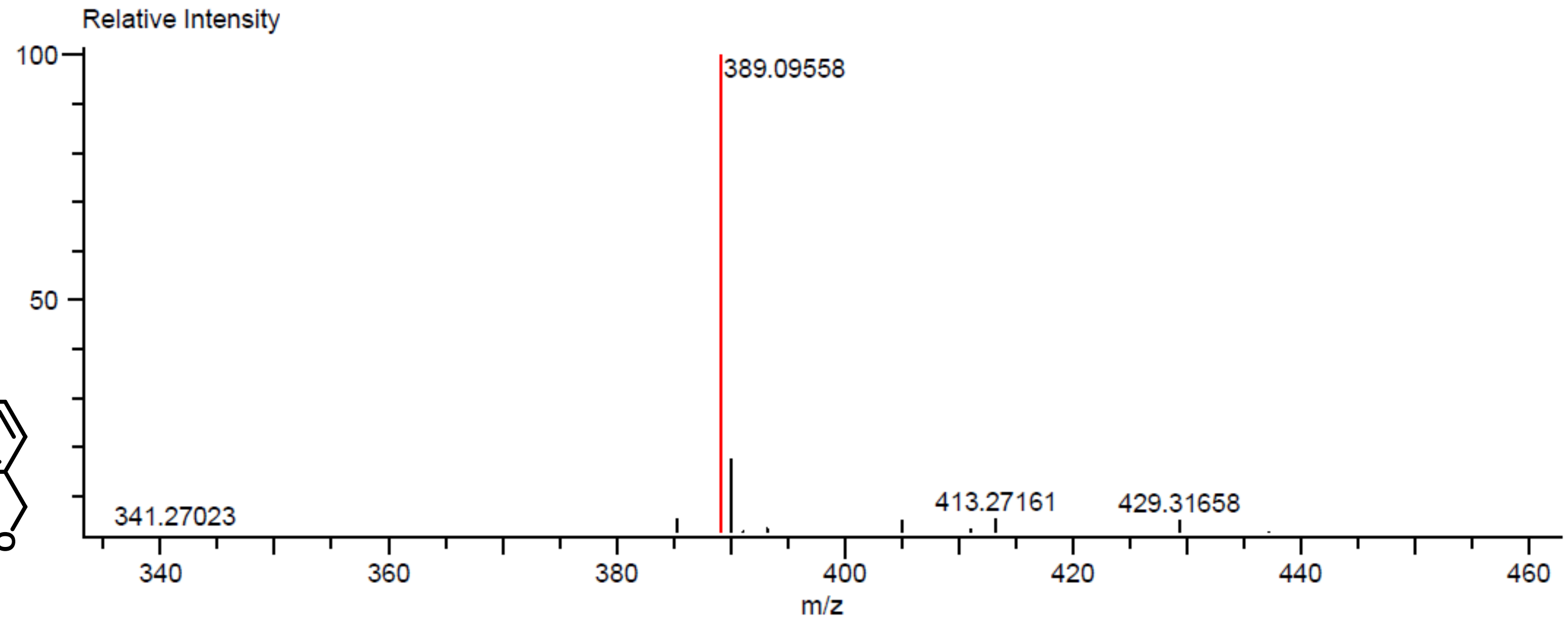

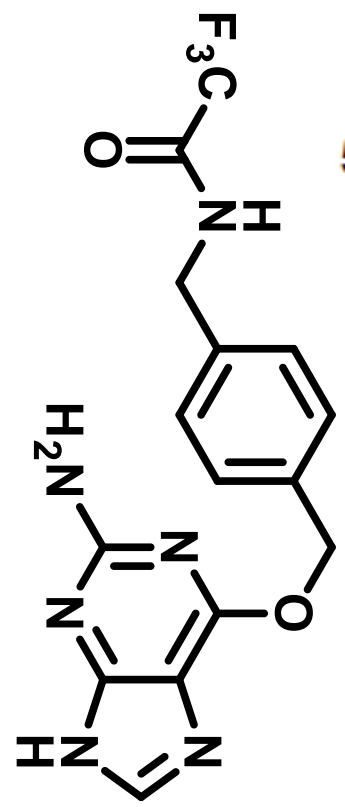

20

Mass	Intensity	Calc. Mass	Mass Difference [mDa]	Mass Difference [ppm]	Possible Formula
389.09558	46233.01	389.09498	0.61	1.56	$^{12}C_{15}{}^{1}H_{13}{}^{19}F_{3}{}^{14}N_{6}{}^{23}Na_{1}{}^{16}O_{2}$

Figure S37. HRMS-ESI spectra of compound **20**.

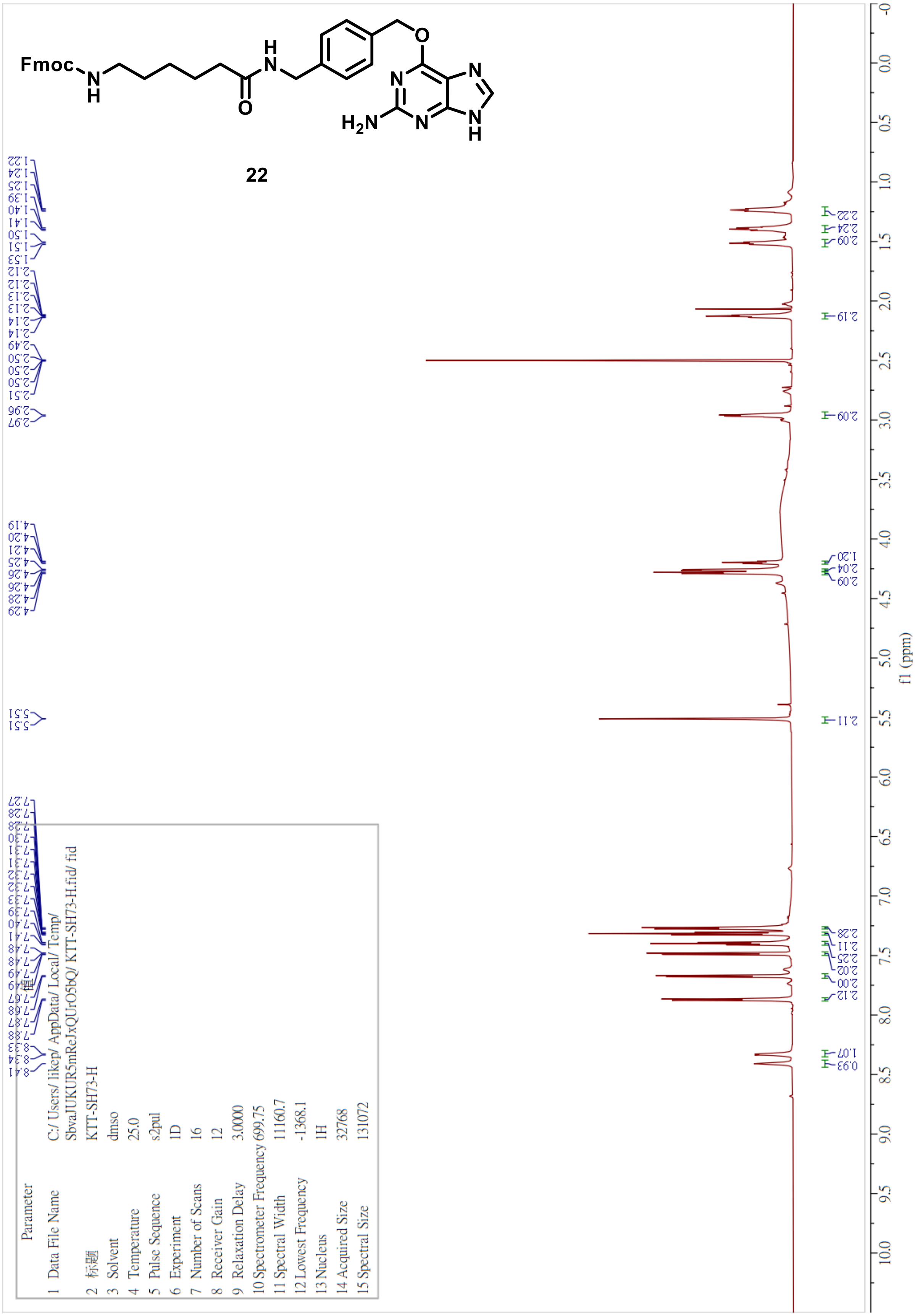

Figure S38. ^{1}H NMR spectra of compound **22**.

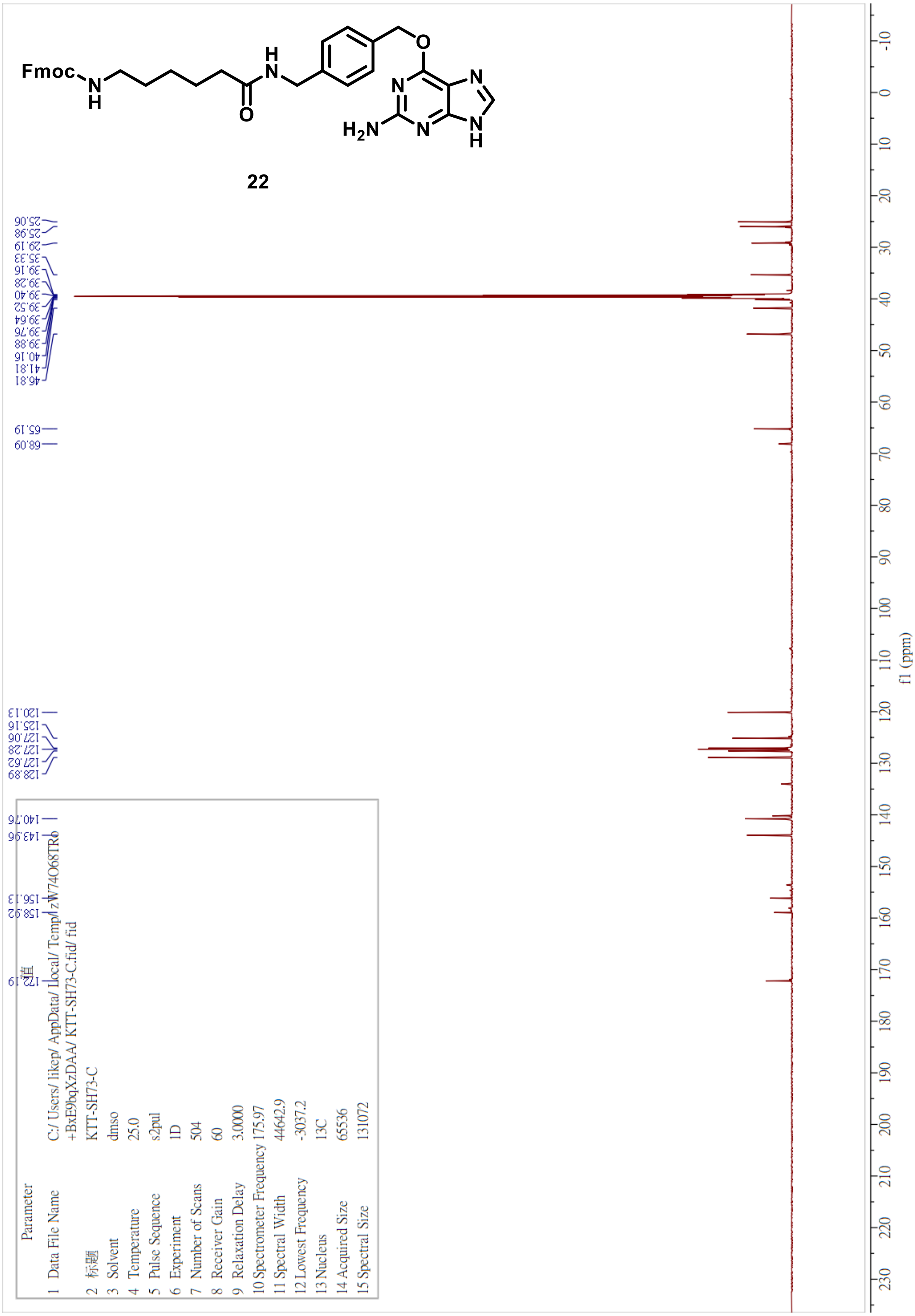

Figure S39. ^{13}C NMR spectra of compound **22**.

Data:SH 73
Comment:
Description:
Ionization Mode:ESI+
History:Average(MS[1] 0.23..0.26)

Acquired:1/24/2022 3:16:46 PM
Operator:AccuTOF
m/z Calibration File:20220117-TFANa_...
Created:1/24/2022 3:29:08 PM
Created by:AccuTOF

Charge number:1
Tolerance:300.00[ppm], 250.00 .. 250....
Unsaturation Number:-100.0 .. 200.0 (...
Element:^{12}C:34 .. 34, ^{1}H:0 .. 36, ^{14}N:7 .. 7, ^{23}Na:0 .. 1, ^{16}O:4 .. 4

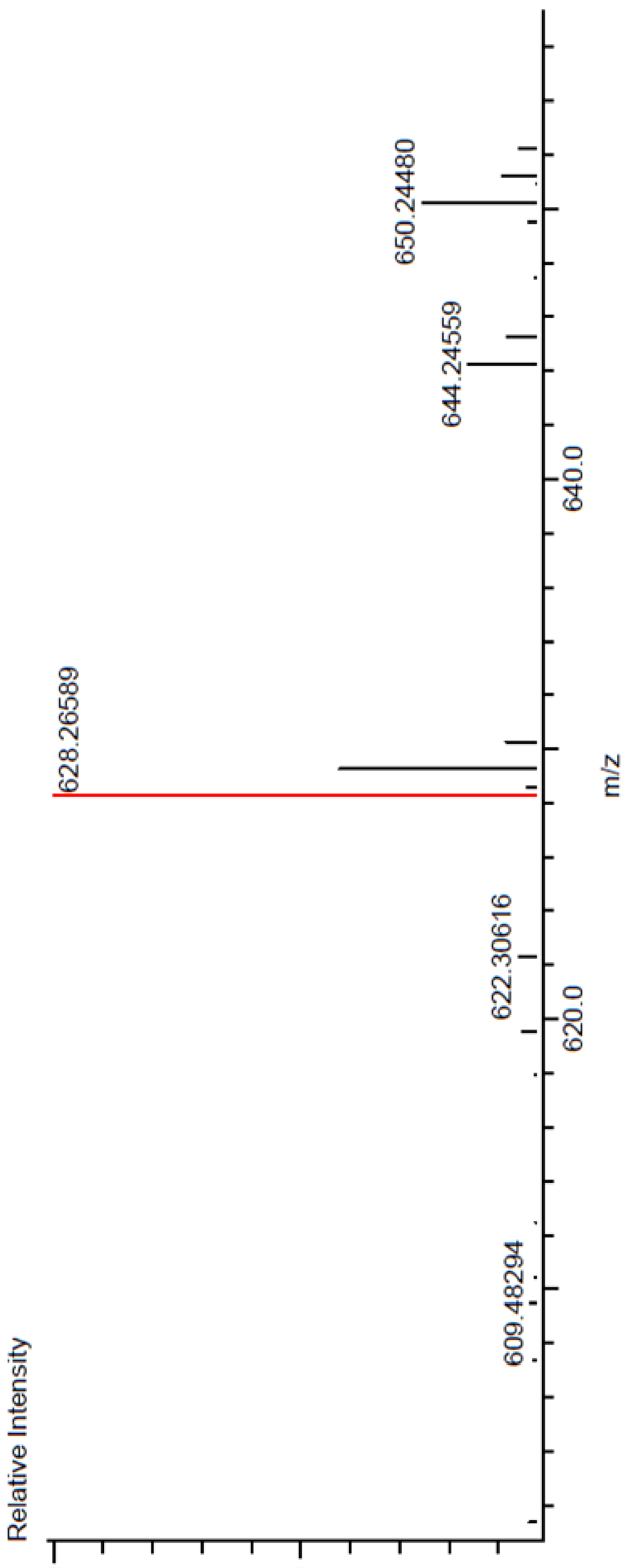

Mass	Intensity	Calc. Mass	Mass Difference [mDa]	Mass Difference [ppm]	Possible Formula
628.26589	9846.33	628.26482	1.07	1.70	$^{12}C_{34}{}^{1}H_{35}{}^{14}N_{7}{}^{23}Na_{1}{}^{16}O_{4}$

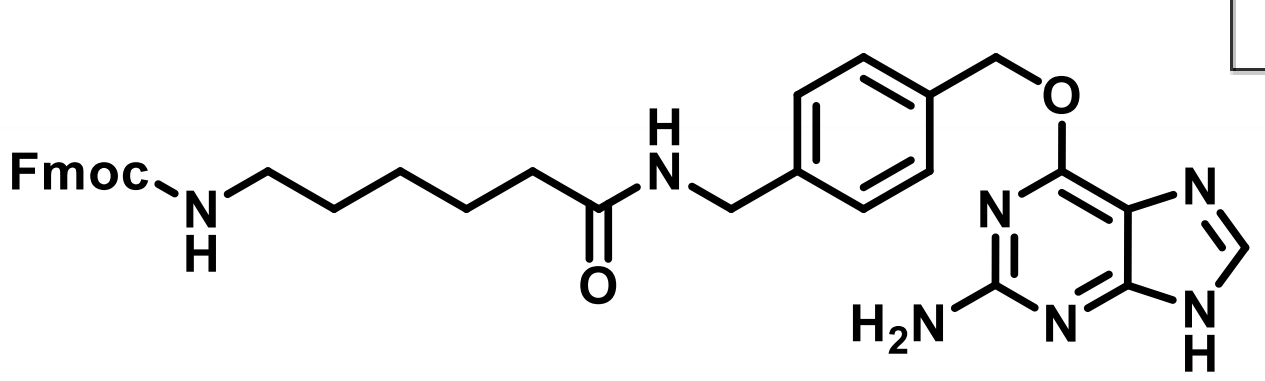

22

Figure S40. HRMS-ESI spectra of compound **22**.

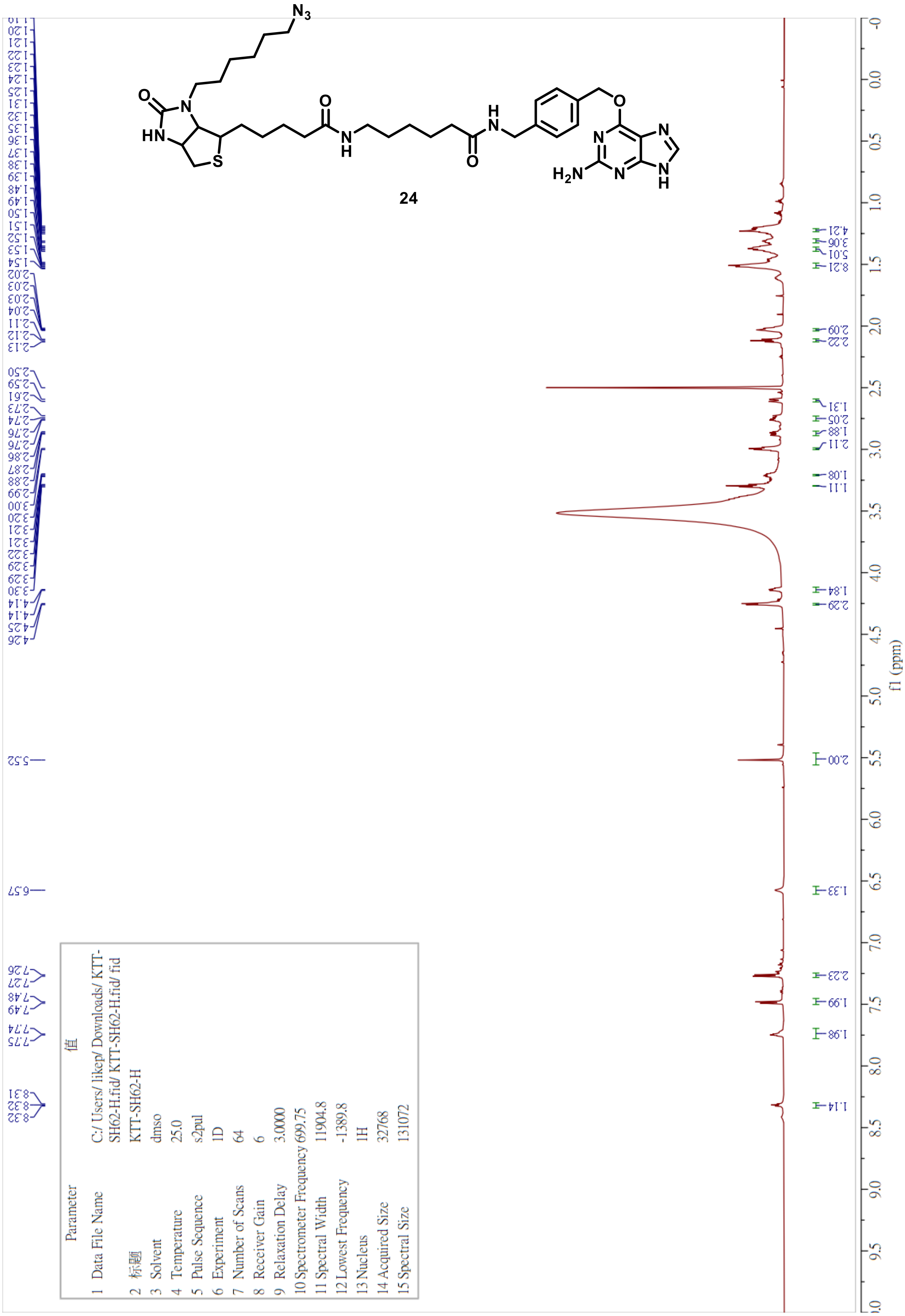

Figure S41. ^{1}H NMR spectra of compound **24**.

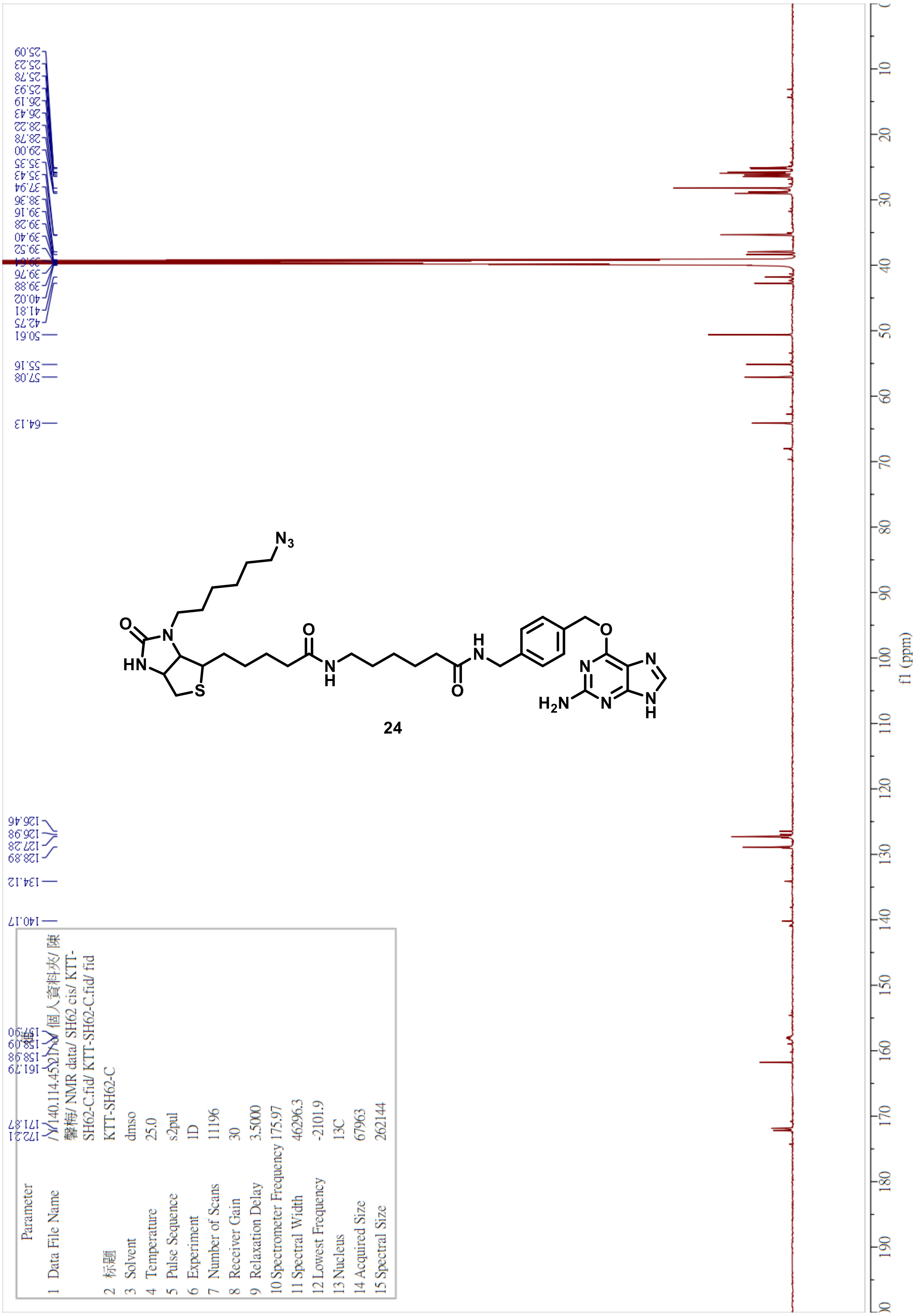

Figure S42. ^{13}C NMR spectra of compound **24**.

Data:SH62
Comment:
Description:
Ionization Mode:ESI+
History:Average(MS[1] 0.30..0.33)

Acquired:11/8/2021 5:13:50 PM
Operator:AccuTOF
m/z Calibration File:20211026-TFANa_...
Created:11/8/2021 5:23:57 PM
Created by:AccuTOF

Charge number:1
Tolerance:300.00[ppm], 250.00 .. 250....
Unsaturation Number:-100.0 .. 200.0 (...
Element:^{12}C:35 .. 35, ^{1}H:50 .. 51, ^{14}N:12 .. 12, ^{23}Na:0 .. 2, ^{16}O:4 .. 4, ^{32}S:1 .. 1

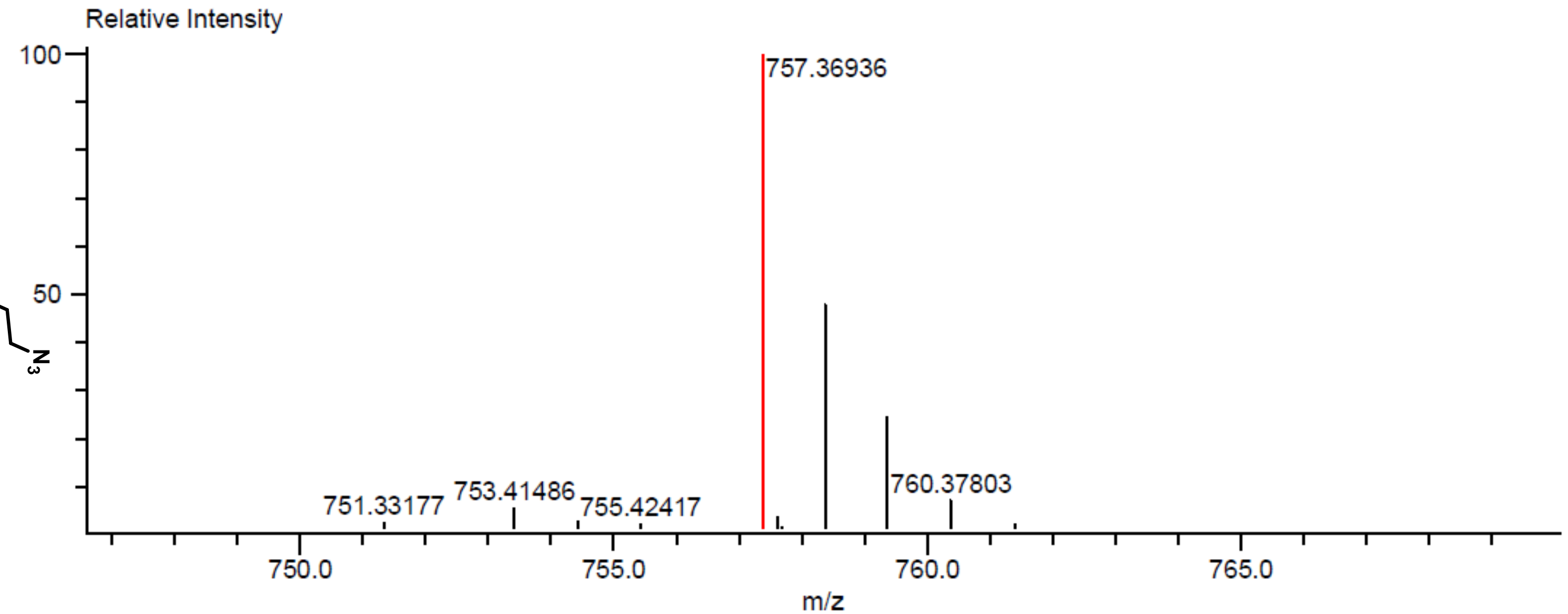

Mass	Intensity	Calc. Mass	Mass Difference [mDa]	Mass Difference [ppm]	Possible Formula
757.36936	13018.88	757.36964	-0.28	-0.36	$^{12}C_{35}{}^{1}H_{50}{}^{14}N_{12}{}^{23}Na_{1}{}^{16}O_{4}{}^{32}S_{1}$

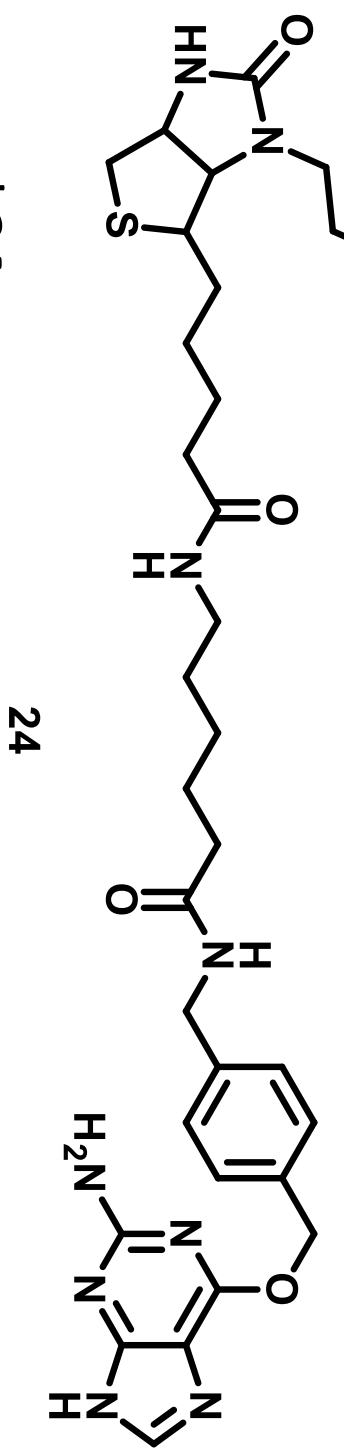

24

Figure S43. HRMS-ESI spectra of compound **24**.

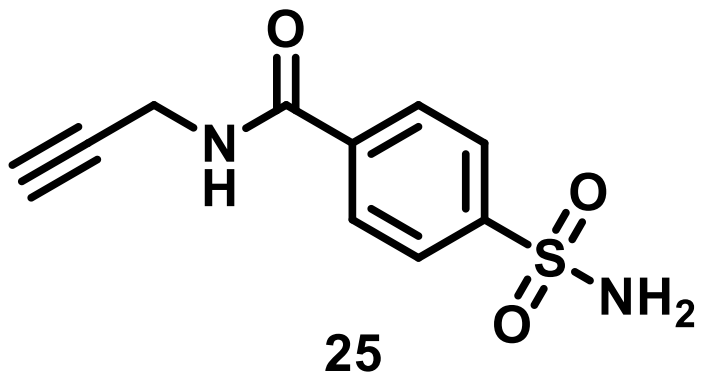

25

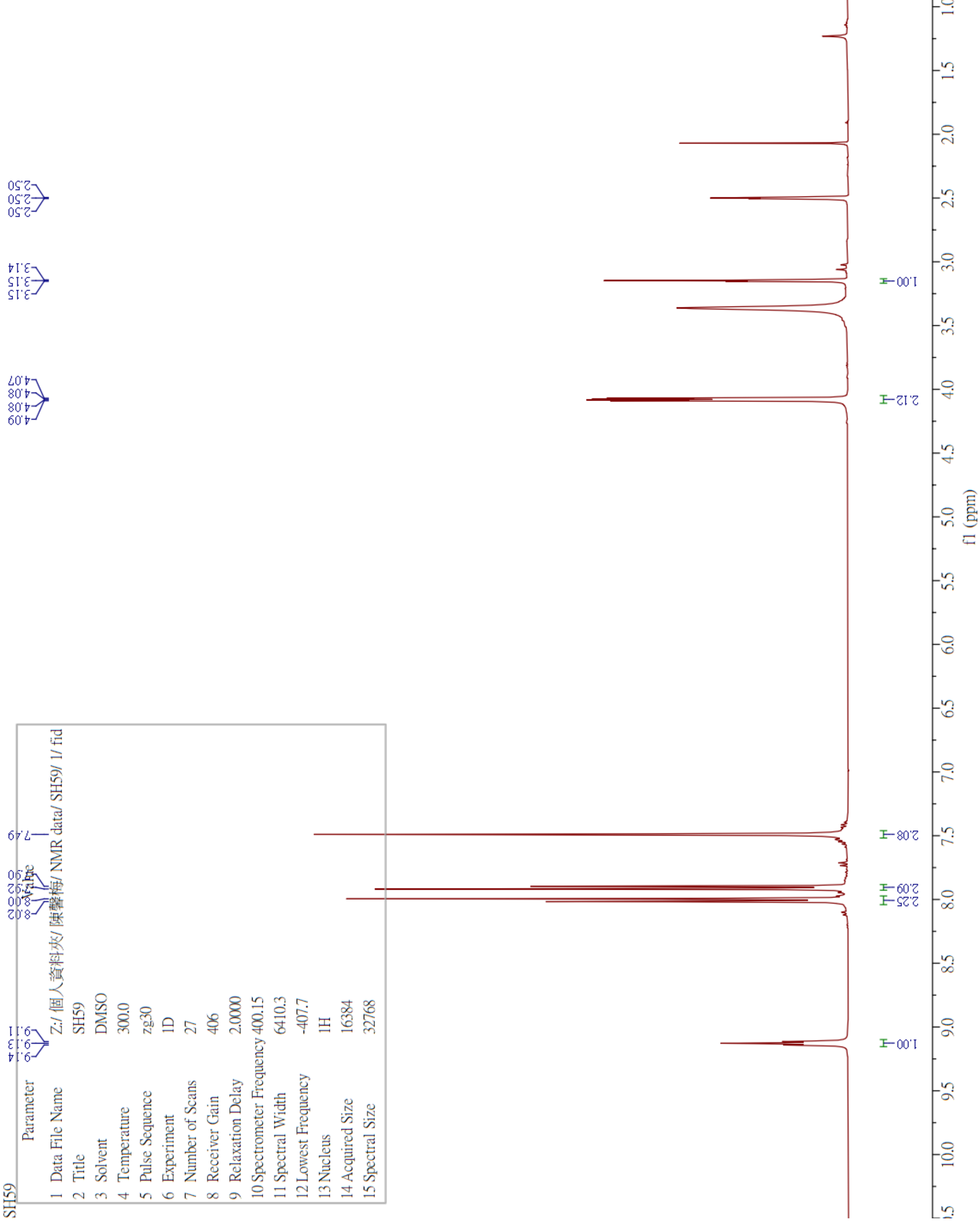

Figure S44. ^{1}H NMR spectra of compound **25**.

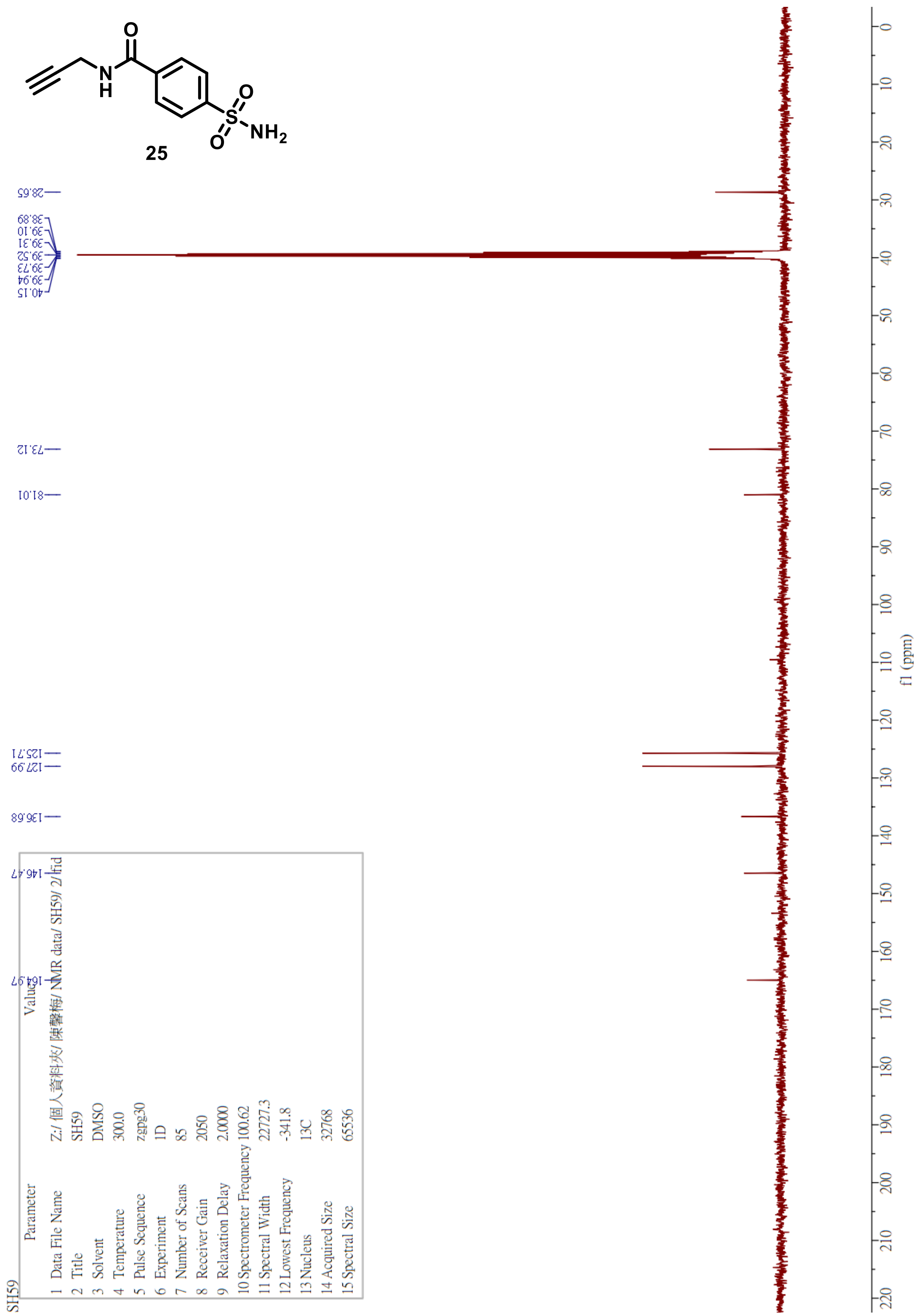

Figure S45. ^{13}C NMR spectra of compound **25**.

Data:SH59
Comment:
Description:
Ionization Mode:ESI-
History:Average(MS[1] 0.29)

Acquired:1/24/2022 10:38:53 AM
Operator:AccuTOF
m/z Calibration File:2021201-TFANa_...
Created:1/24/2022 3:20:14 PM
Created by:AccuTOF

Charge number:1
Tolerance:300.00[ppm], 250.00 .. 250....
Unsaturation Number:-100.0 .. 200.0 (...
Element:^{12}C:10 .. 10, ^{1}H:0 .. 10, ^{14}N:2 .. 2, ^{23}Na:0 .. 1, ^{16}O:3 .. 3, ^{32}S:1 .. 1

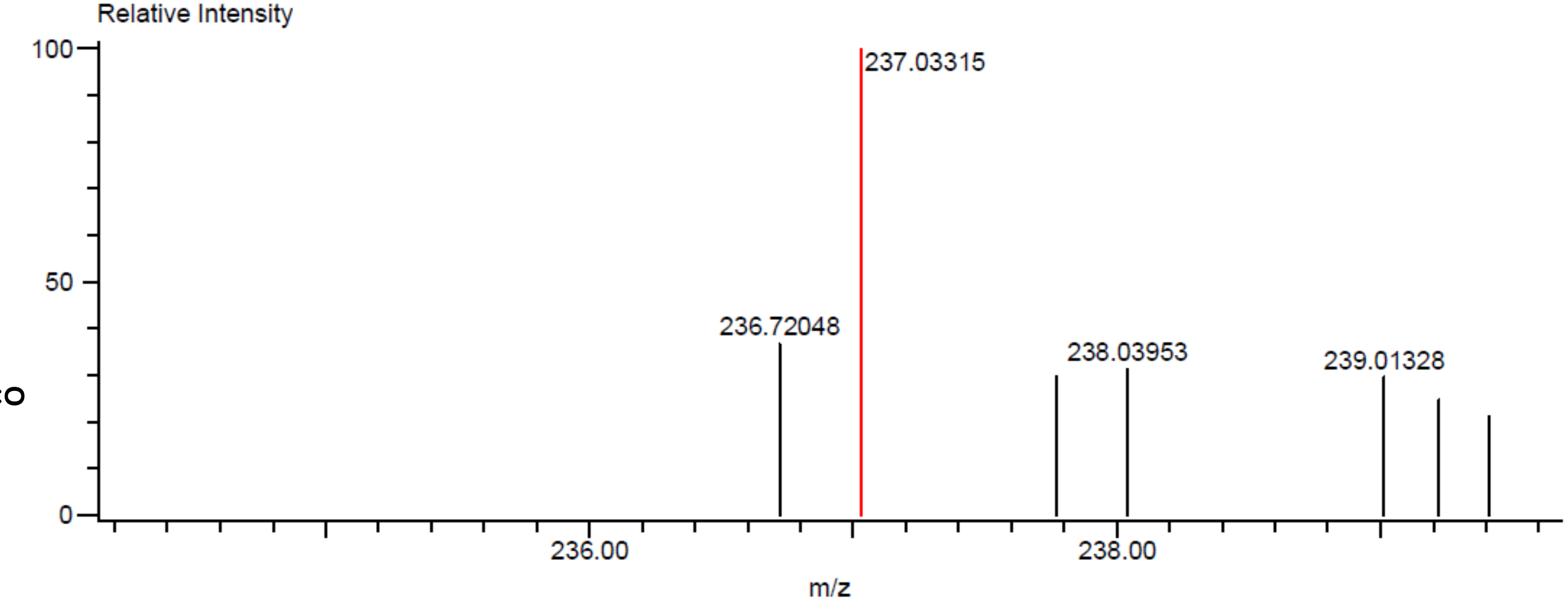

Mass	Intensity	Calc. Mass	Mass Difference [mDa]	Mass Difference [ppm]	Possible Formula
237.03315	1761.69	237.03339	-0.24	-0.99	$^{12}C_{10}{}^{1}H_{9}{}^{14}N_{2}{}^{16}O_{3}{}^{32}S_{1}$

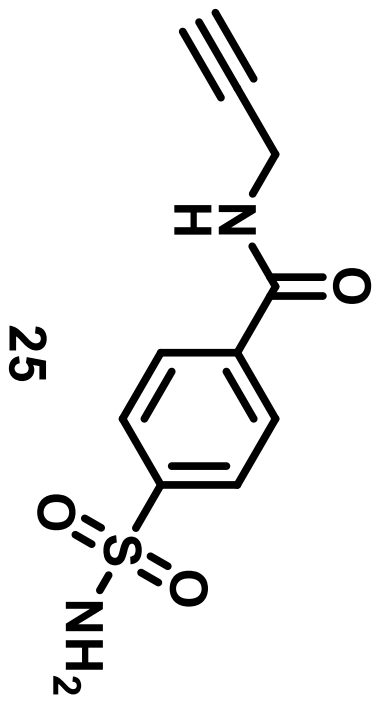

25

Figure S46. HRMS-ESI spectra of compound **25**.

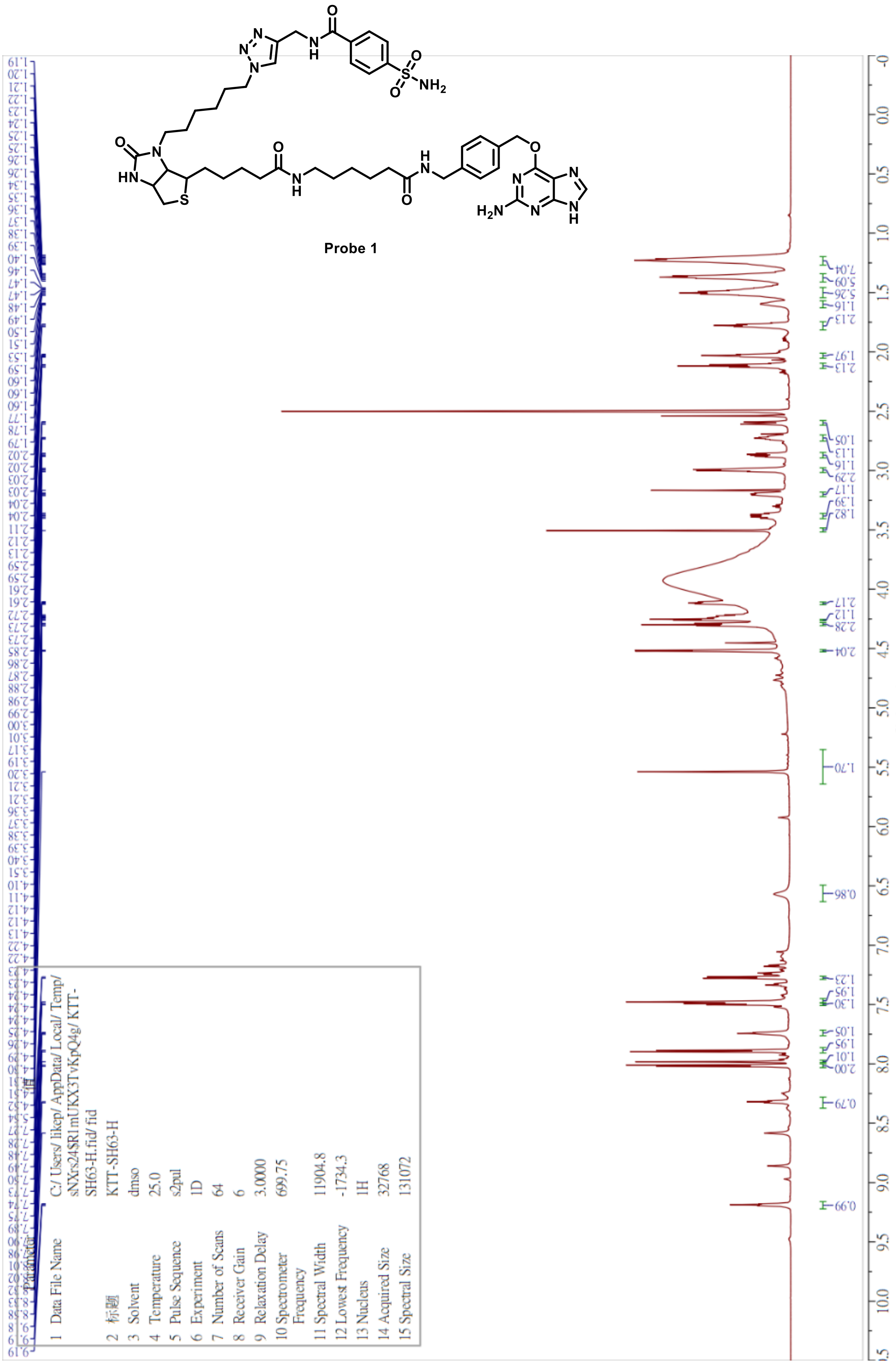

Figure S47. ^{1}H NMR spectra of **Probe 1**.

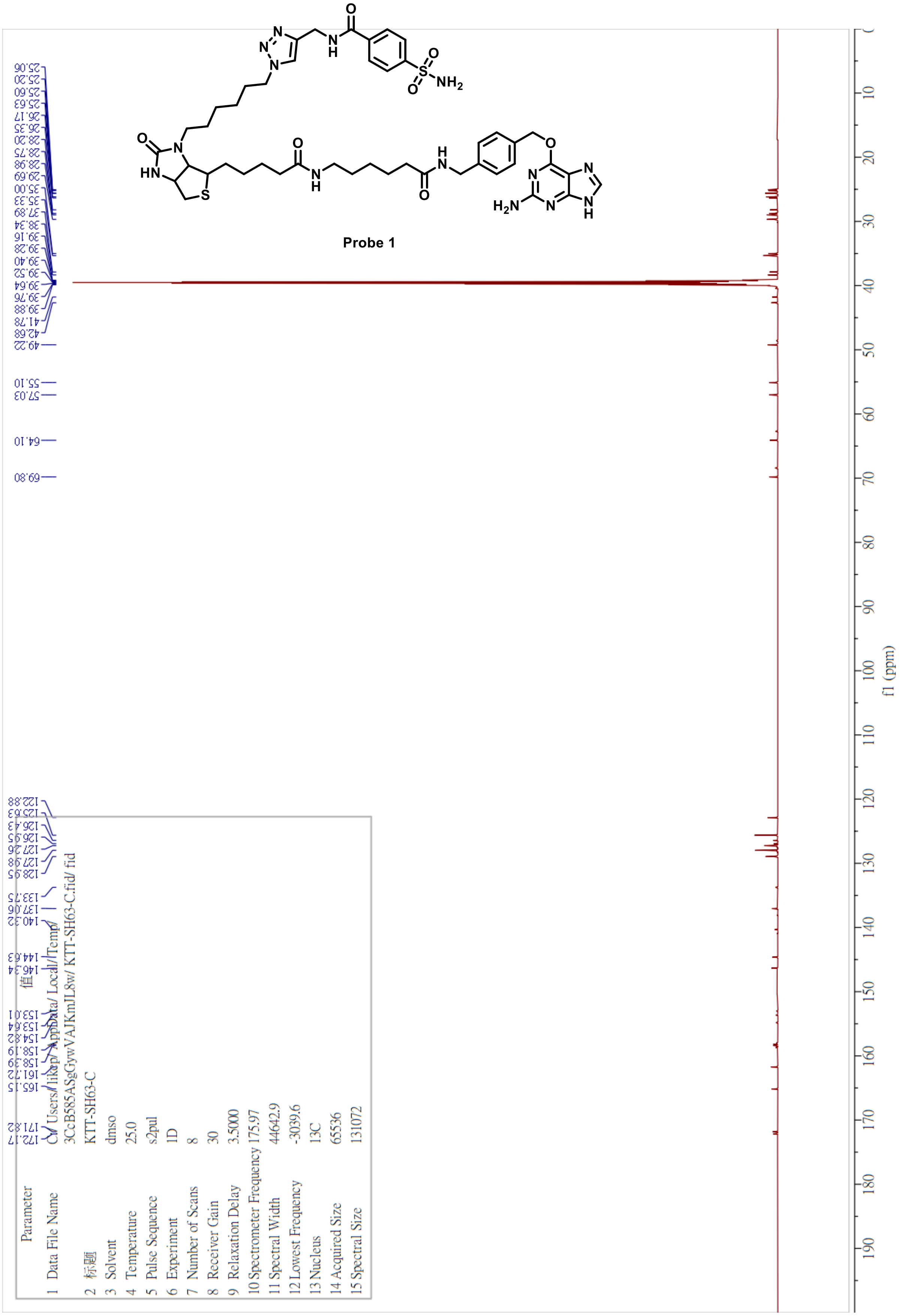

Figure S48. ^{13}C NMR spectra of **Probe 1**.

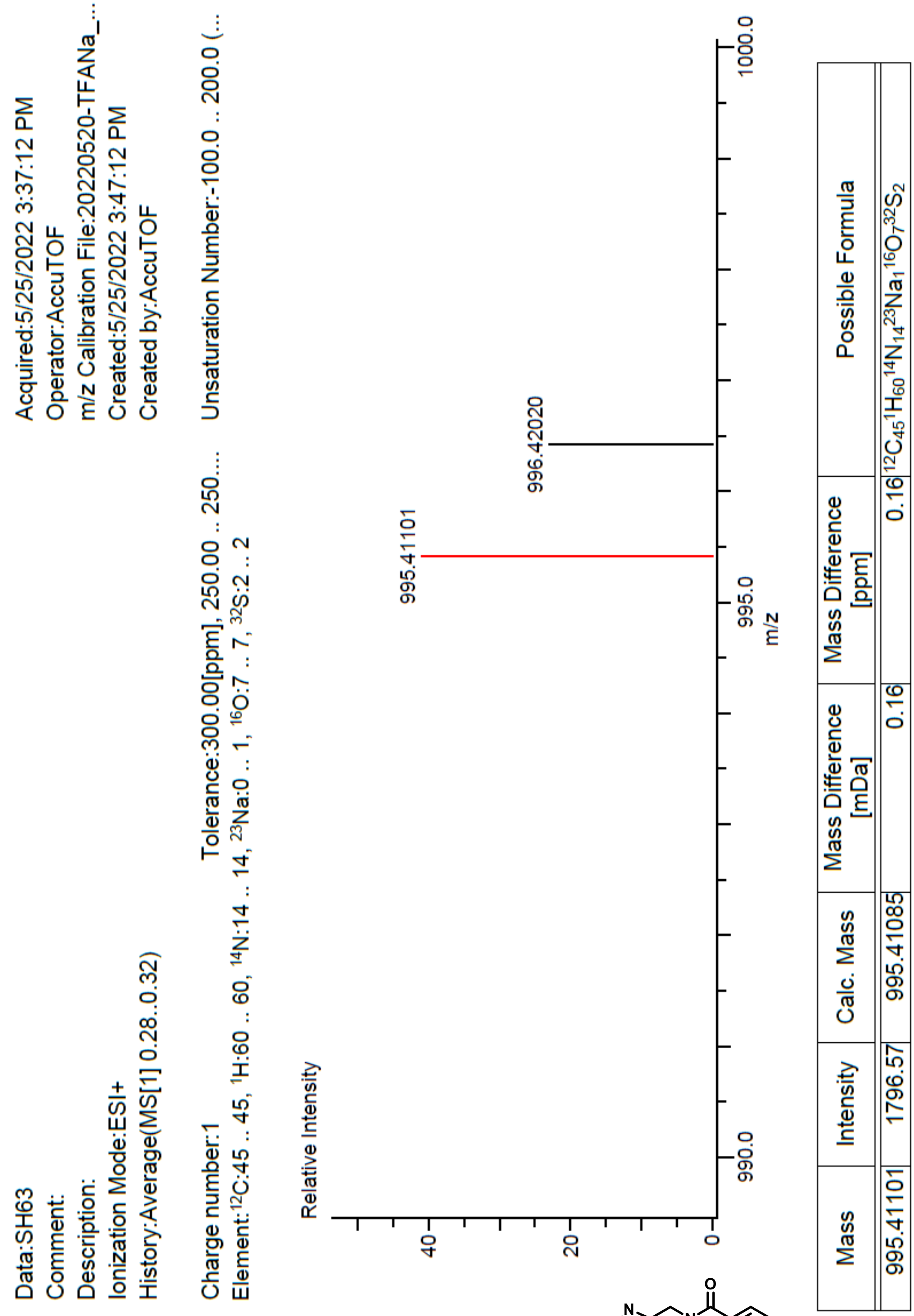

Mass	Intensity	Calc. Mass	Mass Difference [mDa]	Mass Difference [ppm]	Possible Formula
995.41101	1796.57	995.41085	0.16	0.16	$^{12}C_{45}{}^{1}H_{60}{}^{14}N_{14}{}^{23}Na_{1}{}^{16}O_{7}{}^{32}S_{2}$

Figure S49. HRMS-ESI spectra of **Probe 1**.

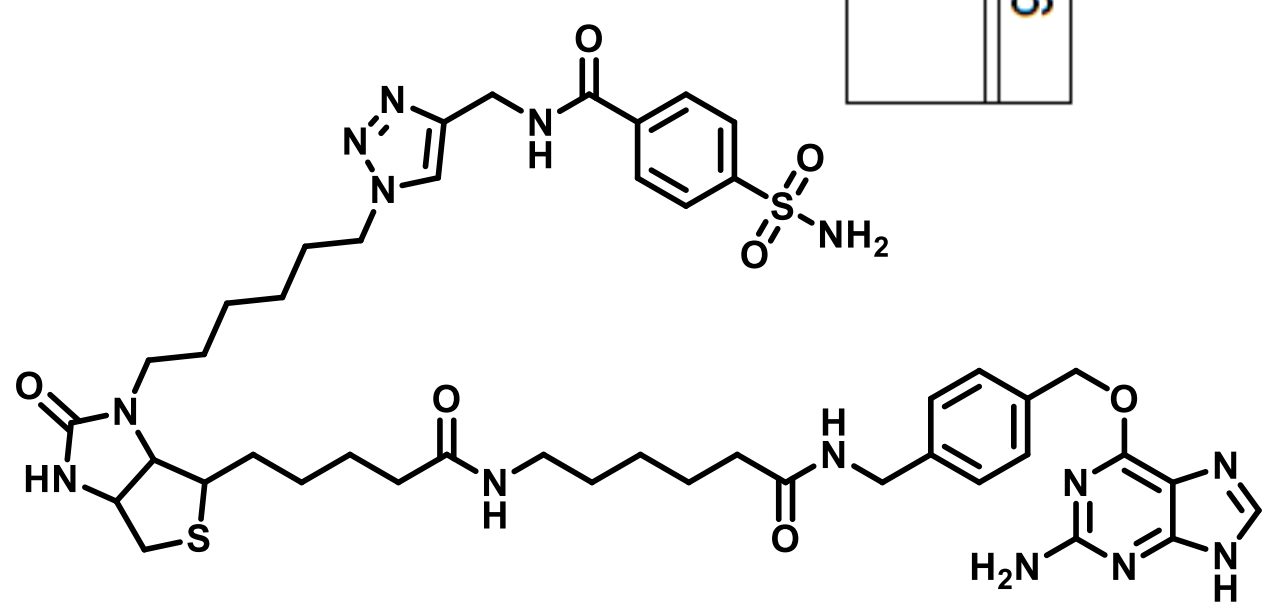

Probe 1

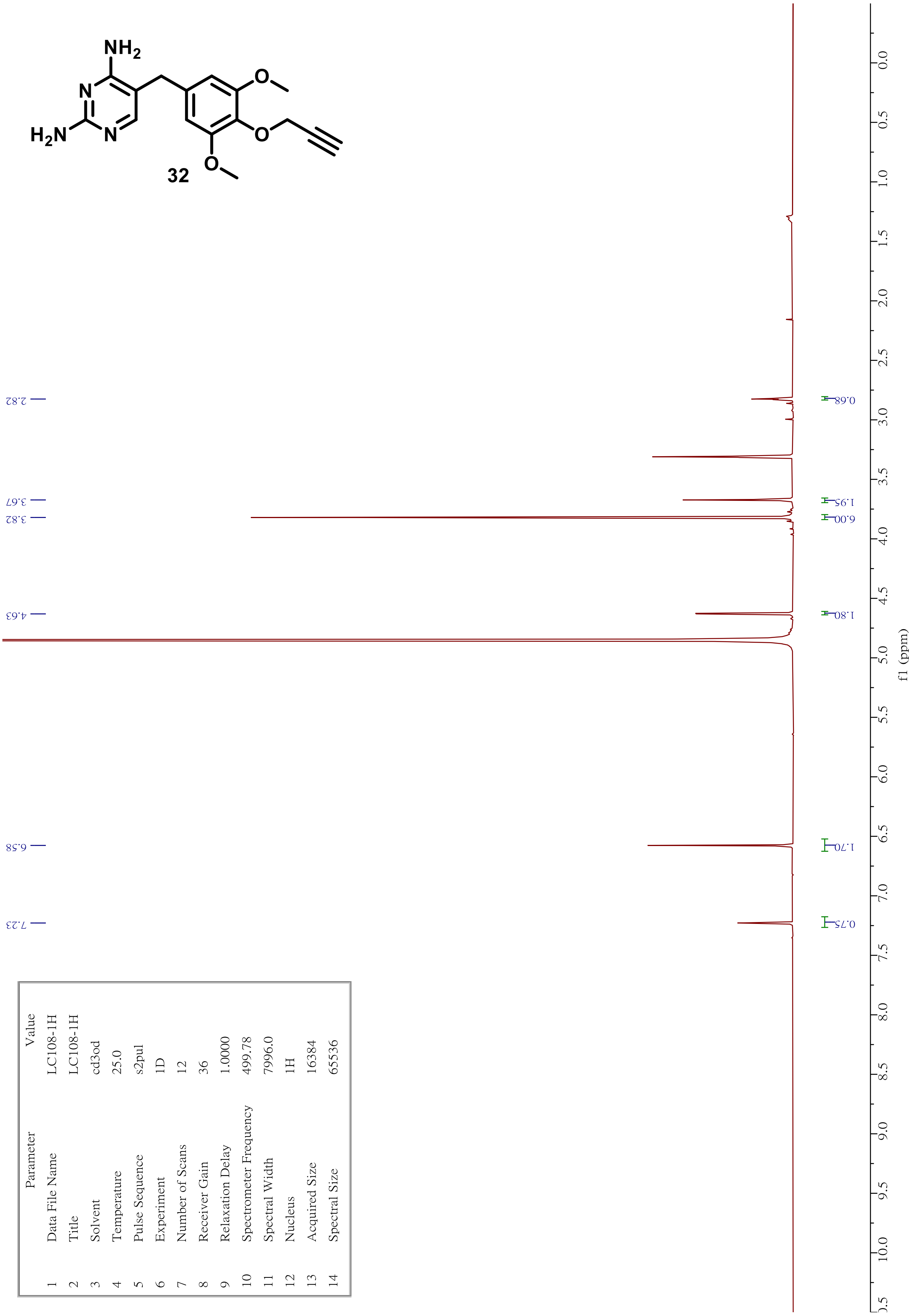

Figure S50. ^{1}H NMR spectra of compound **32**.

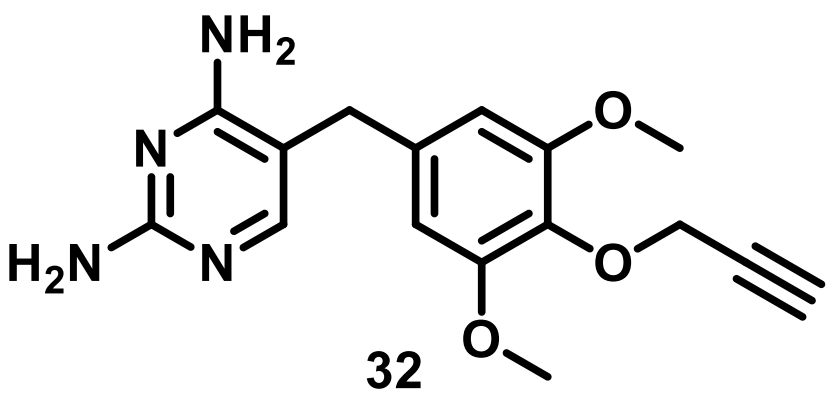

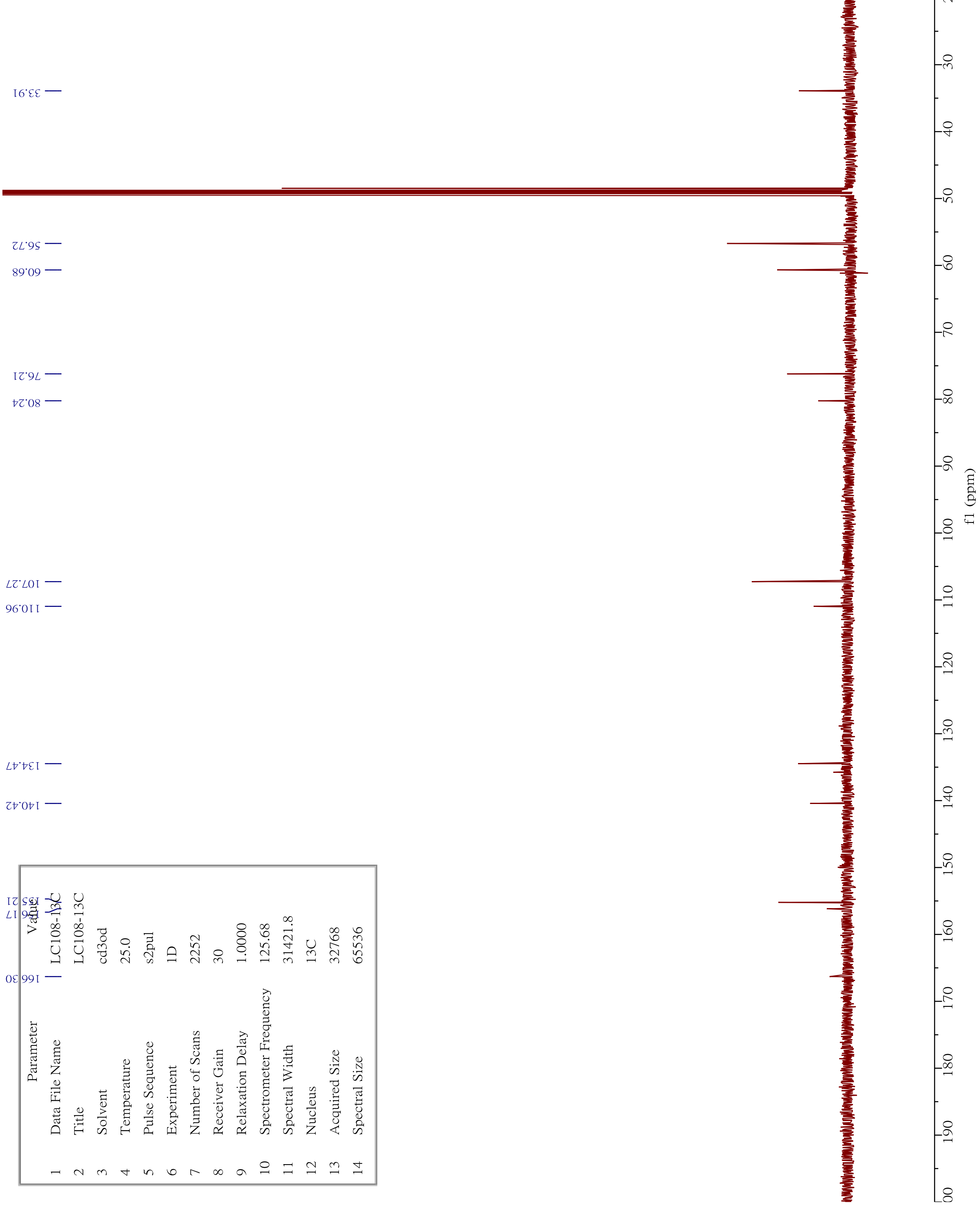

Figure S51. ^{13}C NMR spectra of compound **32**.

Data:LC108
Comment:
Description:
Ionization Mode:ESI+
History:Average(MS[1] 0.24..0.28)

Acquired:2/22/2023 3:38:28 PM
Operator:AccuTOF
m/z Calibration File:20230112-TFANa_...
Created:2/22/2023 3:41:11 PM
Created by:AccuTOF

Charge number:1
Tolerance:200.00[ppm], 250.00 .. 250....
Unsaturation Number:-100.5 .. 200.0 (...
Element:^{12}C:16 .. 16, ^{1}H:0 .. 19, ^{14}N:4 .. 4, ^{23}Na:0 .. 1, ^{16}O:3 .. 3

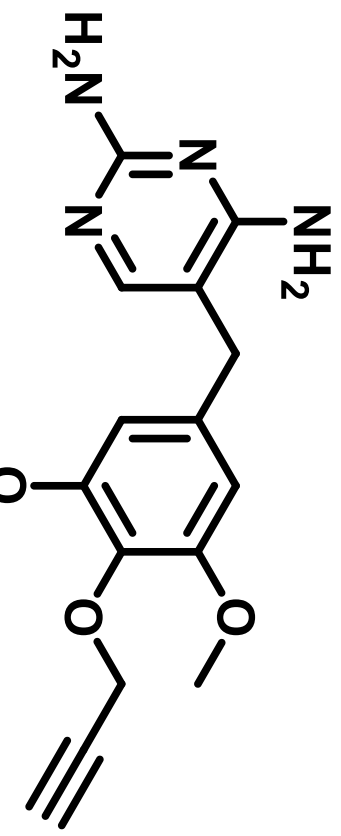

32

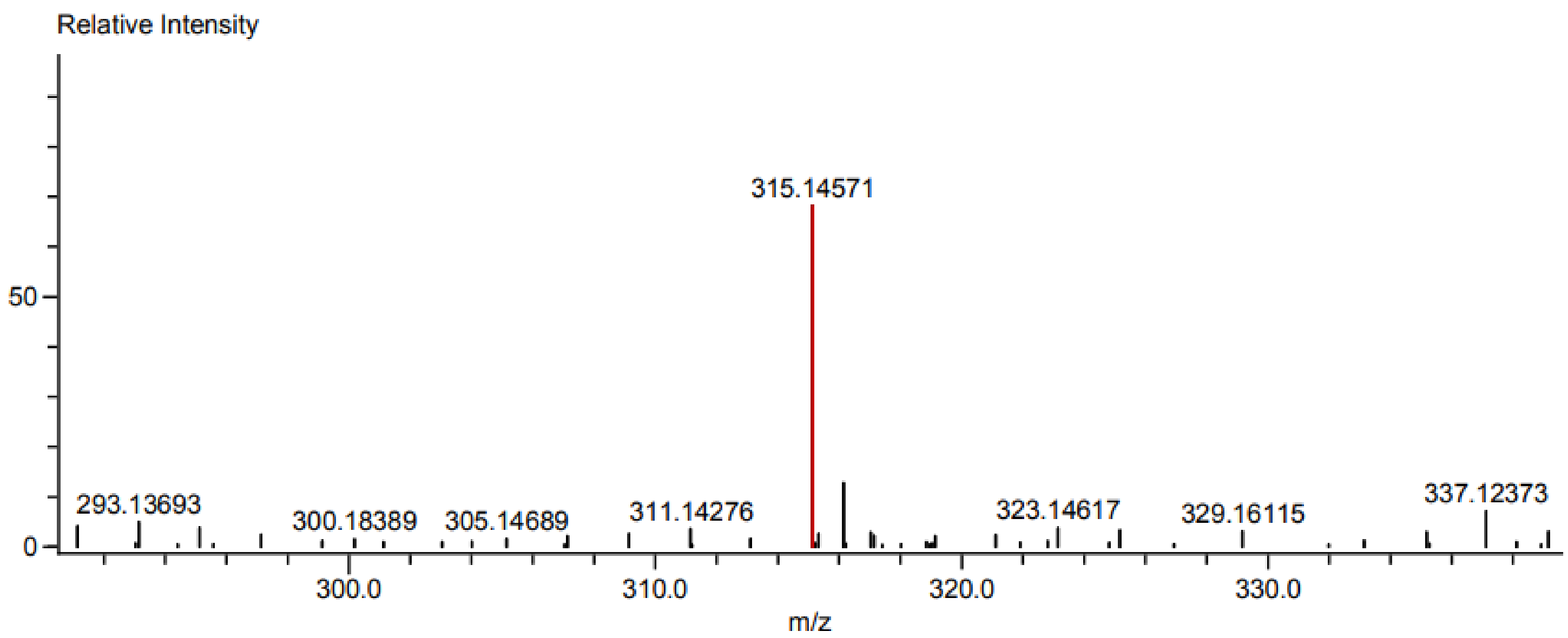

Mass	Intensity	Calc. Mass	Mass Difference [mDa]	Mass Difference [ppm]	Possible Formula
315.14571	15680.53	315.14571	0.00	0.00	$^{12}C_{16}{}^{1}H_{19}{}^{14}N_{4}{}^{16}O_{3}$

Figure S52. HRMS-ESI spectra of compound **32**.

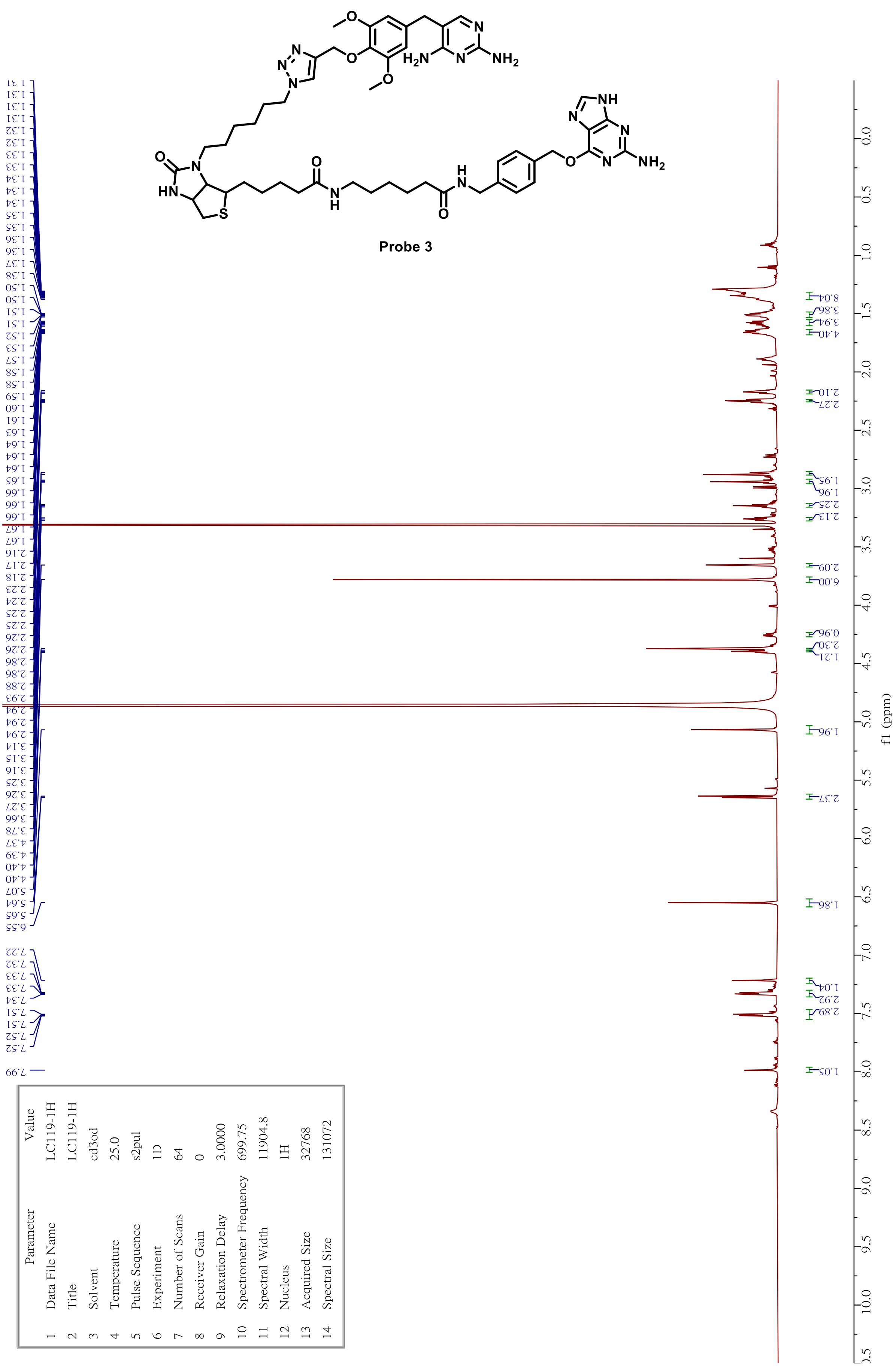

Figure S53. ^{1}H NMR spectra of **Probe 3**.

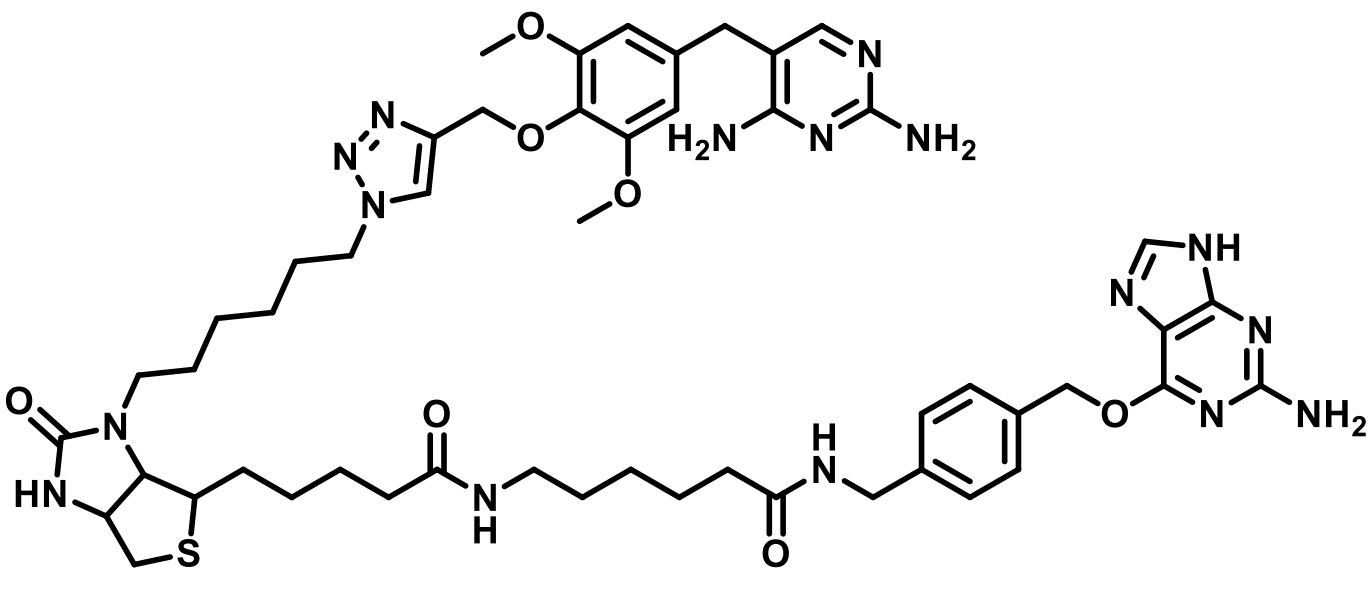

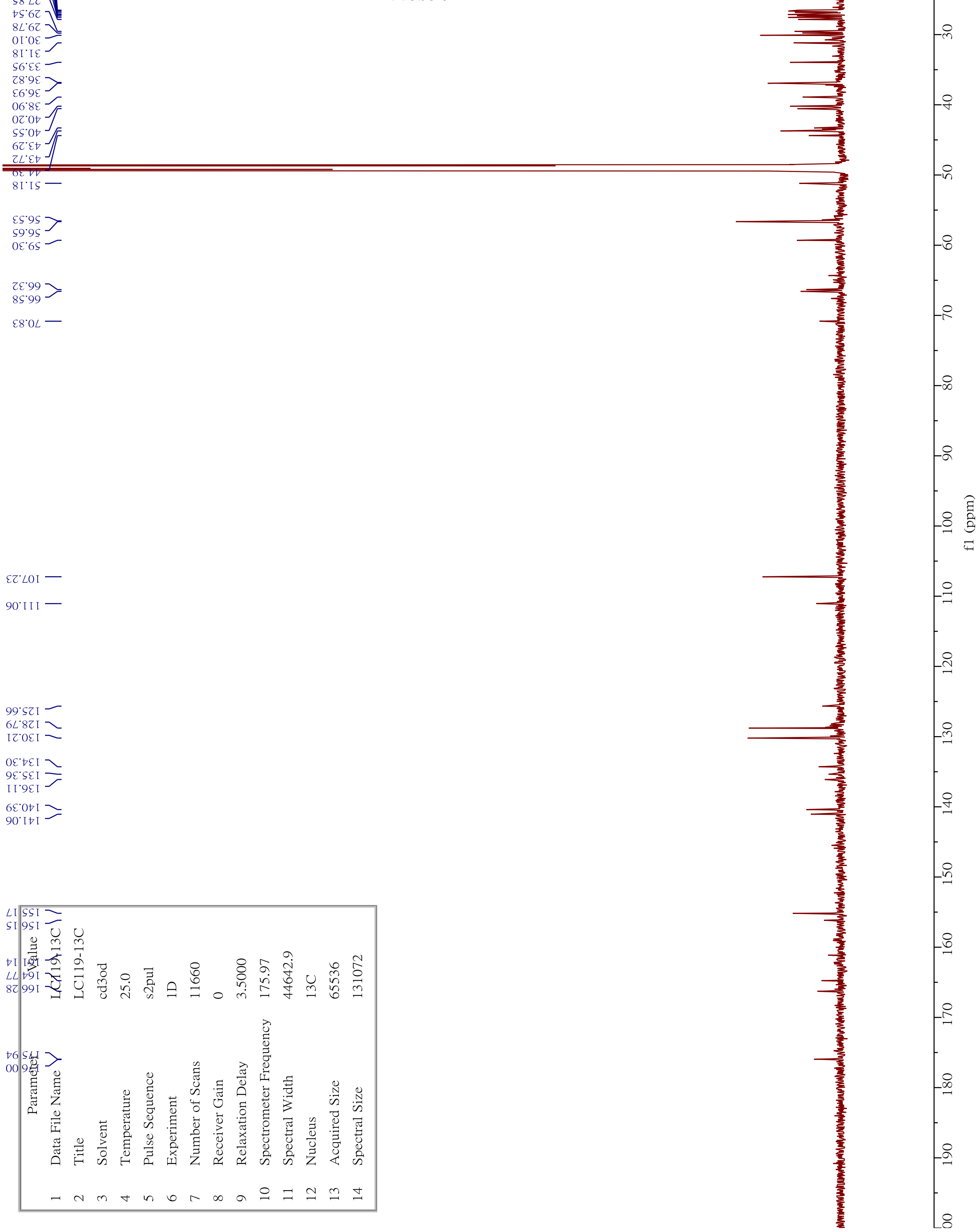

	Parameter	Value
1	Data File Name	LC119-13C
2	Title	LC119-13C
3	Solvent	cd3od
4	Temperature	25.0
5	Pulse Sequence	s2pul
6	Experiment	1D
7	Number of Scans	11660
8	Receiver Gain	0
9	Relaxation Delay	3.5000
10	Spectrometer Frequency	175.97
11	Spectral Width	44642.9
12	Nucleus	13C
13	Acquired Size	65536
14	Spectral Size	131072

Figure S54. ^{13}C NMR spectra of **Probe 3**.

Data:LC119-
Comment:
Description:
Ionization Mode:ESI-
History:Average(MS[1] 0.22..0.32)

Acquired:3/8/2023 3:47:22 PM
Operator:AccuTOF
m/z Calibration File:20221220-TFANa_...
Created:3/8/2023 3:56:06 PM
Created by:AccuTOF

Charge number:1
Tolerance:300.00[ppm], 300.00 .. 300....
Unsaturation Number:-200.5 .. 200.0 (...
Element:^{12}C:51 .. 51, ^{1}H:51 .. 68, ^{35}Cl:0 .. 1, ^{14}N:16 .. 16, ^{23}Na:0 .. 1, ^{16}O:7 .. 7, ^{32}S:1 .. 1

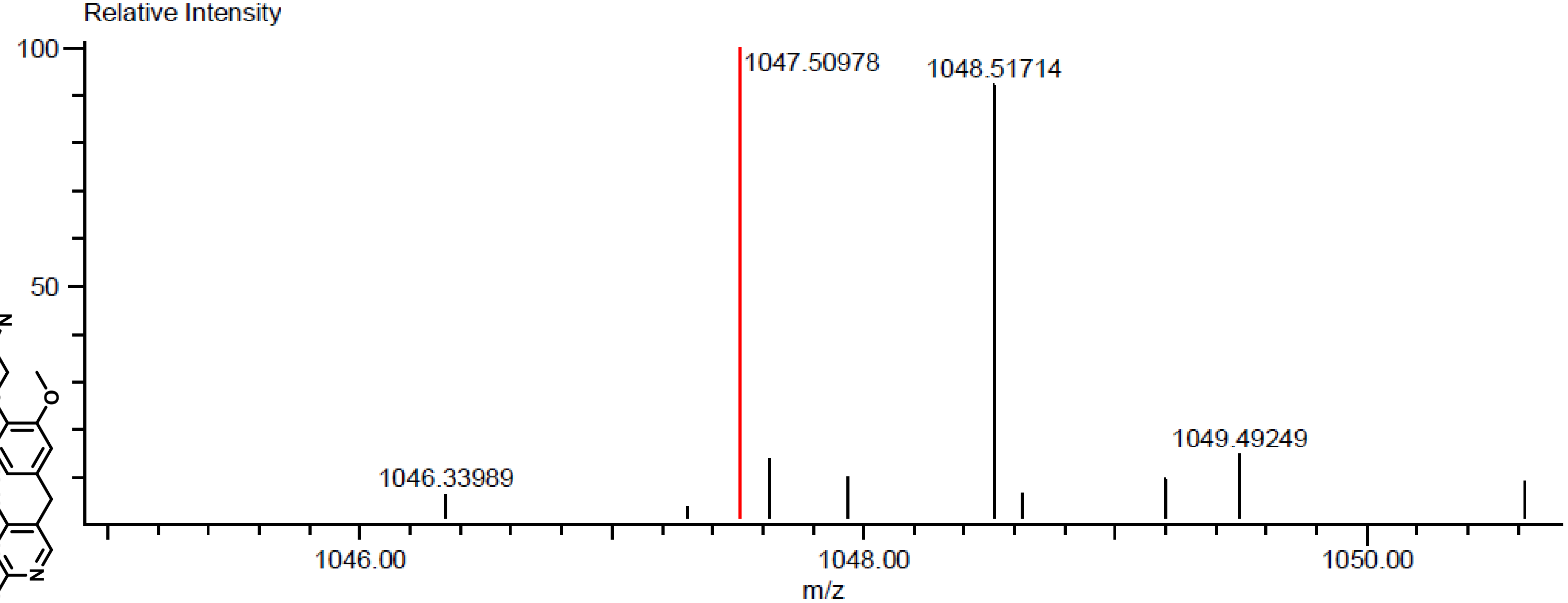

Mass	Intensity	Calc. Mass	Mass Difference [mDa]	Mass Difference [ppm]	Possible Formula
1047.50978	1156.00	1047.50993	-0.15	-0.15	$^{12}C_{51}{}^{1}H_{67}{}^{14}N_{16}{}^{16}O_{7}{}^{32}S_{1}$

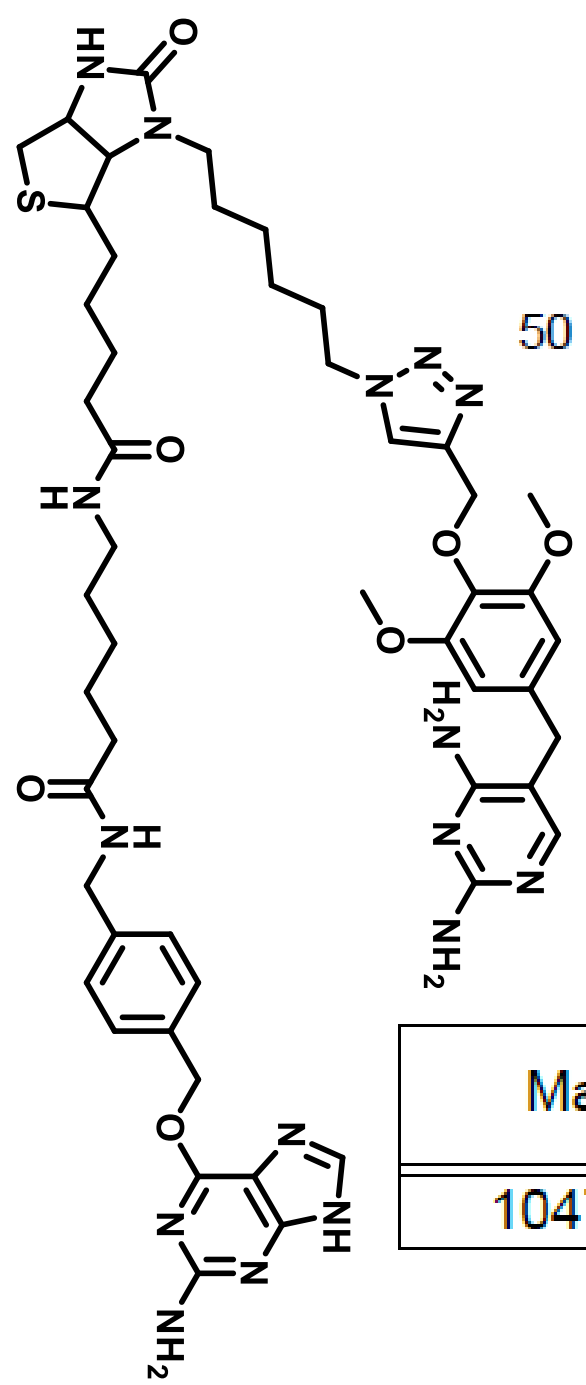

Probe 3

Figure S55. HRMS-ESI spectra of **Probe 3**.

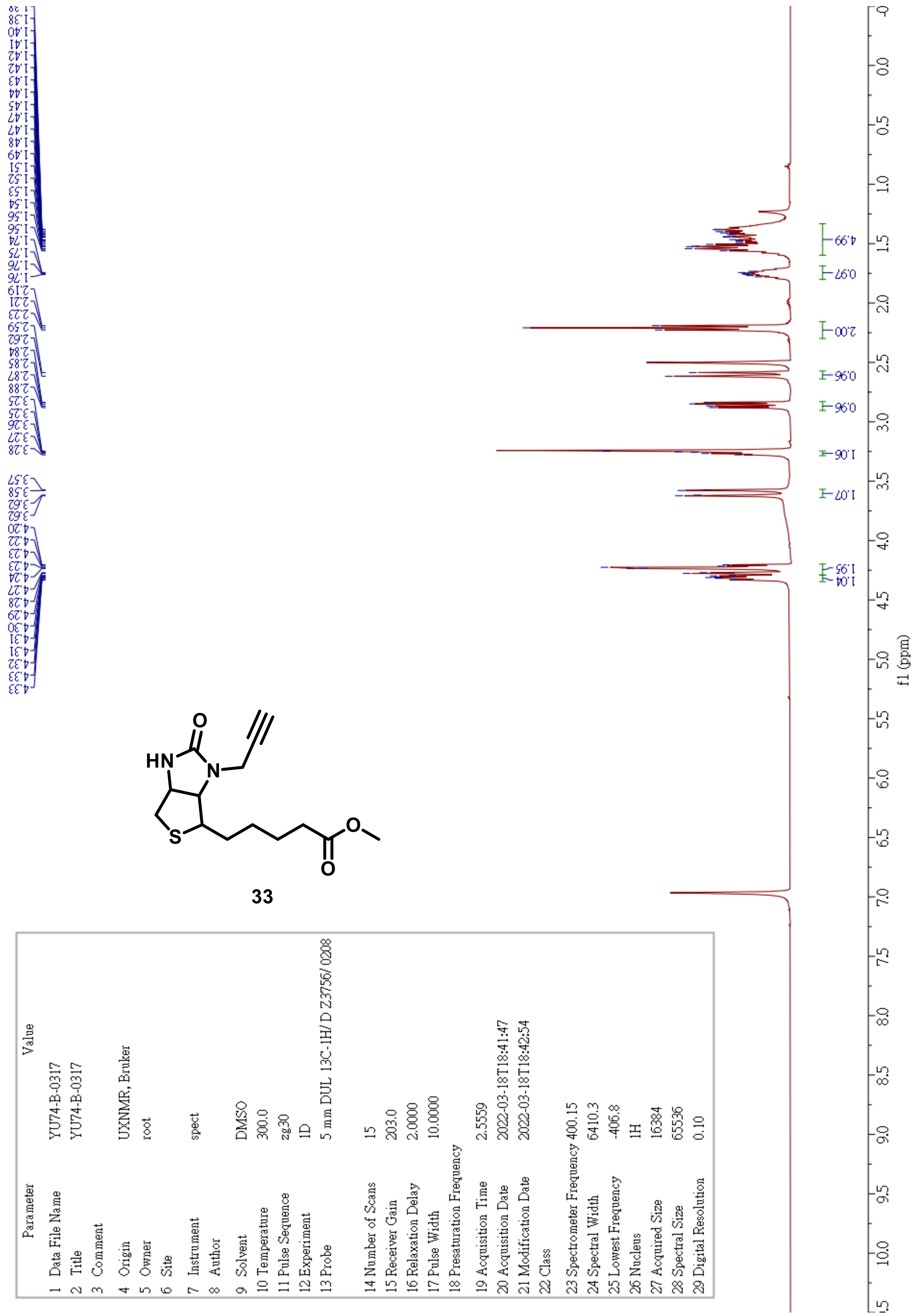

Figure S56. ^{1}H NMR spectra of compound **33**.

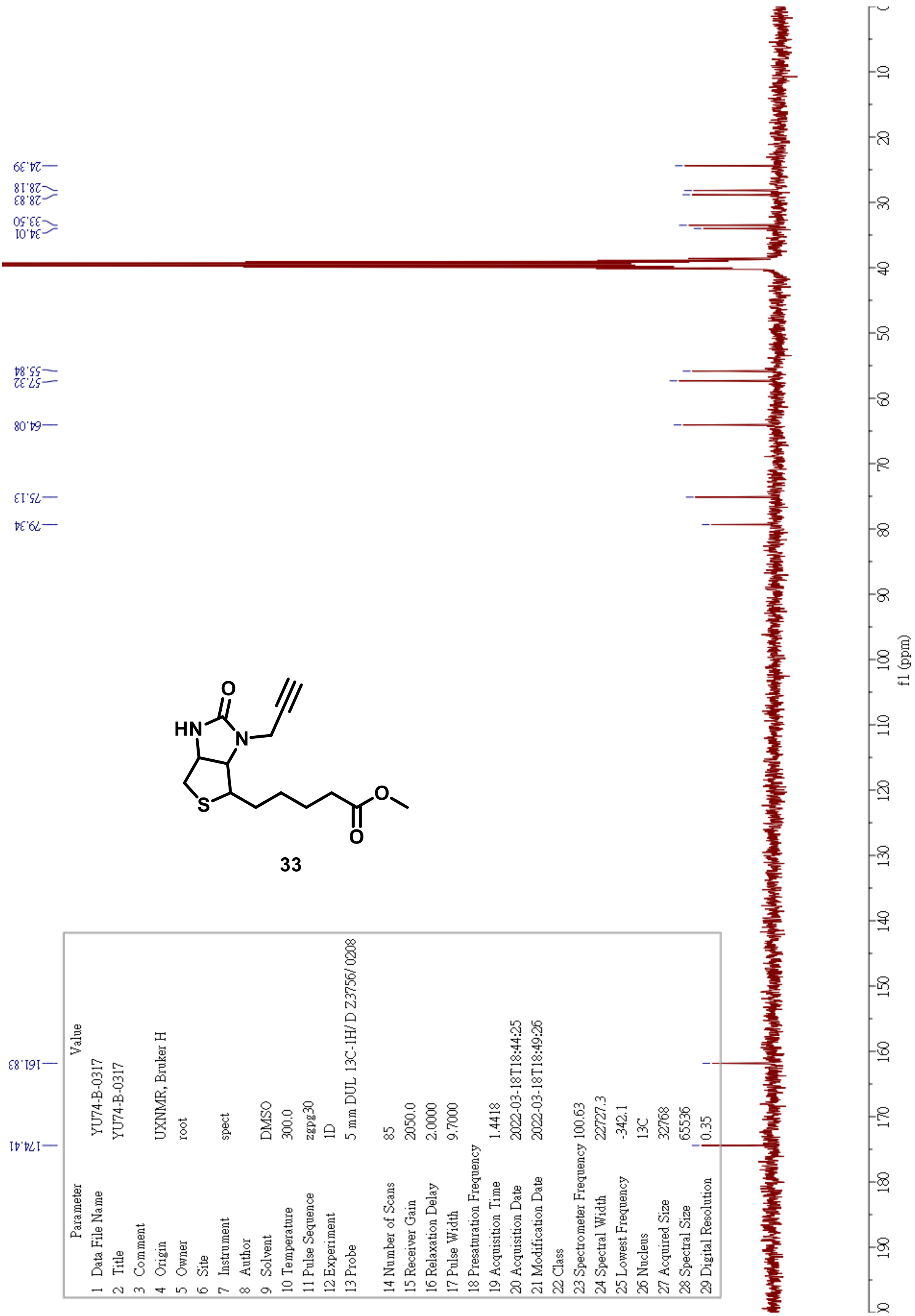

Figure S57. ^{13}C NMR spectra of compound **33**.

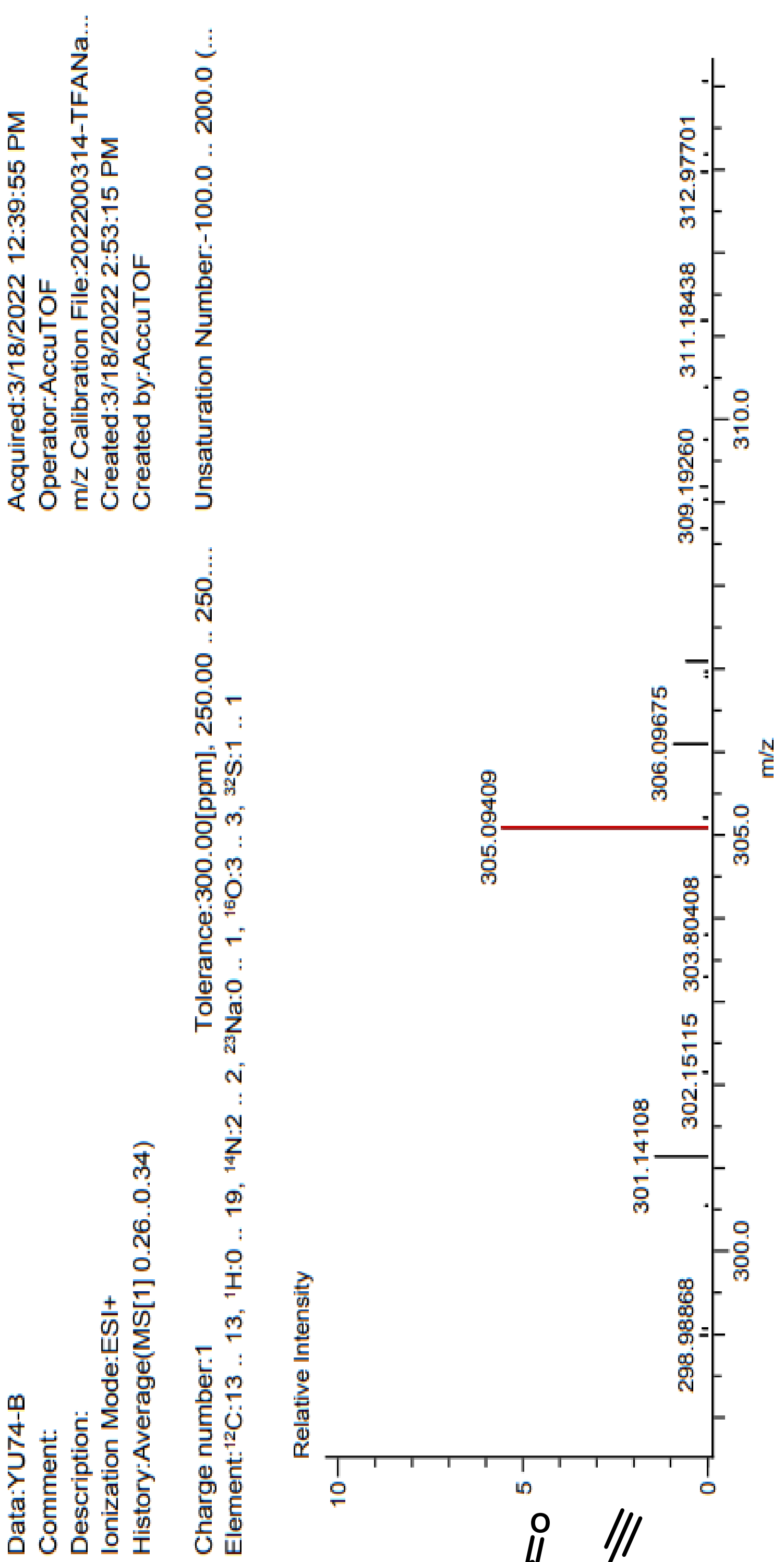

Mass	Intensity	Calc. Mass	Mass Difference [mDa]	Mass Difference [ppm]	Possible Formula
305.09409	9033.51	305.09358	0.51	1.66	$^{12}C_{13}{}^{1}H_{18}{}^{14}N_{2}{}^{23}Na_{1}{}^{16}O_{3}{}^{32}S_{1}$

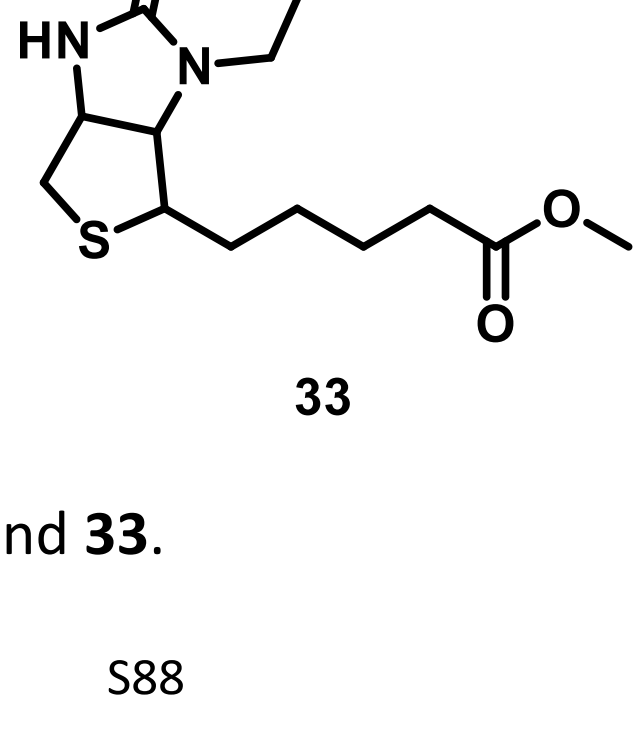

33

Figure S58. HRMS-ESI spectra of compound **33**.

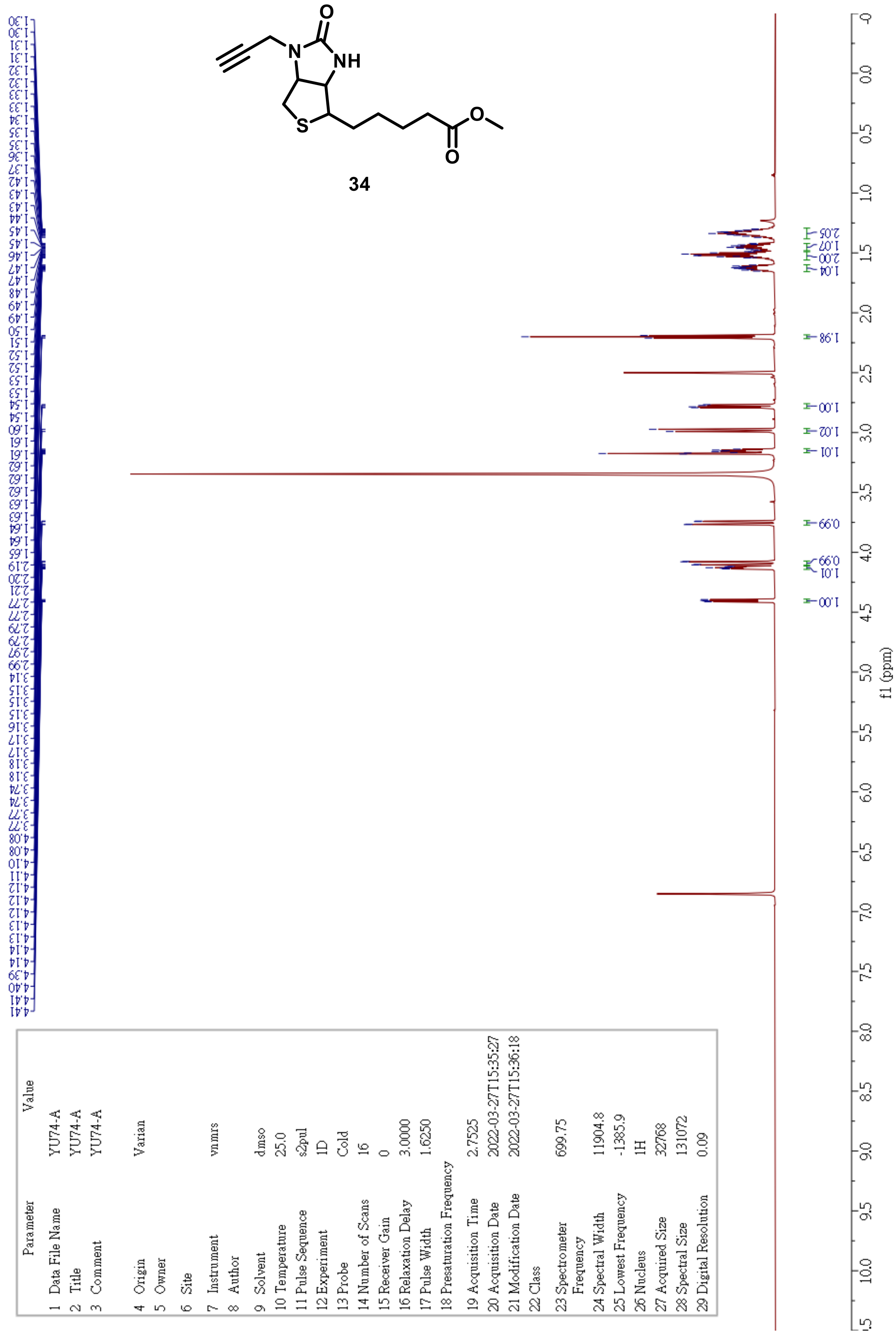

Figure S59. ^{1}H NMR spectra of compound **34**.

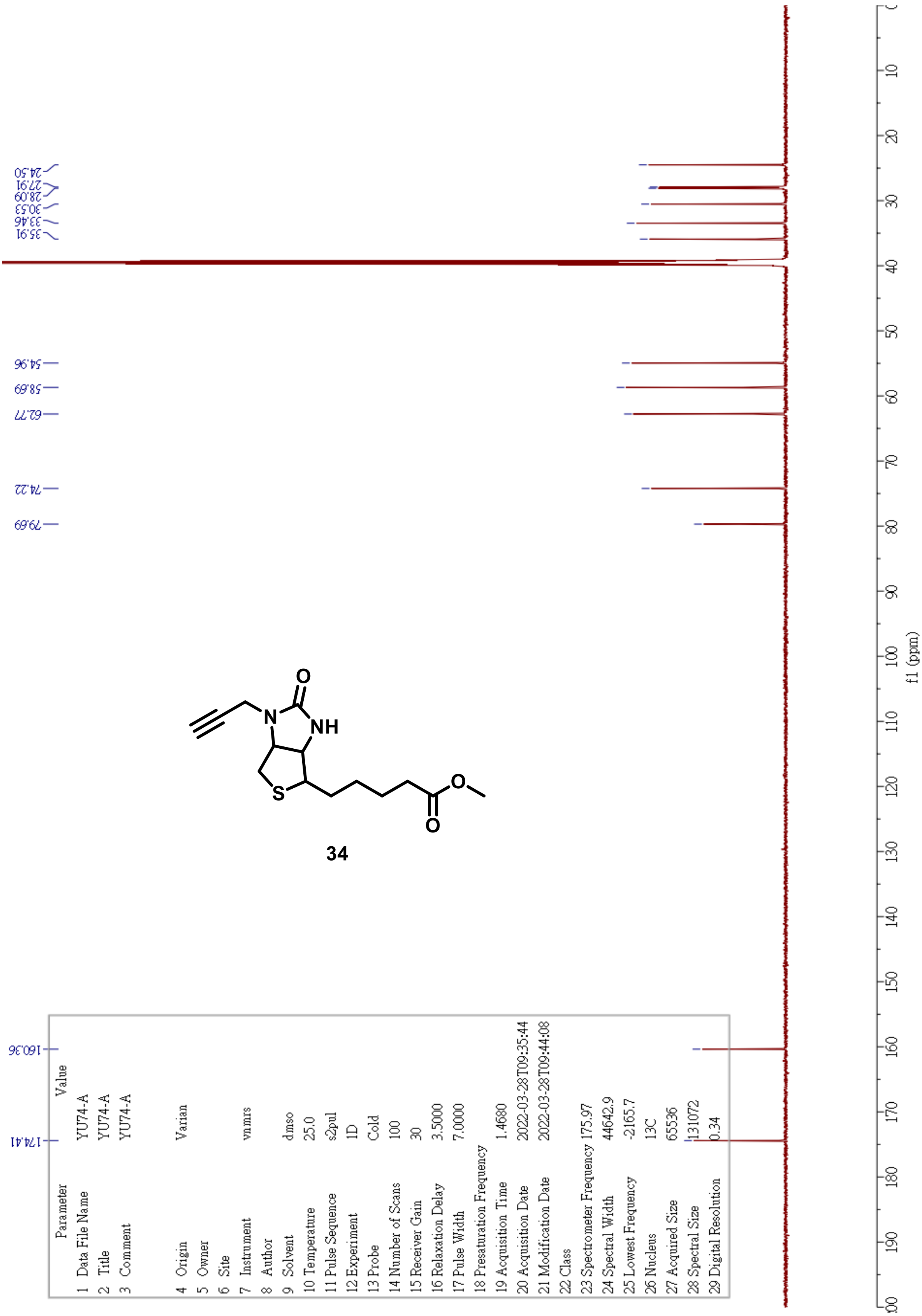

Figure S60. ^{13}C NMR spectra of compound **34**.

Data:YU74-A-
Comment:
Description:
Ionization Mode:ESI-
History:Average(MS[1] 0.39..0.43)

Acquired:3/17/2022 2:34:18 PM
Operator:AccuTOF
m/z Calibration File:2021117-TFANa_...
Created:3/17/2022 4:47:00 PM
Created by:AccuTOF

Charge number:1
Tolerance:300.00[ppm], 250.00 .. 250....
Unsaturation Number:-100.0 .. 200.0 (...
Element:^{12}C:13 .. 13, ^{1}H:0 .. 18, ^{14}N:2 .. 2, ^{23}Na:0 .. 4, ^{16}O:3 .. 3, ^{32}S:1 .. 1

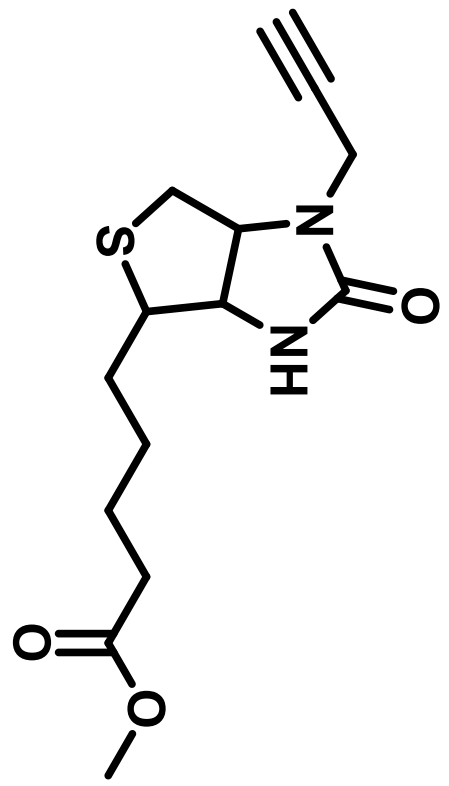

34

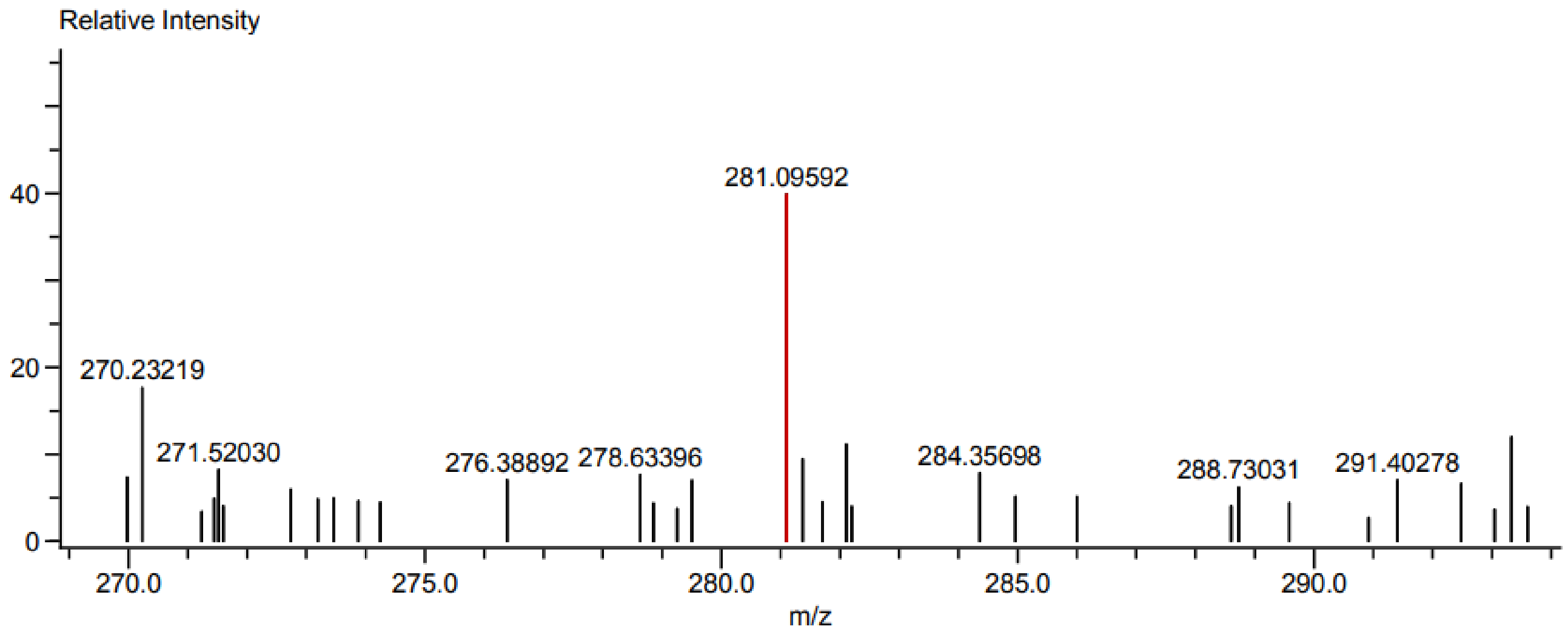

Mass	Intensity	Calc. Mass	Mass Difference [mDa]	Mass Difference [ppm]	Possible Formula
281.09592	1015.33	281.09599	-0.06	-0.22	$^{12}C_{13}{}^{1}H_{17}{}^{14}N_{2}{}^{16}O_{3}{}^{32}S_{1}$

Figure S61. HRMS-ESI spectra of compound **34**.

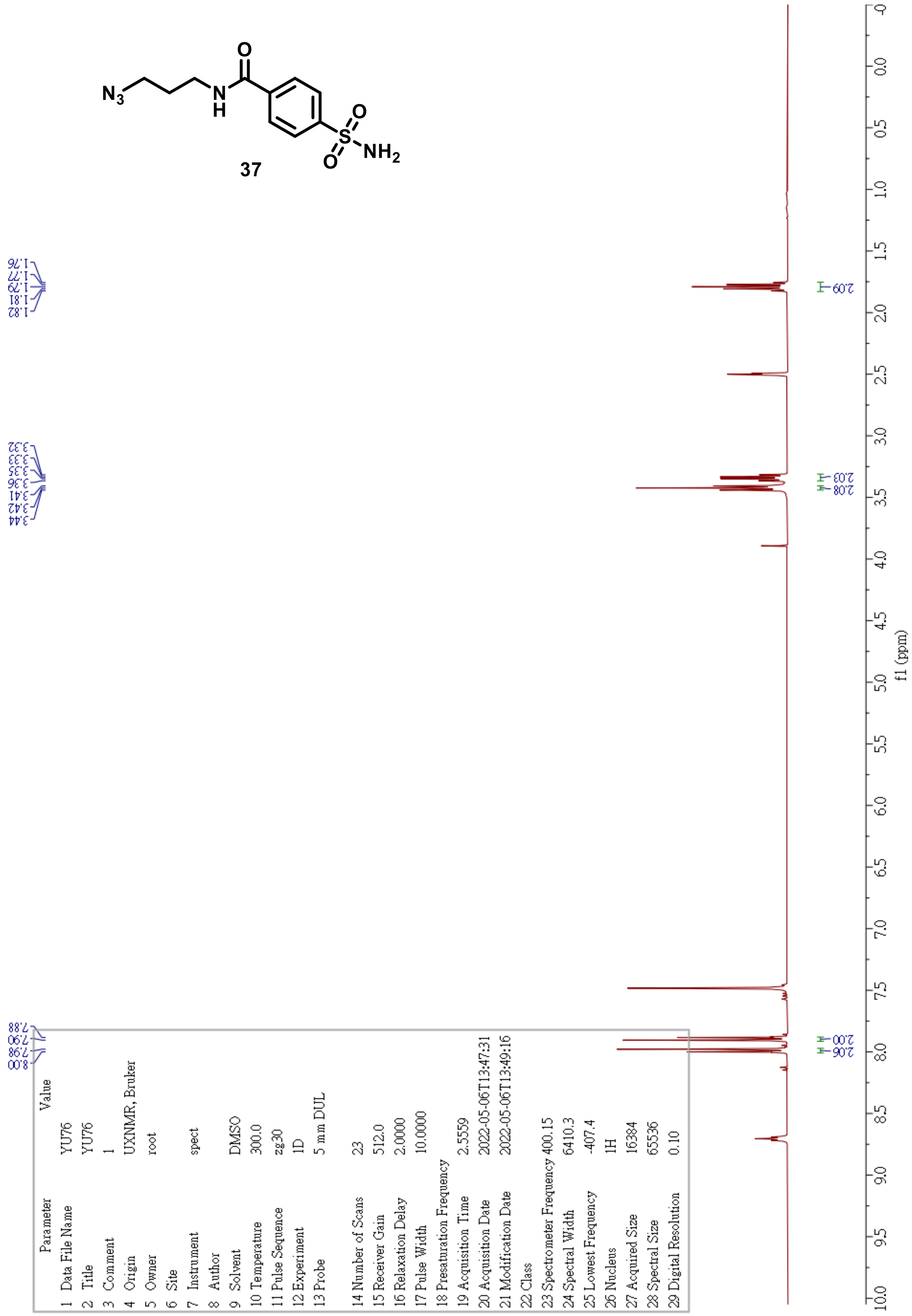

Figure S62. ^{1}H NMR spectra of compound **37**.

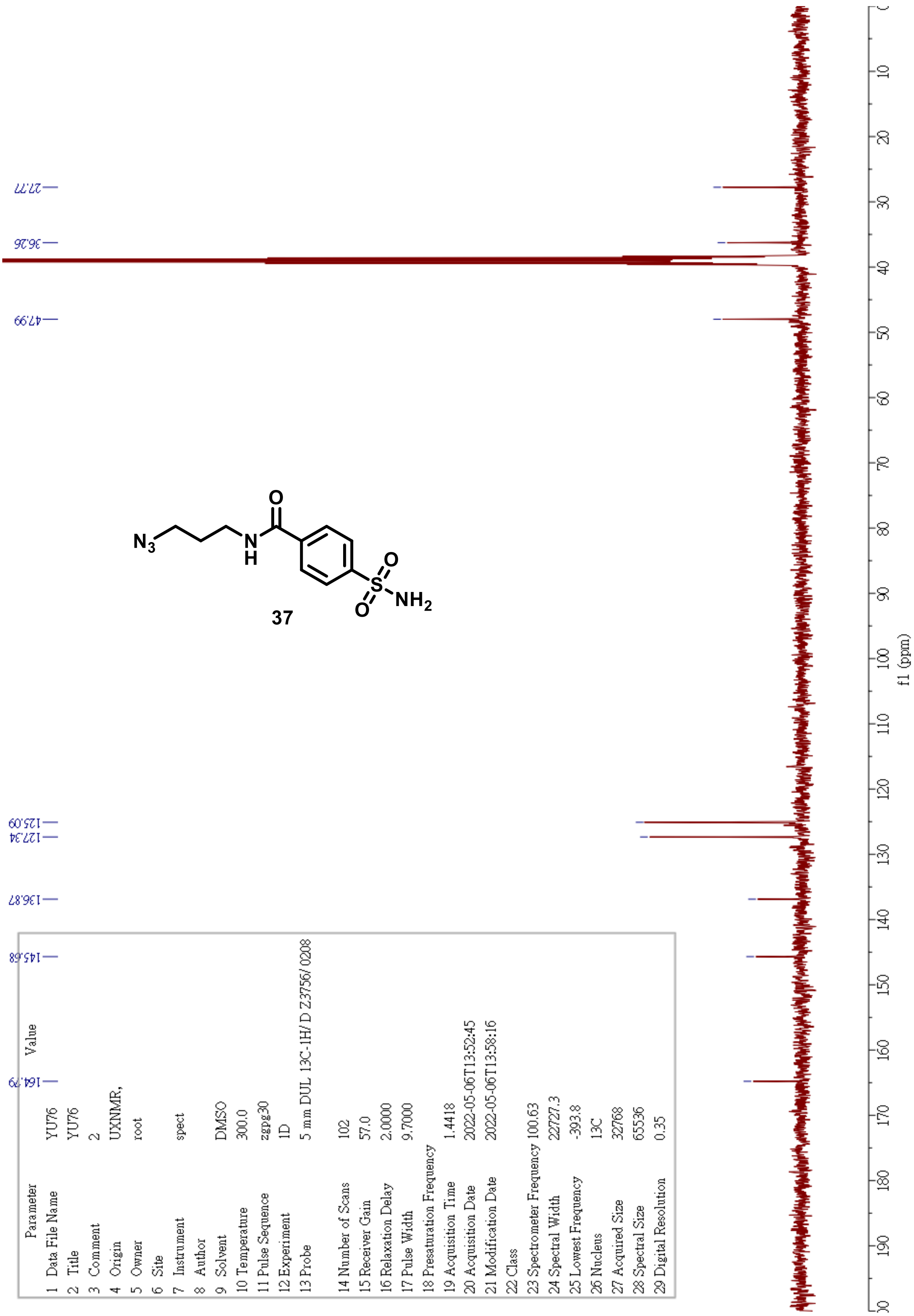

Figure S63. ^{13}C NMR spectra of compound **37**.

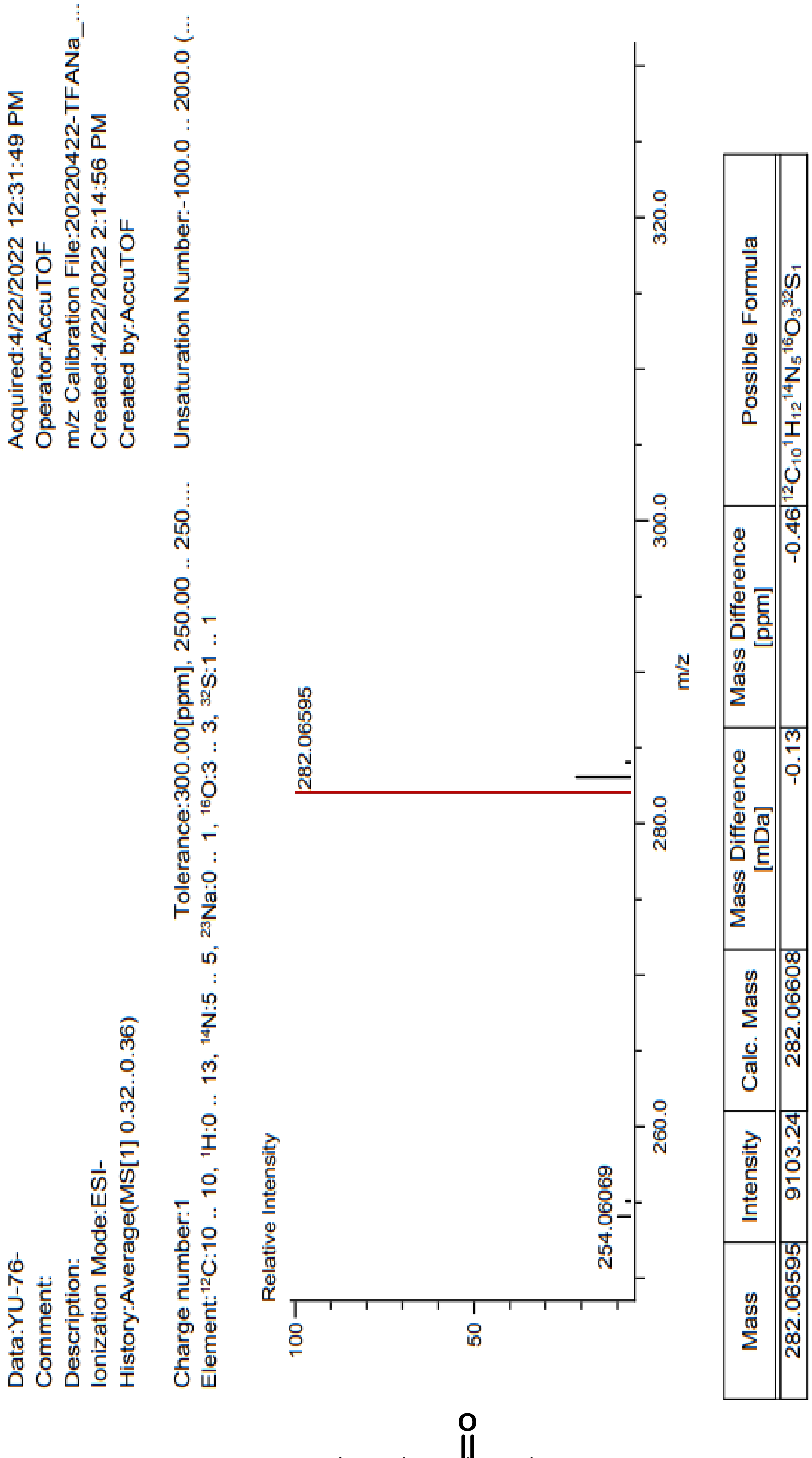

Mass	Intensity	Calc. Mass	Mass Difference [mDa]	Mass Difference [ppm]	Possible Formula
282.06595	9103.24	282.06608	-0.13	-0.46	$^{12}C_{10}\,^{1}H_{12}\,^{14}N_{5}\,^{16}O_{3}\,^{32}S_{1}$

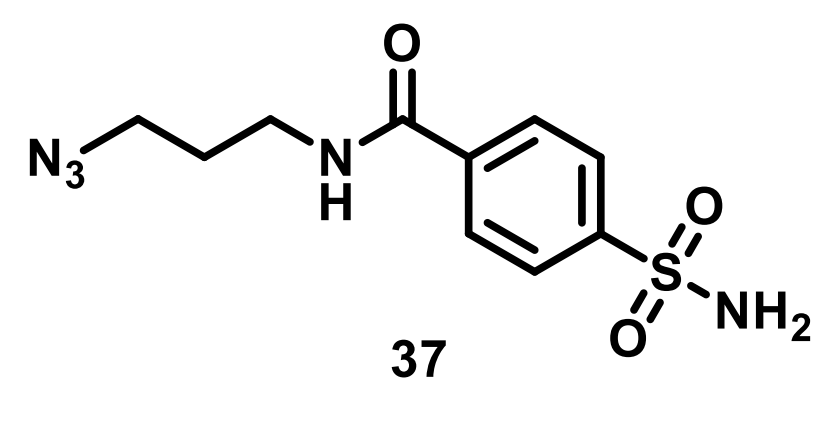

37

Figure S64. HRMS-ESI spectra of compound **37**.

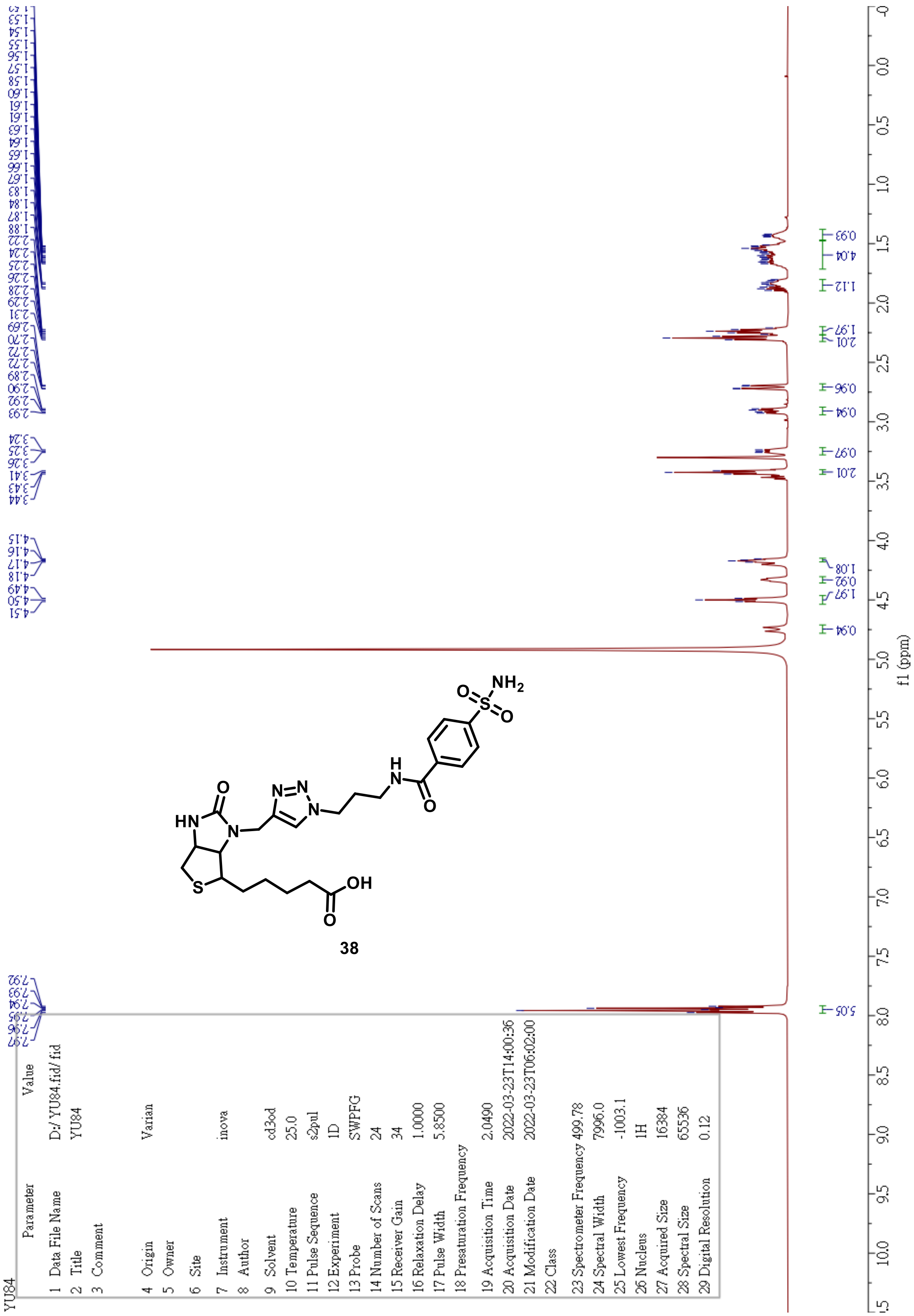

Figure S65. ^{1}H NMR spectra of compound **38**.

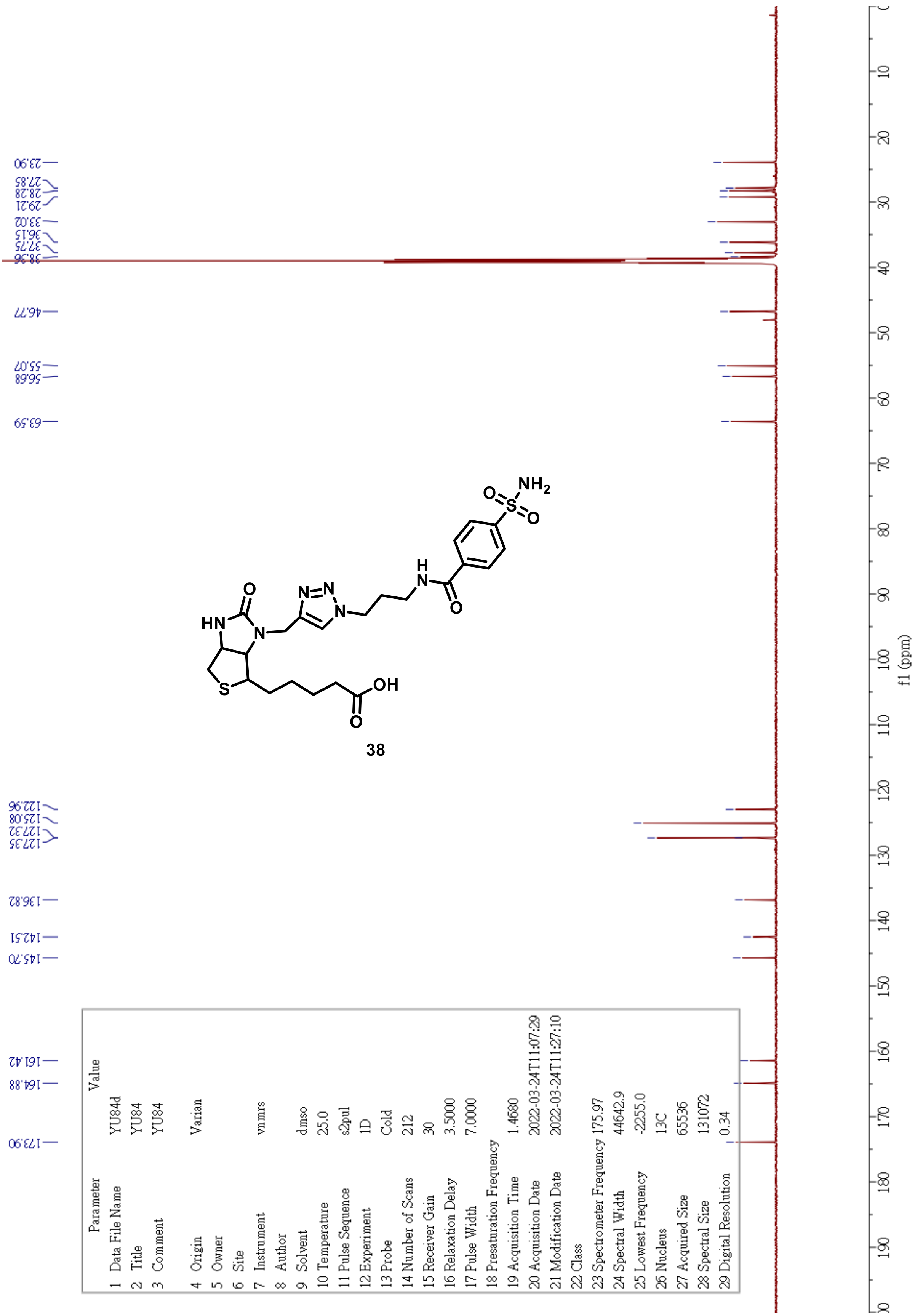

38

Figure S66. ^{13}C NMR spectra of compound **38**.

Data:YU84-
Comment:
Description:
Ionization Mode:ESI+
History:Average(MS[1] 0.33..0.36)

Acquired:4/11/2022 2:33:52 PM
Operator:AccuTOF
m/z Calibration File:202200324-TFANa...
Created:4/11/2022 4:20:02 PM
Created by:AccuTOF

Charge number:1 Tolerance:300.00[ppm], 250.00 .. 250.... Unsaturation Number:-100.0 .. 200.0 (...
Element:^{12}C:23 .. 23, ^{1}H:31 .. 32, ^{14}N:7 .. 7, ^{23}Na:0 .. 2, ^{16}O:6 .. 6, ^{32}S:2 .. 2

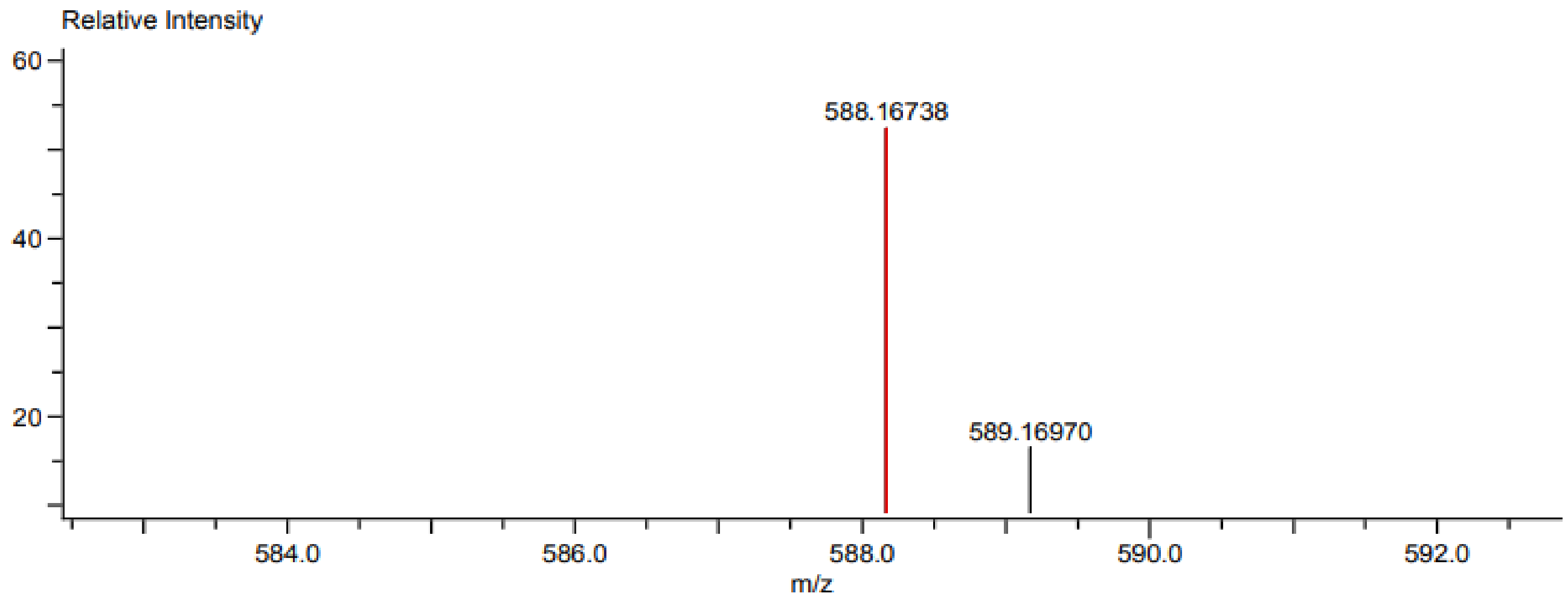

Mass	Intensity	Calc. Mass	Mass Difference [mDa]	Mass Difference [ppm]	Possible Formula
588.16738	31399.18	588.16749	-0.11	-0.18	$^{12}C_{23}{}^{1}H_{31}{}^{14}N_{7}{}^{23}Na_{1}{}^{16}O_{6}{}^{32}S_{2}$

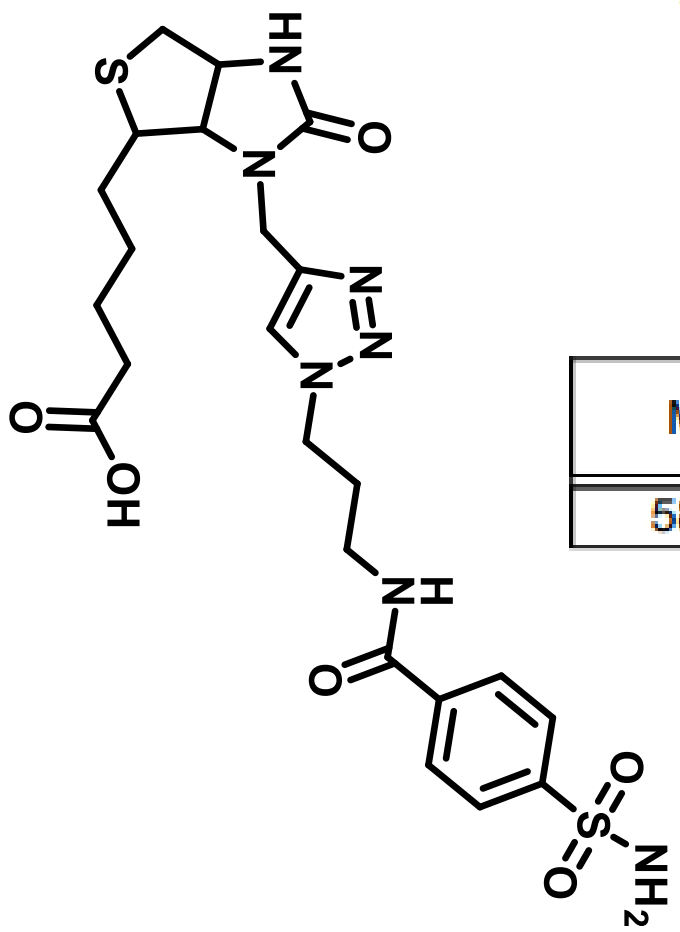

38

Figure S67. HRMS-ESI spectra of compound **38**.

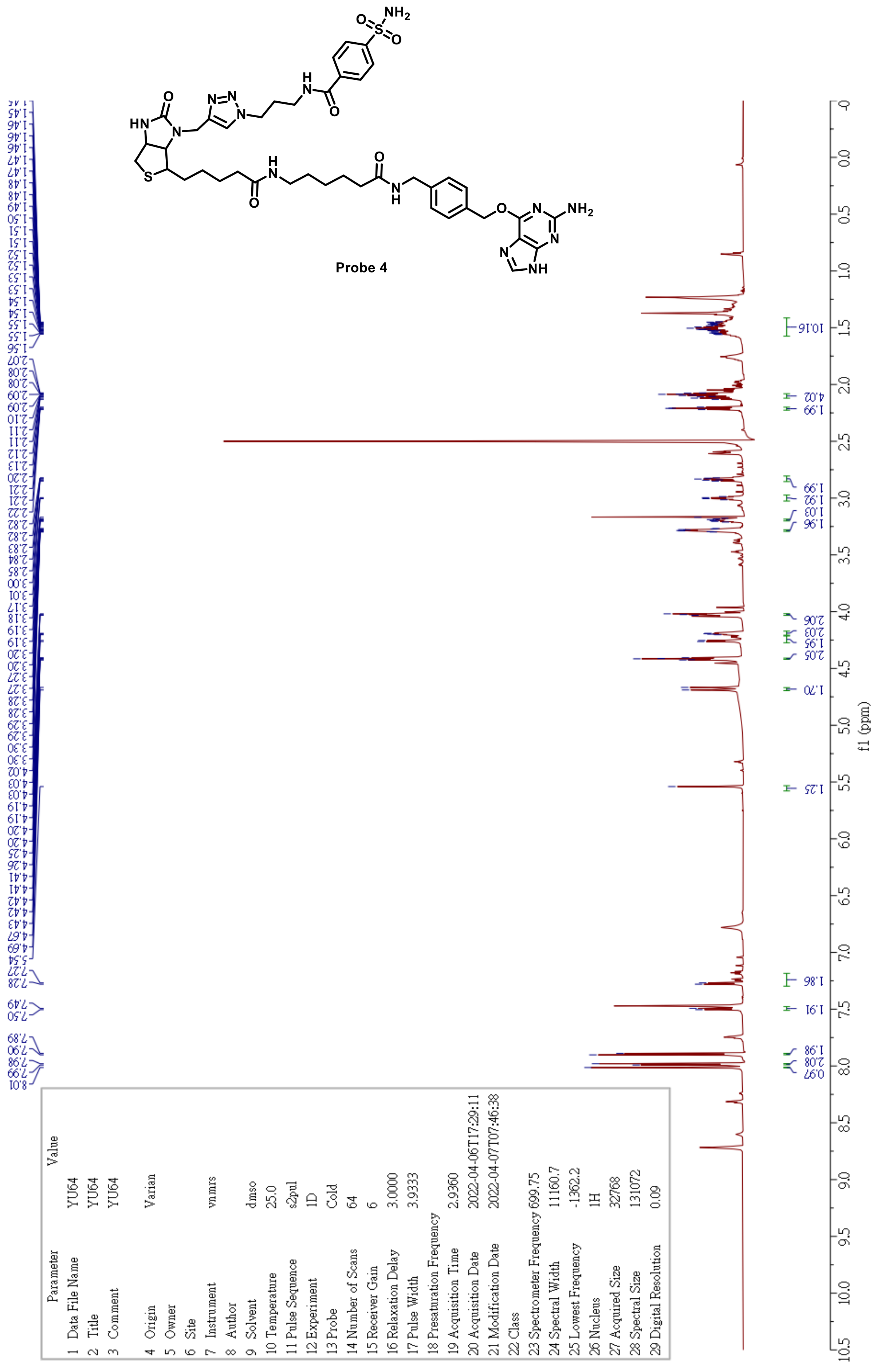

Figure S68. ^{1}H NMR spectra of **Probe 4**.

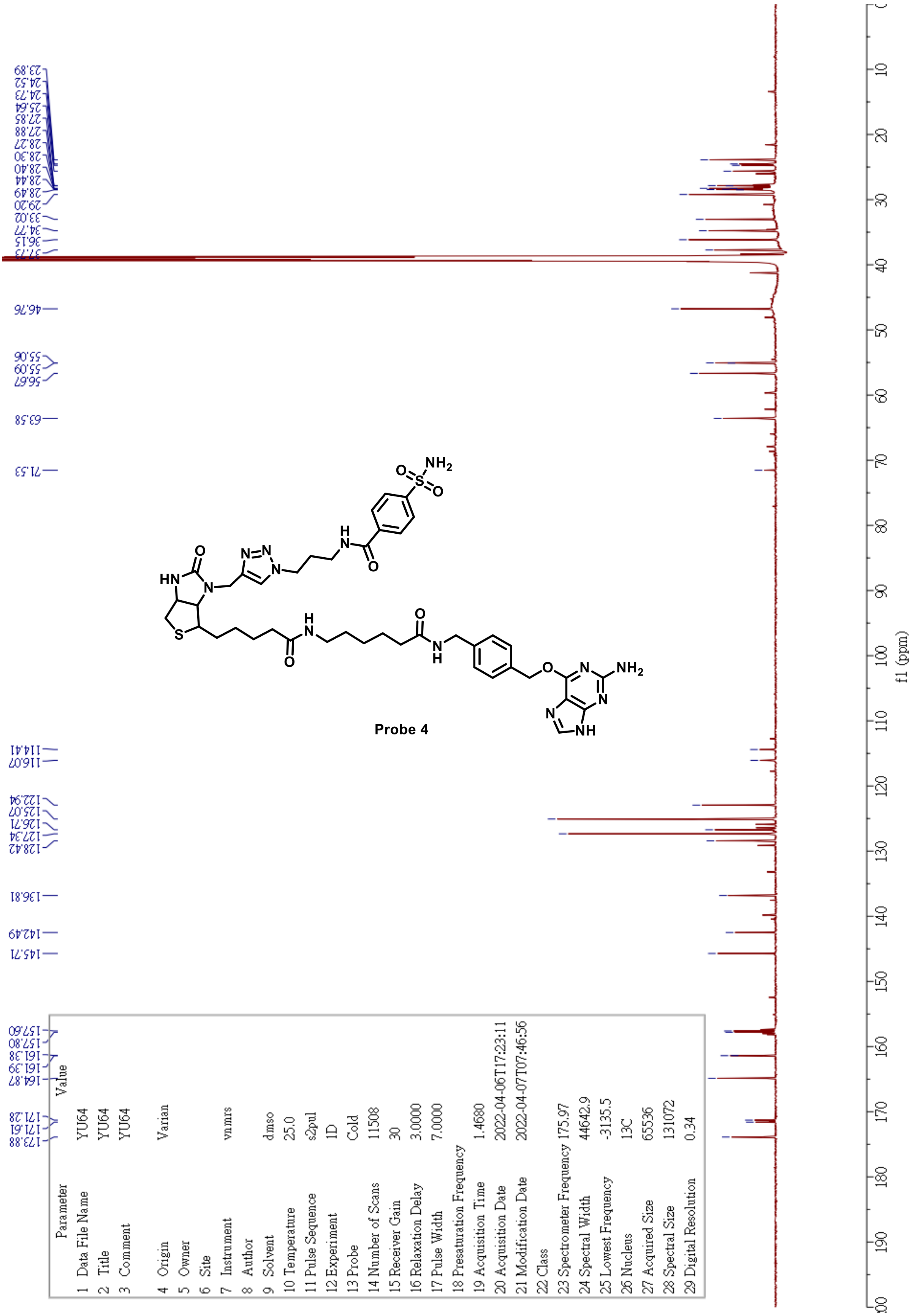

Figure S69. ^{13}C NMR spectra of **Probe 4**.

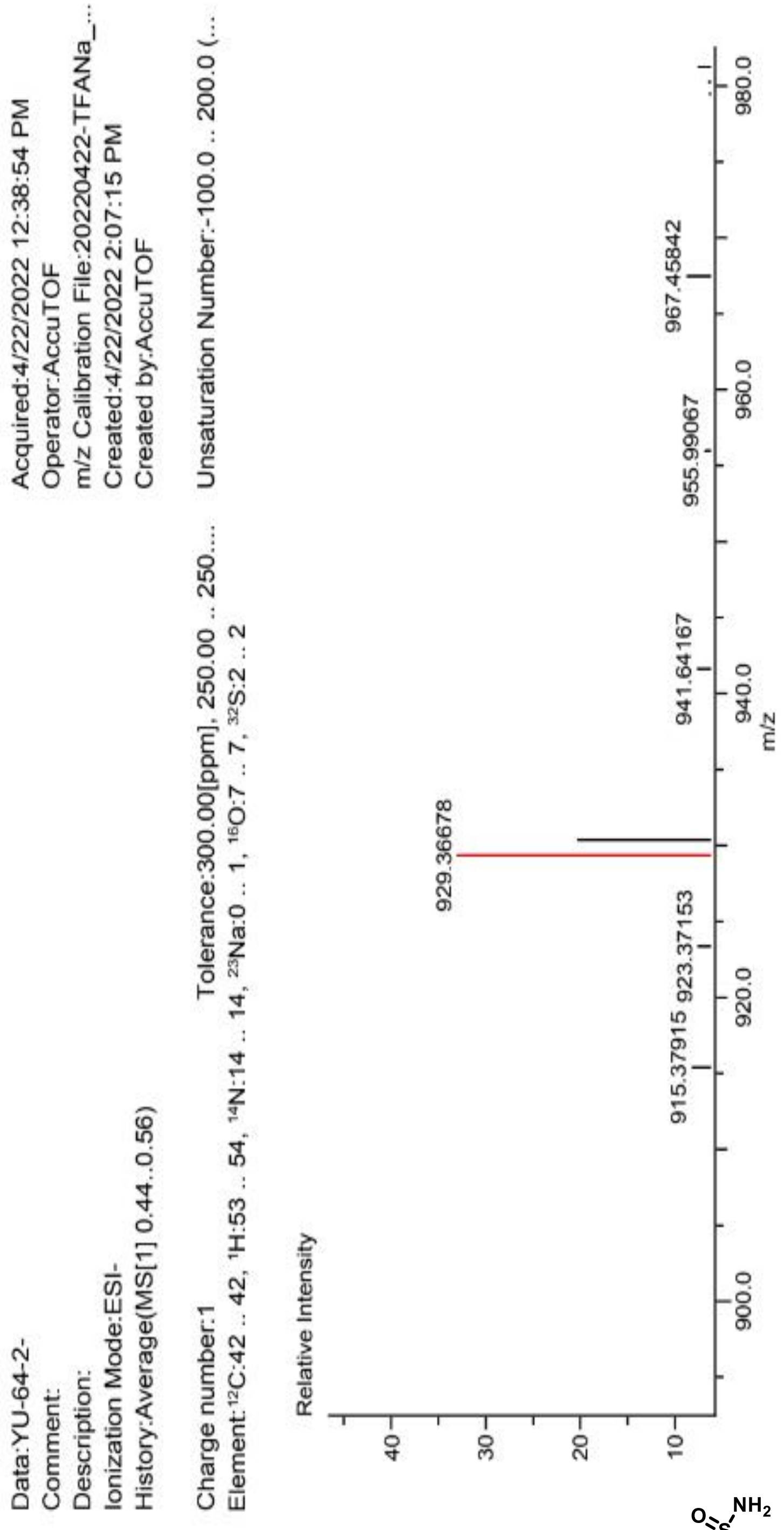

Mass	Intensity	Calc. Mass	Mass Difference [mDa]	Mass Difference [ppm]	Possible Formula
929.36678	1050.66	929.36630	0.48	0.52	$^{12}C_{42}{}^{1}H_{53}{}^{14}N_{14}{}^{16}O_{7}{}^{32}S_{2}$

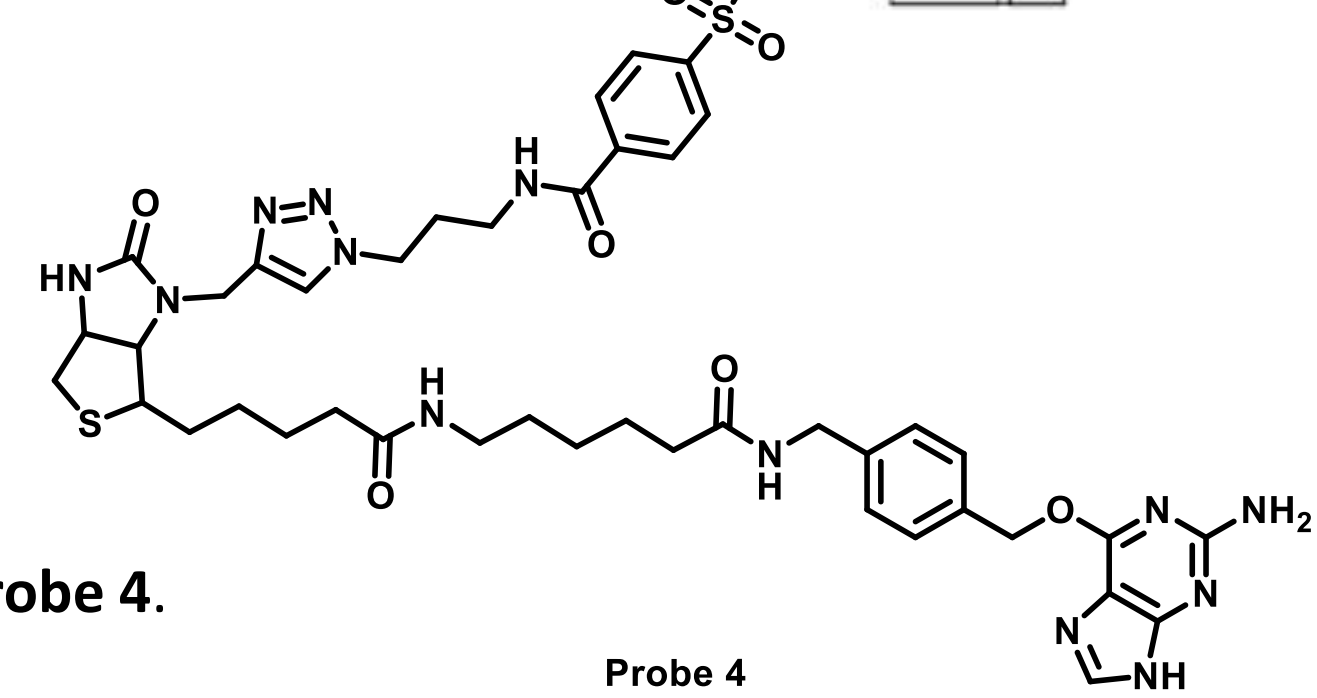

Probe 4

Figure S70. HRMS-ESI spectra of **Probe 4**.

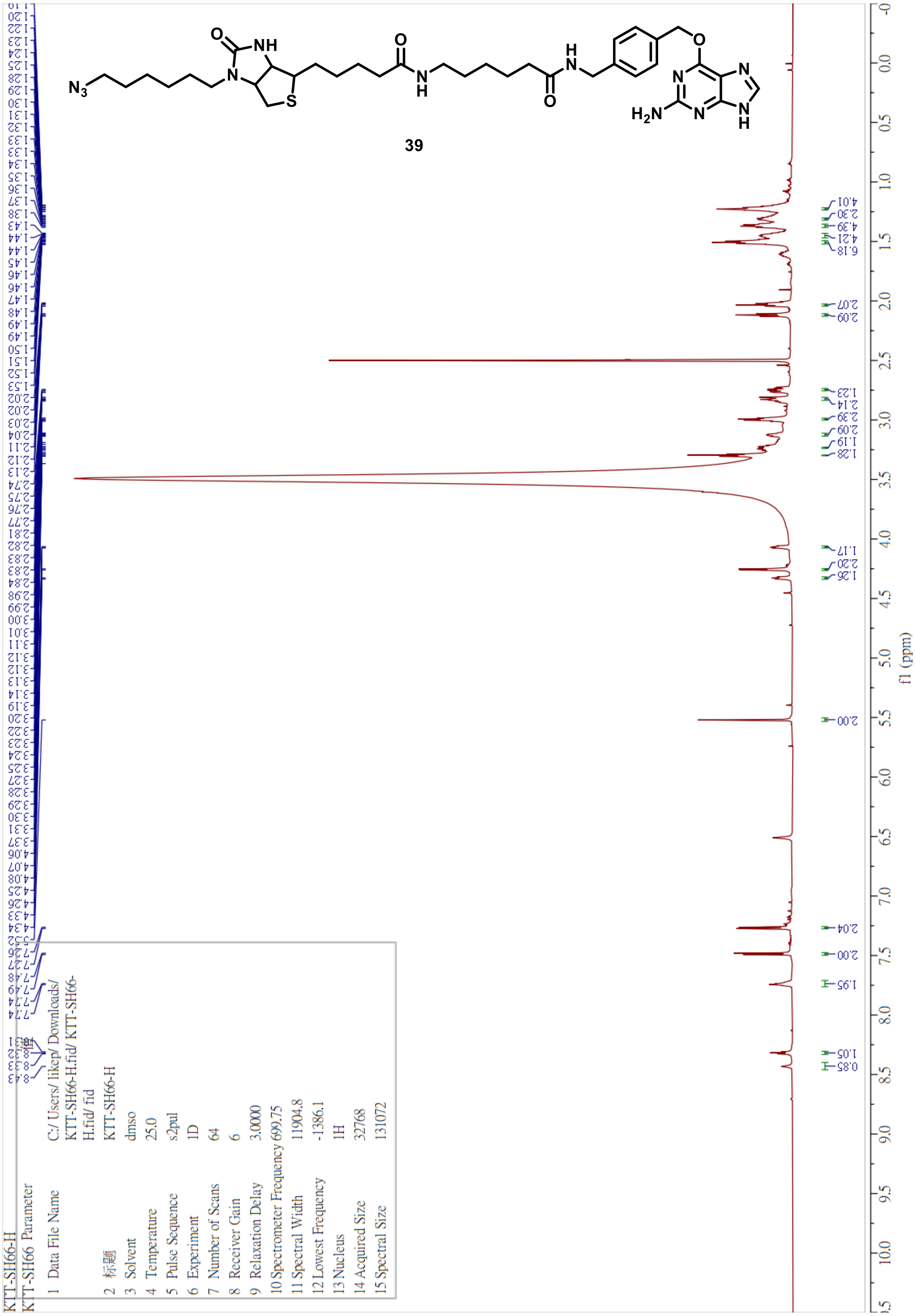

Figure S71. ^{1}H NMR spectra of compound **39**.

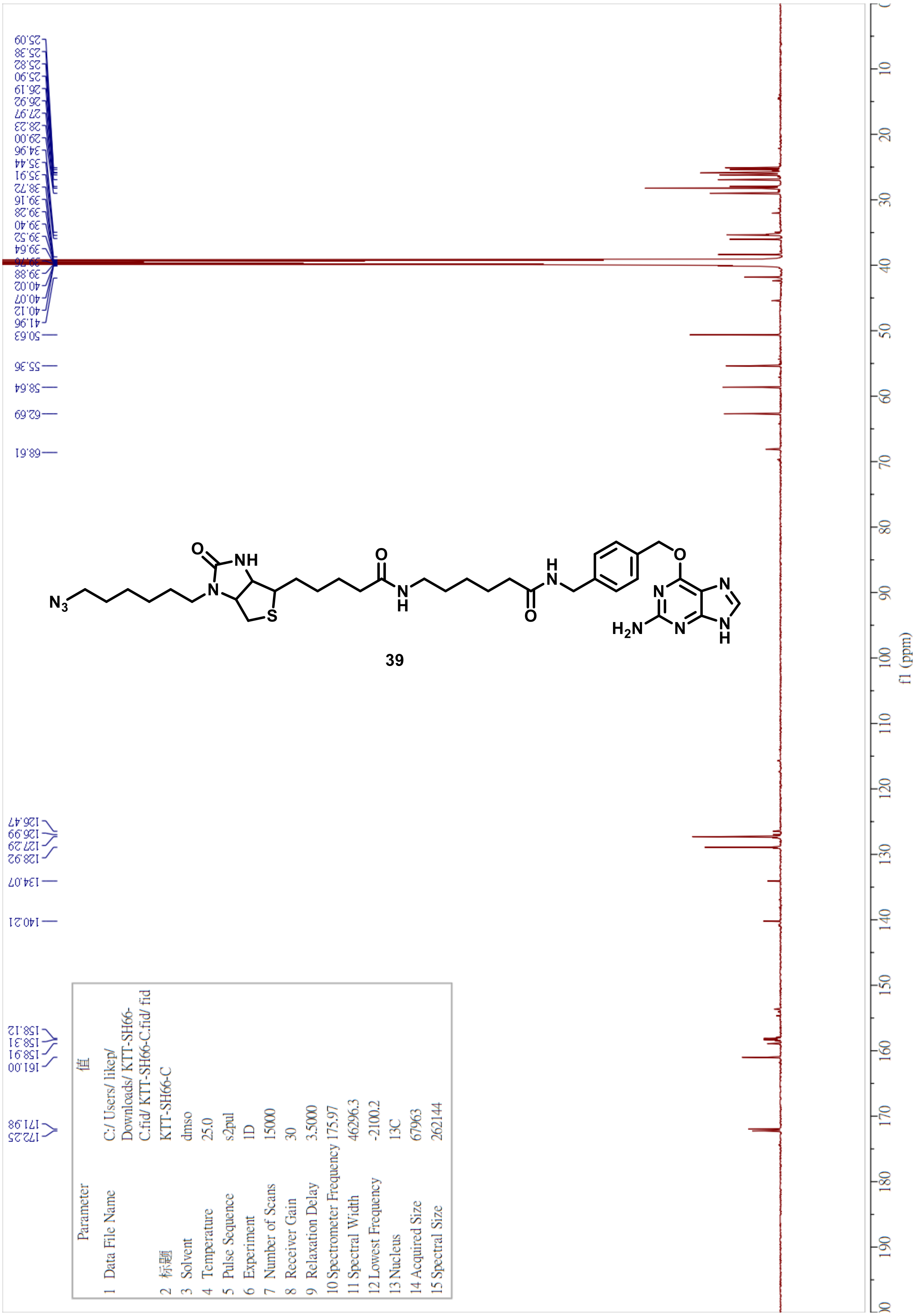

Figure S72. ^{13}C NMR spectra of compound **39**.

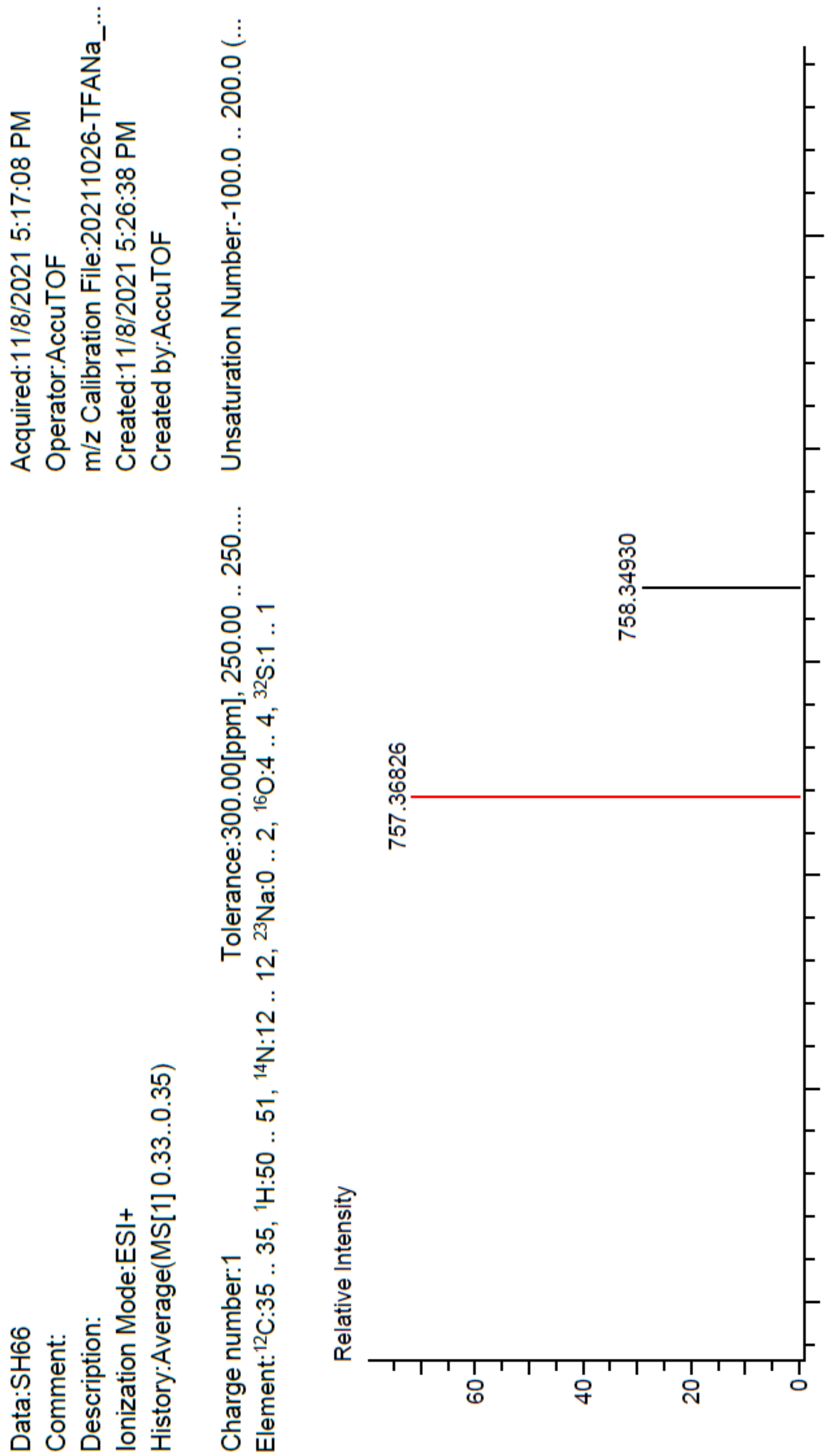

Mass	Intensity	Calc. Mass	Mass Difference [mDa]	Mass Difference [ppm]	Possible Formula
757.36826	2549.34	757.36964	-1.38	-1.82	$^{12}C_{35}{}^{1}H_{50}{}^{14}N_{12}{}^{23}Na_{1}{}^{16}O_{4}{}^{32}S_{1}$

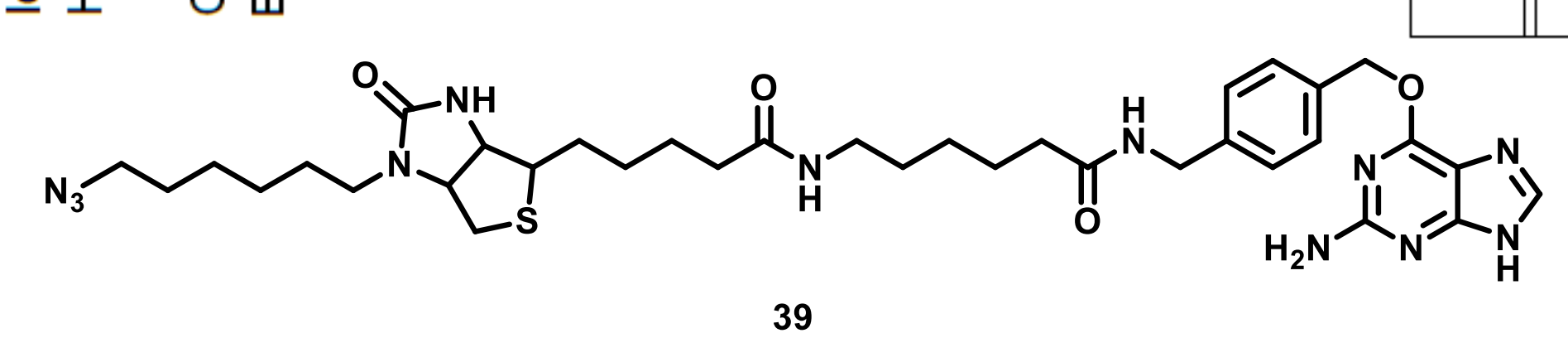

39

Figure S73. HRMS-ESI spectra of compound **39**.

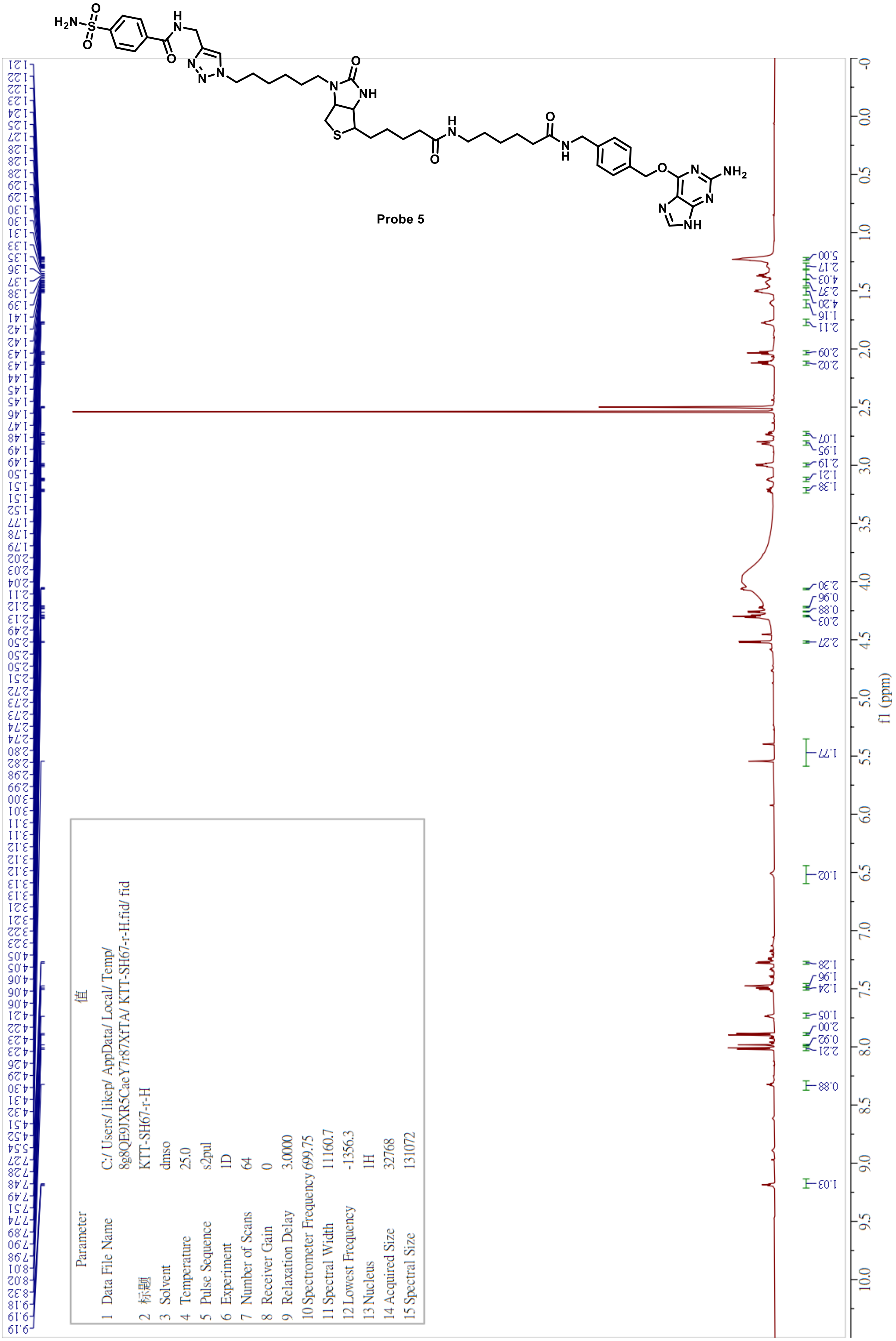

Figure S74. ^{1}H NMR spectra of **Probe 5**.

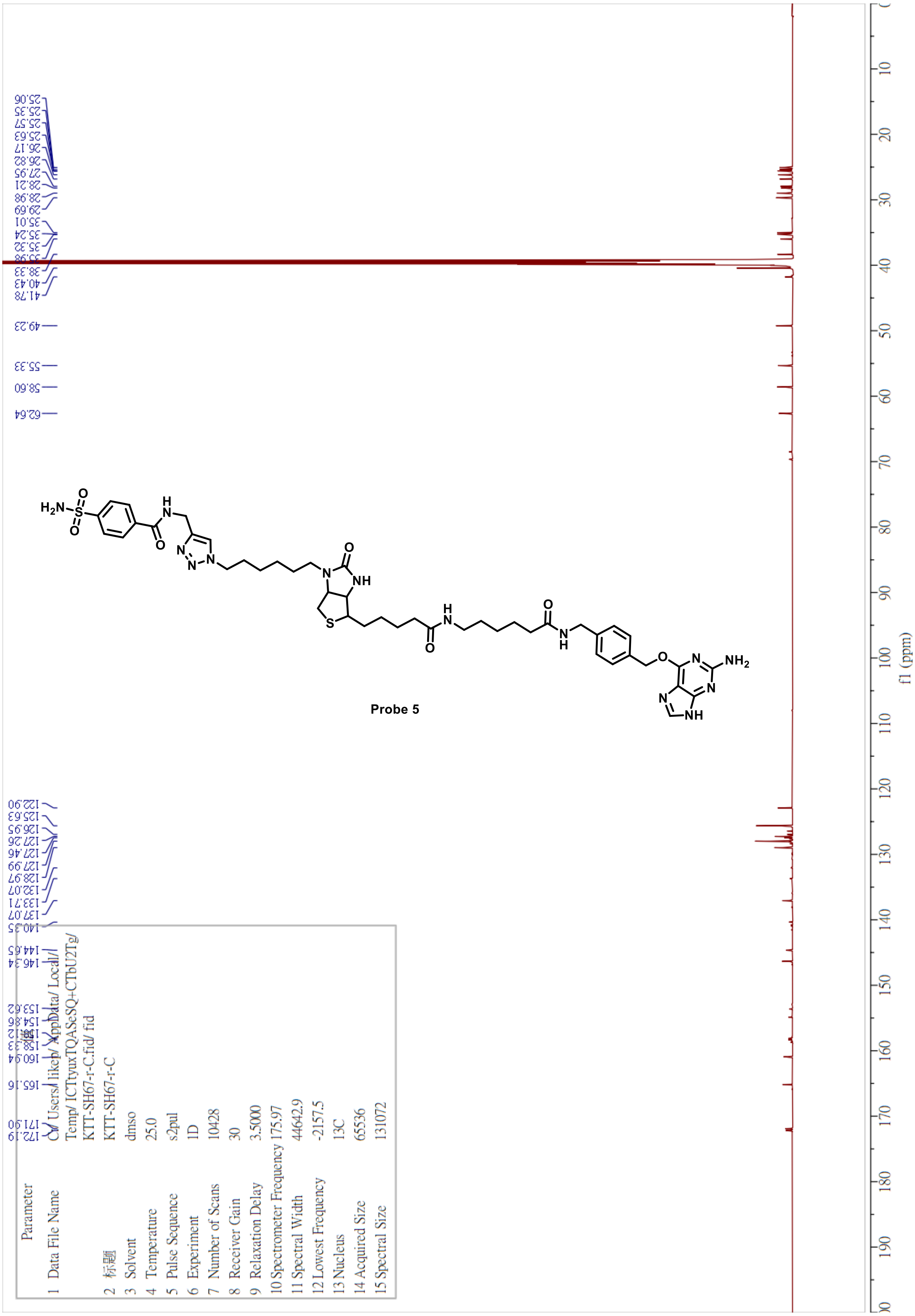

Figure S75. ^{13}C NMR spectra of **Probe 5**.

Data:SH67 -
Comment:
Description:
Ionization Mode:ESI-
History:Average(MS[1] 0.23..0.32)

Acquired:4/21/2022 5:39:53 PM
Operator:AccuTOF
m/z Calibration File:20220216-TFANa_...
Created:4/21/2022 5:48:46 PM
Created by:AccuTOF

Charge number:1
Tolerance:300.00[ppm], 250.00 .. 250....
Unsaturation Number:-100.0 .. 200.0 (...
Element:^{12}C:45 .. 45, ^{1}H:59 .. 60, ^{14}N:14 .. 14, ^{23}Na:0 .. 1, ^{16}O:7 .. 7, ^{32}S:2 .. 2

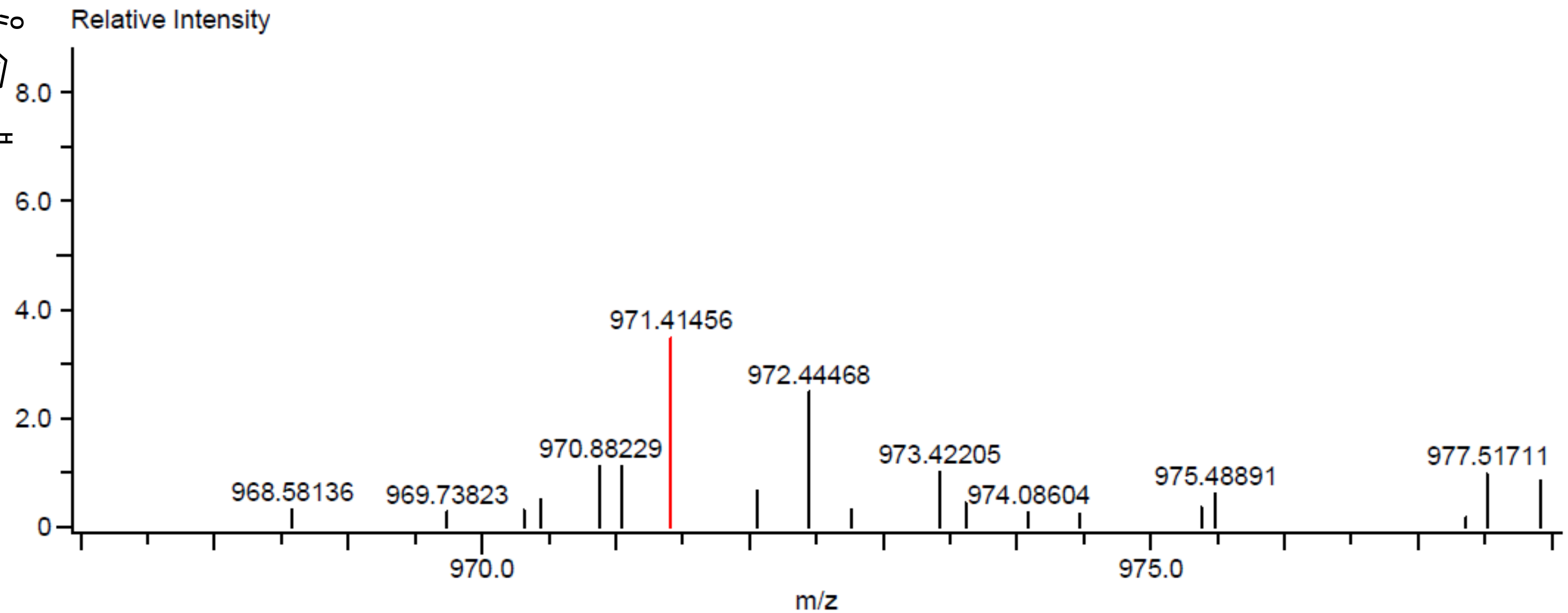

Mass	Intensity	Calc. Mass	Mass Difference [mDa]	Mass Difference [ppm]	Possible Formula
971.41456	701.94	971.41325	1.30	1.34	$^{12}C_{45}{}^{1}H_{59}{}^{14}N_{14}{}^{16}O_{7}{}^{32}S_{2}$

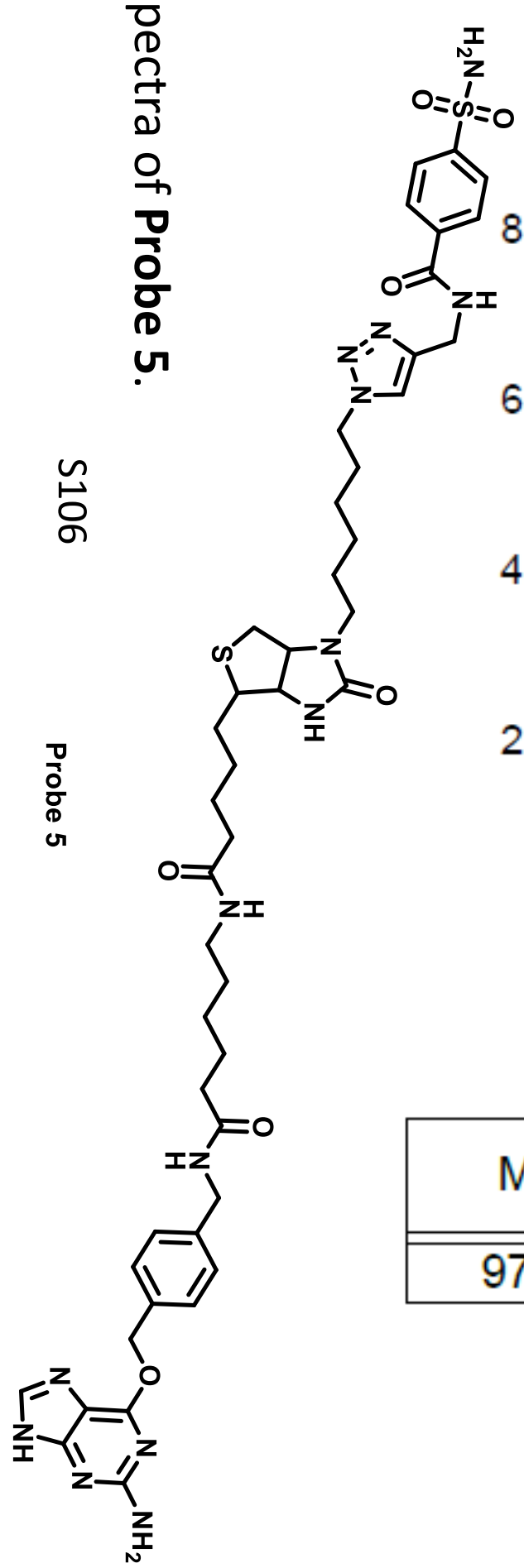

Probe 5

Figure S76. HRMS-ESI spectra of **Probe 5**.

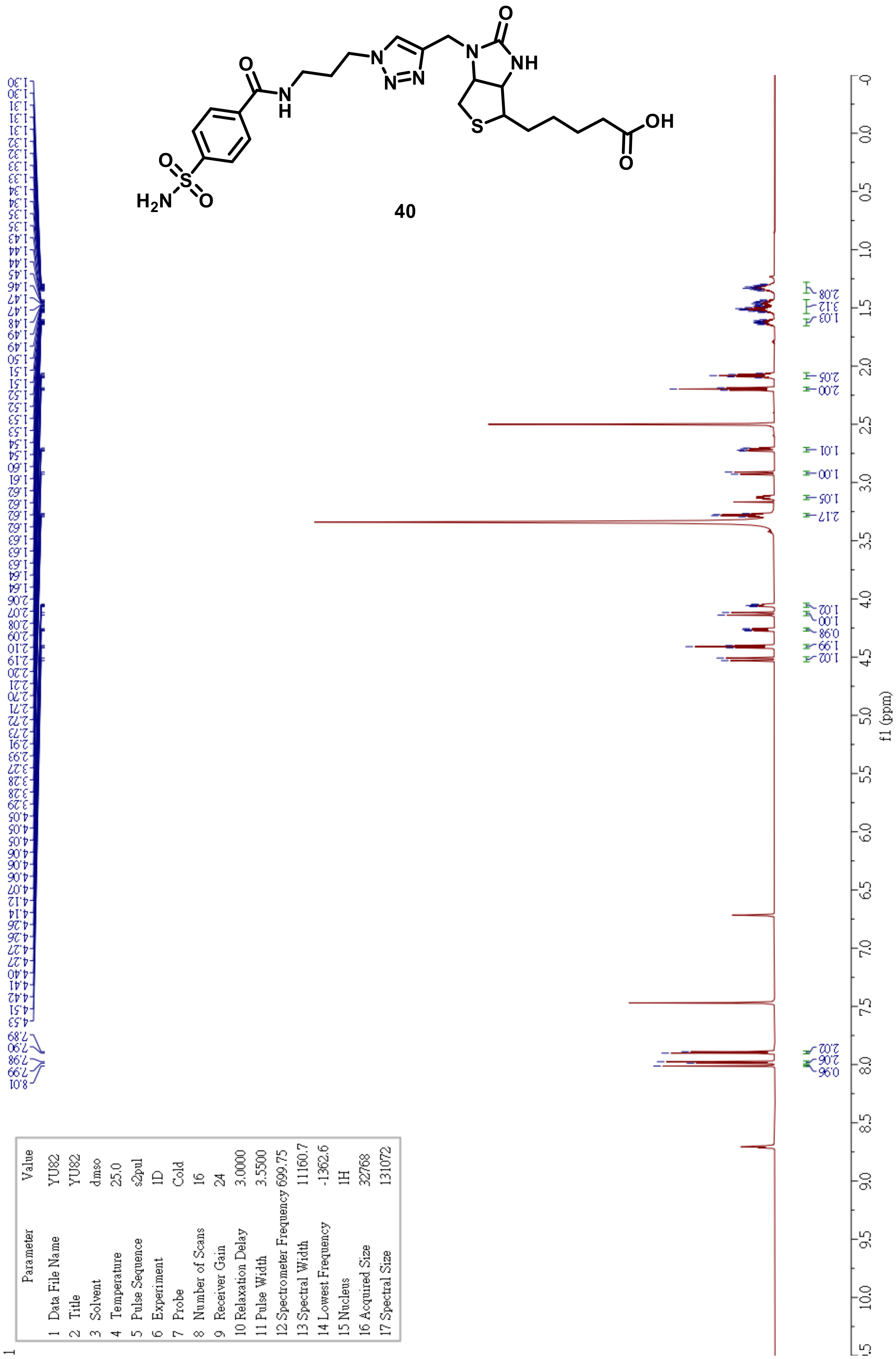

Figure S77. ^{1}H NMR spectra of compound **40**.

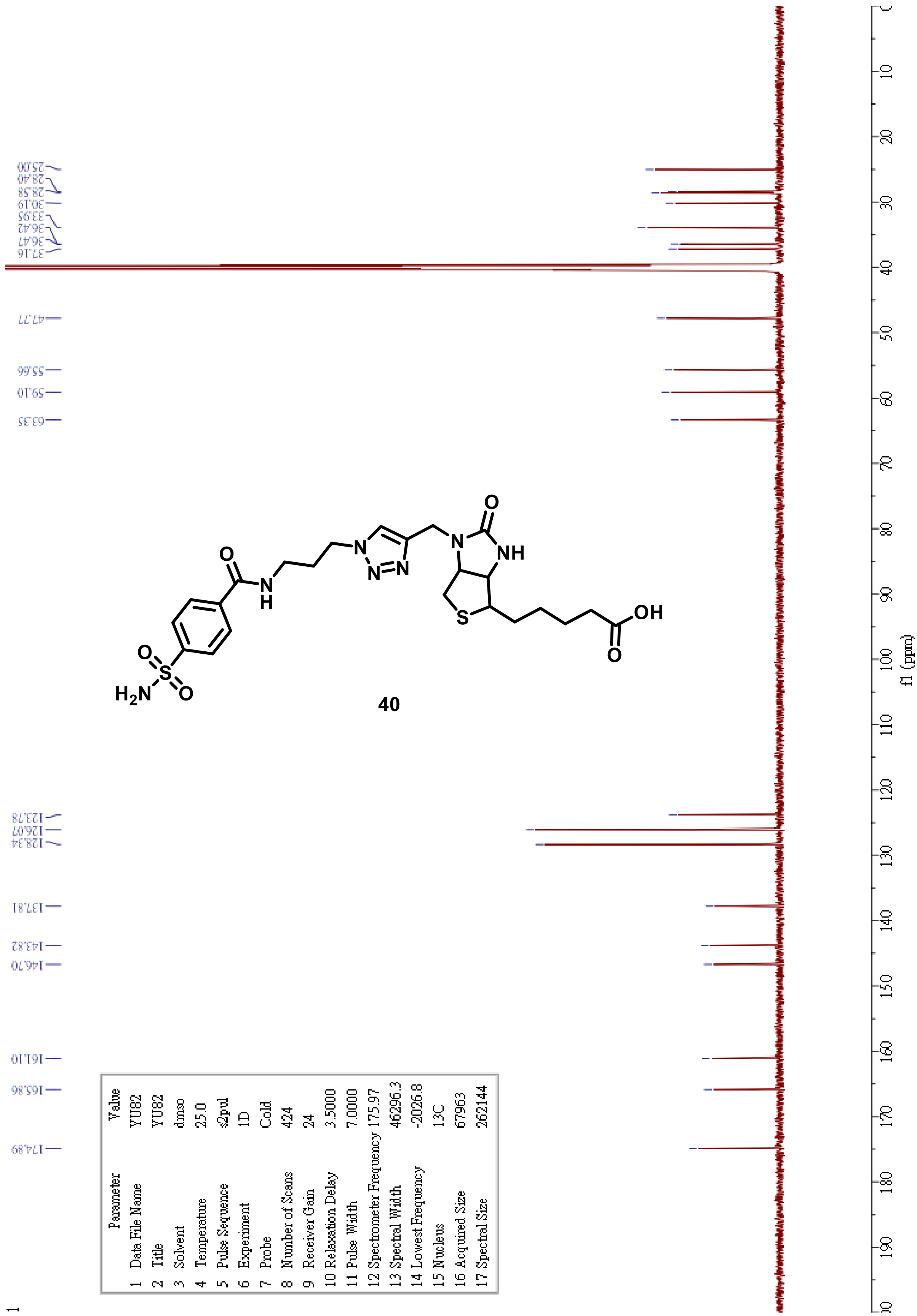

Figure S78. ^{13}C NMR spectra of compound **40**.

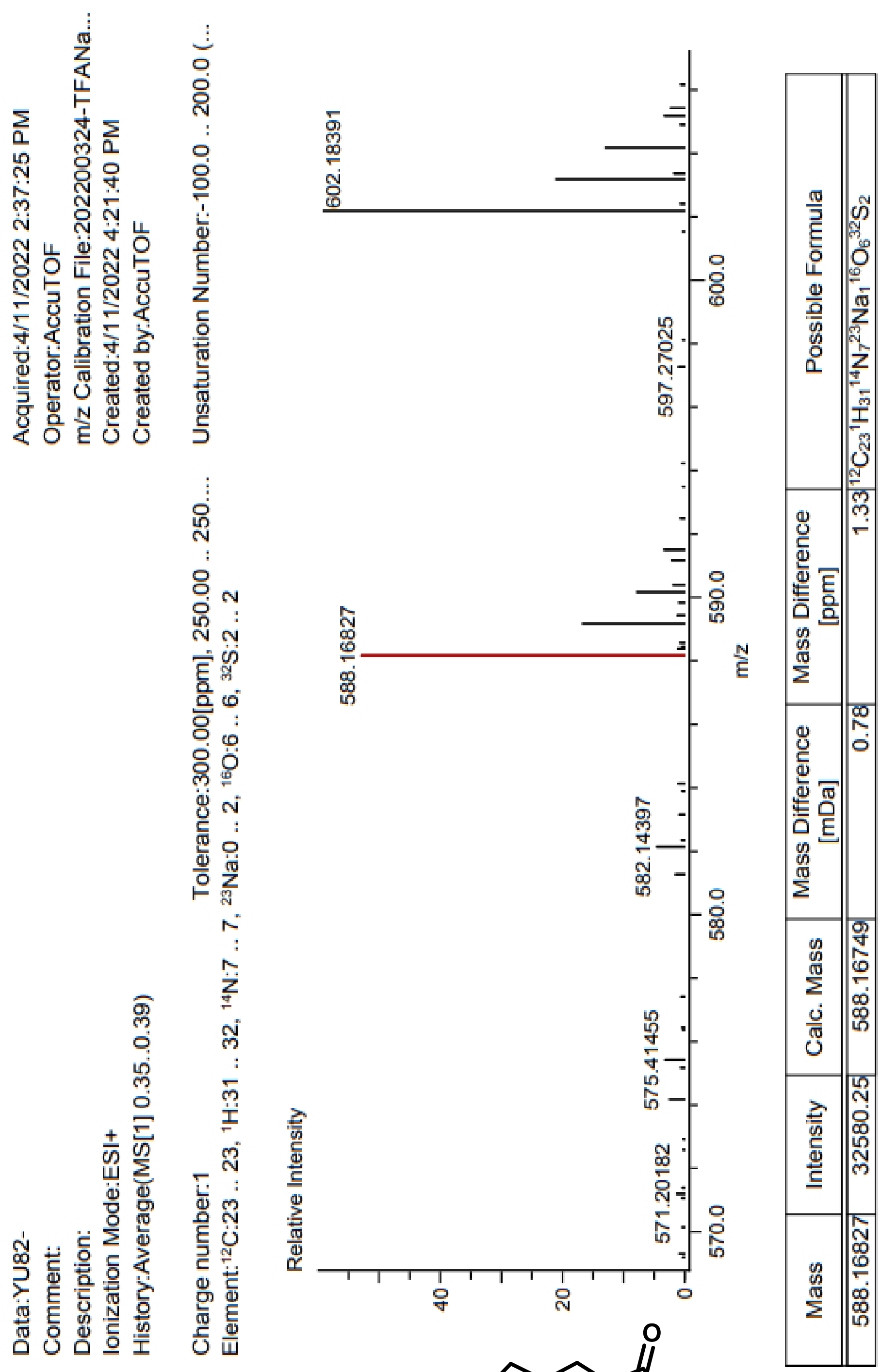

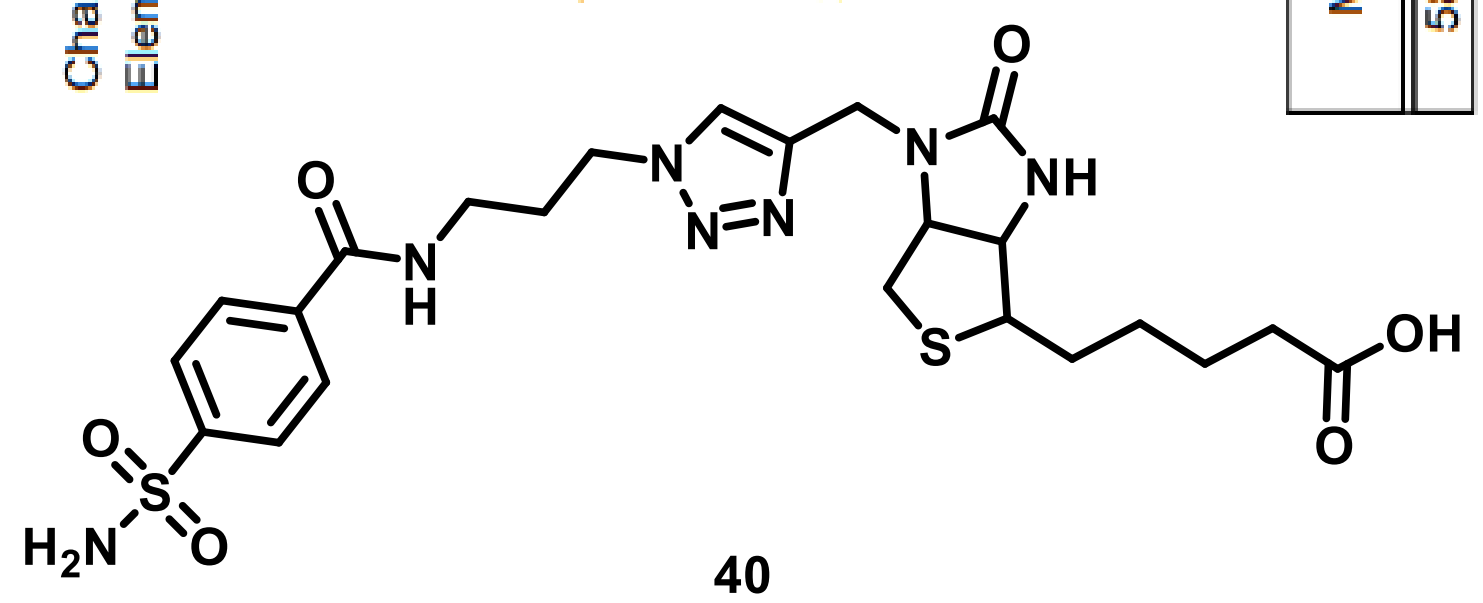

40

Figure S79. HRMS-ESI spectra of compound **40**.

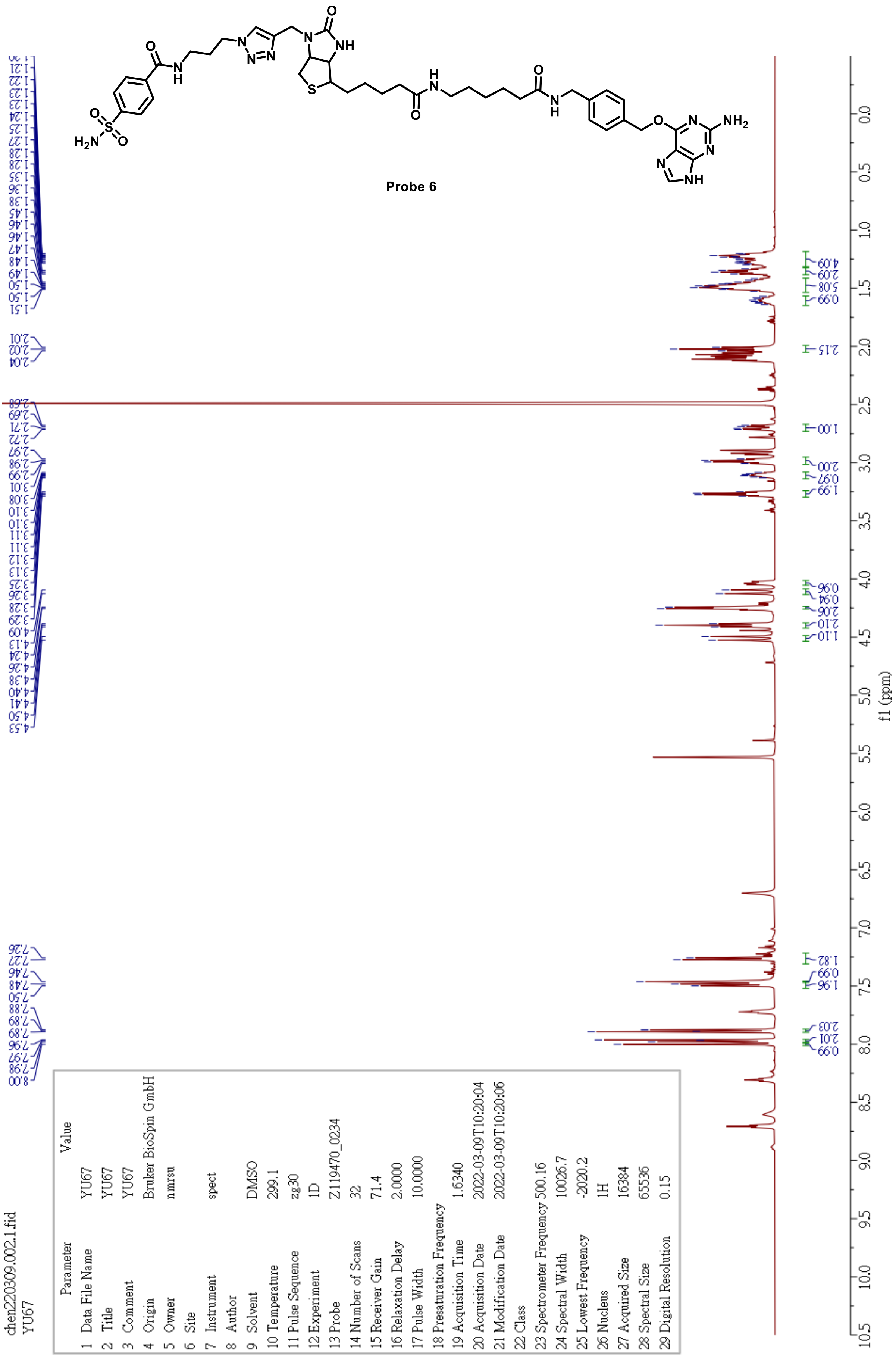

Figure S80. ^{1}H NMR spectra of **Probe 6**.

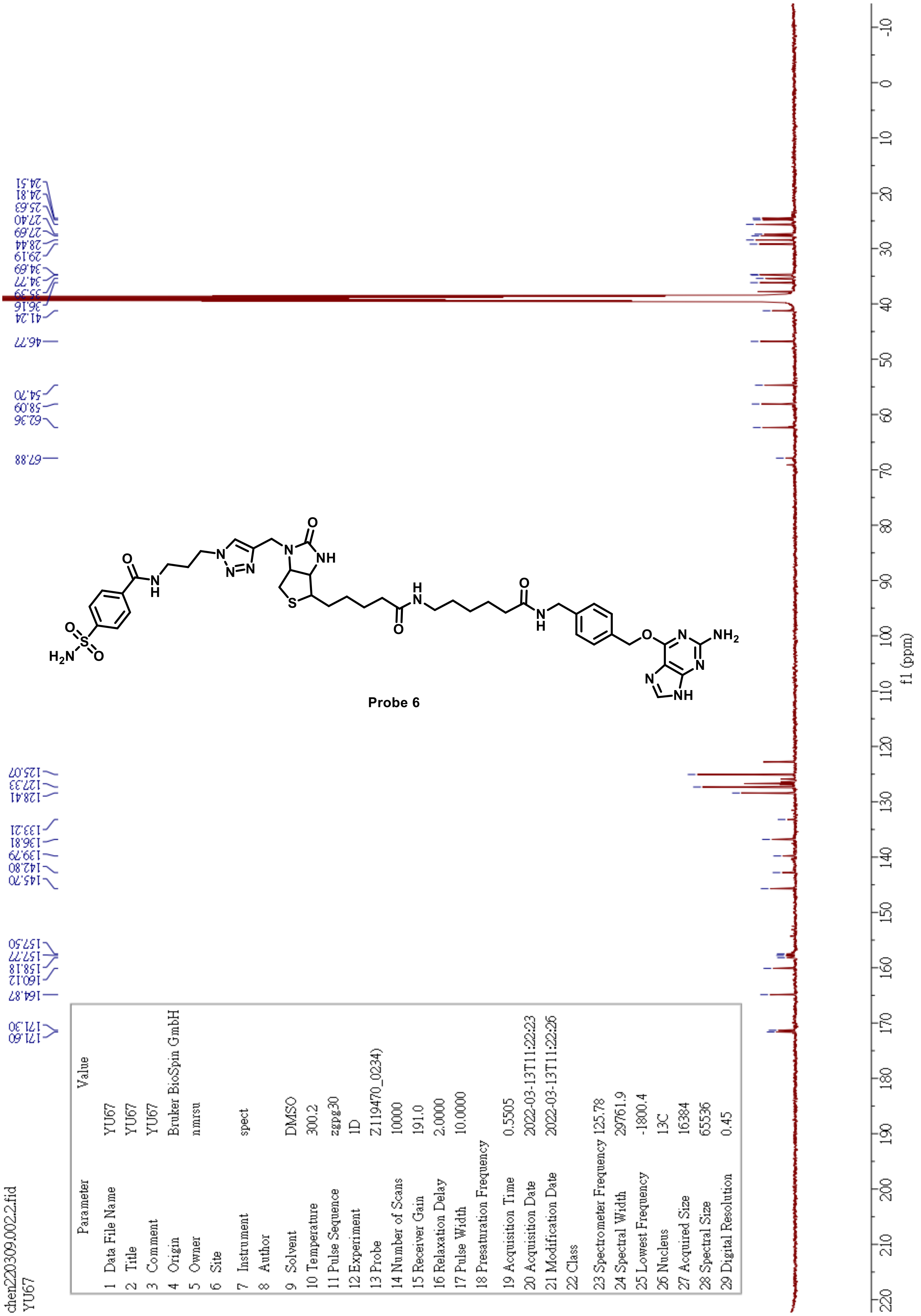

Figure S81. ^{13}C NMR spectra of **Probe 6**.

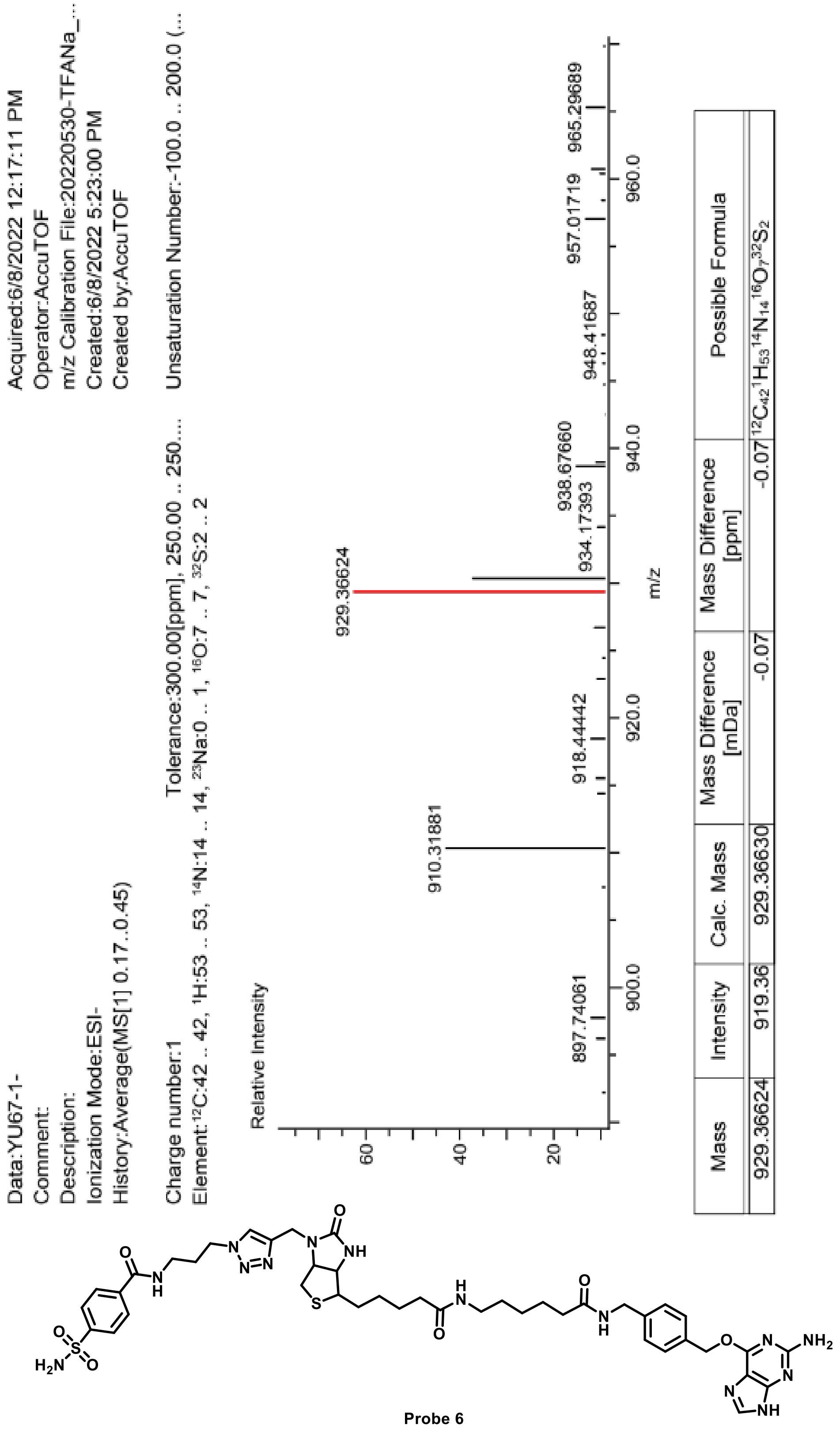

Mass	Intensity	Calc. Mass	Mass Difference [mDa]	Mass Difference [ppm]	Possible Formula
929.36624	919.36	929.36630	-0.07	-0.07	$^{12}C_{42}\,^{1}H_{53}\,^{14}N_{14}\,^{16}O_{7}\,^{32}S_{2}$

Probe 6

Figure S82. HRMS-ESI spectra of **Probe 6**.

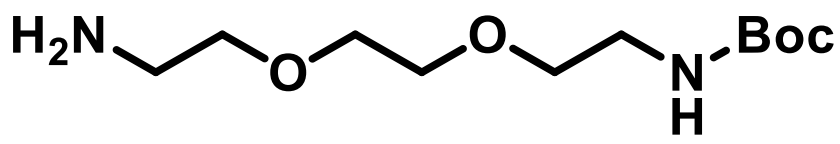

41

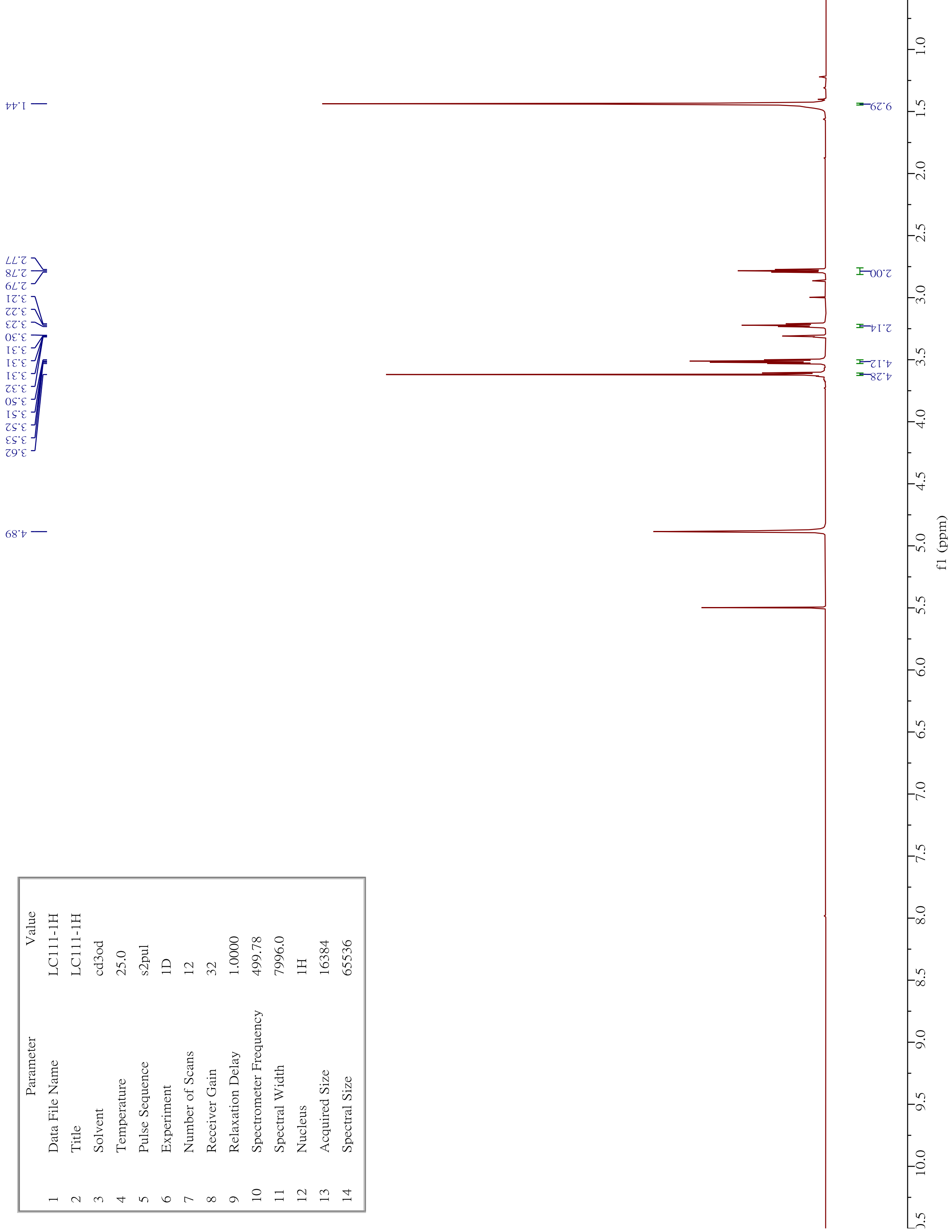

	Parameter	Value
1	Data File Name	LC111-1H
2	Title	LC111-1H
3	Solvent	cd3od
4	Temperature	25.0
5	Pulse Sequence	s2pul
6	Experiment	1D
7	Number of Scans	12
8	Receiver Gain	32
9	Relaxation Delay	1.0000
10	Spectrometer Frequency	499.78
11	Spectral Width	7996.0
12	Nucleus	1H
13	Acquired Size	16384
14	Spectral Size	65536

Figure S83. ^{1}H NMR spectra of compound **41**.

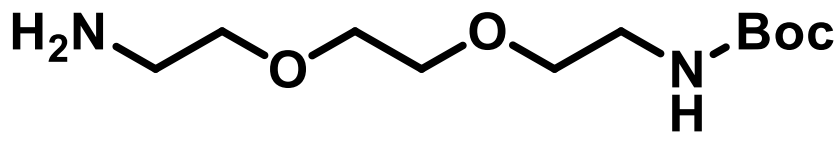

41

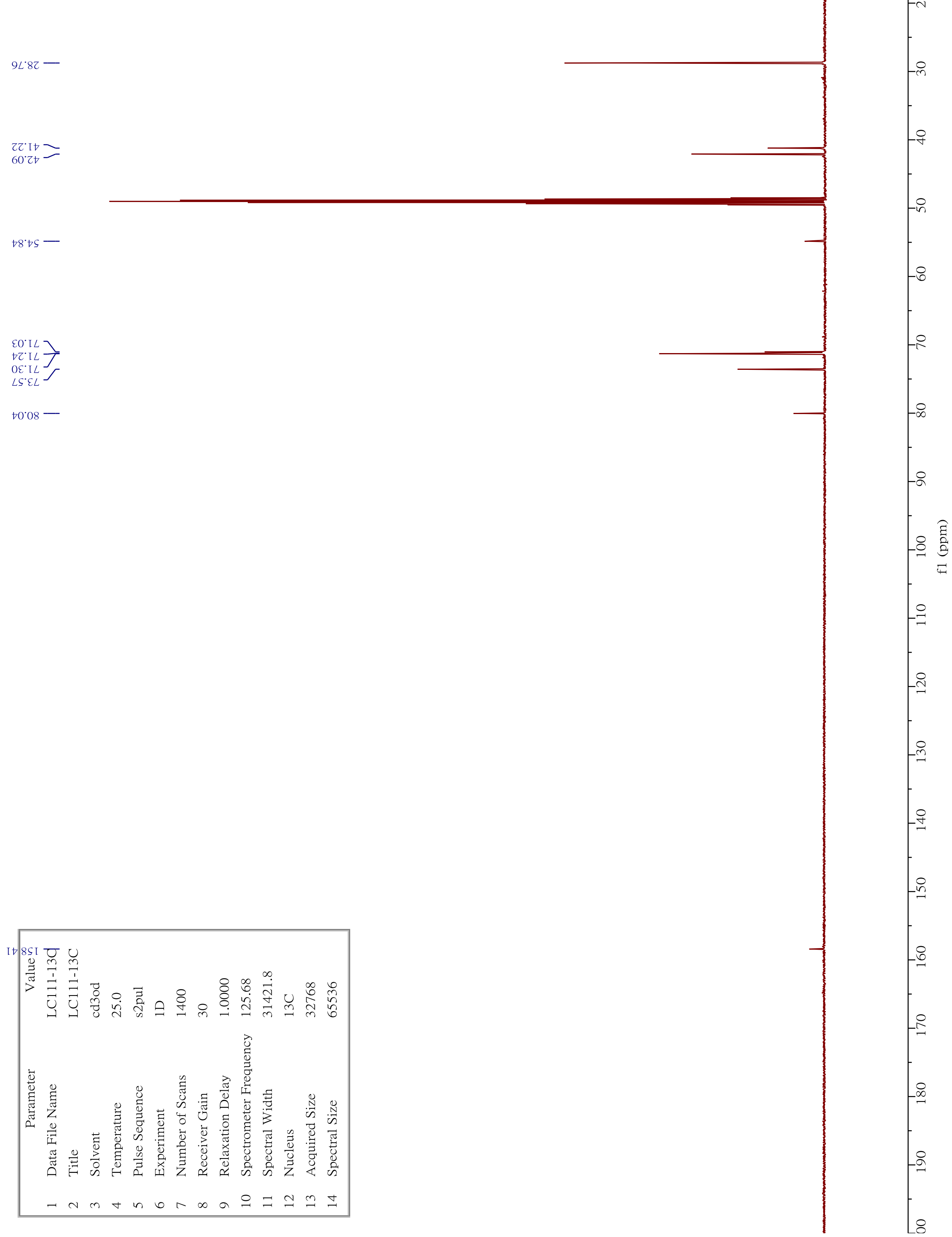

Figure S84. ^{13}C NMR spectra of compound **41**.

Data:LC111
Comment:
Description:
Ionization Mode:ESI+
History:Average(MS[1] 0.39..0.41)

Acquired:2/17/2023 1:26:11 PM
Operator:AccuTOF
m/z Calibration File:20230112-TFANa_...
Created:2/17/2023 5:15:29 PM
Created by:AccuTOF

Charge number:1
Tolerance:200.00[ppm], 250.00 .. 250....
Unsaturation Number:-100.5 .. 200.0 (...
Element:^{12}C:11 .. 11, ^{1}H:20 .. 25, ^{35}Cl:0 .. 1, ^{14}N:2 .. 2, ^{23}Na:0 .. 1, ^{16}O:4 .. 4

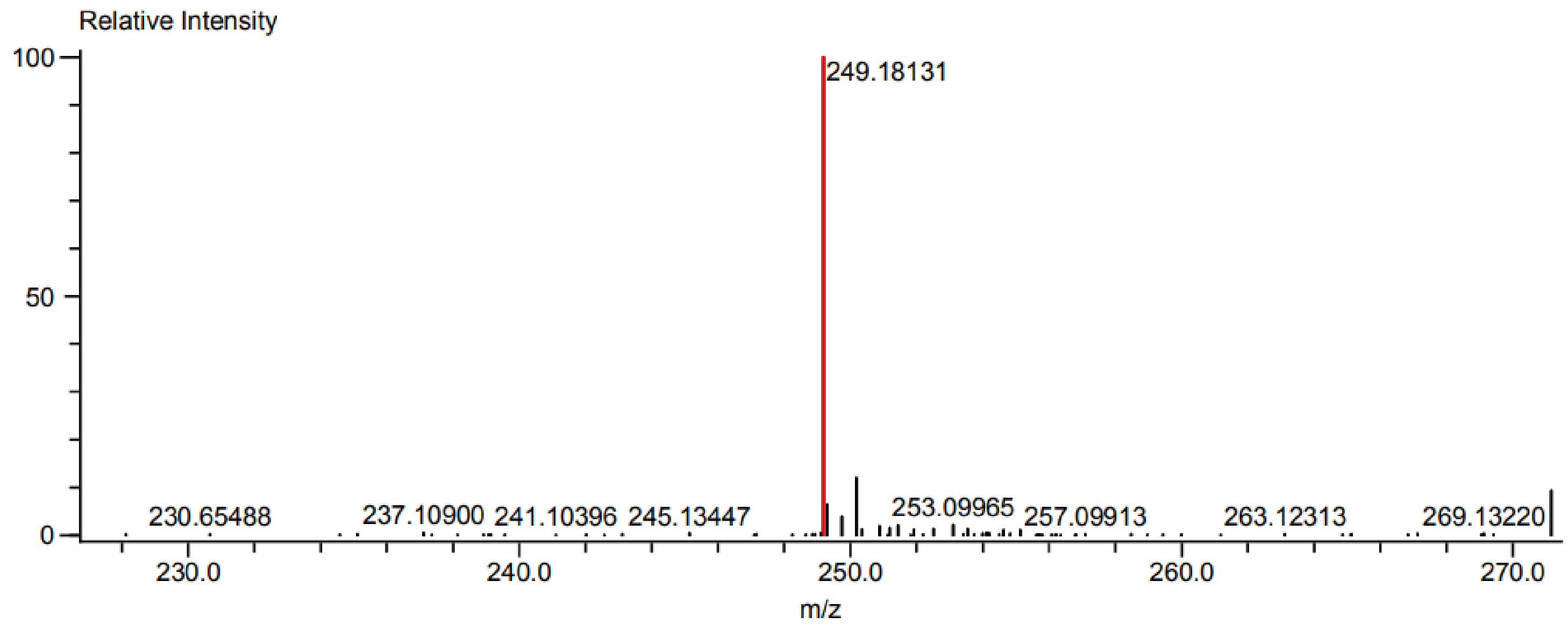

Mass	Intensity	Calc. Mass	Mass Difference [mDa]	Mass Difference [ppm]	Possible Formula
249.18131	471163.50	249.18143	-0.12	-0.49	$^{12}C_{11}{}^{1}H_{25}{}^{14}N_2{}^{16}O_4$

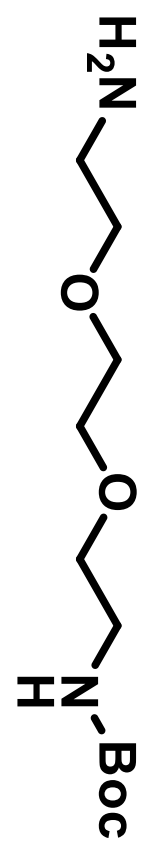

41

Figure S85. HRMS-ESI spectra of compound **41**.

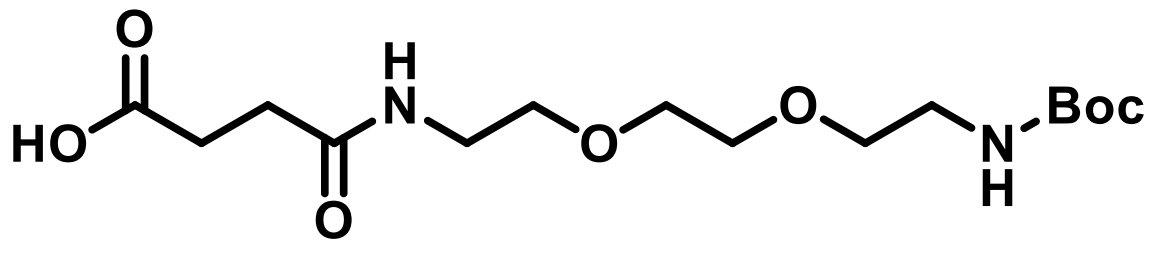

42

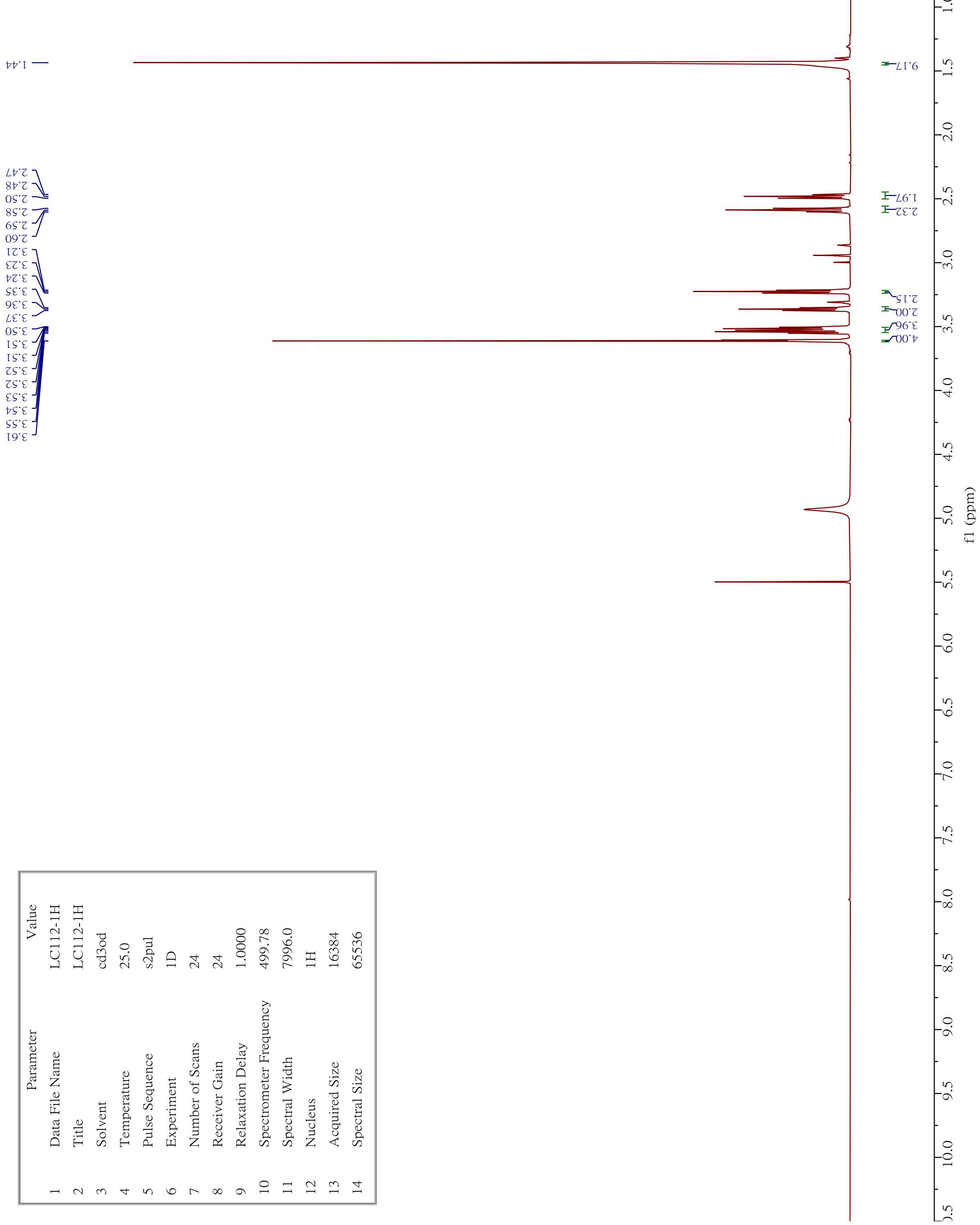

Figure S86. ^{1}H NMR spectra of compound **42**.

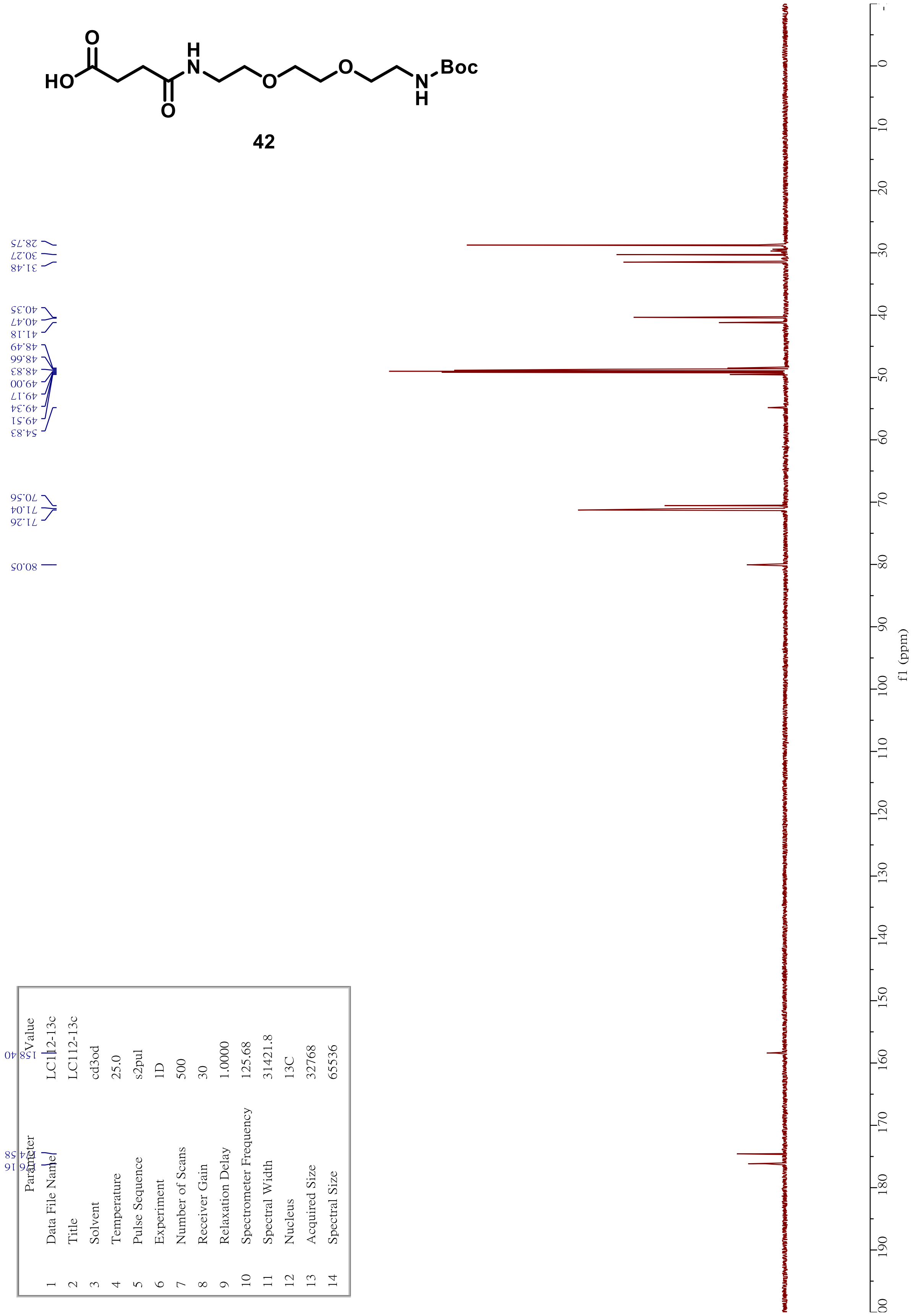

Figure S87. ^{13}C NMR spectra of compound **42**.

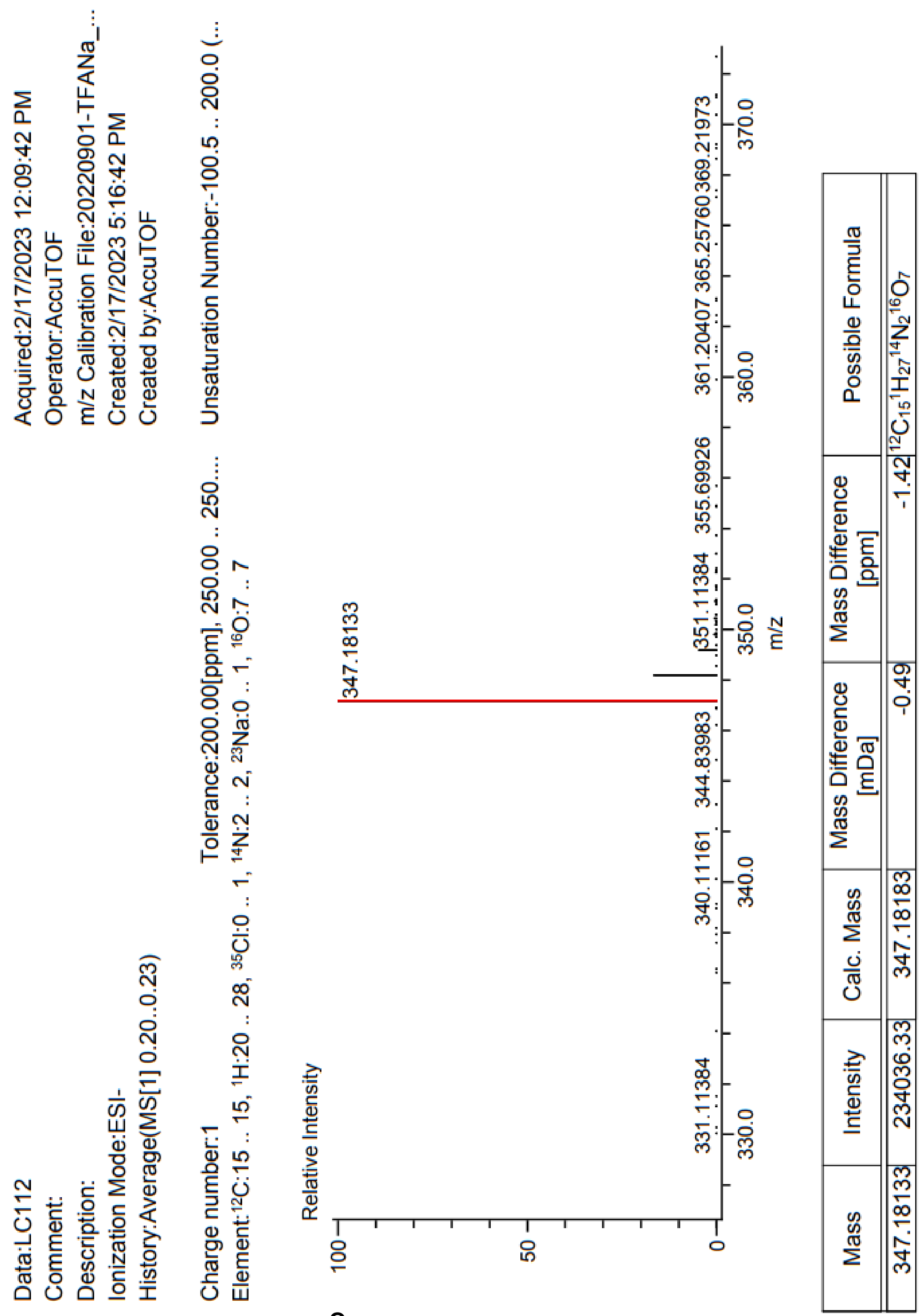

Mass	Intensity	Calc. Mass	Mass Difference [mDa]	Mass Difference [ppm]	Possible Formula
347.18133	234036.33	347.18183	-0.49	-1.42	$^{12}C_{15}{}^{1}H_{27}{}^{14}N_{2}{}^{16}O_{7}$

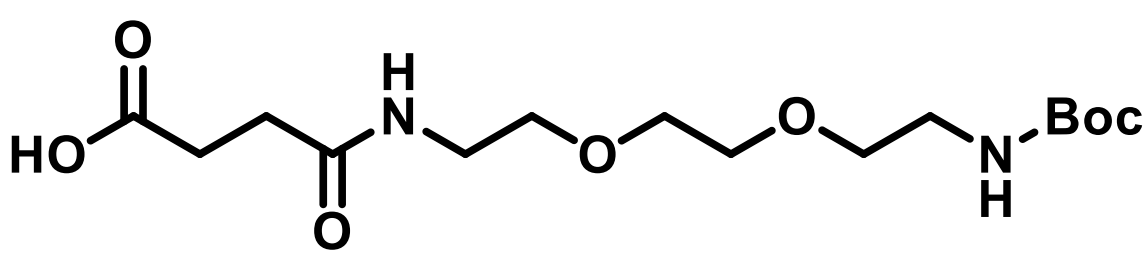

42

Figure S88. HRMS-ESI spectra of compound **42**.

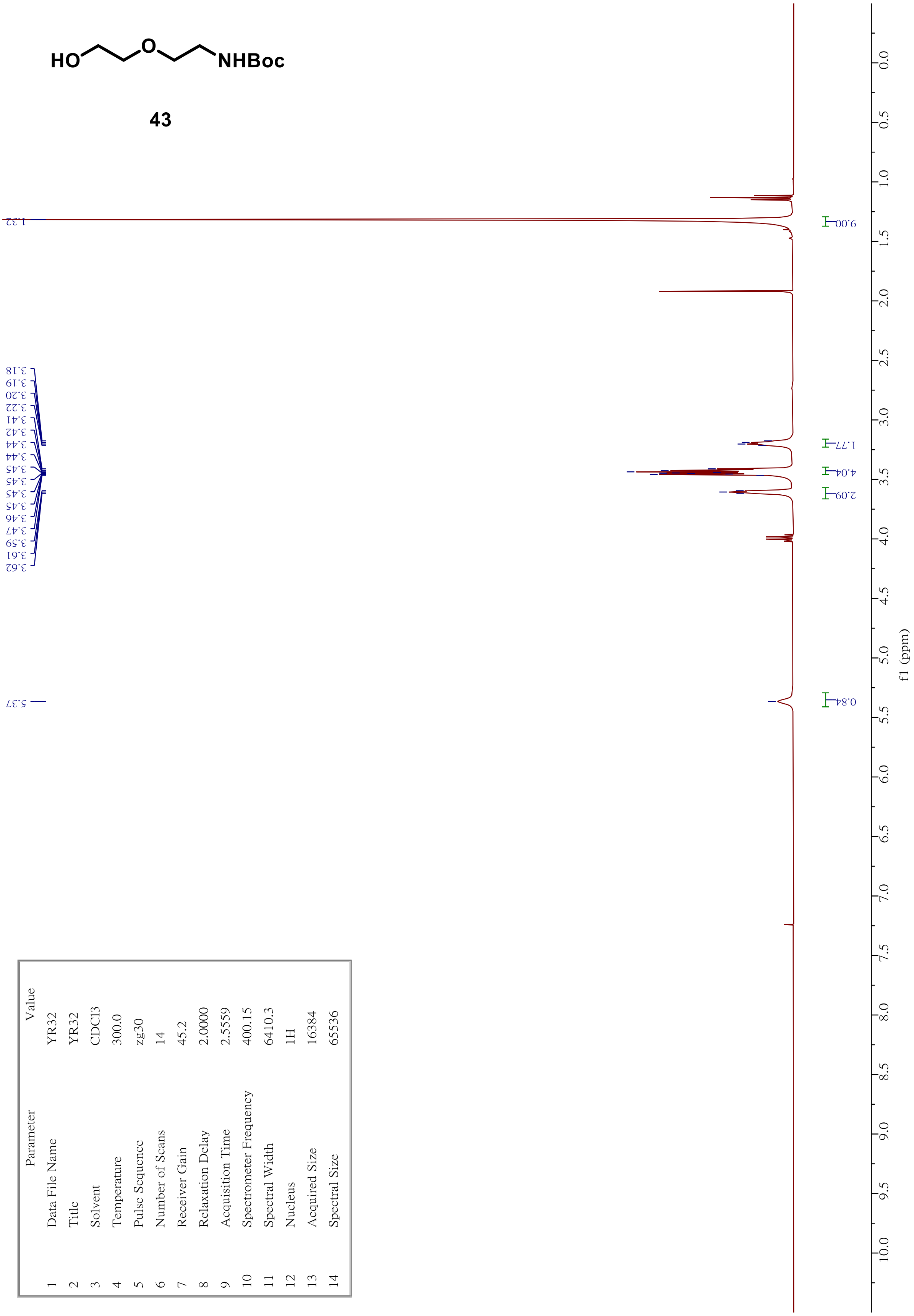

Figure S89. ^{1}H NMR spectra of compound **43**.

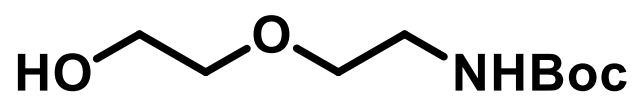

43

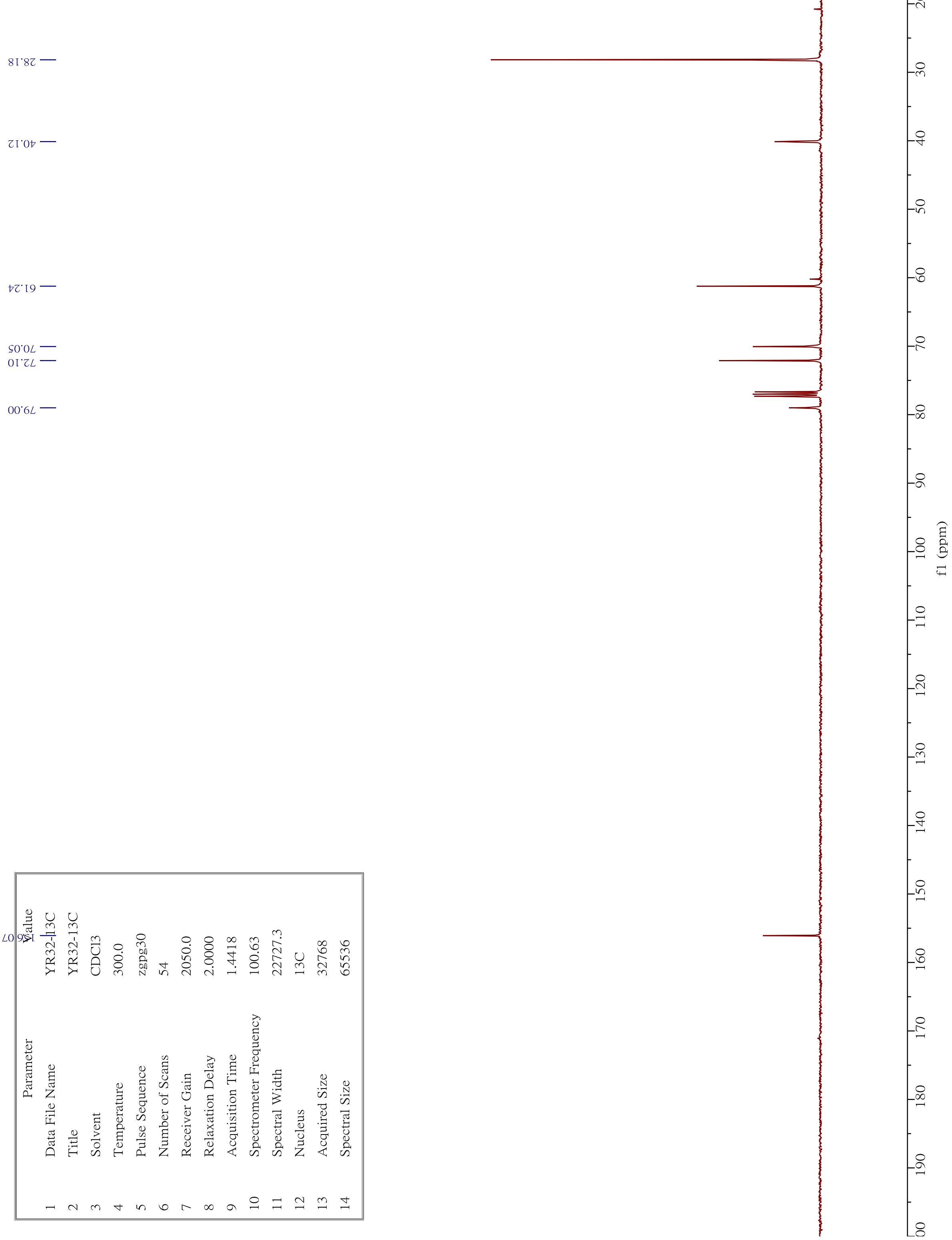

Figure S90. ^{13}C NMR spectra of compound **43**.

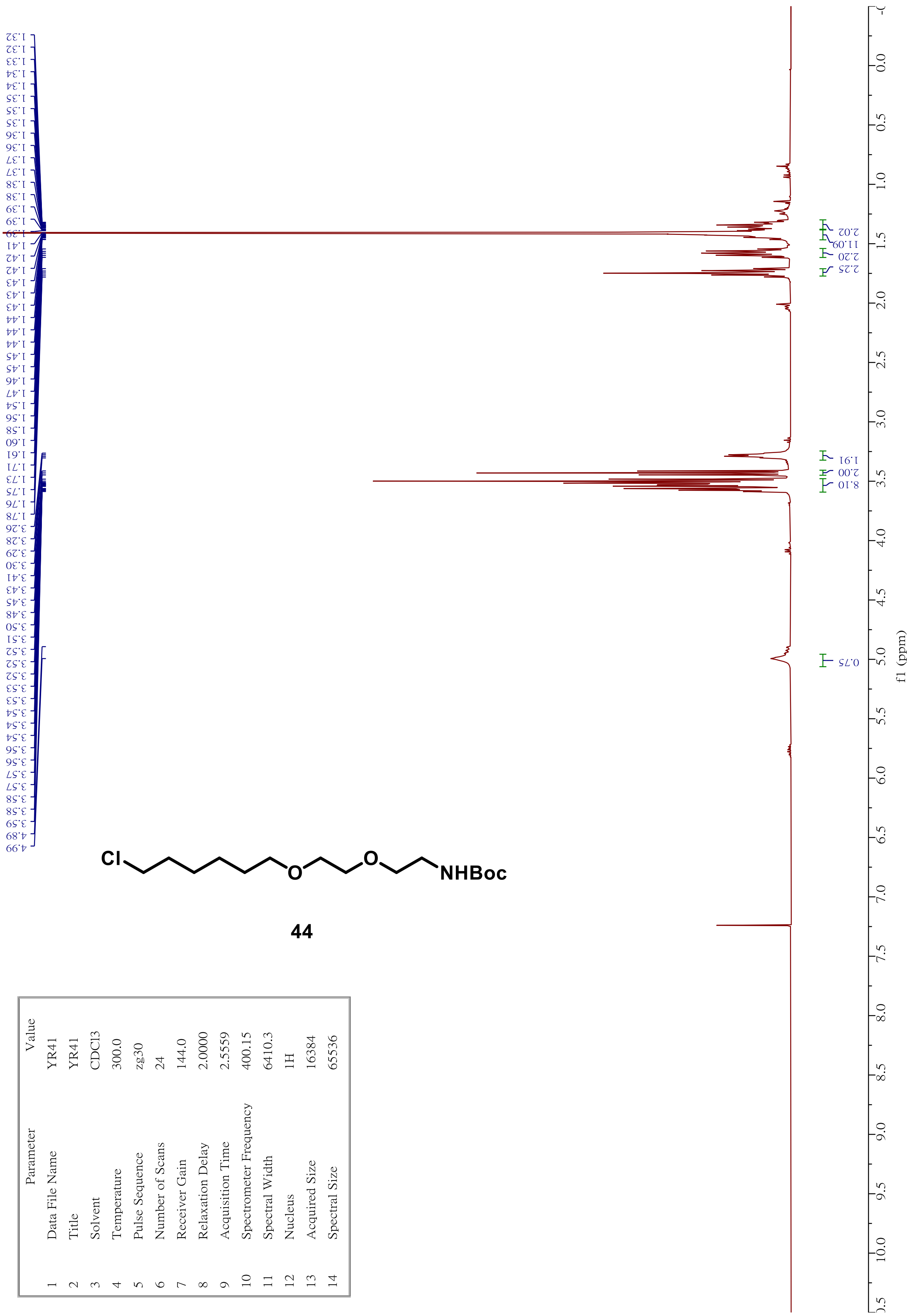

Figure S91. ^{1}H NMR spectra of compound **44**.

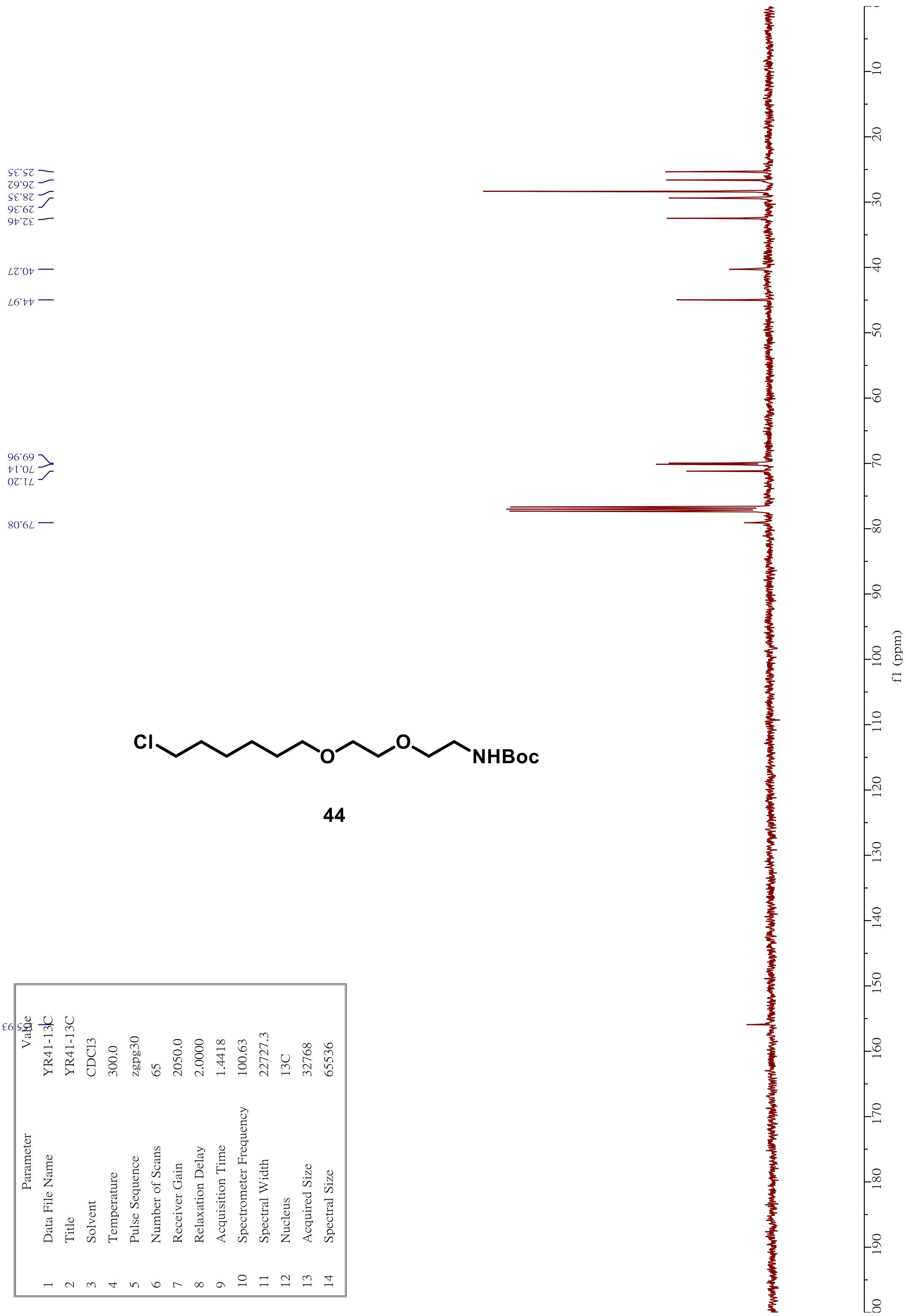

	Parameter	Value
1	Data File Name	YR41-13C
2	Title	YR41-13C
3	Solvent	CDCl3
4	Temperature	300.0
5	Pulse Sequence	zgpg30
6	Number of Scans	65
7	Receiver Gain	2050.0
8	Relaxation Delay	2.0000
9	Acquisition Time	1.4418
10	Spectrometer Frequency	100.63
11	Spectral Width	22727.3
12	Nucleus	13C
13	Acquired Size	32768
14	Spectral Size	65536

Figure S92. ^{13}C NMR spectra of compound **44**.

Data:YR41
Comment:
Description:
Ionization Mode:ESI+
History:Average(MS[1] 0.17..0.20)

Acquired:10/3/2022 5:00:24 PM
Operator:AccuTOF
m/z Calibration File:202200803-TFANa...
Created:10/3/2022 5:04:04 PM
Created by:AccuTOF

Charge number:1
Tolerance:300.00[ppm], 250.00 .. 250....
Unsaturation Number:-100.0 .. 200.0 (...
Element:^{12}C:15 .. 15, ^{1}H:30 .. 31, ^{35}Cl:1 .. 1, ^{14}N:1 .. 1, ^{23}Na:0 .. 2, ^{16}O:4 .. 4

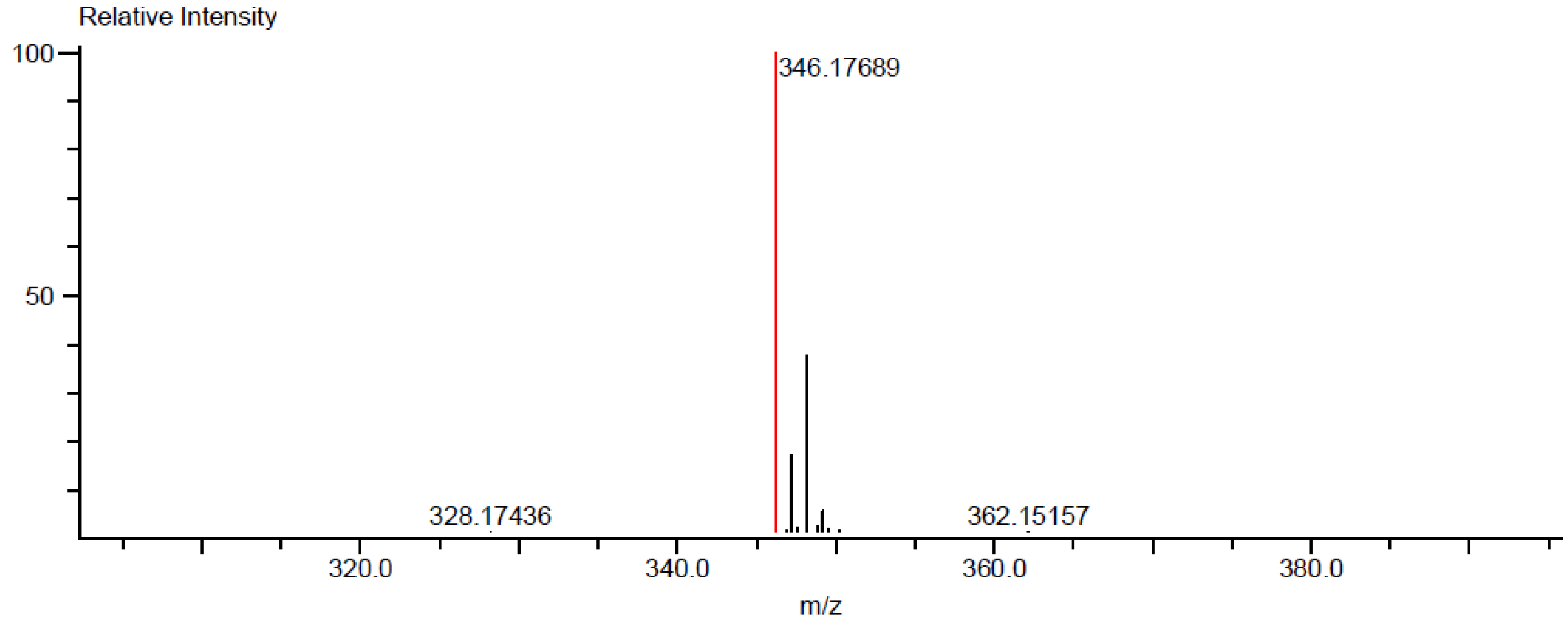

Mass	Intensity	Calc. Mass	Mass Difference [mDa]	Mass Difference [ppm]	Possible Formula
346.17689	369341.75	346.17610	0.79	2.27	$^{12}C_{15}{}^{1}H_{30}{}^{35}Cl_{1}{}^{14}N_{1}{}^{23}Na_{1}{}^{16}O_{4}$

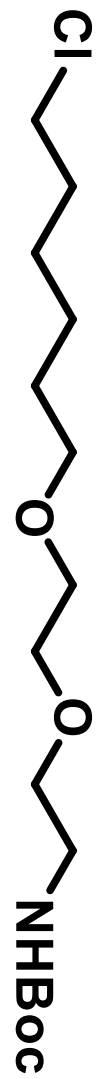

44

Figure S93. HRMS-ESI spectra of compound **44**.

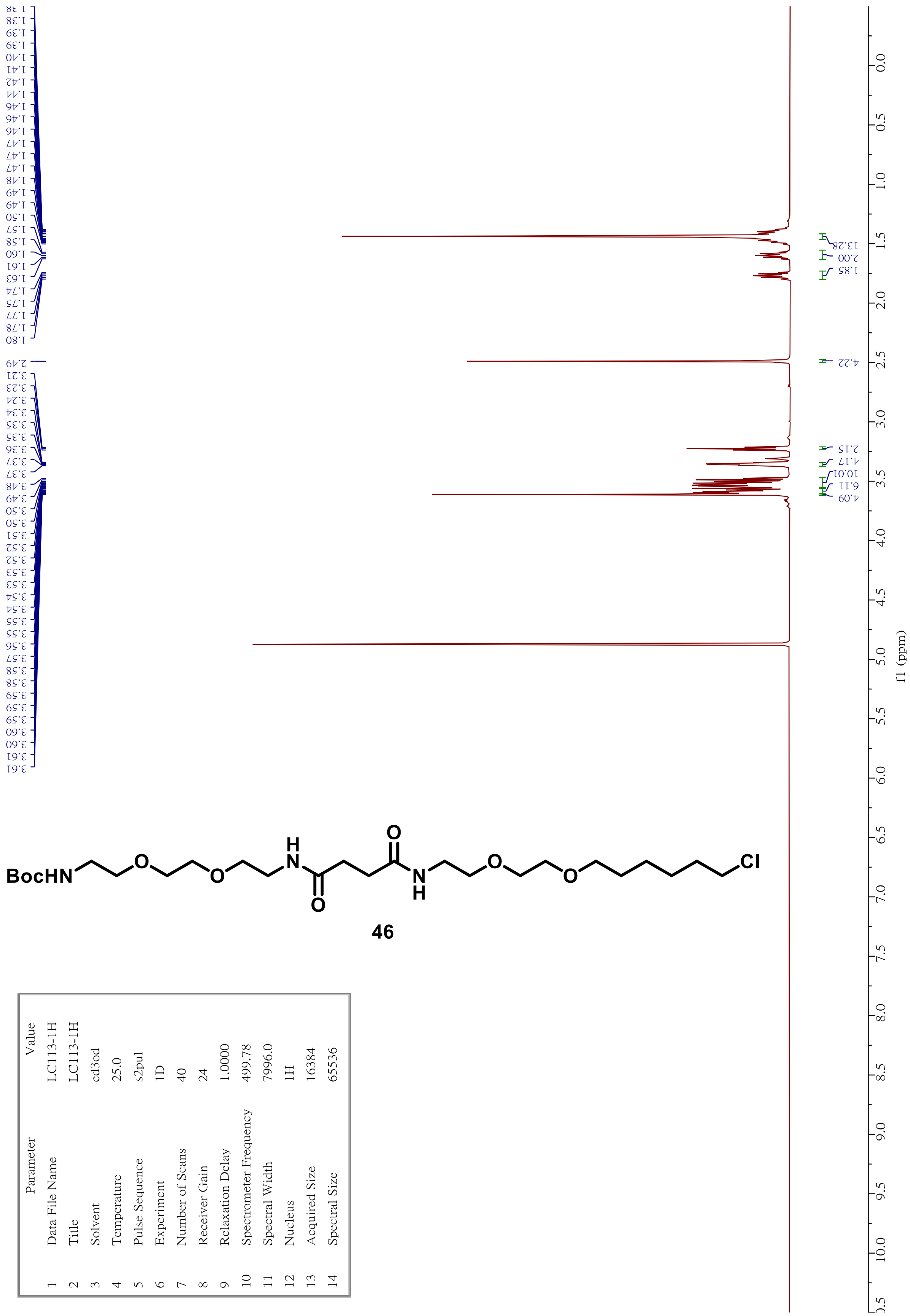

Figure S94. ^{1}H NMR spectra of compound **46**.

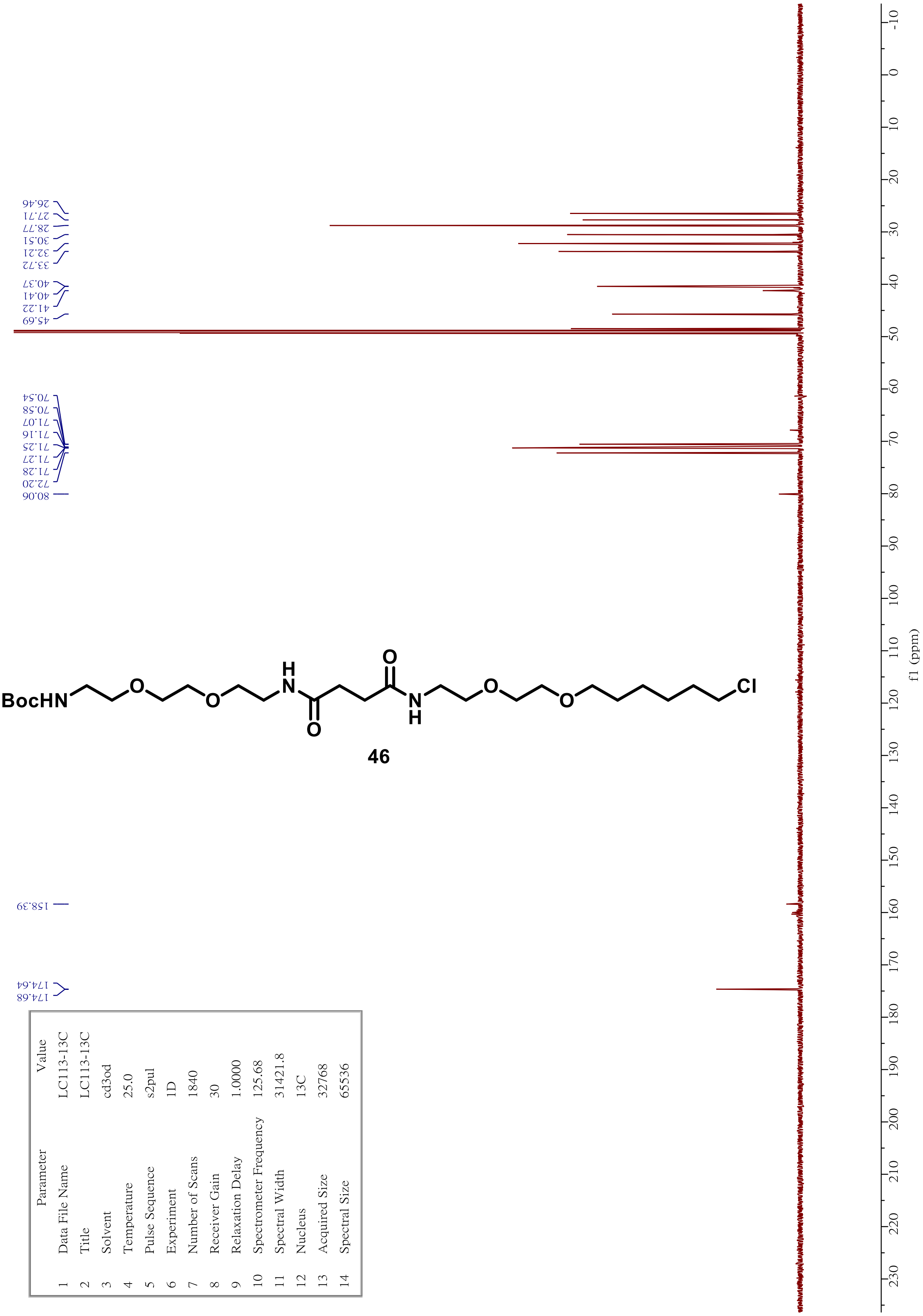

Figure S95. ^{13}C NMR spectra of compound **46**.

Data:LC113
Comment:
Description:
Ionization Mode:ESI+
History:Average(MS[1] 0.20..0.26)

Acquired:2/22/2023 3:42:05 PM
Operator:AccuTOF
m/z Calibration File:20230112-TFANa_...
Created:2/22/2023 3:48:44 PM
Created by:AccuTOF

Charge number:1
Tolerance:200.00[ppm], 250.00 .. 250....
Unsaturation Number:-100.5 .. 200.0 (...
Element:^{12}C:25 .. 25, ^{1}H:0 .. 49, ^{35}Cl:1 .. 1, ^{14}N:3 .. 3, ^{23}Na:0 .. 1, ^{16}O:8 .. 8

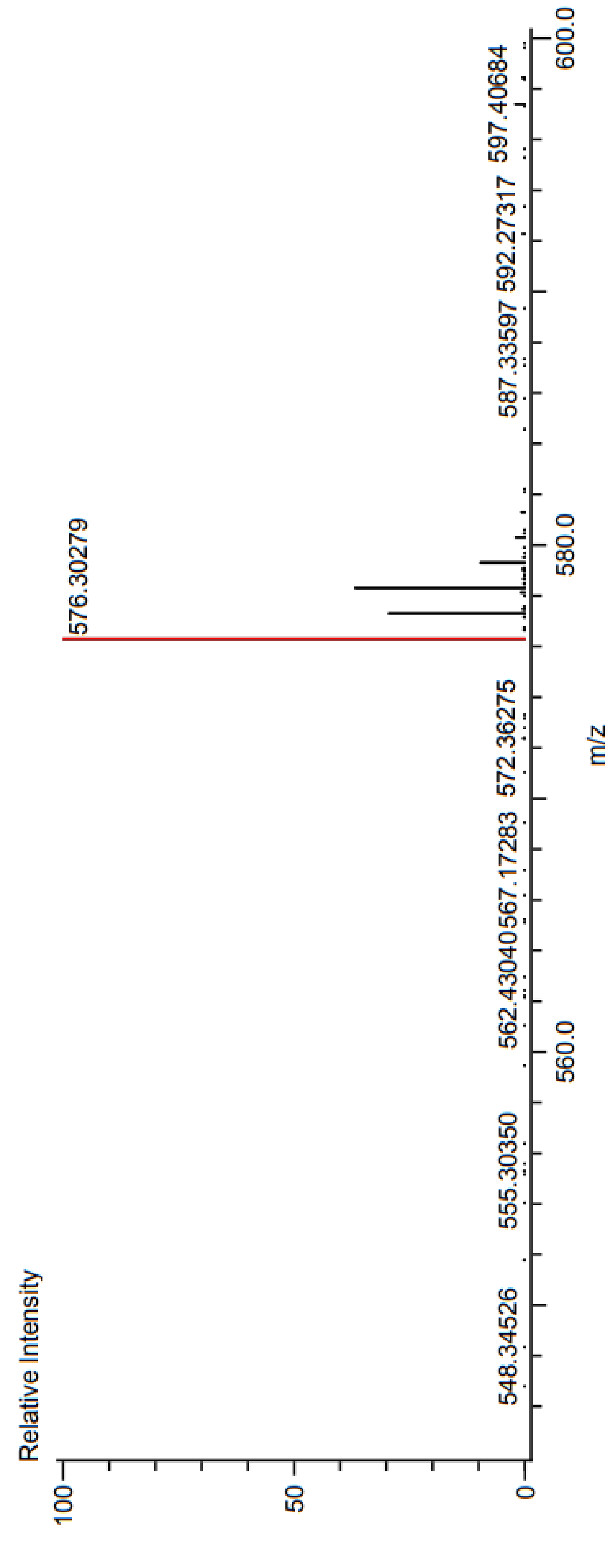

Mass	Intensity	Calc. Mass	Mass Difference [mDa]	Mass Difference [ppm]	Possible Formula
576.30279	92545.01	576.30276	0.03	0.05	$^{12}C_{25}{}^{1}H_{48}{}^{35}Cl_{1}{}^{14}N_{3}{}^{23}Na_{1}{}^{16}O_{8}$

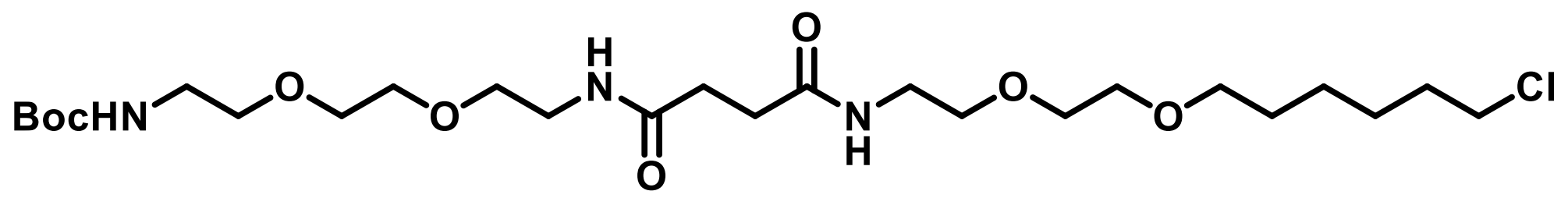

46

Figure S96. HRMS-ESI spectra of compound **46**.

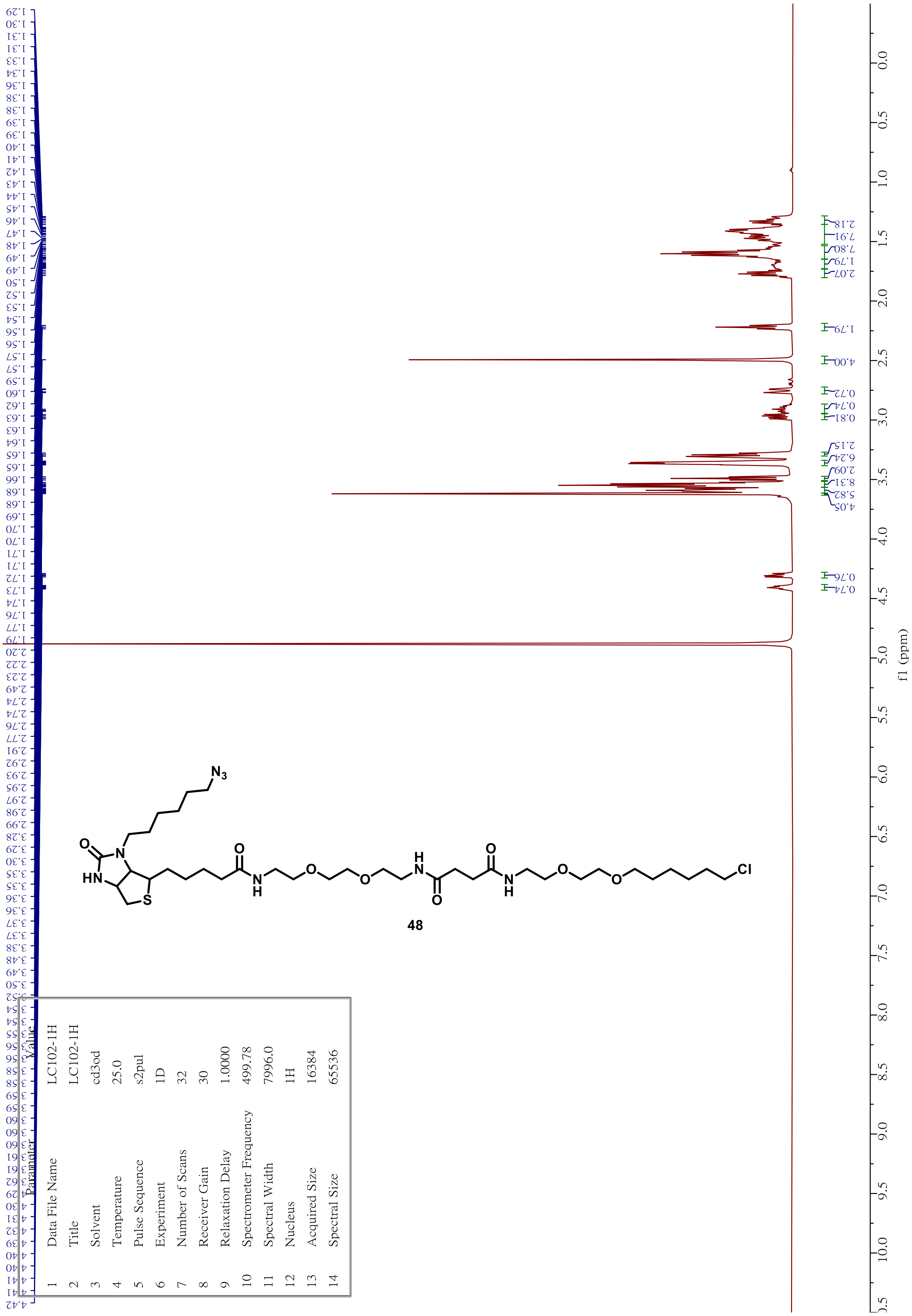

Figure S97. ^{1}H NMR spectra of compound **48**.

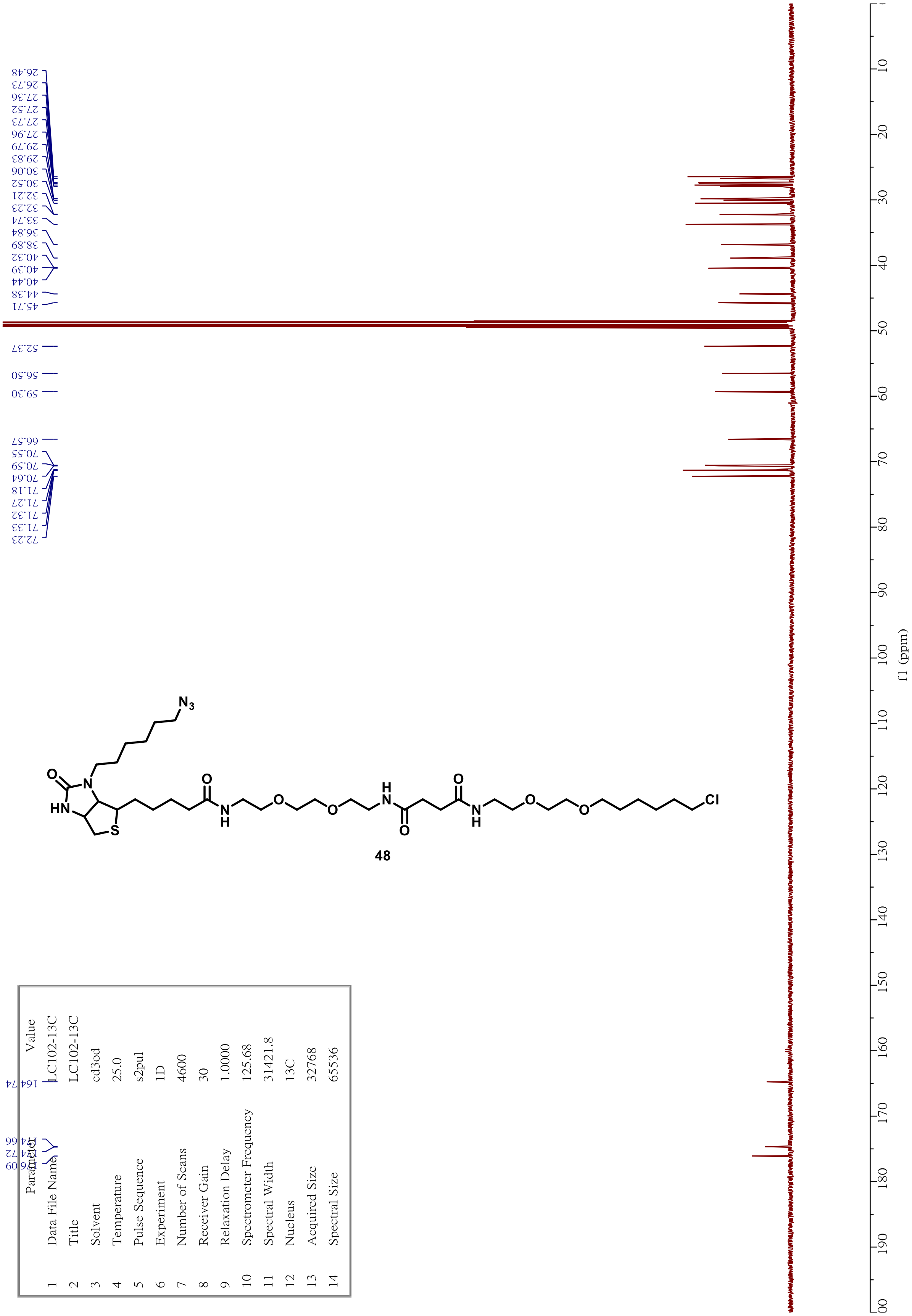

Figure S98. ^{13}C NMR spectra of compound **48**.

Data:LC102
Comment:
Description:
Ionization Mode:ESI+
History:Average(MS[1] 0.24..0.27)

Acquired:12/29/2022 11:47:57 AM
Operator:AccuTOF
m/z Calibration File:20221220-TFANa_...
Created:12/29/2022 2:01:13 PM
Created by:AccuTOF

Charge number:1
Tolerance:300.00[ppm], 50.00 .. 50.00[...
Unsaturation Number:-100.0 .. 200.0 (...
Element:^{12}C:36 .. 36, ^{1}H:0 .. 66, ^{35}Cl:1 .. 1, ^{14}N:8 .. 8, ^{23}Na:0 .. 1, ^{16}O:8 .. 8, ^{32}S:1 .. 1

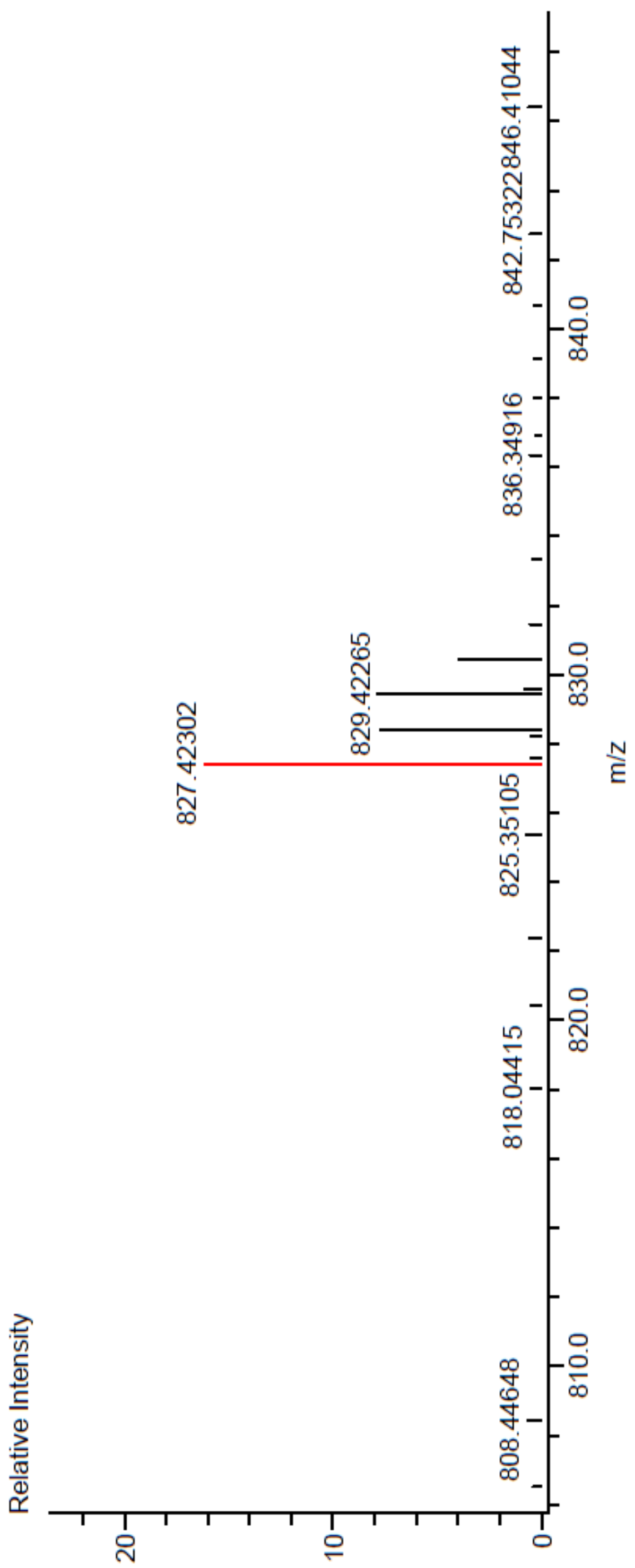

Mass	Intensity	Calc. Mass	Mass Difference [mDa]	Mass Difference [ppm]	Possible Formula
827.42302	5306.12	827.42323	-0.20	-0.24	$^{12}C_{36}{}^{1}H_{65}{}^{35}Cl_{1}{}^{14}N_{8}{}^{23}Na_{1}{}^{16}O_{8}{}^{32}S_{1}$

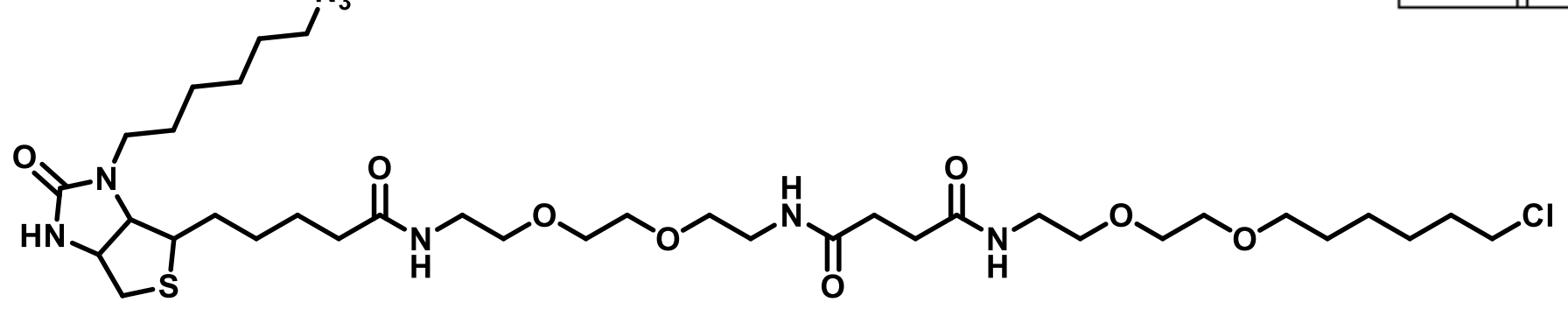

48

Figure S99. HRMS-ESI spectra of compound **48**.

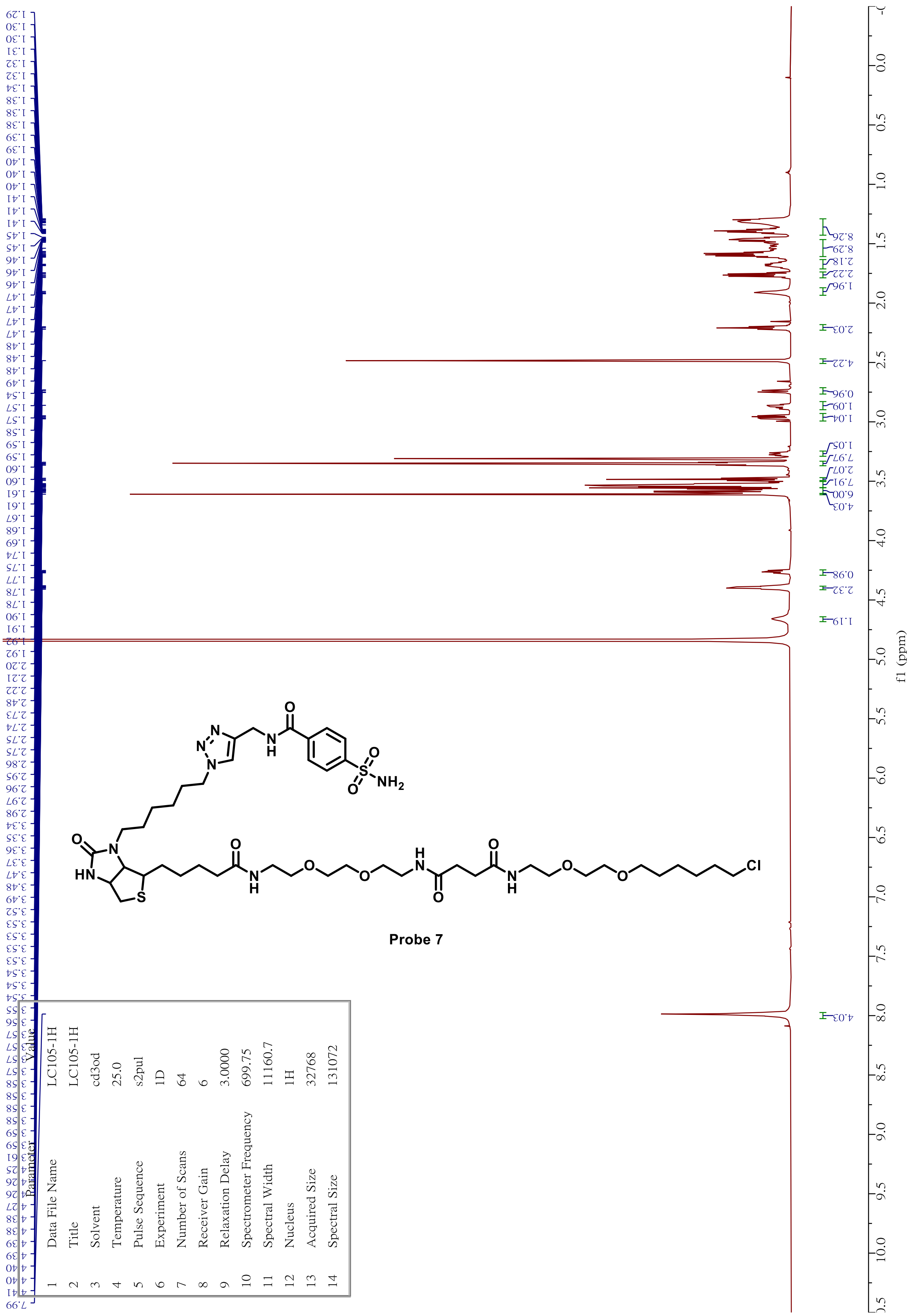

Figure S100. ^{1}H NMR spectra of **Probe 7**.

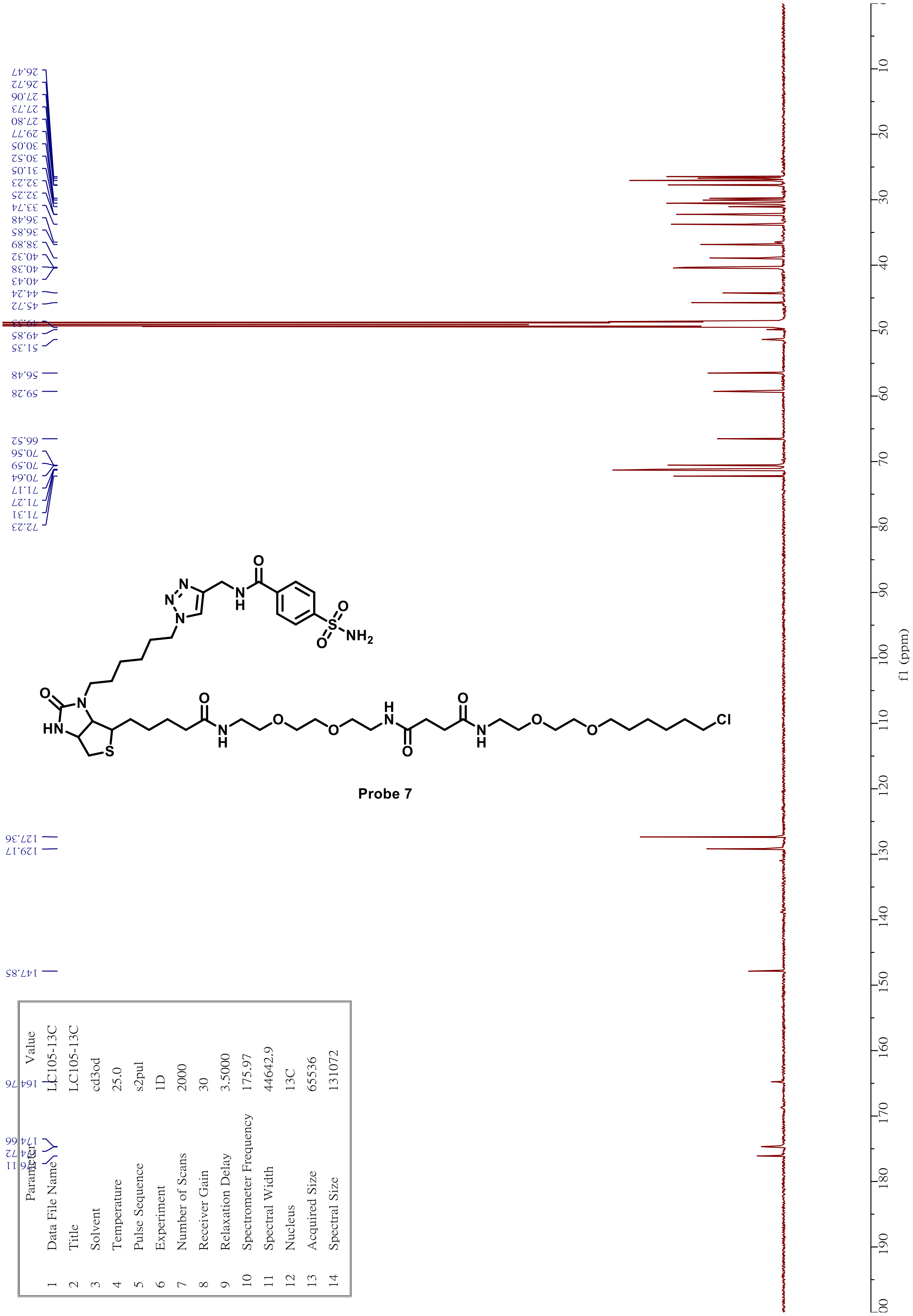

Figure S101. ^{13}C NMR spectra of **Probe 7**.

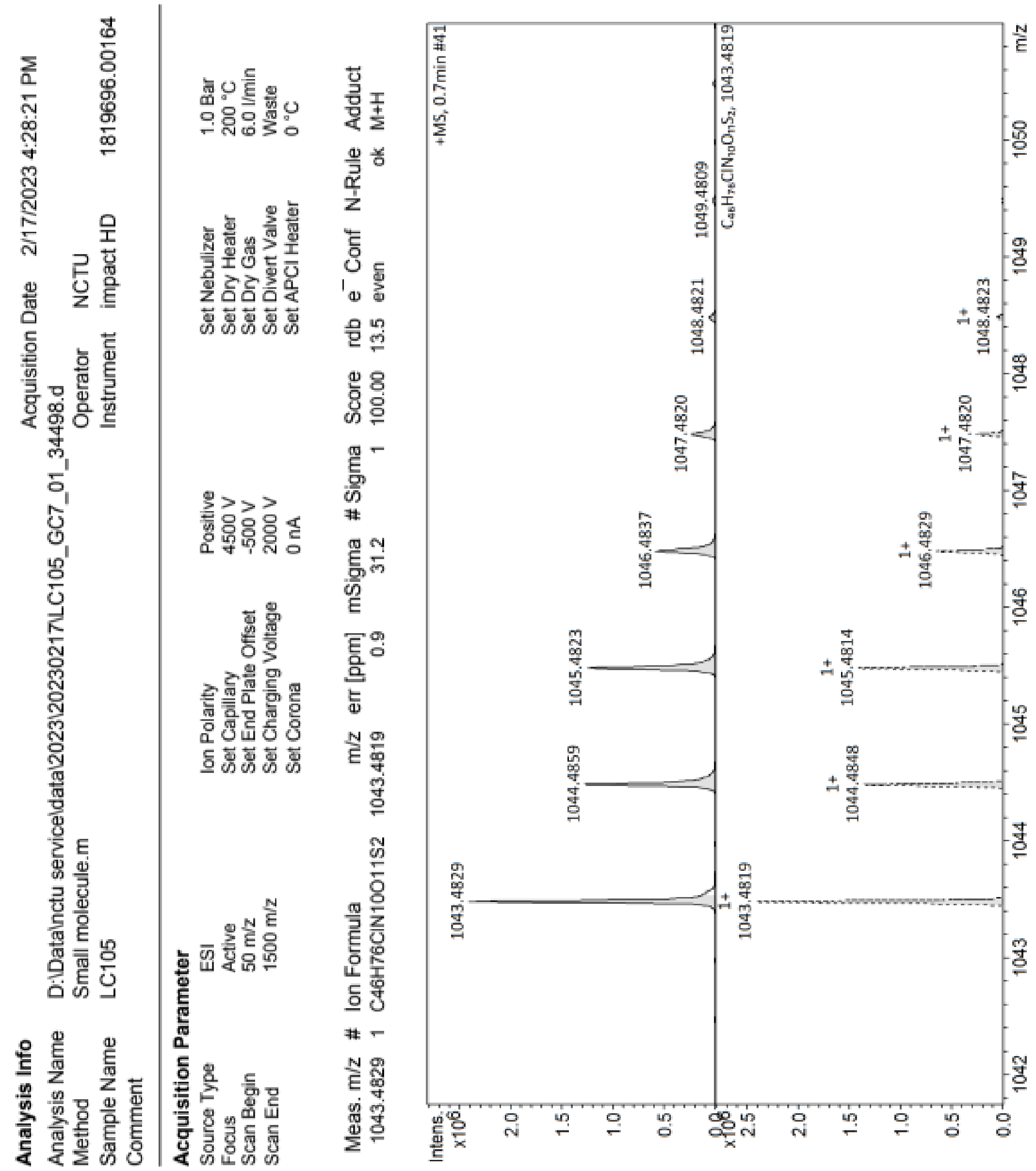

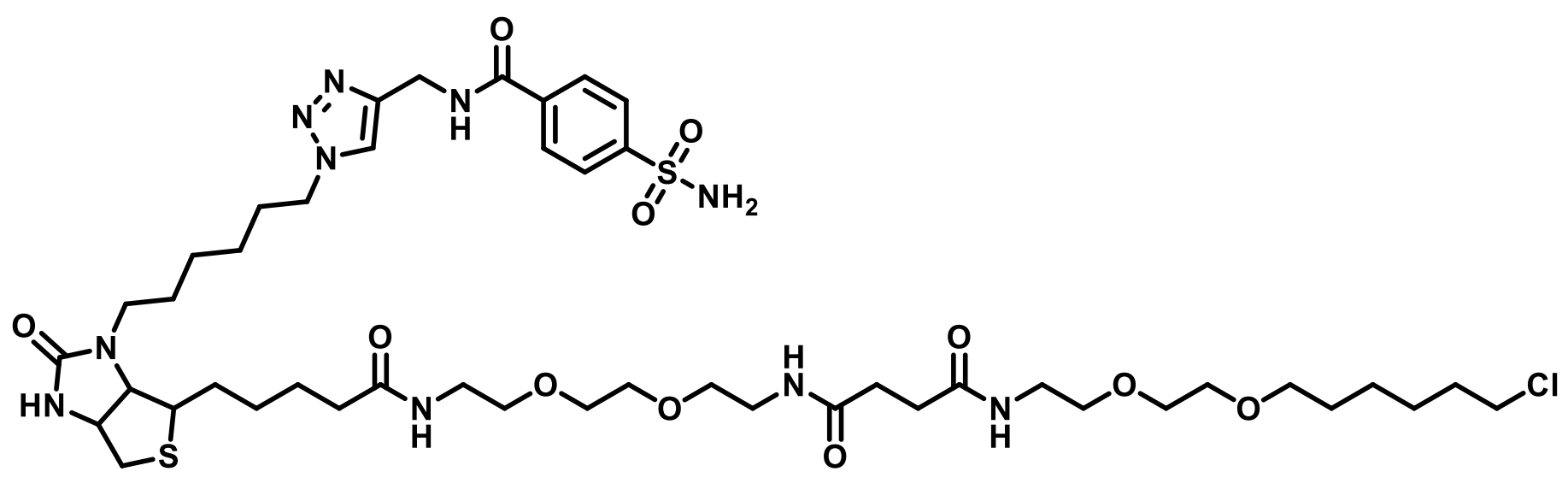

Probe 7

Figure S102. HRMS-ESI spectra of **Probe 7**.
